# Direct Fluoroformylation of the *C3*‐Position of Indoles with 2,4‐Dinitro(trifluoromethoxy)benzene as Fluorocarbonyl Source

**DOI:** 10.1002/open.202500563

**Published:** 2026-02-28

**Authors:** Lilian Wisson, Gilles Hanquet, Fabien Toulgoat, Thierry Billard, Frédéric R. Leroux, Armen Panossian

**Affiliations:** ^1^ Université de Strasbourg, Université de Haute‐Alsace CNRS, LIMA UMR 7042 Strasbourg France; ^2^ CPE Lyon Campus LyonTech‐La Doua 43 Bd du 11 novembre 1918 Villeurbanne France; ^3^ Institute of Chemistry and Biochemistry (ICBMS–UMR CNRS5246) CNRS Université Lyon1, CPELyon 1 rue Victor Grignard Lyon France

**Keywords:** 2,4‐dinitro‐1‐(trifluoromethoxy)benzene, acyl fluoride, fluoroformylation, fluorophosgene, indole

## Abstract

We herein report the direct fluoroformylation of indoles and other heteroaromatic cycles. Acyl fluorides are very useful moieties in coupling reactions with or without metal. However, they are usually obtained from the corresponding carboxylic acids or from aryl halides in pallado‐catalyzed carbonylation/fluorination reactions. Our method uses fluorophosgene generated in situ from 2,4‐dinitro(trifluoromethoxy)benzene (DNTFB) as a fluoroformylating agent without any metal and from carboxylic acid‐free heteroaromatic rings. Moreover, our method can be telescoped with amidification reactions in a one‐pot process.

## Graphical abstract

Indoles bearing acyl fluoride moieties are generally obtained by dehydroxyfluorination of carboxylic acids or by fluoroformylation mediated by palladium catalysts with the disadvantage of requiring toxic reagents or very high pressures. We describe herein the direct, metal‐free, and rapid C3‐fluoroformylation of electro‐enriched indoles using fluorophosgene generated *in situ* by the degradation of trifluoromethoxide anion obtained from 2,4‐dinitro‐1‐(trifluoromethoxy)benzene (DNTFB).

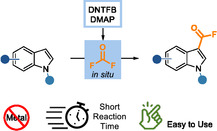

## 1 Introduction

Acyl fluorides (R‐COF) are a topic of interest in synthetic chemistry. Indeed, these carboxylic derivatives show a better stability toward hydrolysis [1, 2, 3], alcoholysis [2, 3], or aminolysis [4] than other acyl halides, making them easier to handle. Several syntheses of R‐COF have been developed from carboxylic acids by dehydroxylative fluorination, from acyl halides or carboxylic anhydrides by exchange with fluoride, by metal‐catalyzed fluorination of aryl halides with carbonyl insertion or from aldehydes by hypervalent bromine‐mediated or radical‐based oxidative fluorination [5, 6, 7, 8, 9, 10, 11]. Although acyl fluorides are more stable than other halogenated equivalents, they remain reactive enough to be used as substrates, such as in peptide synthesis [12, 13, 14, 15, 16] and transition metal‐catalyzed coupling reactions [6, 16, 17, 18, 19, 20]. Indoles, on the other hand, are essential scaffolds in pharmaceuticals [21, 22] and agrochemicals [23, 24]. Many active molecules contain indole scaffolds that are functionalized in the *C3* position. The introduction of an acyl fluoride at this position could therefore be of great interest from a synthesis perspective. In the literature, only two examples of *C3*‐fluoroformylated indoles have been reported; however, they required the use of harmful reagents, expensive and exotic palladium catalysts or very high pressures which limit their applicability [25, 26]. We present here a new, inexpensive, and easy‐to‐use method for the direct fluoroformylation of indoles to access indole‐3‐carbonyl fluoride scaffolds.

**SCHEME 1:**
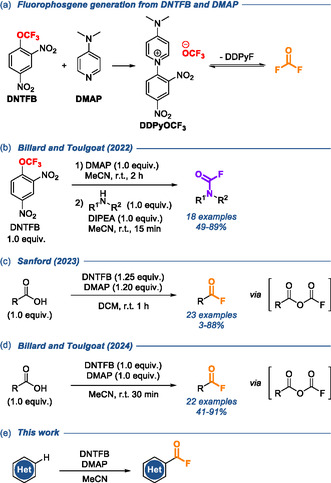
The use of DNTFB and DMAP for (a) the generation of fluorophosgene and the fluoroformylation of (b) amines, (c,d) carboxylic acids, and (e) heteroaromatic rings.

Recently, after using the combination of 2,4‐dinitro(trifluoromethoxy)benzene (DNTFB) and 4‐(dimethylamino)pyridine (DMAP) as trifluoromethoxide anion source [27, 28, 29, 30], our groups reported its use as a source of fluorophosgene (COF_2_) for the synthesis of carbamoyl fluorides with good to excellent yields (Scheme 1b) [31–33], This method is based on the degradation of the trifluoromethoxide anion (CF_3_O^–^) into COF_2_ and fluoride (Scheme 1a) and thus avoids the direct handling of toxic fluorophosgene to introduce the COF moiety on amines. Additionally, DNTFB and DMAP are inexpensive and easy‐to‐use reagents [28, 29]. During the latter project, we observed that the carboxylic acid part of ciprofloxacin underwent conversion into the corresponding acyl fluoride [31]. Therefore, a similar method was applied concomitantly by Sanford et al. [34] (Scheme 1c) and our groups [35] (Scheme 1d) to convert carboxylic acids into acyl fluorides [36] with excellent yields, without using polyfluorosulfur‐based reagents nor hydrofluoric acid. After employing DNTFB and DMAP in *N*‐ and *O*‐fluoroformylation, we were naturally interested in *C*‐fluoroformylation. We chose indoles as substrates due to the reasons mentioned above and due to their good *C*‐nucleophilicity.

## 2 Results and Discussion

On the basis of these methods, we investigated the direct fluoroformylation of 1‐methylindole **1a** using the conditions we developed for the trifluoromethoxylation of arynes [37] employing a large excess of DMAP and even larger excess of DNTFB (Table 1, entry 1). A complete conversion of the starting indole 1a to the desired 1‐methyl‐1*H*‐indole‐3‐carbonyl fluoride **2a** was obtained, with a good ^1^H NMR yield of 84%. The importance of the 2:1 ratio between DNTFB and DMAP was highlighted by the attempt to reduce it to 1:1, in order to increase the rate of degradation of trifluoromethoxide anion into fluorophosgene [28], which surprisingly resulted in the absence of conversion of **1a** (entry 2). Increasing the amount of DNTFB and DMAP used in a 1:1 ratio led to the full conversion of the starting material, but no fluoroformylated indole was observed (see Table S1). An intermediate 1.3:1 ratio gave a complete conversion but a lower yield (see Table S1). Going back to a 2:1 ratio and after reducing the amount of reagents and the reaction time (Table 1, entries 3–5), and after further optimization (full optimization available in Table S1), we obtained **2a** with a NMR yield of 91% using 2.0 equivalents of DNTFB and 1.0 equivalent of DMAP in 1.5 mL of reaction grade acetonitrile (MeCN) at 80°C for 1 h, affording a 84% yield after purification (Table 1, entry 5). Noteworthily, an additional decrease of the amount of DNTFB and DMAP, but still maintaining a 2:1 ratio, led to incomplete conversion but a good 85% yield (entry 6).

**TABLE 1:** Optimization of reaction conditions.^a^

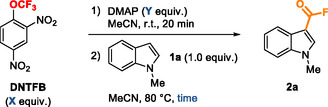				
Entry	X	Y	Time	Yield (**2a** **, %)** b
1	18.1	9.1	16 h	84% (80%)
2	2.1	2.1	16 h	No conversion
3	4.0	2.0	16 h	85%
4	4.0	2.0	1 h	85%
5^c^	2.0	1.0	1 h	91% (84%)
6	1.0	0.5	70 min	85%^d^

a Reaction conditions: DNTFB (X equiv.) and DMAP (Y equiv.) were mixed in anhydrous MeCN (5 mL) under argon for 20 min at r.t. before the addition of **1a** (0.50 mmol) and heating at 80°C.

b Yields were determined by ^1^H NMR with toluene as internal standard. Isolated yields in parentheses.

c Reaction carried out without premixing of DNTFB and DMAP, with reaction grade MeCN (1.5 mL), and under air.

d Conversion = 85%.

**SCHEME 2:**
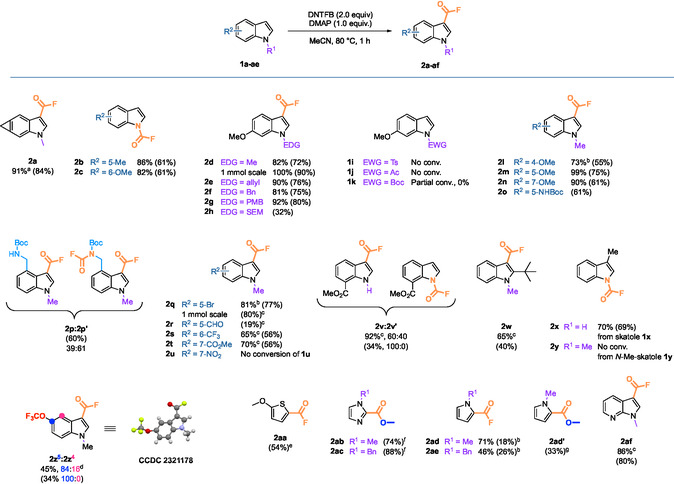
Substrate scope of the fluoroformylation with DNTFB/DMAP. Yields were calculated by ^1^H NMR analysis with bromochloromethane as internal standard. Isolated yields are given in parentheses. ^a^Toluene was used as internal standard. ^b^2 h reaction time. ^c^DNTFB (6.0 equiv.), DMAP (3.0 equiv.), 80°C, 2 h. ^d^Synthesized from the corresponding aryne precursor following the previously reported aryne trifluoromethoxylation procedure[37] ^e^DNTFB (6.0 equiv.), DMAP (3.0 equiv.), 80°C, 16 h. ^f^In situ esterification by addition of methanol on the concentrated crude mixture. ^g^In situ esterification by addition of methanol and triethylamine on the concentrated crude mixture.

**SCHEME 3:**
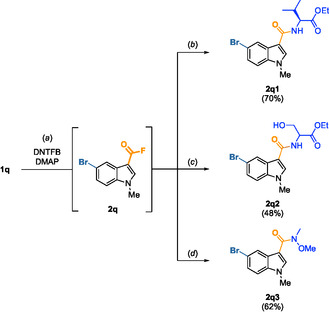
One‐pot, two‐step fluoroformylation and amidification of **1q**. (a) DNTFB (2.0 equiv.), DMAP (1.0 equiv.), MeCN, 80°C, 2 h. (b) Ethyl L‐valinate hydrochloride (3.2 equiv.), KOH (7.0 equiv.), MeCN, 80°C, 18 h. (c) Ethyl‐DL‐serinate hydrochloride (3.2 equiv.), NEt_3_ (7.0 equiv.), MeCN, 80°C, 18 h. (d) *N*‐Methoxymethylamine hydrochloride (3.2 equiv.), KOH (12.0 equiv.), DCM, 80°C, 18 h.

**SCHEME 4:**
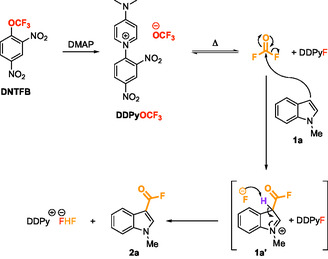
Mechanism of the fluoroformylation of **1a**.

With these conditions in hand, we conducted a study on the scope of the reaction (Scheme 2). We first investigated the influence of the protecting group of the indole nitrogen atom. When indoles were unprotected, the corresponding carbamoyl fluorides were obtained: **2b** with 86% ^1^H NMR yield and 61% isolated yield, and **2c** with 82% ^1^H NMR yield and 61% isolated yield. With the electron‐poor unprotected indole **1v**, 92% of a 60:40 mixture of acyl fluoride **2v** and carbamoyl fluoride **2v**′ was observed by ^1^H NMR analysis. After purification, **2v** was isolated as the unique product with 34% yield. 6‐Methoxyindoles *N‐*protected with electron‐donating groups (EDG) gave moderate to excellent yields. Indeed, whereas the *N*‐SEM protected product **2h** was obtained in 32% yield only, the *N*‐methyl derivative **2d** was isolated with a more satisfying 72% yield which could be improved to 90% on larger scale, while the *N*‐allyl, *N*‐benzyl, and *N*‐*para‐*methoxybenzyl protected derivatives (**2e**, **2f** and **2g** respectively) were obtained with yields of 75%–80% after purification. However, unsurprisingly, indoles that were *N*‐protected with electron‐withdrawing groups (EWG) **1i** and **1j** remained unconverted due to the strong decrease of nucleophilicity. *N*‐Boc‐protected substrate **1k** did not lead to any product **2k** despite a partial conversion observed by ^1^H NMR (Scheme 3). This could be explained by a degradation of the starting indole in the acidic medium formed during the reaction (Scheme 4).

Substituents of the *N*‐methylated indole core were also varied. Indoles bearing electron‐donating substituents on various positions of the 6‐membered ring reacted to afford acyl fluorides **2l‐o** with yields ranging from 55% to 75%. In the case of *N*‐Boc‐protected 4‐(aminomethyl) substrate **1p**, a mixture of mono‐ and bis‐fluroformylated indoles **2p** and **2p**′ was obtained with a 39:41 ratio and 60% yield after isolation. This indicates that primary amines must be fully protected to prevent the formation of carbamoyl fluoride in addition to the fluoroformylation of the *C3* position of the indole. Interestingly, such *N*‐Boc‐protected carbamoyl fluorides are, to the best of our knowledge, unprecedented. Pursuing the study of the scope of the fluoroformylation reaction, deactivating groups were also assessed. Longer reaction times or more DNTFB and DMAP equivalents were needed to reach full conversions and good yields for **2q**,**s–t**. Although the aldehyde moiety was partially tolerated as **2r** was obtained with a moderate yield of 19% explained by deoxyfluorination side‐reactions, the highly deactivating nitro group gave no conversion in our conditions. It is also worth‐mentioning that 5‐bromo‐1‐methyl‐1*H*‐indole‐3‐carbonyl fluoride **2q**, displaying a functionalizable C–Br bond was also obtained with a good yield of 80% on a 1.0 g scale using 6.0 equivalents of DNTFB, 3.0 equivalents of DMAP and a reaction time of 2 h.

Steric hindrance had a negative impact on the reaction. 4‐Methoxy‐1‐methyl‐1*H*‐indole‐3‐carbonyl fluoride **2l** was isolated with 55% yield, which was lower than for indoles substituted by methoxy groups on other positions of the carbocycle (**2d, m–n**). Although the yield difference was not always large, unlike its congeners, the isolated product **2l** showed a poor stability at room temperature and degraded into the corresponding carboxylic acid after 24 h. A similar observation was made with the 2‐(*tert*‐butyl)‐substituted product **2w** which not only required 6.0 equivalents of DNTFB, 3.0 equivalents of DMAP and 2 h of reaction to be formed with a 40% yield, but also proved unstable at room temperature, degrading to give back the starting product **1w**. We hypothesized a hydrolysis of the acyl fluoride to the corresponding carboxylic acid followed by a decarboxylation of the latter. When the *C3* position was substituted, fluoroformylation did not occur, as observed with *N*‐methylated skatole **1y**. However, when skatole was used, the corresponding carbamoyl fluoride was obtained with 70% ^1^H NMR yield and 69% isolated yield.

Finally, we wondered if it would be possible, with the DNTFB/DMAP system, to carry out at the same time the fluoroformylation and the trifluoromethoxylation [37] of an indolyne derivative. Gratifyingly, indoles **2z** ^**5**^ and **2z** ^**4**^, which differ by the position of the OCF_3_ group due to the two electrophilic positions of the aryne intermediates, were formed in a moderate 45% ^19^F NMR yield and an 84:16 regioisomeric ratio of the 5‐ and 4‐OCF_3_ products, respectively. We isolated the 5‐OCF_3_ isomer **2z** ^**5**^ with 34% yield and its structure was confirmed by X‐ray diffraction crystallography (CCDC number 2 321 178).

Other heteroaromatic cycles underwent fluoroformylation. A good yield of 54% was obtained for 5‐methoxythiophene‐2‐carbonyl fluoride **2aa**. However, due to its lower reactivity compared with indoles, it required more equivalents of DNTFB and DMAP and an overnight reaction time. Unfortunately, we were unable to isolate fluoroformylated imidazoles due to hydrolysis during the aqueous workup. Therefore, we derivatized them into the corresponding methyl esters by stirring concentrated crude reaction mixtures in methanol. Using this method, we were able to isolate methyl 1‐methyl‐1*H*‐imidazole‐2‐carboxylate **2ab** and methyl 1‐benzyl‐1*H*‐imidazole‐2‐carboxylate **2ac** with excellent yields of 74% and 88% respectively. *N*‐Methyl and *N*‐benzyl pyrroles gave corresponding acyl fluorides **2ad** and **2ae** with 71% and 46% NMR yields. They were isolated in modest yields of 18% and 26% respectively. The difference between NMR and isolated yields of **2ad** and **2ae** could be attributed to the instability of the product during workup or the column. To overcome this issue, the derivatization method was employed with *N*‐methylpyrrole to give a modest isolated yield of 33% of methyl 1‐methyl‐1*H*‐pyrrole‐2‐carboxylate **2ad**′ after treatment of the concentrated crude with methanol and triethylamine. Finally, we obtained 1‐methyl‐1*H*‐pyrrolo[2,3‐*b*]pyridine‐3‐carbonyl fluoride **2af** with an excellent yield of 80%. However, 3‐bromofuran, benzofuran, and benzothiophene did not undergo conversion, indicating insufficient nucleophilicity to react with fluorophosgene under our conditions (refer to Supporting Information for further details). Electronically enriched aromatic rings such as *N*,*N*‐dimethylaniline or 1,3,5‐trimethoxybenzene were not reactive either under our conditions.

Acyl fluorides are often used in peptide synthesis [9, 12, 13, 14, 15, 16], which encouraged us to merge our new method with amidification reactions. The amidification protocol was inspired by the work of Qin et al*.* [38] who used in situ generated acyl fluorides to synthesize amide bonds with primary and secondary amines as nucleophiles in the presence of KOH. To take into account the reaction between amino acids and 2,4‐dinitrofluorobenzene (DNFB, Sanger's reagent) [39], a side‐product of the fluoroformylation reaction, we had to use high excesses of amine and base. Therefore, we performed the fluoroformylation of **1q** followed by an amide bond formation in a one‐pot, two‐step procedure (Scheme 3). Valine and serine derivatives produced **2q1** and **2q2** with 70% and 48% yields respectively. Triethylamine was used as the base with the serine derivative to avoid transesterification reactions occurring when KOH was employed. Similarly, we obtained the analogous Weinreb amide **2q3** with a good yield of 62%. These results along with the esterification reaction used to obtain **2ab** and **2ac** demonstrate the compatibility of our method with nucleophilic additions on the formed acyl fluorides in one‐pot strategies.

Finally, the plausible mechanism of the reaction is depicted in Scheme 4. After formation of the DDPyOCF_3_ salt from DNTFB and DMAP, fluorophosgene is generated in situ by the degradation of the CF_3_O^–^ ion. An aromatic electrophilic substitution (S_E_Ar) by fluorophosgene then takes place at the nucleophilic *C3* carbon of the indole. The indolium intermediate formed is then deprotonated to restore the aromaticity of the molecule. Control experiments were conducted (see Supporting Information for more details) and confirmed that the fluoride anion was able to play the role of the proton trap, which concurs with the findings of Sanford et al. [34] and Zhang et al. [32, 33, 36] and that fluorophosgene alone is able to mediate the fluoroformylation reaction. However, a sufficient activation of DNTFB is required to obtain fluoroformylated heteroaromatic rings in good yields.

## 3 Conclusion

In conclusion, we have developed a new, easy‐to‐use, and cheap method for the direct fluoroformylation of indoles on the *C3* position using the combination of DNTFB and DMAP as fluoroformylating system. The method is safer than the direct use of gaseous fluorophosgene, a toxic gas, which is here produced in situ in controlled amount. This method is compatible with both electron‐donating groups on the nitrogen atom or the benzene ring of indoles, and with electron‐withdrawing groups on the arene ring, resulting in fluoroformylated indoles with good to excellent yields. However, protecting the nitrogen atom of the indole core with an electron‐withdrawing group prevented conversion while leaving it unprotected resulted in the formation of the corresponding carbamoyl fluorides unless an electron‐poor ring was used. This strategy has also been successfully applied to other heteroaromatic cycles, such as pyrroles, imidazoles or thiophenes. Additionally, the mild process was tolerated by acid‐sensitive functions, and esterification and amidification reactions were carried out in a one‐pot, two‐step process starting from the *C3*‐H indole with good overall yields.

## Supporting Information

Additional supporting information can be found online in the Supporting Information Section. The authors have cited additional references within the Supporting Information [40, 41, 42, 43, 44, 45, 46, 47, 48, 49, 50, 51, 52, 53, 54, 55, 56, 57, 58, 59, 60, 61, 62, 63, 64, 65, 66, 67, 68, 69, 70]. **Supporting Table S1:** Optimization of the fluoroformylation of 1‐methylindole **1a**. **Supporting Table S2:** Specific rotation measurements for a solution of **2** **q** **1** in MeOH. **Supporting Table S3:** Atomic coordinates (× 10^4^) and equivalent isotropic displacement parameters (Å^2^ × 10^3^) for **2z^5^**. U(eq) is defined as one third of the trace of the orthogonalized Uij tensor. **Supporting Table S4:** Bond lengths [Å] and angles [°] for **2z^5^**. **Supporting Table S5:** Anisotropic displacement parameters (Å^2^ × 10^3^) for **2z^5^**. The anisotropic displacement factor exponent takes the form: ‐2 pi^2^ [ h^2^ a*^2^ U11 + … + 2 h k a* b* U12 ]. **Supporting Table S6:** Hydrogen coordinates (× 10^4^) and isotropic displacement parameters (Å^2^ × 10^3^) for **2z^5^**. **Supporting Table S7:** Torsion angles [°] for **2z^5^**.

## Funding

This study was supported by Agence Nationale de la Recherche (grant ANR‐ 20‐CE07‐0004‐02 (Ap‐PET‐I)).

## Conflicts of Interest

The authors declare no conflicts of interest.

## Supporting information

Supplementary Material

## Data Availability

The data that support the findings of this study are available in the supplementary material of this article.

## References

[1] C. G. Swain and C. B. Scott , “Rates of Solvolysis of Some Alkyl Fluorides and Chlorides,” Journal of the American Chemical Society 75 (1953): 246.

[2] B. D. Song and W. P. Jencks , “Mechanism of Solvolysis of Substituted Benzoyl Halides.,” Journal of the American Chemical Society 111 (1989): 8470.

[3] D. N. Kevill and M. J. D’Souza , “Correlation of the Rates of Solvolysis of Benzoyl Fluoride and a Consi deration of Leaving‐Group Effects,” The Journal of Organic Chemistry 69 (2004): 7044. 15471451 10.1021/jo0492259

[4] M. L. Bender and J. M. Jones , “Nucleophilic Reactions of Morpholine with the Benzoyl Halides. The Presence of an Element Effect,” The Journal of Organic Chemistry 27 (1962): 3771.

[5] F. Aldabbagh , “Acid Halides.” In Comprehensive Organic Functional Group Transformations II, (Elsevier, 2005). 1.

[6] Y. Ogiwara and N. Sakai , “Acyl Fluorides in Late‐Transition‐Metal Catalysis,” Angewandte Chemie International Edition 59 (2020): 574. 30969455 10.1002/anie.201902805

[7] M. Gonay , C. Batisse , and J.‐F. Paquin , “Recent Advances in the Synthesis of Acyl Fluorides,” Synthesis 53 (2021): 653.

[8] F. Pulikkottil , J. S. Burnett , J. Saiter , C. A. I. Goodall , B. Claringbold , and K. Lam , “eFluorination for the Rapid Synthesis of Carbamoyl Fluorides from Oxamic Acids,” Organic Letters 26 (2024): 6103. 39016380 10.1021/acs.orglett.4c01605 PMC11287745

[9] J. Lai , C. Bahri , M. P. Truong , K. T. Downey , and G. M. Sammis , “Rapid Peptide Synthesis using a Methylimidazolium Sulfinyl Fluoride Salt,” Communications Chemistry 8 (2025): 53. 39987317 10.1038/s42004-025-01456-8 PMC11846836

[10] E. M. Mahmoud , M. A. Marzouk , W. S. Shehab , D. A. Elsayed , T. S. Ibrahim , and I. M. Salem , “Triphosgene/KF‐Mediated acyl Fluoride Synthesis via in Situ Fluoro(chloro)phosgene,” Organic and Biomolecular Chemistry 23 (2025): 6116. 40454442 10.1039/d5ob00735f

[11] D. Zhang , P. Shen , Y. Zhang , et al., “A TFPN‐Mediated Acyl Fluoride Platform: Efficient Synthesis of Esters, Thioesters, and Macrolactones from Carboxylic Acids with Diverse Nucleophiles,” Organic Chemistry Frontiers 12 (2025): 5414.

[12] J.‐N. Bertho , A. Loffet , C. Pinel , F. Reuther , and G. Sennyey , “Amino Acid Fluorides: Their Preparation and use in Peptide Synthesis,” Tetrahedron Letters 32 (1991): 1303.

[13] L. A. Carpino , M. Beyermann , H. Wenschuh , and M. Bienert , “Peptide Synthesis via Amino Acid Halides,” Accounts of Chemical Research 29 (1996): 268.

[14] G. Karygiannis , C. Athanassopoulos , P. Mamos , et al., “Preparation and Properties of Enantiomerically Pure N(alpha)‐Tritylamino Acid Fluorides,” Acta Chemica Scandinavica 52 (1998): 1144.

[15] L. A. Carpino , D. Ionescu , A. El‐Faham , et al., “Complex Polyfluoride Additives in Fmoc‐Amino Acid Fluoride Coupling Pr ocesses. Enhanced Reactivity and Avoidance of Stereomutation,” Organic Letters 5 (2003): 975. 12659552 10.1021/ol020235a

[16] T. Tian , Q. Chen , Z. Li , and Y. Nishihara , “Recent Advances in C–F Bond Activation of Acyl Fluorides Directed toward Catalytic Transformation by Transition Metals, N‐Heterocyclic Carbenes, or Phosphines,” Synthesis 54 (2022): 3667.

[17] Y. Ogiwara , D. Sakino , Y. Sakurai , and N. Sakai , “Acid Fluorides as Acyl Electrophiles in Suzuki–Miyaura Coupling,” European Journal of Organic Chemistry 2017 (2017): 4324.

[18] N. Blanchard and V. Bizet , “Acid Fluorides in Transition‐Metal Catalysis: A Good Balance between Stability and Reactivity,” Angewandte Chemie International Edition 58 (2019): 6814. 30964591 10.1002/anie.201900591

[19] L. Fu , Q. Chen , and Y. Nishihara , “Recent Advances in Transition‐metal‐catalyzed C−C Bond Formation via C(sp^2^)−F Bond Cleavage,” Chemical Record 21 (2021): 3394. 33852203 10.1002/tcr.202100053

[20] L. V. Hooker and J. S. Bandar , “Synthetic Advantages of Defluorinative C−F Bond Functionalization,” Angewandte Chemie International Edition 62 (2023): e202308880. 37607025 10.1002/anie.202308880 PMC10843719

[21] T. V. Sravanthi and S. L. Manju , “Indoles — A Promising Scaffold for Drug Development,” European Journal of Pharmaceutical Sciences 91 (2016): 1. 27237590 10.1016/j.ejps.2016.05.025

[22] A. Dorababu , “Indole – A Promising Pharmacophore in Recent Antiviral Drug Discovery,” Rsc Medicinal Chemistry 11 (2020): 1335. 34095843 10.1039/d0md00288g PMC8126882

[23] A. Andreani and M. Rambaldi , “Indole Derivatives as Agrochemicals,” Journal of Heterocyclic Chemistry 25 (1988): 1519.

[24] P. Sun , Y. Huang , S. Chen , X. Ma , Z. Yang , and J. Wu , “Indole Derivatives as Agrochemicals: An Overview,” Chinese Chemical Letters 35 (2023): 109005.

[25] A. Boreux , K. Indukuri , F. Gagosz , and O. Riant , “Acyl Fluorides as Efficient Electrophiles for the Copper‐Catalyzed Boroacylation of Allenes,” Acs Catalysis 7 (2017): 8200.

[26] C. Yue , Q. Xing , P. Sun , Z. Zhao , H. Lv , and F. Li , “Enhancing Stability by Trapping Palladium Inside N‐Heterocyclic Carbene‐Functionalized Hypercrosslinked Polymers for Heterogeneous C‐C Bond Formations,” Nature Communications 12 (2021): 1875. 10.1038/s41467-021-22084-5 PMC7994585 33767184

[27] O. Marrec , T. Billard , J.‐P. Vors , S. Pazenok , and B. R. Langlois , “A New and Direct Trifluoromethoxylation of Aliphatic Substrates with 2,4‐Dinitro(trifluoromethoxy)benzene,” Advanced Synthesis & Catalysis 352 (2010): 2831.

[28] C. Bonnefoy , E. Chefdeville , A. Panossian , et al., “Study of a Stable “Trifluoromethoxide Anion Solution” Arising from 2,4‐Dinitro‐Trifluoromethoxybenzene,” Chemistry: A European Journal 27 (2021): 15986. 34496078 10.1002/chem.202102809

[29] G. Duran‐Camacho , D. M. Ferguson , J. W. Kampf , D. C. Bland , and M. S. Sanford , “Isolable Pyridinium Trifluoromethoxide Salt for Nucleophilic Trifluoromethoxylation,” Organic Letters 23 (2021): 5138. 34139121 10.1021/acs.orglett.1c01664

[30] C. Bonnefoy , A. Panossian , G. Hanquet , F. R. Leroux , F. Toulgoat , and T. Billard , “Comprehensive Study and Development of a Metal‐Free and Mild Nucleophilic Trifluoromethoxylation,” Chemistry: A European Journal 29 (2023): e202301513. 37278564 10.1002/chem.202301513

[31] C. Bonnefoy , E. Chefdeville , C. Tourvieille , et al., “Study of Carbamoyl Fluoride: Synthesis, Properties and Applications,” Chemistry: A European Journal 28 (2022): e202201589. 35639343 10.1002/chem.202201589

[32] Zhang et al. reported a similar method using trifluoromethyl triflate as the source of trifluoromethoxide anion, which collapses in situ into fluorophosgene; see: H. X. Song , Z. Z. Han , and C. P. Zhang, “Concise and Additive‐Free Click Reactions between Amines and CF_3_SO_3_CF_3_ ,” Chemistry: A European Journal 25 (2019): 10907. 31304646 10.1002/chem.201901865

[33] See also the related review and references cited therein: L. Liu , Y. C. Gu , and C. P. Zhang , “Recent Advances in the Synthesis and Transformation of Carbamoyl Fluorides, Fluoroformates, and Their Analogues,” Chemical Record 23 (2023): e202300071. 37098875 10.1002/tcr.202300071

[34] A. V. R. D. Lisboa , G. Duran‐Camacho , A. K. Ehrlacher , M. R. Lasky , and M. S. Sanford , “Deoxyfluorination of Carboxylic Acids via an In Situ Generated Trifluoromethoxide Salt,” Organic Letters 25 (2023): 9025. 38064366 10.1021/acs.orglett.3c03706 PMC10774922

[35] C. Bonnefoy , A. Gallego , C. Delobel , et al., “Unlocking the Power of Acyl Fluorides: A Comprehensive Guide to Synthesis and Properties,” European Journal of Organic Chemistry 27 (2024): e202400142.

[36] Once Again, Analogous Work Had Been Reported with Trifluoromethyl Triflate as the Source of Trifluoromethoxide and Fluorophosgene, See: X. Song , Z. Y. Tian , J. C. Xiao , and C. P. Zhang , “Tertiary‐Amine‐Initiated Synthesis of Acyl Fluorides from Carboxylic Acids and CF_3_SO_2_OCF_3_ ,” Chemistry: A European Journal 26 (2020): 16261, 32954583 10.1002/chem.202003756

[37] L. Wisson , G. Hanquet , F. Toulgoat , T. Billard , A. Panossian , and F. R. Leroux , European “Trifluoromethoxylation of Arynes using 2,4‐Dinitro‐1‐(trifluoromethoxy benzene) as Trifluoromethoxide Anion Source,” Journal of Organic Chemistry 27 (2024): e202400388.

[38] Q.‐X. Wu , T. Shu , W.‐Y. Fang , and H.‐L. Qin , “Discovery of KOH/BrCH_2_SO_2_F as Water‐Removable System for Clean, Mild and Robust Synthesis of Amides and Peptides,” European Journal of Organic Chemistry 2022 (2022): e202200719.

[39] F. Sanger , “The Free Amino Groups of Insulin,” The Biochemical Journal 39 (1945): 507. 16747948 10.1042/bj0390507 PMC1258275

[40] J. F. Stadlwieser and M. E. Dambaur , “Convenient Synthesis of 1H‐Indol‐1‐yl Boronates via Palladium(0)‐Catalyzed Borylation of Bromo‐1H‐indoles with ‘Pinacolborane’,” Helvetica Chimica Acta 89 (2006): 936.

[41] H. Ishikawa , D. A. Colby , S. Seto , et al., “Total Synthesis of Vinblastine, Vincristine, Related Natural Products, and Key Structural Analogues,” Journal of the American Chemical Society 131 (2009): 4904. 19292450 10.1021/ja809842b PMC2727944

[42] S. Potavathri , K. C. Pereira , S. I. Gorelsky , A. Pike , A. P. LeBris , and B. DeBoef , “Regioselective Oxidative Arylation of Indoles Bearing N‐Alkyl Protecting Groups: Dual C−H Functionalization via a Concerted Metalation−Deprotonation Mechanism,” Journal of the American Chemical Society 132 (2010): 14676. 20863119 10.1021/ja107159b PMC2954267

[43] S. Sar , A. Tripathi , K. D. Dubey , and S. Sen , “Iodine‐Catalyzed Aerobic Diazenylation–Amination of Indole Derivatives,” The Journal of Organic Chemistry 85 (2020): 3748. 32019297 10.1021/acs.joc.9b03392

[44] E. M. Galathri , L. D. Terlizzi , M. Fagnoni , S. Protti , and C. G. Kokotos , “Friedel–Crafts Arylation of Aldehydes with Indoles Utilizing Arylazosulfones as the Photoacid Generator,” Organic & Biomolecular Chemistry 21 (2023): 365. 36512428 10.1039/d2ob02214a

[45] E. G. L. Robert , V. Pirenne , M. D. Wodrich , and J. Waser , “Donor‐Acceptor Aminocyclobutane Monoesters: Synthesis and Silylium‐Catalyzed (4+2) Annulation with Indoles,” Angewandte Chemie International Edition 62 (2023): e202302420. 37074758 10.1002/anie.202302420

[46] A. Kasahara , T. Izumi , S. Murakami , K. Miyamoto , and T. Hino , “A Regiocontrolled Synthesis of Substituted Indoles by Palladium‐Catalyzed Coupling of 2‐Bromonitrobenzenes and 2‐Bromoacetanilides,” Journal of Heterocyclic Chemistry 26 (1989): 1405.

[47] J.‐L. Zhou , M.‐C. Ye , X.‐L. Sun , and Y. Tang , “Trisoxazoline/Cu(II)‐Catalyzed Asymmetric Intramolecular Friedel–Crafts Alkylation Reaction of Indoles,” Tetrahedron 65 (2009): 6877.

[48] A. D. Yamaguchi , D. Mandal , J. Yamaguchi , and K. Itami , “Oxidative C–H/C–H Coupling of Azine and Indole/Pyrrole Nuclei: Palladium Catalysis and Synthesis of Eudistomin U,” Chemistry Letters 40 (2011): 555.

[49] R. R. Reddy , K. Adlak , and P. Ghorai , “Catalyst‐free Synthesis of 6‐Hydroxy Indoles via the Condensation of Carboxymethyl Cyclohexadienones and Amines,” The Journal of Organic Chemistry 82 (2017): 8426. 28714691 10.1021/acs.joc.7b01136

[50] C. Wei , J. Wu , L. Zhang , and Z. Xia , “Gold(I)‐Catalyzed Selective Hydroarylation of Indoles with Haloalkynes,” Organic Letters 24 (2022): 4689. 35714368 10.1021/acs.orglett.2c01921

[51] J. G. Allen , K. Briner , M. P. Cohen , et al., “2,3,4,5‐Tetrahydro‐1H‐Benzo[d]azepines Substitués En Position 6 En Tant Qu'agonistes De Recepteur 5‐Ht2c,.” 2005: WO2005082859A1.

[52] C. Ostacolo , V. Di Sarno , G. Lauro , et al., “Identification of an Indol‐Based Multi‐Target Kinase Inhibitor Through Phenotype Screening and Target Fishing using Inverse Virtual Screening Approach,” European Journal of Medicinal Chemistry 167 (2019): 61. 30763817 10.1016/j.ejmech.2019.01.066

[53] S. Choi , J. Park , E. Yu , J. Sim , and C.‐M. Park , “Electrosynthesis of Dihydropyrano[4,3‐b]indoles Based on a Double Oxidative [3+3] Cycloaddition,” Angewandte Chemie International Edition 59 (2020): 11886. 32329937 10.1002/anie.202003364

[54] W. Zhuang , J. Zhang , C. Ma , et al., “Scalable Electrochemical Aerobic Oxygenation of Indoles to Isatins without Electron Transfer Mediators by Merging with an Oxygen Reduction Reaction,” Organic Letters 24 (2022): 4229. 35678516 10.1021/acs.orglett.2c01545

[55] S. Maiti , J. S. Kim , and J. Kim , “Cu‐Catalyzed Aerobic Oxidative Dehydrogenation of Tertiary Indolines to Indoles Using Azo/Hydrazide Redox,” New Journal of Chemistry 48 (2024): 3342.

[56] M. Kitano , A. Kojima , K. Nakano , A. Miyagishi , T. Noguchi , and N. Ohashi , “Synthesis and Biological Activity of N‐(Aminoiminomethyl)‐1H‐Indole Carboxamide Derivatives as Na+/H+ Exchanger Inhibitors,” Chemical and Pharmaceutical Bulletin 47 (1999): 1538. 10605052 10.1248/cpb.47.1538

[57] L. Xiong , X.‐L. Zhu , H.‐W. Gao , et al., “Discovery of Potent Succinate‐Ubiquinone Oxidoreductase Inhibitors via Pharmacophore‐linked Fragment Virtual Screening Approach,” Journal of Agricultural and Food Chemistry 64 (2016): 4830. 27225833 10.1021/acs.jafc.6b00325

[58] S. Zhou , J. Wang , L. Wang , K. Chen , C. Song , and J. Zhu , “Co(III)‐Catalyzed, Internal and Terminal Alkyne‐Compatible Synthesis of Indoles,” Organic Letters 18 (2016): 3806. 27434348 10.1021/acs.orglett.6b01805

[59] Y.‐Z. Ji , H.‐J. Li , Y.‐R. Wang , Z.‐Y. Zhang , and Y.‐C. Wu , “Sulfoxide‐Promoted Chlorination of Indoles and Electron‐Rich Arenes with Chlorine as Nucleophile,” Advanced Synthesis & Catalysis 362 (2020): 1039.

[60] N. Jacob , Y. Zaid , J. C. A. Oliveira , L. Ackermann , and J. Wencel‐Delord , “Cobalt‐Catalyzed Enantioselective C–H Arylation of Indoles,” Journal of the American Chemical Society 144 (2022): 798. 35001624 10.1021/jacs.1c09889

[61] S. Liu , Y. Huang , J. Wang , F.‐L. Qing , and X.‐H. Xu , “General Synthesis of N‐Trifluoromethyl Compounds with N‐Trifluoromethyl Hydroxylamine Reagents,” Journal of the American Chemical Society 144 (2022): 1962. 35045700 10.1021/jacs.1c12467

[62] S. M. Bronner , K. B. Bahnck , and N. K. Garg , “Indolynes as Electrophilic Indole Surrogates: Fundamental Reactivity and Synthetic Applications” Organic Letters 11 (2009): 1007. 19178159 10.1021/ol802958a

[63] Y. Du , Y. Wang , X. Li , et al., “N‐Heterocyclic Carbene Organocatalytic Reductive β,β‐Coupling Reactions of Nitroalkenes via Radical Intermediates,” Organic Letters 16 (2014): 5678. 25343564 10.1021/ol5027415

[64] Y. Ogiwara , Y. Sakurai , H. Hattori , and N. Sakai , “Palladium‐Catalyzed Reductive Conversion of Acyl Fluorides via Ligand‐Controlled Decarbonylation,” Organic Letters 20 (2018): 4204. 29963866 10.1021/acs.orglett.8b01582

[65] S. Zhao , Y. Guo , Z. Su , C. Wu , W. Chen , and Q.‐Y. Chen , “Deoxyfluorination of Carboxylic, Sulfonic, Phosphinic Acids and Phosphine Oxides by Perfluoroalkyl Ether Carboxylic Acids Featuring CF_2_O Units,” Chinese Journal of Chemistry 39 (2021): 1225.

[66] M. G. Darnowski , T. D. Lanosky , A. R. Paquette , and C. N. Boddy , “Synthesis of a Constitutional Isomer of Armeniaspirol A, Pseudoarmenia spirol A, via Lewis Acid‐Mediated Rearrangement,” The Journal of Organic Chemistry 87 (2022): 15634. 36322913 10.1021/acs.joc.2c02331

[67] J. K. Laha , M. K. Hunjan , T. Maity , and A. Hazra , “Site‐Selective Decarboxylative Direct Formylation of Nitrogen Heterocycles (Azaindoles, Indoles, Pyrroles) and Anilines Utilizing Glyoxylic Acid,” Advanced Synthesis & Catalysis 365 (2023): 1238.

[68] F. J. Lundevall and H.‐R. Bjørsvik , “Lithiation and Alkylation of the Imidazole Backbone,” European Journal of Organic Chemistry 26 (2023): e202201504.

[69] B. G. Nangunuri , R. P. Shirke , and M.‐h. Kim , “Metal‐Free Synthesis of Dihydrofuran Derivatives as Anti‐Vicinal Amino Alcohol Isosteres,” Organic & Biomolecular Chemistry 21 (2023): 960. 36625241 10.1039/d2ob02077g

[70] N. Ree , A. H. Göller , and J. H. Jensen , “Automated Quantum Chemistry for Estimating Nucleophilicity and Electrophilicity with Applications to Retrosynthesis and Covalent Inhibitors,” Digital Discovery 3 (2024): 347.

