# Supplementary material for: Direct Fluoroformylation of the C3‐Position of Indoles with 2,4‐Dinitro(trifluoromethoxy)benzene as Fluorocarbonyl Source
Source: ChemistryOpen. 2026 Feb 28;15(3):e202500563. doi: 10.1002/open.202500563 (PMC12949452; doi:10.1002/open.202500563)
Supplement: Supplementary file 1 — Supplementary Material [file OPEN-15-e202500563-s001.zip › open70109-sup-0001-SuppData-S1/open70109-sup-0001-SuppData-S1.pdf]

ChemistryOpen

Supporting Information

Direct Fluoroformylation of the *C3*-position of Indoles with 2,4-Dinitro(trifluoromethoxy)benzene as fluorocarbonyl source.

L. Wisson, G. Hanquet, F. Toulgoat, T. Billard, F. R. Leroux* and A. Panossian*

I. Table of contents

I. General Information 3

II. Substrate Synthesis 4

III. Optimization of the reaction conditions 14

IV. Control experiments 16

A. Fluoroformylation of 1-methylindole 1a without DMAP 16

B. Fluoroformylation of 1-methylindole 1a with a two-tube set-up 16

C. Reaction of 1-methylindole 1a with DNTFB without DMAP 17

D. Reaction of 1-methylindole 1a with DNTFB and a catalytic amount of DMAP 17

V. DFT calculation of the nucleophilicity of indoles and other heteroaromatic rings with ESNuEL 19

VI. Fluoroformylation of indoles and other heteroaromatic cycles 20

VII. Post-functionalisation reactions 34

A. Chiral-HPLC analyses of 2q1 and *rac*-2q1 37

VIII. Bibliography 39

IX. Crystal description of 2z5 40

X. NMR spectra 45

I. General Information

Starting materials, when commercially available, were purchased from standard suppliers such as Sigma-Aldrich, Fluorochem, Fischer scientific, Apollo scientific, TCI, ABCR, Alfa Aesar or BLD pharm. They were used without further purification unless otherwise noted. Technical grade solvents used for extraction, precipitation or chromatography (pentane, cyclohexane, dichloromethane (DCM), diethyl ether, ethyl acetate or petroleum ether (PE)) were purchased and used without further purification. Anhydrous solvents purchased from standard chemical suppliers were used as received. Air- and moisture-sensitive materials were stored and handled under an argon atmosphere. When needed, reactions were carried out under argon atmosphere and heated with an oil bath or a heating block.

Reactions were monitored using thin-layer chromatography (TLC) with precoated silica on aluminium foils (0.25 mm thickness, Merck silica-gel (60-F254)). TLCs were visualized using fluorescence quenching under a 254 nm UV lamp or by staining with potassium permanganate. Flash column chromatography purifications were performed using VWR silica gel (40-63 µm) using the indicated solvents system specified in V:V.

Spectroscopic NMR and MS data were obtained using chromatographically homogeneous samples. ^{1}H NMR (400 or 500 MHz), ^{19}F NMR (376 or 472 MHz) and ^{13}C NMR (101 or 126 MHz) spectra were recorded on Bruker Avance III HD 400 and 500 MHz instruments respectively in $CDCl_3$ or CD_3CN. All chemical shifts (δ) are reported in parts per million (ppm) and are referenced to residual solvent peaks in ^{1}H and ^{13}C NMR experiments such as partially deuterated chloroform (δ[^{1}H] = 7.26 ppm for $CHCl_3$ and δ[^{13}C] = 77.2 ppm for $CDCl_3$) or partially deuterated acetonitrile (δ[^{1}H] = 1.96 ppm for CH_3CN and δ[^{13}C] = 1.32 and 118.26 ppm for CD_3CN). ^{19}F spectra were calibrated using the ^{1}H spectrum as absolute reference. For the determination of ^{1}H and ^{19}F NMR yields, commercially available bromochloromethane and α,α,α-trifluorotoluene were used as internal standard. Spectra were processed with the program MestReNova (Mestreab Research, version 14.2). Multiplicities are abbreviated as br s (broad signal), s (singlet), d (doublet), t (triplet), q (quartet), pentet (p), septet (se), octet (o), m (multiplet), td (triplet of doublets), dd (doublet of doublets) and ddd (doublet of doublets of doublets). Coupling constants *J* are given in Hz. The notation ^{TS}J is used for through-space coupling constants and ^{x}J is used for unclear coupling constants. Infra-red spectra were recorded using a Perkin Elmer's Spectrum one™ spectrometer (ATR) coupled with a Diamond Attenuated Total Reflectance accessory. The data were analyzed with Spectrum 10, with a range of 4000 cm^{-1} to 350 cm^{-1} and a resolution of 4 cm^{-1}. Wavelengths (ν) are reported in cm^{-1} and signal intensities are abbreviated as s (strong), m (medium) and w (weak). Low-resolution mass spectra were obtained using an Agilent 7820A or 8060 spectrometer (mass sensitive detector: 5977E or 5977B) using electronic impact ionization (EI at 70 eV). High-resolution mass spectrometry (HRMS) analyses were performed with a Bruker MicroTOF mass analyser under ESI in positive ionization mode detection (measurement accuracy ≤ 15 ppm) by the analytical facility at the University of Strasbourg. X-ray crystallographic structure analyses were performed by the radio-crystallographic facility at the University de Strasbourg; analyses were carried out on a Bruker PHOTON III DUO CPAD diffractometer equipped with an Oxford Cryosystem liquid N_2 device, using Mo-Kα radiation (λ = 0.71073 Å). Optical rotations were recorded on an Anton Paar MCP-200 automatic polarimeter at 589 nm and reported as follows: $[\alpha]_{589}^{20}$. Chiral HPLC measurements were performed on a Shimadzu system with a quaternary low-pressure LC-20AD pump, an automatic SIL-20A HT injector, a CTO-10 AS oven and a SPD-M20 A diode array detector (DAD). The injection volume was 1 µL, the temperature of the oven set to 35 °C and the concentration of the sample around 1 g/L. The chiral column Chiralcel® OD-H by Daicel Chiral Technologies was used. Melting points were recorded on a Büchi Melting Point M-560 apparatus. Measures were repeated three times. Values reported are mean values of the triplicates.

II. Substrate Synthesis

Representative procedure 1 (RP1) for the synthesis of *N*-methylated-1*H*-indoles[1]

NaH (60w% in mineral oil, 160 mg, 4.00 mmol 2.0 equiv.) was added to a solution of the desired *1H*-indole derivative (2.00 mmol, 1.0 equiv.) in anhydrous dimethoxyethane DME (5 mL) and dimethyl sulfoxide (DMSO) (1 mL). The resulting solution was stirred at 25 °C for 40 min, then MeI (0.19 mL, 3.00 mmol, 1.5 equiv.) was added dropwise. The resulting mixture was stirred for 70 min at 25 °C. The reaction was then quenched with H_2O (5 mL) and brine (5 mL). The biphasic mixture was further diluted with Et_2O (20 mL). The layers were separated, and the aqueous layer was extracted with Et_2O (2 × 20 mL). The combined organic layers were washed with H_2O (2 x 20 mL), brine (1 x 20 mL), then dried over $MgSO_4$. The resulting solution was concentrated under reduced pressure. The resulting material was purified by filtration through a silica pad (with DCM as eluent) or by flash column chromatography with the appropriate eluent system (given in V:V).

1-Methyl-6-methoxy-1*H*-indole 1d (CAS Number: 1968-17-8)

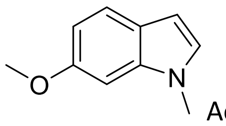

According to RP1, 1-methyl-6-methoxy-1*H*-indole **1d** was obtained from 6-methoxyindole (221 mg, 1.50 mmol, 1.0 equiv.), NaH (60w% in mineral oil, 157 mg, 3.94 mmol 2.6 equiv.) and MeI (0.14 mL, 2.25 mmol, 1.5 equiv.). The crude material was purified by flash column chromatography (Pentane/Et_2O 90:10, R_f = 0.43) to give **1d** as a colourless solid (242 mg, 1.50 mmol, quant.). **^{1}H NMR** (400 MHz, $CDCl_3$) δ 7.50 (d, $^3J_{H-H}$ = 9.3 Hz, 1H), 6.95 (d, $^4J_{H-H}$ = 3.2 Hz, 1H), 6.82 – 6.76 (m, 2H), 6.42 (dd, $^3J_{H-H}$ = 3.1 Hz, $^4J_{H-H}$ = 0.8 Hz, 1H), 3.89 (s, 3H), 3.75 (s, 3H). **^{13}C{^{1}H} NMR** (126 MHz, $CDCl_3$) δ 156.4, 137.5, 127.9, 122.9, 121.6, 109.4, 100.9, 92.9, 55.9, 33.0. **LRMS** *(m/z)* = $[M]^{\bullet+}$ 161.08 (90.3%), $[M - Me]^+$ 146.06 (100.0%), $[M - OMe]^+$ 130.05 (3.6%).

In accordance with the literature.[2]

6-Methoxy-1-(prop-2-en-1-yl)-1*H*-indole 1e (CAS Number: 1309774-31-9)

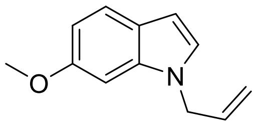

The product was synthetized according to the work of Kokotos *et al.*[3] A 50 mL round-bottom flask equipped with a stir bar was charged with 6-methoxy-1*H*-indole (291 mg, 1.98 mmol, 1.0 equiv.) and crushed KOH (339 mg, 6.04 mmol, 3.0 equiv.). Then, DMSO (5 mL) was added to the flask and the solution was stirred at 25 °C for 15 min. Next, allyl bromide (0.35 mL, 4.02 mmol, 2.0 equiv.) was added. The reaction mixture was further stirred at 25 °C for 18 h, then diluted with DCM (15 mL). The organic layer was washed with water (15 mL), and dried over $MgSO_4$, filtered and concentrated under reduced pressure. The crude product was purified by flash chromatography (c-hexane/EtOAc 90:10, R_f = 0.47) to give **1e** as a yellow oil (278 mg, 1.49 mmol, 75%). **^{1}H NMR** (400 MHz, $CDCl_3$) δ 7.50 (d, $^3J_{H-H}$ = 9.3 Hz, 1H), 6.99 (d, $^4J_{H-H}$ = 3.2 Hz, 1H), 6.81 – 6.76 (m, 2H), 6.45 (dd, $^3J_{H-H}$ = 3.2 Hz, $^4J_{H-H}$ = 0.8 Hz, 1H), 5.99 (ddt, $^3J_{H-H\ trans}$ = 17.1 Hz, $^3J_{H-H\ cis}$ = 10.4 Hz, $^3J_{H-H}$ = 5.3 Hz, 1H), 5.21 (app. dq, $^3J_{H-H\ cis}$ = 10.3 Hz, $^2J_{H-H}$ = 1.5 Hz, 1H), 5.09 (dtd, $^3J_{H-H\ trans}$ = 17.1 Hz, $^4J_{H-H}$ = 1.8 Hz, $^2J_{H-H}$ = 1.2 Hz, 1H), 4.68 (dt, $^3J_{H-H}$ = 5.3 Hz, $^4J_{H-H}$ = 1.7 Hz, 2H), 3.86 (s, 3H). **^{13}C{^{1}H} NMR** (126 MHz, $CDCl_3$)

δ 156.4, 137.0, 133.5, 126.9, 123.1, 121.6, 117.3, 109.4, 101.4, 93.5, 55.9, 49.0. **IR** (ATR) 3084 (w), 3004 (w), 2930 (br w), 2835 (w), 1618 (m), 1575 (w), 1510 (m), 1492 (m), 1461 (m), 1440 (m), 1329 (m), 1311 (m), 1261 (s), 1212 (s), 1175 (m), 1117 (w), 1083 (m), 1037 (m), 988 (m), 929 (m), 807 (m), 754 (s), 714 (s). **HRMS-ESI** *(m/z)* $[M]^+$ calculated for $C_{12}H_{14}ON$, 188.1070; found, 188.1066.

1-Benzyl-6-methoxy-1*H*-indole 1f (CAS Number: 90811-54-4)

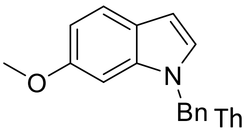

The product was synthetized according to the work of Kokotos *et al.*[3] To a solution of 6-methoxy-1*H*-indole (294 mg, 2.00 mmol, 1.0 equiv.) in dry tetrahydrofuran (THF) (4 mL) at 0 °C, NaH (60w% in mineral oil, 120 mg, 3.01 mmol, 1.5 equiv.) was added under an argon atmosphere. The heterogenous mixture was stirred at 0 °C for 15 min and then at 25 °C for 1 h. It was then cooled down to 0 °C and benzyl bromide (0.31 mL, 2.60 mmol, 1.3 equiv.) was added and the mixture was allowed to warm to 25 °C. After completion of the reaction, the reaction mixture was cooled again to 0 °C, quenched with sat. aq. NH_4Cl (4 mL) and extracted with Et_2O (3 x 40 mL). The combined organic layers were washed with brine (40 mL), dried over $MgSO_4$ and concentrated under reduced pressure. The resulting material was purified by flash column chromatography (Pentane/Et_2O 95:5, R_f = 0.45) to give **1f** as a yellowish cloudy oil (207 mg, 0.87 mmol, 44%). **^{1}H NMR** (400 MHz, $CDCl_3$) δ 7.52 (d, $^3J_{H-H}$ = 8.6 Hz, 1H), 7.34 – 7.26 (m, 3H), 7.14 – 7.09 (m, 2H), 7.02 (d, $^4J_{H-H}$ = 3.2 Hz, 1H), 6.79 (dd, $^3J_{H-H}$ = 8.6 Hz, $^4J_{H-H}$ = 2.2 Hz, 1H), 6.74 (d, $^4J_{H-H}$ = 2.3 Hz, 1H), 6.48 (dd, $^3J_{H-H}$ = 3.2 Hz, $^4J_{H-H}$ = 0.9 Hz, 1H), 5.27 (s, 2H), 3.80 (s, 3H). **^{13}C{^{1}H} NMR** (126 MHz, $CDCl_3$) δ 156.3, 137.5, 137.1, 128.8 (2C), 127.6, 127.3, 126.8 (2C), 123.1, 121.5, 109.4, 101.6, 93.5, 55.7, 50.1. **LRMS** *(m/z)* = $[M]^{\bullet+}$ 237.09 (100.0%), $[M - Me]^+$ 222.07 (21.2%), $[M - Bn]^+$ 146.02 (61.5%), $[Bn]^{\bullet+}$ 91.04 (91.4%).

In accordance with the literature.[4]

6-Methoxy-1-[(4-methoxyphenyl)methyl]-1*H*-indole 1g (CAS Number: 1142120-31-7)

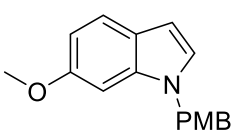

The product was synthetized according to the work of Boger *et al.*[5] A solution of 6-methoxy-1*H*-indole (441 mg, 3.00 mmol, 1.0 equiv.) in DMF (4.5 mL) was treated with NaH (60w% in mineral oil., 152 mg, 3.81 mmol, 1.3 equiv.) at 25 °C. 4-Methoxybenzyl bromide (0.52 mL, 3.61 mmol, 1.2 equiv.) was added dropwise at 25 °C and the mixture was stirred for 16 h. It was quenched by the addition of saturated aqueous NH_4Cl and partitioned between Et_2O and water. The aqueous layer was extracted with Et_2O and the combined organic layers were washed with a saturated aqueous $NaHCO_3$ solution and brine. The organic layer was dried over anhydrous $MgSO_4$, filtered, and concentrated under reduced pressure. The resulting material was purified by flash column chromatography (Pentane/Et_2O 80:20, R_f = 0.61) to give **1g** as a cloudy yellow oil (528 mg, 1.97 mmol, 66%). **^{1}H NMR** (400 MHz, $CDCl_3$) δ 7.51 (d, $^3J_{H-H}$ = 8.5 Hz, 1H), 7.10 – 7.05 (m, 2H), 7.00 (d, $^4J_{H-H}$ = 3.1 Hz, 1H), 6.87 – 6.82 (m, 2H), 6.79 (dd, $^3J_{H-H}$ = 8.5 Hz, $^4J_{H-H}$ = 2.2 Hz, 1H), 6.76 (d, $^3J_{H-H}$ = 2.3 Hz, 1H), 6.46 (dd, $^3J_{H-H}$ = 3.2 Hz, $^4J_{H-H}$ = 0.9 Hz, 1H) 5.20 (s, 2H), 3.82 (s, 3H), 3.78 (s, 3H). **^{13}C{^{1}H} NMR** (126 MHz, $CDCl_3$) δ 159.2, 156.4, 137.1, 129.6, 128.3 (2C), 127.2, 123.2, 121.6, 114.3 (2C), 109.4, 101.6, 93.6, 55.8, 55.4, 49.7. **LRMS** *(m/z)* = $[M]^{\bullet+}$ 267.11 (46.8%), $[M - PMB]^+$ 146.05 (7.1%), $[PMB]^{\bullet+}$ 121.10 (100.0%).

In accordance with the literature.[5]

6-Methoxy-1-{[2-(trimethylsilyl)ethoxy]methyl}-1*H*-indole 1h (CAS Number: 1247072-09-8)

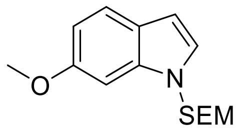

The product was synthetized according to the work of DeBoef *et al*.[6] To a solution of KOH (186 mg, 3.31 mmol, 1.1 equiv.) in DMF (15 mL) at 0 °C was added a solution of 6-methoxy-1*H*-indole (441 mg, 3.00 mmol, 1.0 equiv.) in DMF (2.5 mL) and the reaction mixture was stirred for 15 min. Then, a solution of SEM-Cl (0.53 mL, 2.99 mmol, 1.0 equiv.) in DMF (2.8 mL) was added and the mixture was stirred for 6 h at 0 °C. After this time, the mixture was diluted with 40 mL of H_2O and extracted with EtOAc (3 x 40 mL). The combined organic layers were washed with H_2O (2 x 40 mL) and dried over $MgSO_4$. After filtration, the solvent was removed under reduced pressure and the crude product was purified by flash column chromatography (Pentane/Et_2O 90:10, R_f = 0.46) to give **1h** as a yellow oil (381 mg, 1.37 mmol, 46 %). **^{1}H NMR** (400 MHz, $CDCl_3$) δ 7.49 (d, $^3J_{H-H}$ = 8.6 Hz, 1H), 7.05 (d, $^3J_{H-H}$ = 3.2 Hz, 1H), 6.97 (dt, $^4J_{H-H}$ = 2.3, 0.7 Hz, 1H), 6.82 (dd, $^3J_{H-H}$ = 8.6 Hz, $^4J_{H-H}$ = 2.3 Hz, 1H), 6.45 (dd, $^3J_{H-H}$ = 3.2 Hz, $^4J_{H-H}$ = 0.8 Hz, 1H), 5.43 (s, 2H), 3.88 (s, 3H), 3.52 – 3.44 (m, 2H), 0.95 – 0.83 (m, 2H), -0.05 (s, 9H). **^{13}C{^{1}H} NMR** (126 MHz, $CDCl_3$) δ 156.7, 137.3, 127.2, 123.4, 121.6, 110.3, 102.4, 93.8, 75.9, 65.8, 55.9, 17.9, -1.3. **LRMS** *(m/z)* = $[M]^{\bullet+}$ 277.16 (58.8%), [M - 2 x $CH_3]^{\bullet+}$ 247.14 (5.6%), [M - TMS]$^{\bullet}$ 204.1 (66.9%), [M - $OCH_2CH_2TMS]^+$ 160.09 (72.1%), [M - SEM]$^+$ 146.08 (25.3%), [$OCH_2CH_2TMS]^{\bullet+}$ 117.06 (34.1%), [SEM]$^{\bullet+}$ 73.09 (100.0%).

In accordance with the literature.[6]

6-Methoxy-1-(4-toluenesulfonyl)-1*H*-indole 1i (CAS Number: 107734-26-9)

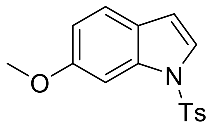

The product was synthetized according to the work of Itami *et al*.[7] To a stirred solution of 6-methoxy-1*H*-indole (222 mg, 1.51 mmol, 1.0 equiv.) in anhydrous THF (15 mL) was added NaH (60w% in mineral oil, 95.4 mg, 2.39 mmol, 1.6 equiv.) at 0 °C. The reaction mixture was allowed to warm to 25 °C and was stirred for 30 min. After cooling down again to 0 °C, tosyl chloride (353 mg, 1.85 mmol, 1.2 equiv.) was added to the reaction mixture, which was stirred at 25 °C for 16 h. It was then quenched with a saturated aqueous $NaHCO_3$ solution (10 mL). The aqueous layer was extracted with EtOAc (2 x 10 mL), and the combined organic layers were washed with brine, dried over $MgSO_4$ and concentrated under reduced pressure. The crude mixture was purified by flash column chromatography (*c*-hexane/EtOAc 90:10, R_f = 0.30) to give **1i** as a colourless solid (216 mg, 0.72 mmol, 47%). **^{1}H NMR** (400 MHz, $CDCl_3$) δ 7.74 (d, $^3J_{H-H}$ = 8.4 Hz, 2H), 7.53 (d, $^4J_{H-H}$ = 2.3 Hz, 1H), 7.44 (d, $^4J_{H-H}$ = 3.7 Hz, 1H), 7.38 (d, $^3J_{H-H}$ = 8.7 Hz, 1H), 7.22 (d, $^3J_{H-H}$ = 8.7 Hz, 2H), 6.85 (dd, $^3J_{H-H}$ = 8.6 Hz, $^4J_{H-H}$ = 2.3 Hz, 1H), 6.57 (dd, $^3J_{H-H}$ = 3.7 Hz, $^4J_{H-H}$ = 0.8 Hz, 1H), 3.87 (s, 3H), 2.34 (s, 3H). **^{13}C{^{1}H} NMR** (126 MHz, $CDCl_3$) δ 158.1, 145.0, 136.0, 135.5, 130.0 (2C), 126.9 (2C), 125.3, 124.6, 121.9, 112.7, 109.1, 98.0, 55.9, 21.7. **LRMS** *(m/z)* = $[M]^{\bullet+}$ 301.05 (16.9%), [M - Ts]$^+$ 146.05 (100.0%), [$PhCH_3]^+$ 91.04 (25.8%).

In accordance with the literature.[8]

1-Acetyl-6-methoxy-1*H*-indole 1j (CAS Number: 126759-60-2)

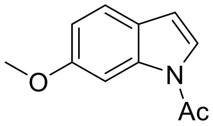

The product was synthetized according to the work of Xia *et al.*[9] To a stirring solution of 6-methoxy-1*H*-indole (298 mg, 2.03 mmol, 1.0 equiv.) in anhydrous DMF (8 mL), NaH (60w% in mineral oil, 123 mg, 3.09 mmol, 1.5 equiv.) was added portion wise at 0 °C and the stirring was continued for 30 min. Then, acetyl chloride (0.30 mL, 4.20 mmol, 2.1 equiv.) was added dropwise and the mixture was allowed to warm to 25 °C and stirred for 2 h. After that time, the reaction was quenched with a saturated aqueous $NaHCO_3$ solution (25 mL) and extracted with EtOAc (3 × 25 mL). The combined organic layers were dried over $MgSO_4$ and concentrated under reduced pressure. The resulting material was purified by flash column chromatography (*c*-hexane/EtOAc 90:10, R_f = 0.20) to give **1j** as a colourless solid (145 mg, 0.77 mmol, 38%). **^{1}H NMR** (400 MHz, $CDCl_3$) δ 8.06 (d, $^3J_{H-H}$ = 2.3 Hz, 1H), 7.43 (d, $^3J_{H-H}$ = 8.7 Hz, 1H), 7.30 (d, $^4J_{H-H}$ = 3.8 Hz, 1H), 6.91 (dd, $^3J_{H-H}$ = 8.6 Hz, $^4J_{H-H}$ = 2.4 Hz, 1H), 6.57 (dd, $^3J_{H-H}$ = 3.8 Hz, $^4J_{H-H}$ = 0.7 Hz, 1H), 3.89 (s, 3H), 2.63 (s, 3H). **^{13}C{^{1}H} NMR** (126 MHz, $CDCl_3$) δ 168.9, 158.4, 136.6, 124.0, 123.9, 121.1, 113.0, 109.1, 100.7, 55.7, 24.0. **IR** (ATR) 3385 (w), 3146 (w), 3121 (w), 3004 (w), 2927 (br w), 2853 (w), 2832 (w), 1692 (m), 1615 (m), 1535 (m), 1477 (m), 1464 (m), 1437 (w), 1384 (m), 1363 (m), 1329 (s), 1317 (m), 1280 (m), 1252 (m), 1215 (s), 1135 (m), 1114 (m), 1086 (m), 1037 (m), 948 (m), 923 (m), 874 (m), 813 (s), 764 (m), 717 (m). **HRMS-ESI** *(m/z)* $[M]^+$ calculated for $C_{11}H_{11}O_2NNa$, 212.0682; found, 212.0679.

In accordance with the literature.[10]

***tert*-Butyl 6-methoxy-1*H*-indole-1-carboxylate 1k (CAS Number: 138344-18-0)**

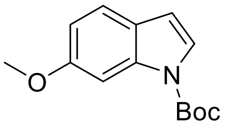

The product was synthetized according to the work of Xia *et al.*[11] To a stirred solution of 6-methoxy-1*H*-indole (297 mg, 2.02 mmol, 1.0 equiv.) in anhydrous THF (21 mL) were added Boc_2O (563 mg, 2.58 mmol, 1.3 equiv.) and then DMAP (72.6 mg, 0.59 mmol, 0.3 equiv.). The resulting mixture was stirred at 25 °C for 16 h, then concentrated and the resulting material was purified by filtration over silica gel (Pentane/EtOAc 90:10 R_f = 0.90) to give **1k** as a colourless oil (497 mg, 1.97 mmol, 97%). **^{1}H NMR** (400 MHz, $CDCl_3$) δ 7.75 (s, 1H), 7.47 (d, $^4J_{H-H}$ = 3.7 Hz, 1H), 7.42 (d, $^3J_{H-H}$ = 8.6 Hz, 1H), 6.87 (dd, $^3J_{H-H}$ = 8.6 Hz, $^4J_{H-H}$ = 2.4 Hz, 1H), 6.49 (dd, $^3J_{H-H}$ = 3.7 Hz, $^4J_{H-H}$ = 0.8 Hz, 1H), 3.88 (s, 3H), 1.67 (s, 9H). **^{13}C{^{1}H} NMR** (126 MHz, $CDCl_3$) δ 157.8, 150.0, 124.7, 124.4, 121.4, 112.3, 107.3, 99.4, 83.6, 55.7, 28.4. **LRMS** *(m/z)* = $[M]^{\bullet+}$ 247.07 (15.1%), [M - *t*Bu + H]$^{\bullet+}$ 191.01 (84.8%), [M - Boc + H]$^+$ 147.03 (77.0 %), [M - Boc - Me + H]$^+$ 132.01 (100.0%).

In accordance with the literature.[11]

4-Methoxy-1-methyl-1*H*-indole 1l (CAS Number: 7556-35-6)

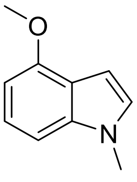

According to RP1, 4-methoxy-1-methyl-1*H*-indole **1l** was obtained from 4-methoxy-1*H*-indole (222 mg, 1.51 mmol, 1.0 equiv.), NaH (60w% in mineral oil, 151 mg, 2.50 mmol, 2.5 equiv.) and MeI (0.12 mL, 1.95 mmol, 1.3 equiv.). The crude material was purified by flash column chromatography (Pentane/Et_2O 90:10, R_f = 0.46) to give **1l** as a colourless solid (212 mg, 1.32 mmol, 87%). **^{1}H NMR** (400 MHz, $CDCl_3$) δ 7.15 (t, $^3J_{H\text{-}H}$ = 8.0 Hz, 1H), 6.99 – 6.94 (m, 2H), 6.59 (dd, $^3J_{H\text{-}H}$ = 3.1 Hz, $^4J_{H\text{-}H}$ = 0.9 Hz, 1H), 6.53 (d, $^3J_{H\text{-}H}$ = 7.7 Hz, 1H), 3.97 (s, 3H), 3.78 (s, 3H). **^{13}C{^{1}H} NMR** (126 MHz, $CDCl_3$) δ 153.6, 138.3, 127.4, 122.5, 119.1, 102.8, 99.4, 98.3, 55.5, 33.2. **LRMS** *(m/z)* = $[M]^{\bullet+}$ 161.05 (100.0%), $[M - Me]^+$ 146.03 (100.0%), $[M - OMe]^+$ 130.04 (5.3%).

In accordance with the literature.[12]

7-Methoxy-1-methyl-1*H*-indole 1n (CAS Number: 51460-41-4)

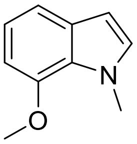

According to RP1, 7-methoxy-1-methyl-1*H*-indole **1m** was obtained from 7-methoxy-1*H*-indole (230 mg, 1.99 mmol, 1.0 equiv.), NaH (60w% in mineral oil, 160 mg, 4.01 mmol, 2.0 equiv.) and MeI (0.19 mL, 2.98 mmol, 1.5 equiv.). The crude material was purified by filtration through a silica plug (with DCM as eluent) to give **1n** as a light-yellow solid (321.0 mg, 1.99 mmol, quant.). **^{1}H NMR** (400 MHz, $CDCl_3$) δ 7.20 (dd, $^3J_{H\text{-}H}$ = 8.0 Hz, $^3J_{H\text{-}H}$ = 0.9 Hz, 1H), 6.97 (t, $^3J_{H\text{-}H}$ = 7.8 Hz, 1H), 6.92 (d, $^3J_{H\text{-}H}$ = 3.0 Hz, 1H), 6.61 (d, $^3J_{H\text{-}H}$ = 7.7 Hz, 1H), 6.41 (d, $^3J_{H\text{-}H}$ = 3.0 Hz, 1H), 4.06 (s, 3H), 3.93 (s, 3H). **^{13}C{^{1}H} NMR** (126 MHz, $CDCl_3$) δ 148.0, 131.0, 129.9, 126.6, 119.9, 113.9, 102.4, 101.1, 55.5, 36.6. **LRMS** *(m/z)* = $[M]^{\bullet+}$ 161.14 (99.1%), $[M - Me]^+$ 146.12 (100.0%), $[M - OMe]^+$ 130.09 (8.0%).

In accordance with the literature.[2]

***tert*-Butyl (1-methyl-1*H*-indol-5-yl)carbamate 1o (CAS Number: 1616246-58-2)**

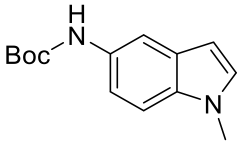

The product was synthetized following the work of C-M. Park *et al.*[13] 1-Methyl-1*H*-indol-5-amine (731 mg, 5.00 mmol, 1.0 equiv.) and Boc_2O (1.25 g, 5.73 mmol, 1.2 equiv.) were dissolved in EtOAc (50 mL) and the mixture was stirred at 25 °C. After the completion of the reaction, the solution was diluted with EtOAc (20 mL) and washed with water (3 x 30 mL). The combined organic layer was dried over $MgSO_4$, filtered and concentrated. The crude material was purified by flash chromatography (c-hexane/EtOAc 90:10, R_f = 0.48) to give *tert*-butyl (1-methyl-1*H*-indol-5-yl)-carbamate **1o** as an orange solid (1.17 g, 4.73 mmol, 95%). **^{1}H NMR** (400 MHz, $CDCl_3$) δ 7.66 (br s, 1H), 7.22 (app. dt, $^3J_{H\text{-}H}$ = 8.7 Hz, $^4J_{H\text{-}H}$ = 0.8 Hz, 1H), 7.14 (dd, $^3J_{H\text{-}H}$ = 8.8 Hz, $^4J_{H\text{-}H}$ = 2.0 Hz, 1H), 7.02 (d, $^3J_{H\text{-}H}$ = 3.1 Hz, 1H), 6.43 (br s, 1H), 6.41 (dd, $^3J_{H\text{-}H}$ = 3.1 Hz, $^4J_{H\text{-}H}$ = 0.8 Hz, 2H), 3.76 (s, 3H), 1.53 (s, 9H). **^{13}C{^{1}H} NMR** (126 MHz, $CDCl_3$) δ 153.5, 133.8, 130.5, 129.5, 128.6, 115.1, 111.3, 109.2, 100.8, 79.9, 32.9, 28.4

(3C). **LRMS** *(m/z)* = $[M]^{•+}$ 246.13 (8.1%), [M - *t*Bu + H]$^{•+}$ 190.07 (41.1%), [M - *t*BuO + H]$^{+}$ 172.03 (51.3%), [M - Boc + H]$^{•+}$ 146.05 (100.0%), [M - Boc - Me + H]$^{•+}$ 131.05 (31.8%).

In accordance with the literature.[13]

1-Methyl-1*H*-indole-4-carbonitrile 3 (CAS Number: 628711-58-0)

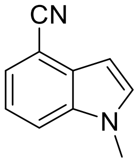

According to RP1, 1-methyl-1*H*-indole-4-carbonitrile **3** was obtained from 1*H*-indole-4-carbonitrile (501 g, 3.53 mmol, 1.0 equiv.), NaH (60w% in mineral oil, 355 mg, 8.9 mmol, 2.5 equiv.) and MeI (0.33 mL, 5.3 mmol, 1.5 equiv.). The crude material was purified by flash column chromatography (*c*-hexane/EtOAc 100:0 to 60:40, R_f = 0.29 in *c*-hexane/EtOAc 80:20) to give **3** as an off-white solid (352 mg, 2.30 mmol, 64%). **^{1}H NMR** (500 MHz, $CDCl_3$) δ 7.54 (app dt, $^3J_{H-H}$ = 8.2 Hz, $^xJ_{H-H}$ = 0.9 Hz, 1H), 7.47 (dd, $^3J_{H-H}$ = 7.4 Hz, $^4J_{H-H}$ = 0.9 Hz, 1H), 7.27 – 7.22 (m, 2H), 6.70 (dd, $^3J_{H-H}$ = 3.1 Hz, $^xJ_{H-H}$ = 0.9 Hz, 1H), 3.85 (s, 3H). **^{13}C{^{1}H} NMR** (126 MHz, $CDCl_3$) δ 136.4, 131.4, 129.7, 124.8, 121.1, 118.8, 114.0, 103.1, 100.1, 33.1. **LRMS** *(m/z)* = $[M]^{•+}$ 156.11 (100.0%), [M - Me]$^{+}$ 141.07 (8.0%), [M - Me - CN]$^{•+}$ 115.05 (6.9%).

In accordance with the literature.[14]

1-(1-Methyl-1*H*-indol-4-yl)-methanamine 4 (CAS Number: 864264-02-8)

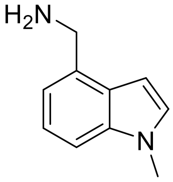

The product was synthetized following WO2005082859A1.[15] 1-Methyl-1*H*-indole-4-carbonitrile **3** (342 mg, 2.20 mmol, 1.0 equiv.) was dissolved in anhydrous THF (3.5 mL) and $LiAlH_4$ (1 M in THF, 4.4 mL, 4.40 mmol, 2.0 equiv.) was added slowly at room temperature. The reaction mixture was heated at 50 °C for 17 hours, then allowed to cool down to room temperature before being quenched with water (5 mL). The solid was filtered through Celite® and the filtrate was evaporated under reduced pressure. The residual material was washed with a saturated aqueous $NaHCO_3$ solution (3 x 10 mL), dried over $MgSO_4$ and concentrated under reduced pressure to afford **4** as a yellow solid (114 mg, 0.71 mmol, 32%). **^{1}H NMR** (500 MHz, $CDCl_3$) δ 7.27 – 7.20 (m, 2H), 7.09 – 7.05 (m, 2H), 6.56 (dd, $^3J_{H-H}$ = 3.1 Hz, $^xJ_{H-H}$ = 0.9 Hz, 1H), 4.15 (s, 2H), 3.81 (s, 3H), 1.61 (s, 2H). **^{13}C{^{1}H} NMR** (126 MHz, $CDCl_3$) δ 136.9, 135.8, 128.8, 126.8, 121.9, 117.3, 108.2, 98.8, 44.7, 33.1. **IR** (ATR) 3345 (w), (3090 (w), 2912 (w), 1609 (w), 1584 (w), 1498 (m), 1446 (m), 1418 (m), 1357 (w), 1329 (m), 1289 (m), 1243 (w), 1157 (m), 1089 (m), 890 (s), 745 (s). **HRMS-ESI** *(m/z)* $[M]^+$ calculated for $C_{10}H_{13}N_2$, 161.1073; found, 161.1080. **mp**: 37.8 °C

tert-Butyl _N_-[(1-methyl-1_H_-indol-4-yl)-methyl]carbamate 1p

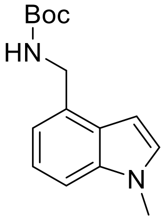

1-(1-Methyl-1*H*-indol-4-yl)methanamine **4** (494 mg, 3.10 mmol, 1.0 equiv.) and Boc_2O (0.73 mL, 3.4 mmol, 1.1 equiv.) were dissolved in EtOAc (30 mL) and the mixture was stirred at 25 °C until total conversion of the starting amine. The solution was then diluted with EtOAc (15 mL) and washed with water (3 x 20 mL). The combined organic layers were dried over $MgSO_4$, filtered and concentrated. The crude material was purified by flash chromatography (*c*-hexane/EtOAc 100:0 to 60:40, R_f = 0.41 in *c*-hexane/EtOAc 80:20) to afford **1p** as a yellow oil (643 mg, 2.50 mmol, 80%). **^{1}H NMR** (500 MHz, $CDCl_3$) δ 7.27 (app d, $^3J_{H\text{-}H}$ = 8.5 Hz, 1H), 7.19 (dd, $^3J_{H\text{-}H}$ = 8.3 Hz, $^3J_{H\text{-}H}$ = 7.1 Hz, 1H), 7.08 (d, $^3J_{H\text{-}H}$ = 3.1 Hz, 1H), 7.03 (dd, $^3J_{H\text{-}H}$ = 7.1, $^xJ_{H\text{-}H}$ = 0.9 Hz, 1H), 6.57 (d, $^3J_{H\text{-}H}$ = 3.1 Hz, 1H), 4.82 (s, 1H), 4.61 (d, $^3J_{H\text{-}H}$ = 5.6 Hz, 2H), 3.81 (s, 3H), 1.47 (s, 9H). **^{13}C{^{1}H} NMR** (126 MHz, $CDCl_3$) δ 156.0, 137.0, 130.7, 129.0, 127.2, 121.7, 118.6, 108.9, 99.3, 79.4, 43.2, 33.1, 28.6 (3 x C). **IR** (ATR) 3364 (m), 2977 (w), 2915 (w), 1679 (s), 1520 (s), 1495 (m), 1446 (w), 1424 (w), 1363 (m), 1280 (s), 1246 (m), 1169 (s), 1083 (m), 1046 (m), 929 (m), 868 (m), 742 (s), 711 (m), 622 (m). **HRMS-ESI** *(m/z)* $[M + Na]^+$ calculated for $C_{15}H_{20}N_2NaO_2$, 283.1417; found, 283.1423. **mp**: 82.9 °C

5-Formyl-1-methyl-1_H_-indole 1r (CAS Number: 90923-75-4)

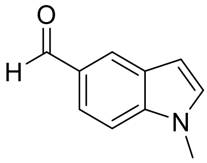

According to RP1, 5-formyl-1-methyl-1*H*-indole **1r** was obtained from 5-indolealdehyde (219 mg, 1.51 mmol, 1.0 equiv.), NaH (60w% in mineral oil, 158 mg, 3.96 mmol, 2.6 equiv.) and MeI (0.14 mL, 2.25 mmol, 1.5 equiv.). The crude material was purified by flash column chromatography (Pentane/Et_2O 80:20, R_f = 0.22) to give **1r** as a colourless solid (237 mg, 1.49 mmol, 99%). **^{1}H NMR** (400 MHz, $CDCl_3$) δ 10.04 (s, 1H), 8.15 (d, $^4J_{H\text{-}H}$ = 1.6 Hz, 1H), 7.80 (dd, $^3J_{H\text{-}H}$ = 8.6 Hz, $^4J_{H\text{-}H}$ = 1.5 Hz, 1H), 7.41 (dd, $^3J_{H\text{-}H}$ = 8.7 Hz, $^4J_{H\text{-}H}$ = 0.8 Hz, 1H), 7.15 (d, $^3J_{H\text{-}H}$ = 3.2 Hz, 1H), 6.65 (dd, $^3J_{H\text{-}H}$ = 3.2 Hz, $^4J_{H\text{-}H}$ = 0.9 Hz, 1H), 3.85 (s, 3H).**^{13}C{^{1}H} NMR** (126 MHz, $CDCl_3$) δ 192.7, 140.1, 130.9, 129.5, 128.4, 126.6, 122.0, 109.9, 103.4, 33.3. **LRMS** *(m/z)* = $[M]^{\bullet+}$ 159.0 (100.0%), $[M - CHO]^+$ 130.05 (61.3%), $[M - Me - CHO]^{\bullet+}$ 115.10 (3.9%).

In accordance with the literature.[16]

1-Methyl-6-(trifluoromethyl)-1_H_-indole 1s (CAS Number: 1146637-26-4)

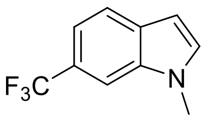

According to RP1, 1-methyl-6-(trifluoromethyl)-1*H*-indole **1s** was obtained from 6-(trifluoromethyl)-1*H*-indole (370 mg, 2.00 mmol, 1.0 equiv.), NaH (60w% in mineral oil, 186 mg, 4.65 mmol, 2.3 equiv.) and MeI (0.19 mL, 3.05 mmol, 1.5 equiv.). The crude material was purified by filtration on a silica pad (with DCM as eluent) to give **1r** as a light yellow solid (382 mg, 1.92 mmol,

96%). ^{1}**H NMR** (400 MHz, $CDCl_3$) δ 7.70 (dt, $^{3}J_{H-H}$ = 8.3 Hz, $^{4}J_{H-H}$ = 0.8 Hz, 1H), 7.61 (m, 1H), 7.35 (dd, $^{3}J_{H-H}$ = 8.3 Hz, $^{4}J_{H-H}$ = 1.5 Hz, 1H), 7.20 (d, $^{3}J_{H-H}$ = 3.1 Hz, 1H), 6.55 (dd, $^{3}J_{H-H}$ = 3.1 Hz, $^{4}J_{H-H}$ = 0.9 Hz, 1H), 3.85 (s, 3H). ^{13}**C{^{1}H} NMR** (126 MHz, $CDCl_3$) δ 135.7, 131.6, 130.9, 125.5 (q, $^{1}J_{C-F}$ = 271 Hz), 123.8 (q, $^{2}J_{C-F}$ = 31.8 Hz), 121.3, 116.1 (q, $^{3}J_{C-F}$ = 3.5 Hz), 106.9 (q, $^{3}J_{C-F}$ = 4.5 Hz), 101.5, 32.9. ^{19}**F{^{1}H} NMR** (377 MHz, $CDCl_3$) δ -60.4. **LRMS** *(m/z)* = $[M]^{\bullet+}$ 199.15 (100.0%), $[M - F]^{+}$ 180.09 (23.55%), $[M - CF_3]^{\bullet+}$ 130.09 (4.9%).

In accordance with the literature.[17,18]

Methyl 1-methyl-1*H*-indole-7-carboxylate 1t (CAS Number: 167479-21-2)

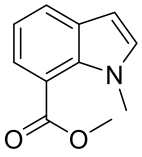

According to RP1, methyl 1-methyl-1*H*-indole-7-carboxylate **1t** was obtained from methyl 1*H*-indole-7-carboxylate **1v** (245 mg, 1.40 mmol, 1.0 equiv.), NaH (60w% in mineral oil, 113 mg, 2.80 mmol, 2.0 equiv.) and MeI (0.13 mL, 2.10 mmol, 1.5 equiv.). The crude material was purified by flash column chromatography (PE/Et_2O 80:20, R_f = 0.46) to give **1t** as a yellowish oil (218 mg, 1.15 mmol, 82%). ^{1}**H NMR** (400 MHz, $CDCl_3$) δ 7.78 (dd, $^{3}J_{H-H}$ = 7.8 Hz, $^{4}J_{H-H}$ = 1.2 Hz, 1H), 7.67 (dd, $^{3}J_{H-H}$ = 7.4 Hz, $^{4}J_{H-H}$ = 1.2 Hz, 1H), 7.10 (t, $^{3}J_{H-H}$ = 7.6 Hz, 1H), 7.06 (d, $^{3}J_{H-H}$ = 3.2 Hz, 1H), 6.55 (d, $^{3}J_{H-H}$ = 3.2 Hz, 1H), 3.97 (s, 3H), 3.89 (s, 3H). ^{13}**C{^{1}H} NMR** (126 MHz, $CDCl_3$) δ 168.1, 133.8, 132.1, 131.2, 125.4, 125.0, 118.5, 116.1, 101.8, 52.1, 37.4. **LRMS** *(m/z)* = $[M]^{\bullet+}$ 189.14 (100.0%), $[M - Me]^{+}$ 174.08 (10.3%), $[M - OMe]^{\bullet+}$ 158.09 (69.0%), $[M - CO_2Me]^{+}$ 130.11 (62.6%).

In accordance with the literature.[19]

1-Methyl-7-nitro-1*H*-indole 1u (CAS Number: 101489-23-0)

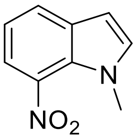

According to RP1, 1-methyl-7- nitro-1*H*-indole **1u** was obtained from 7-nitro-1*H*-indole (240 mg, 1.48 mmol, 1.0 equiv.), NaH (60w% in mineral oil, 155 mg, 3.88 mmol, 2.6 equiv.) and MeI (0.14 mL, 2.25 mmol, 1.5 equiv.). The crude material was purified by flash column chromatography (Pentane/Et_2O 90:10, R_f = 0.51) to give **1u** as a yellow solid (218 mg, 1.24 mmol, 84%). ^{1}**H NMR** (400 MHz, $CDCl_3$) δ 7.85 (dd, $^{3}J_{H-H}$ = 7.8 Hz, $^{4}J_{H-H}$ = 1.1 Hz, 1H), 7.82 (dd, $^{3}J_{H-H}$ = 7.9 Hz, $^{4}J_{H-H}$ = 1.1 Hz, 1H), 7.16 – 7.10 (m, 2H), 6.63 (d, $^{3}J_{H-H}$ = 3.2 Hz, 1H), 3.86 (s, 3H). ^{13}**C{^{1}H} NMR** (126 MHz, $CDCl_3$) δ 136.7, 133.7, 127.7, 127.2, 119.9, 118.6, 102.8, 37.6. **LRMS** *(m/z)* = $[M]^{\bullet+}$ 176.03 (100.0%), $[M - NO_2]^{+}$ 130.20 (68.2%), $[M - NO_2 - Me]^{\bullet+}$ 116.01 (26.8%).

In accordance with the literature.[20]

2-*tert*-Butyl-1-methyl-1*H*-indole 1w (CAS Number: 46275-90-5)

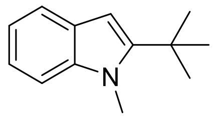

According to RP1, 2-*tert*-butyl-1-methyl-1*H*-indole **1w** was obtained from 2-*tert*-butyl-1*H*-indole (258 mg, 1.49 mmol, 1.0 equiv.), NaH (60w% in mineral oil, 183 mg, 4.57 mmol, 3.1 equiv.) and MeI (0.23 mL, 3.69 mmol, 2.5 equiv.) with a reaction time of 3 hours after the addition of MeI. The crude material was purified by flash column chromatography (PE/DCM 80:20, R_f = 0.59) to give **1w** as a yellow oil (267 mg, 1.43 mmol, 96%). **^{1}H NMR** (400 MHz, $CDCl_3$) δ 7.54 (d, $^3J_{H-H}$ = 7.7 Hz, 1H), 7.29 – 7.24 (m, 2H), 7.21 – 7.13 (m, 1H), 7.11 – 7.04 (m, 1H), 6.31 (d, $^4J_{H-H}$ = 0.9 Hz, 1H), 3.88 (s, 3H), 1.48 (s, 9H). **^{13}C{^{1}H} NMR** (126 MHz, $CDCl_3$) δ 149.3, 138.8, 127.3, 121.0, 120.1, 119.4, 108.7, 98.2, 32.5, 30.4 (3C). The tertiary carbon of the *tert*-butyl group was not detected on the NMR. **LRMS** *(m/z)* = $[M]^{\bullet+}$ 187.18 (69.7%), $[M - Me]^+$ 172.20 (100.0%), $[M - 2 \times Me]^{\bullet+}$ 157.13 (36.8%), [M - *t*Bu]$^+$ 130.09 (15.0%).

In accordance with the literature.[21]

1,3-Dimethyl-1*H*-indole 1y (CAS Number: 875-30-9)

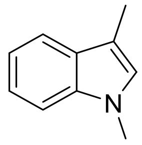

According to RP1, 1,3-dimethyl-1*H*-indole **1y** was obtained from 3-methylindole **1x** (262 mg, 2.00 mmol, 1.0 equiv.), NaH (60w% in mineral oil, 161 mg, 4.04 mmol, 2.0 equiv.) and MeI (0.19 mL, 3.05 mmol, 1.5 equiv.). The crude material was purified by flash column chromatography (PE/Et_2O 90:10, R_f = 0.71) to give **1y** as a yellowish oil (254 mg, 1.75 mmol, 88%). **^{1}H NMR** (400 MHz, $CDCl_3$) δ 7.59 – 7.56 (m, 1H), 7.32 – 7.27 (m, 1H), 7.26 – 7.19 (m, 1H), 7.14 – 7.08 (m, 1H), 6.83 (br s, 1H), 3.74 (s, 3H), 2.34 (s, 3H). **^{13}C{^{1}H} NMR** (126 MHz, $CDCl_3$) δ 137.1, 128.8, 126.6, 121.5, 119.1, 118.6, 110.2, 109.1, 32.6, 9.7. **LRMS** *(m/z)* = $[M]^{\bullet+}$ 145.16 (96.7%), $[M - H]^+$ 144.17 (100.0%), $[M - 2 \times Me]^{\bullet+}$ 115.08 (17.3%).

In accordance with the literature. [22]

1-Methyl-4-(trimethylsilyl)-1*H*-indol-5-yl trifluoromethanesulfonate 1z (CAS Number: 1121707-11-6)

The product was synthetized according to a previously reported procedure by N. Garg *et al.*[23] from 5-benzyloxyindole as commercial staring point. Only the last step is described here.

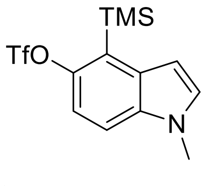

To a solution of 1-methyl-4-(trimethylsilyl)-1*H*-indol-5-yl N-(propan-2-yl)-carbamate (424 mg, 1.39 mmol, 1.0 equiv.) in anhydrous MeCN (4.3 mL) were added DBU (0.52 mL, 3.48 mmol, 2.5 equiv.) and diethylamine (0.22 mL, 2.14 mmol, 1.5 equiv). The resulting mixture was heated at 40 °C for 50 min, then allowed to cool down to room temperature. Next, a solution of *N*-phenyltrifluoromethanesulfonimide (750 mg, 2.10 mmol, 1.5 equiv.) in anhydous MeCN (4.3 mL) was added. The reaction mixture was stirred for 80 min at 25 °C, then passed through a plug

of silica gel (EtOAc as eluent). Evaporation of volatiles under reduced pressure afforded a crude material, which was further purified by flash column chromatography (Pentane/DCM 80:20, R_f = 0.41) to give **1z** as a colourless to yellowish oil (396 mg, 1.13 mmol, 81%). **^{1}H NMR** (400 MHz, $CDCl_3$) δ 7.33 (dd, $^3J_{H-H}$ = 9.0 Hz, $^5J_{H-H}$ = 0.9 Hz, 1H), 7.19 – 7.16 (m, 2H), 6.68 (dd, $^3J_{H-H}$ = 3.3 Hz, $^5J_{H-H}$ = 0.9 Hz, 1H), 3.81 (s, 3H), 0.51 (s, 9H). **^{13}C{^{1}H} NMR** (126 MHz, $CDCl_3$) δ 148.9, 134.5, 133.1, 130.7, 124.1, 118.9 (q, $^1J_{C-F}$ = 321 Hz), 114.2, 111.5, 103.6, 33.3, 1.2. **^{19}F{^{1}H} NMR** (377 MHz, $CDCl_3$) δ -73.0. **LRMS** *(m/z)* = $[M]^{\bullet+}$ 351.03 (24.3%), $[M - Me]^+$ 336.00 (11.4%), $[M - Tf]^+$ 218.09 (54.4%), $[M - TfO]^+$ 203.07 (64.6%), $[M - Tf - 2 \times Me]^{\bullet+}$ 188.04 (25.6%), $[M - Tf - TMS - H]^{\bullet+}$144.07 (100.0%).

In accordance with the literature.[23]

1-Benzylimidazole 1ac (CAS Number: 4238-71-5)

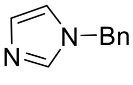

NaH (60w% in mineral oil, 129 mg, 3.22 mmol, 1.5 equiv.) was added to a solution of imidazole (146 mg, 2.14 mmol, 1.0 equiv.) in anhydrous THF (4 mL) at 0 °C under an argon atmosphere. The heterogeneous mixture was stirred at 0 °C for 15 min and it was allowed to warm up to 25 °C for 1 h. Then, the cloudy solution was cooled down to 0 °C, benzyl bromide (0.33 mL, 2.76 mmol, 1.3 equiv.) was added and the solution was allowed to warm to 25 °C. After total consumption of the starting imidazole, the reaction mixture was cooled again to 0 °C, quenched with sat. aq. NH_4Cl (4 mL), H_2O (10 mL), and the aqueous layer was extracted with Et_2O (3 x 10 mL). The combined organic layers were washed with brine (10 mL), dried over anhydrous $MgSO_4$ and concentrated under reduced pressure. The resulting material was purified by flash column chromatography (DCM/MeOH 90:10, R_f = 0.47) to give **1ac** as a yellow oil (213 mg, 1.35 mmol, 63%). **^{1}H NMR** (400 MHz, $CDCl_3$) δ 7.55 (app. s, 1H), 7.40 – 7.29 (m, 3H), 7.20 –7.13 (m, 2H), 7.09 (t, $^3J_{H-H}$ = 1.1 Hz, 1H), 6.90 (t, $^3J_{H-H}$ = 1.3 Hz, 1H), 5.12 (s, 2H). **^{13}C{^{1}H} NMR** (126 MHz, $CDCl_3$) δ 137.6, 136.4, 130.0, 129.1 (2C), 128.4, 127.4 (2C), 119.4, 50.9. **LRMS** *(m/z)* = $[M]^{\bullet+}$ 158.12 (60.3%), $[Bn]^{\bullet+}$ 91.10 (100.0%), $[M - Bn]^+$ 65.06 (17.4%).

In accordance with the literature.[24]

1-Benzyl-1*H*-pyrrole 1ae (CAS Number : 2051-97-0)

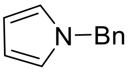

NaH (60w% in mineral oil, 162 mg, 4.04 mmol, 2.0 equiv.) was added to a solution of pyrrole (0.14 mL, 2.00 mmol, 1.0 equiv.) in anhydrous THF (4 mL) at 0 °C under an argon atmosphere. The heterogeneous mixture was stirred at 0 °C. Then, benzyl bromide (0.36 mL, 3.01 mmol, 1.5 equiv.) was added and the solution was allowed to warm to 25 °C. After total consumption of the starting pyrrole, the reaction mixture was cooled again to 0 °C, quenched with sat. NH_4Cl (4 mL) and H_2O (10 mL) and the aqueous layer was extracted with Et_2O (3 x 10 mL). The combined organic layers were washed with brine (10 mL), dried over anhydrous $MgSO_4$ and concentrated under reduced pressure. The resulting material was purified by flash column chromatography (PE/Et_2O 95:5, R_f = 0.67) to give **1ae** as a yellow oil (233 mg, 1.48 mmol, 74%). **^{1}H NMR** (400 MHz, $CDCl_3$) δ 7.36 – 7.27 (m, 3H), 7.14 – 7.10 (m, 2H), 6.70 (t, $^3J_{H-H}$ = 2.1 Hz, 2H), 6.20 (t, $^3J_{H-H}$ = 2.1 Hz, 2H), 5.08 (s, 2H). **^{13}C{^{1}H} NMR** (126 MHz, $CDCl_3$) δ 138.2, 128.9 (2C), 127.8, 127.1, 121.3 (2C), 108.6, 53.5. **LRMS** *(m/z)* = $[M]^{\bullet+}$ 157.10 (61.4%), $[Bn]^{\bullet+}$ 91.10 (100.0%), $[M - Bn]^+$ 65.05 (15.9%).

In accordance with the literature.[25]

III. Optimization of the reaction conditions

Warning: the reaction generates fluorophosgene, an extremely toxic gas. Experiments must be carried out under a well-ventilated fume hood, especially for the opening of the tube at the end of the reaction.

All reactions were conducted on 0.50 mmol of 1-methylindole **1a** with the following procedure. In a 10 mL screw-cap tube closed by a septum, 2,4-dinitro-1-(trifluoromethoxy)benzene (DNTFB) was added to a DMAP solution in anhydrous MeCN (5 mL) under argon and the septum was quickly replaced by the screw-cap. The solution was stirred for 20 min at 25 °C. The tube was then opened to add 1-methylindole (64 µL, 0.50 mmol, 1.0 equiv.) and closed again rapidly. The solution was stirred at 80 °C for the indicated time. The tube was allowed to cool down to room temperature before the addition of toluene (53 µL, 0.50 mmol, 1.0 equiv.) as internal standard for quantitative NMR analysis.

*Table S1: Optimization of the fluoroformylation of 1-methylindole **1a***

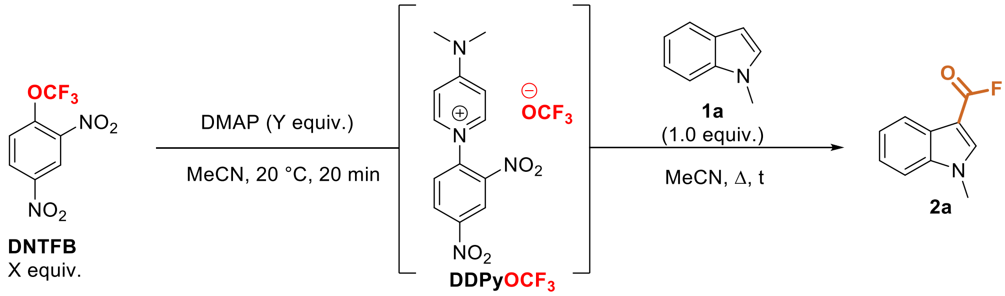

Entry	DNTFB (equiv.)	DMAP (equiv.)	Conditions	^{1}H NMR yield[a] (isolated)
1	18.1	9.1	80 °C / 16 h	84% (80%)
2	1.9	2.0	40 °C / 16 h	No conversion
3	2.1	2.1	80 °C / 16 h	Traces
4	9.1	9.0	80 °C / 16 h	0%
5	4.0	2.0	80 °C / 16 h	85%
6	8.0	4.0	80 °C / 16 h	82%
7	2.0	1.0	80 °C / 16 h	82%
8	4.0	2.0	80 °C / 4 h	86%
9	4.0	2.0	80 °C / 1 h	85%
10	4.0	3.0	80 °C/ 1 h	67%
11[b]	1.0	0.5	80 °C/ 70 min	85%[c]
12	4.0	2.0	80 °C / 30 min	80%
13[b]	4.0	2.0	80 °C / 16 h	86%
14[d]	4.0	2.0	80 °C / 16 h	86%
**15[b,d,e]**	**2.0**	**1.0**	**80 °C / 1 h**	**91% (84%)**

a) Toluene was used as internal standard. b) 1.5 mL of MeCN were used. c) Incomplete conversion. d) Reagent grade MeCN. e) No $DDPyOCF_3$ preformation step was carried out.

When 9.1 equiv. of DNTFB and 9.0 equiv. of DMAP were used the reaction mixture was purified in order to identify the side-product coming from **1a** formed during the reaction. After 16h, the tube was allowed to cool down to room temperature and 10 mL of H_2O were added to the crude mixture in a separatory funnel. The aqueous layer was extracted with DCM (3 x 10 mL), and the combined organic layers were dried over $MgSO_4$ and concentrated under reduced pressure. The resulting material was purified by flash column chromatography (pentane/Et_2O 90:10, R_f = 0.50) to give a product that we assume to be 2,2-difluoro-1-methyl-2,3-dihydro-1*H*-indol-3-one **5** (3.2 mg, 0.02 mmol, 4%).

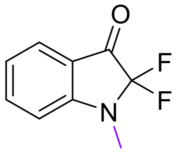

5: **^{1}H NMR** (400 MHz, $CDCl_3$) δ 7.64 – 7.53 (m, 2H), 6.93 (td, $^3J_{H-H}$ = 7.5 Hz, $^4J_{H-H}$ = 0.8 Hz, 1H), 6.75 (d, $^3J_{H-H}$ = 8.1 Hz, 1H), 3.02 (t, $^{TS}J_{C-F}$ = 1.3 Hz). **^{19}F{^{1}H} NMR** (377 MHz, $CDCl_3$) δ -107.4. **LCMS** *(m/z)* = $[M]^{\bullet+}$ 183.03 (100.0%), $[M – NCH_3]^+$ 154.03 (57.0%), $[Ph\text{-}NCH_3]^+$ 105.05 (47.1%).

During the ^{13}C{^{1}H} NMR analysis of **5**, the product underwent hydrolysis to 1-methylindole-2,3-dione **5'** in the NMR tube.

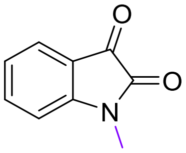

5': **^{1}H NMR** (400 MHz, $CDCl_3$) δ 7.61 (m, 2H), 7.13 (td, $^3J_{H-H}$ = 7.5 Hz, $^4J_{H-H}$ = 0.8 Hz, 1H), 6.89 (dd, $^3J_{H-H}$ = 8.3 Hz, $^4J_{H-H}$ = 0.9 Hz, 1H), 3.26 (s, 3H). **^{13}C{^{1}H} NMR** (126 MHz, $CDCl_3$) δ 183.3, 158.2, 151.5, 138.4, 125.3, 123.8 (2 x C), 117.5, 109.9 (2 x C), 26.2. **LCMS** *(m/z)* = $[M]^{\bullet+}$ 161.02 (74.4%), $[M – CO]^+$ 133.03 (29.0%), $[Ph\text{-}C(O)]^{\bullet+}$ 104.04 (100.0%).

Data in accordance with the literature.[26]

Compound **5** was observed in large amounts by GC-MS analysis of the reaction mixture involving 9.1 equiv. of DNTFB and 9.0 equiv. of DMAP. It was also observed as traces when 2.0 equiv. of both DNTFB and DMAP were used. A side-reaction, whose mechanism remains undetermined, takes place when a 1:1 ratio between DNTFB and DMAP is employed. This side-reaction prevents the formation of the desired acyl fluoride. The fluoroformylation hence required an excess of DNTFB compared to DMAP.

IV. Control experiments

A. Fluoroformylation of 1-methylindole 1a without DMAP

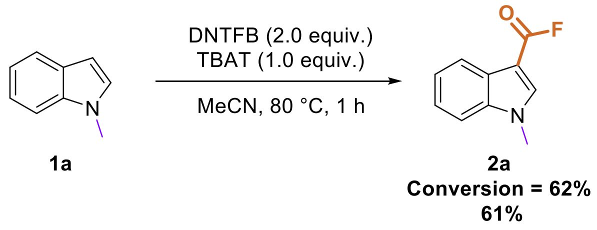

In a 10 mL screw-cap tube, DNTFB (0.16 mL, 1.03 mmol, 2.1 equiv.) was added to a solution of tetrabutylammonium difluorotriphenylsilicate (TBAT) (270 mg, 0.50 mmol, 1.0 equiv.) and **1a** (64 µL, 0.50 mmol, 1.0 equiv.) in MeCN (1.5 mL). The tube was closed by a screw-cap and the solution was stirred at 80 °C for 60 min. The tube was allowed to cool down to room temperature before the addition of bromochloromethane (32 µL, 0.50 mmol, 1.0 equiv.) as internal standard for quantitative NMR analysis. The quantitative ^{1}H NMR analysis gave a 62% conversion of the starting indole **1a** and an NMR yield of 61% of the desired fluoroformylated indole **2a**. This result suggests that fluoride is a competent enough base to trap the proton released during the rearomatization of the indole during the S_EAr process.

B. Fluoroformylation of 1-methylindole 1a with a two-tube set-up

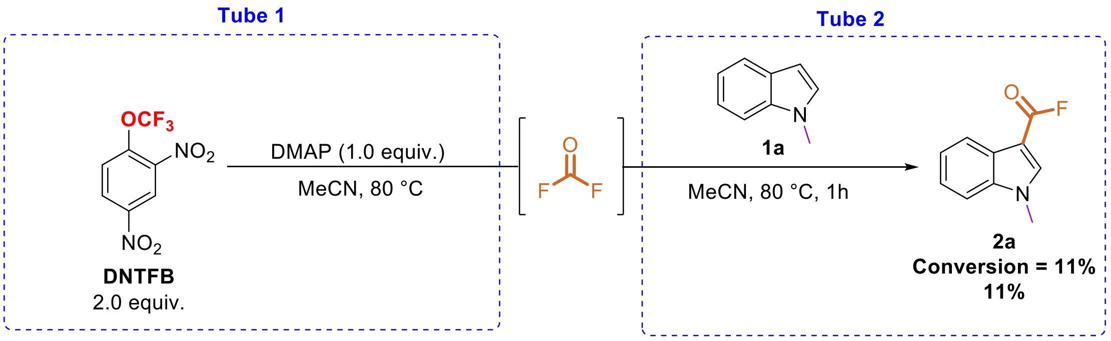

In a 5 mL crimp-cap heavy-wall tube equipped with a septum (***Tube 1***), DNTFB (0.16 mL, 1.03 mmol, 2.1 equiv.) was added to a solution of DMAP (63.7 mg, 0.52 mmol, 1.0 equiv.) in MeCN (1.5 mL) (***Tube 1***). In a second crimp-cap heavy-wall tube (***Tube 2***), 1-methylindole (64 µL, 0.50 mmol, 1.0 equiv.) was solubilized in MeCN (1.5 mL) (***Tube 2***). The two tubes were closed and crimped, connected by a cannula (with the cannula dipped in the solution of ***Tube 2***) and stirred at 80 °C for 60 min. The tube was allowed to cool down to room temperature before the addition of bromochloromethane (32 µL, 0.50 mmol, 1.0 equiv.) as internal standard for quantitative NMR analysis (CAUTION: the cannula must be removed before the tube is extracted from the heating aluminum block to prevent the transfer of the ***Tube 2*** solution into the ***Tube 1***). The quantitative ^{1}H NMR analysis gave a 11% conversion of the starting indole **1a** and an NMR yield of 11% of the

desired fluoroacylated indole **2a**. This result suggests that DNTFB and its derivatives —or more precisely, the dinitrophenyl motif of DNTFB or of resulting species— do not play a pivotal role in the fluoroformylation step. The reaction is also able to proceed with fluorophosgene only; the modest 11% yield obtained is due to the low amount of fluorophosgene solubilized in the solution of ***Tube 2***.

C. Reaction of 1-methylindole 1a with DNTFB without DMAP

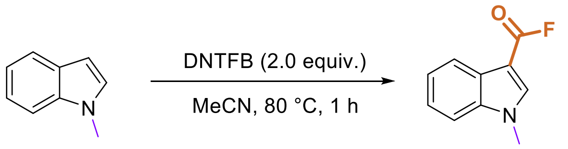

In a 10 mL screw-cap tube, DNTFB (0.16 mL, 1.03 mmol, 2.1 equiv.) was added to a solution of **1a** (64 µL, 0.50 mmol, 1.0 equiv.) in MeCN (1.5 mL). The tube was closed by a screw-cap and the solution was stirred at 80 °C for 60 min. The tube was allowed to cool down to room temperature before the addition of 4-trifluoromethylanisole (70 µL, 0.49 mmol, 1.0 equiv.) as internal standard for quantitative ^{1}H et ^{19}FNMR analyses. The latter indicated no conversion of both indole **1a** and DNTFB. This confirmed that DNFTB does not play a pivotal role in the fluoroformylating step. It also indicated that **1a** does not activate DNTFB to generate the trifluoromethoxide anion.

D. Reaction of 1-methylindole 1a with DNTFB and a catalytic amount of DMAP

In a 10 mL screw-cap tube, DNTFB (0.08 mL, 0.52 mmol, 1.0 equiv.) was added to a solution of DMAP (7.5 mg, 0.12 mmol, 0.1 equiv.) and 1-methylindole **1a** (64 µL, 0.50 mmol, 1.0 equiv.) in MeCN (1.5 mL). The solution was stirred at 80 °C for 60 min. The tube was then allowed to cool down before the addition of bromochloromethane (32 µL, 0.50 mmol, 1.0 equiv.) as internal standard for quantitative ^{1}H NMR. NMR analysis gave approximatively 15% conversion of **1a** and a 15% yield of **2a**. This indicates that a sufficient amount of DMAP has to be employed to activate the DNTFB to reach a full conversion of **1a** and a good fluoroformylation yield in an interesting time scale. Moreover, ^{19}F NMR analysis of the reaction mixture revealed the presence of the DDPy^{+}HF$_{2}^{-}$ salt. This salt, already described by Sanford *et al.*[27] confirmed the role of fluoride ions in the rearomatization step in the proposed mechanism.

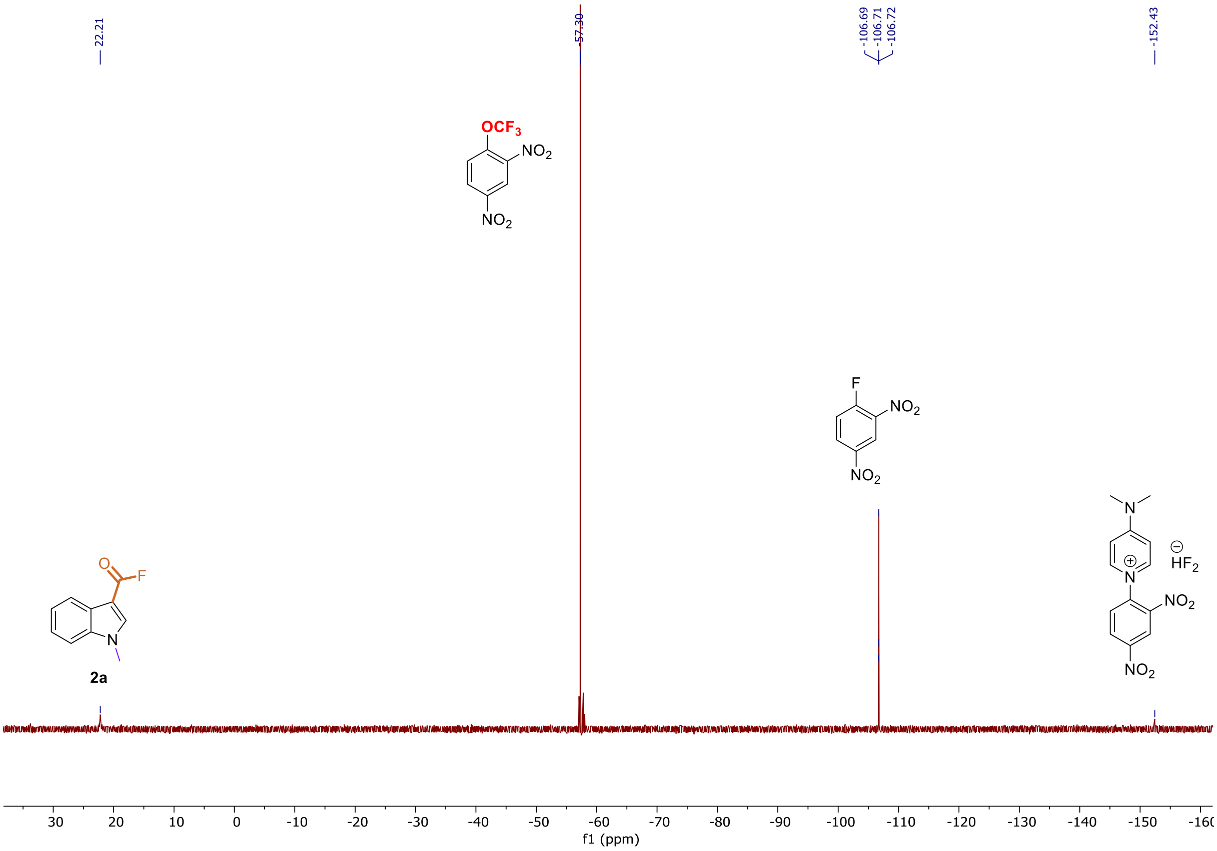
22.21
-57.30
-106.69
-106.71
-106.72
-152.43
OCF3
NO2
NO2
F
NO2
NO2
O
F
N
2a
N
N
HF2
NO2
NO2
30 20 10 0 -10 -20 -30 -40 -50 -60 -70 -80 -90 -100 -110 -120 -130 -140 -150
f1 (ppm)

V. DFT calculation of the nucleophilicity of indoles and other heteroaromatic rings with ESNuEL

The trends observed experimentally in terms of activity of substrates in the fluoroformylation reaction agree quite well with the estimation of their nucleophilicity by the EsNuEL tool (https://www.esnuel.org/). This website allowed us to calculate the nucleophilicity of our substrate on reactive positions observed during our scope study. They were estimated through calculations of their methyl cation affinities (MCA) based on r^{2}SCAN-3c SMD(DMSO) single-point calculations on GFN1-xTB ALPB(DMSO) level of theory. This web application is based on the work of Jensen *et al.*[28]

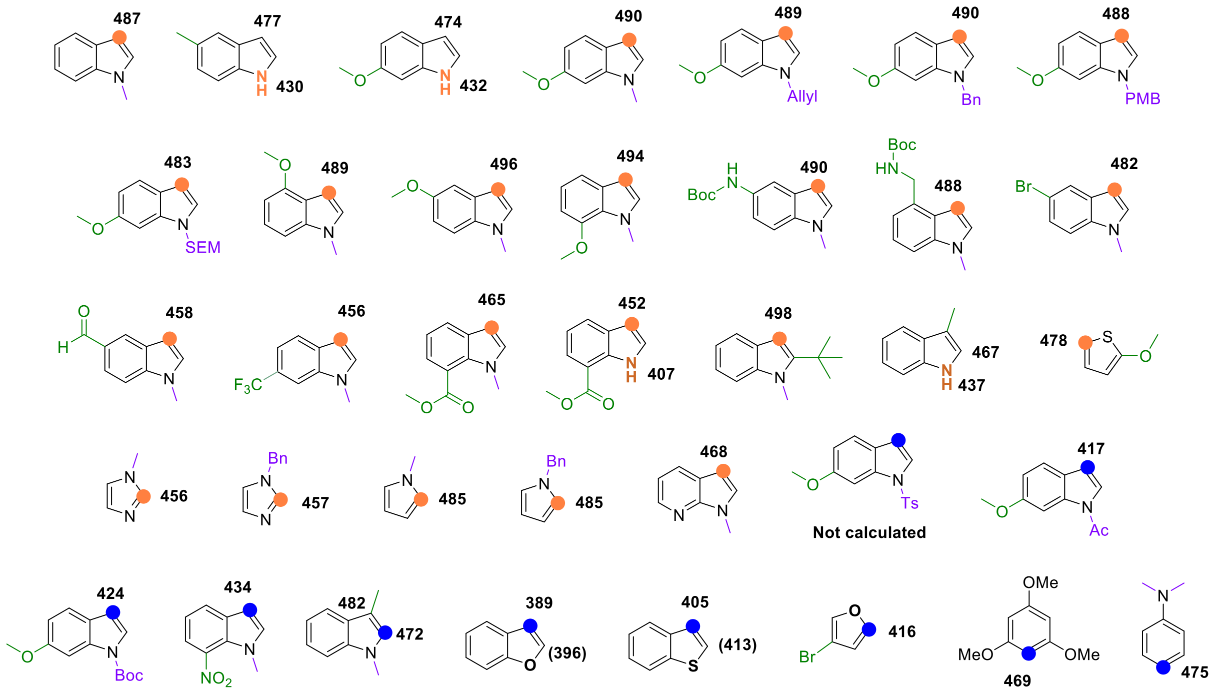

Figure S1 : Calculated nucleophilicity (in kJ/mol) of fluoroformylated positions (orange dots) or expected site of fluoroformylation (blue dots) based on r²SCAN-3c SMD(DMSO) single-point calculations on GFN1-xTB ALPB(DMSO) level of theory. The web application was not able to calculate a value for the N-tosyl-1H-indole derivative.

By comparing calculated values with reaction results we could exhibit a qualitative link between the MCA values of the expected site of reaction and the ability of the substrate to react under our conditions. Indeed, indoles *N*-protected by an EWG and 7-nitro-*1H*-methylindole showed no conversion under our conditions, which can be linked with calculated MCA values lower than 435 kJ/mol on positions expected to react (blue dots, Figure S1). The same assumption could be made for 2-bromofurane, benzofurane and benzothiophene. For 3-methyl-*1H*-methylindole, the MCA value of the *C3* position is equivalent to the one of 5-bromo-*1H*-methylindole, but the latter did not show any conversion under our conditions due to the methyl blocking the position. The free *C2* position was not reactive under our conditions despite a high MCA value of 471.67 kJ/mol. The hindrance of the position is then also an important factor. Finally, electron-rich aromatic rings like 1,3,5-trimethoxybenzene or *N,N*-dimethylaniline were not reactive under our conditions despite MCA values of 469.02 kJ/mol and 475.30 kJ/mol respectively. A high MCA value is not sufficient to allow the fluoroformylation to occur, and a heteroatomatic ring seems to be mandatory.

VI. Fluoroformylation of indoles and other heteroaromatic cycles

Warning: the reaction generates fluorophosgene, an extremely toxic gas. Experiments must be carried out under a well-ventilated fumehood, especially for the opening of the tube at the end of the reaction.

Note: Occasionally, trace signals of 1-fluoro-2,4-dinitrobenzene (Sanger's reagent, formed as side-product of the reaction), were observed in the purified fluoroformylation products, since it sometimes proved difficult to remove totally.

Optimized procedure 1 (OP1) for the fluoroformylation of indoles

In a 10 mL screw-cap tube, DNTFB (0.16 mL, 1.03 mmol, 2.0 equiv.) was added to a solution of DMAP (61.1 mg, 0.50 mmol, 1.0 equiv.) and indole or other heteroaromatic cycle (0.50 mmol, 1.0 equiv.) in MeCN (1.5 mL). The solution was stirred at 80 °C for 60 min. The tube was then allowed to cool down to room temperature and 10 mL of H_2O were added to the crude mixture in a separatory funnel. The aqueous layer was extracted with DCM (3 x 10 mL), and the combined organic layers were dried over $MgSO_4$ and concentrated under reduced pressure. The resulting material was purified by flash column chromatography with the appropriate eluent system (given in V:V).

Substrates **1a-c**, **1m**, **1q**, **1v, 1x**, **1aa, 1ab** and **1ad** were purchased from Doug discovery and BLD Pharm and used without further purification.

1-Methyl-1*H*-indole-3-carbonyl fluoride 2a (CAS Number: 2232892-44-1)

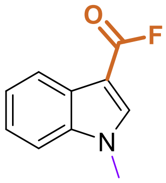

According to OP1, 1-methyl-1*H*-indole-3-carbonyl fluoride **2a** was obtained from 1-methyl-1*H*-indole **1a** (64 µL, 0.50 mmol, 1.0 equiv.), DNTFB (0.16 mL, 2.01 mmol, 2.1 equiv.) and DMAP (61.2 mg, 0.50 mmol, 1.0 equiv.). The crude material was purified by flash column chromatography (Pentane/Et_2O 70:30, R_f = 0.43) to give **2a** as a light-pink solid (74.2 mg, 0.42 mmol, 84%). **^{1}H NMR** (400 MHz, $CDCl_3$) δ 8.14 – 8.07 (m, 1H), 7.86 (s, 1H), 7.44 – 7.33 (m, 3H), 3.90 (s, 3H).**^{13}C{^{1}H} NMR** (126 MHz, $CDCl_3$) δ 154.7 (d, $^1J_{C-F}$ = 327 Hz), 137.9, 137.6 (d, $^4J_{C-F}$ = 2.7 Hz), 127.1 (d, $^3J_{C-F}$ = 6.3 Hz), 124.0, 123.2, 121.4, 110.4, 101.2 (d, $^2J_{C-F}$ = 70.1 Hz), 33.98. **^{19}F{^{1}H} NMR** (377 MHz, $CDCl_3$) δ 22.7. **IR** (ATR) 2921 (w), 1775 (s), 1652 (w), 1535 (s), 1464 (m), 1372 (m), 1341 (m), 1283 (w), 1261 (w), 1239 (w), 1206 (s), 1129 (m), 1077 (m), 1043 (m), 1009 (w), 948 (m), 914 (s), 773 (m), 748 (s), 730 (s). **HRMS-ESI** *(m/z)* $[M]^+$ calculated for $C_{10}H_8ONF$, 200.0482; found, 200.0479. **mp**: 115.4 °C.

In accordance with the literature.[29]

5-Methyl-1*H*-indole-1-carbonyl fluoride 2b

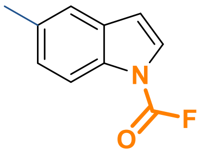

According to OP1, 5-methyl-1*H*-indole-1-carbonyl fluoride **2b** was obtained from 5-methyl-1*H*-indole **1b** (65.1 mg, 0.50 mmol, 1.0 equiv.), DNTFB (0.16 mL, 2.01 mmol, 2.1 equiv.) and DMAP (62.3 mg, 0.51 mmol, 1.0 equiv.). The crude material was purified by flash column chromatography (Pentane/DCM 90:10, R_f = 0.52) to give **2b** as a colourless oil (53.6 mg, 0.30 mmol, 61%). **^{1}H NMR** (400 MHz, $CDCl_3$) δ 8.03 (br s, 1H), 7.39 (d, $^4J_{H-H}$ = 1.6 Hz, 1H), 7.36 (s, 1H), 7.23 (d, $^3J_{H-H}$ = 8.6 Hz, 1H), 6.64 (s, 1H), 2.46 (s, 3H). **^{13}C{^{1}H} NMR** (126 MHz, $CDCl_3$) δ 140.2 (d, $^1J_{C-F}$ = 307 Hz), 134.4, 133.5, 130.9, 126.9, 124.5, 121.5, 114.4 (d, $^{TS}J_{C-F}$ = 3.2 Hz), 110.7, 21.3. **^{19}F{^{1}H} NMR** (377 MHz, $CDCl_3$) δ -5.6 (br s, 0.79F), -7.1 (br s, 0.21F) (rotamers signals). **IR** (ATR): 3149 (w), 3029 (w), 2927 (w), 2860 (w), 1809 (s), 1587 (w), 1547 (w), 1464 (m), 1381 (s), 1344 (m), 1329 (m), 1286 (w), 1249 (m), 1212 (m), 1178 (m), 1145 (w), 1114 (m), 1086 (w), 1074 (w), 1016 (w), 969 (m), 886 (w), 807 (m), 764 (m), 742 (m), 711 (m), 628 (w). **HRMS-ESI** *(m/z)* $[M]^+$ calculated for $C_{10}H_8FNO$, 177.0584; found, 177.0579.

6-Methoxy-1*H*-indole-1-carbonyl fluoride 2c

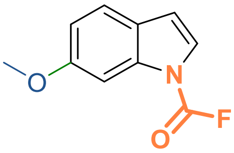

According to OP1, 6-methoxy-1*H*-indole-1-carbonyl fluoride **2c** was obtained from 6-methoxy-1*H*-indole **1c** (72.5 mg, 0.49 mmol, 1.0 equiv.), DNTFB (0.16 mL, 2.01 mmol, 2.1 equiv.) and DMAP (61.1 mg, 0.50 mmol, 1.0 equiv.). The crude material was purified by flash column chromatography (Pentane/Et_2O 80:20, R_f = 0.80) to give **2c** as a yellowish solid (57.7 mg, 0.30 mmol, 61%). **^{1}H NMR** (400 MHz, $CDCl_3$) δ 7.73 (s, 1H), 7.46 (d, $^3J_{H-H}$ = 8.6 Hz, 1H), 7.29 (s, 1H), 6.96 (dd, $^3J_{H-H}$ = 8.6 Hz, $^4J_{H-H}$ = 2.3 Hz, 1H), 6.64 (s, 1H), 3.90 (s, 3H). **^{13}C{^{1}H} NMR** (126 MHz, $CDCl_3$) δ 158.7, 140.3 (d, $^1J_{C-F}$ = 301 Hz), 136.5, 124.0, 123.1, 122.0, 113.7, 110.7, 99.2, 55.7. **^{19}F{^{1}H} NMR** (377 MHz, $CDCl_3$) δ -4.7. **IR** (ATR) 3152 (w),3214 (w), 2921 (w), 1809 (s), 1615 (m), 1541 (m), 1492 (m), 1440 (m), 1387 (m), 1338 (s), 1286 (m), 1249 (m), 1249 (m), 1218 (s), 1178 (m), 1129 (m), 1101 (m), 1086 (m), 1025 (m), 976 (m), 917 (s), 880 (m), 843 (m), 813 (s), 760 (m), 742 (s), 720 (s). **HRMS-ESI** *(m/z)* $[M]^+$ calculated for $C_{10}H_9FNO_2$, 194.0612; found, 194.0610. **mp**: 75.2 °C.

6-Methoxy-1-methyl-1*H*-indole-3-carbonyl fluoride 2d

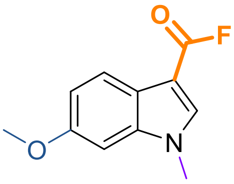

According to OP1, 6-methoxy-1-methyl-1*H*-indole-3-carbonyl fluoride **2d** was obtained from 6-methoxy-1-methyl-1*H*-indole **1d** (80.9 mg, 0.50 mmol, 1.0 equiv.), DNTFB (0.15 mL, 0.97 mmol, 1.9 equiv.) and DMAP (61.7 mg, 0.51 mmol, 1.0 equiv.). The crude material was purified by

flash column chromatography (Pentane/Et_2O 50:50, R_f = 0.30) to give **1d** as a yellowish solid (75.3 mg, 0.36 mmol, 72%). Also synthetized on 1.0 mmol scale (186.6 mg, 0.90 mmol, 90 %). **^{1}H NMR** (400 MHz, $CDCl_3$) δ 7.96 (dd, $^3J_{H-H}$ = 8.6 Hz, $^5J_{H-H}$ = 1.7 Hz, 1H), 7.76 (s, 1H), 7.00 (dd, $^3J_{H-H}$ = 8.8 Hz, $^4J_{H-H}$ = 2.2 Hz, 1H), 6.83 (d, $^4J_{H-H}$ = 2.2 Hz, 1H), 3.90 (s, 3H), 3.83 (s, 3H). **^{13}C{^{1}H} NMR** (126 MHz, $CDCl_3$) δ 157.6, 154.6 (d, $^1J_{C-F}$ = 327 Hz), 138.3 (d, $^4J_{C-F}$ = 2.9 Hz), 136.9, 121.9, 120.8 (d, $^3J_{C-F}$ = 6.2 Hz), 112.5, 101.1 (d, $^2J_{C-F}$ = 70.1 Hz), 93.9, 55.7, 33.8. **^{19}F{^{1}H} NMR** (377 MHz, $CDCl_3$) δ 22.1. **IR** (ATR) 3131 (w), 3080 (w), 2942 (br w), 1769 (s), 1624 (m), 1580 (w), 1533 (m), 1508 (m), 1453 (m), 1446 (m), 1406 (m), 1370 (m), 1312 (w), 1243 (s), 1196 (m), 1127 (m), 1080 (m), 1044 (s), 939 (s), 892 (m), 841 (s), 812 (s), 754 (m), 732 (s). **HRMS-ESI** *(m/z)* $[M+Na]^+$ calculated for $C_{11}H_{10}FNO_2Na$, 230.0588; found, 230.0583. **mp**: 133.1 °C.

6-Methoxy-1-(prop-2-en-1-yl)-1*H*-indole-3-carbonyl fluoride 2e

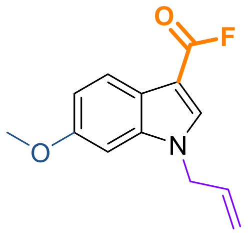

According to OP1, 6-methoxy-1-(prop-2-en-1-yl)-1*H*-indole-3-carbonyl fluoride **2e** was obtained from 6-methoxy-1-(prop-2-en-1-yl)-1*H*-indole **1e** (95.9 mg, 0.51 mmol, 1.0 equiv.), DNTFB (0.16 mL, 1.03 mmol, 2.0 equiv.) and DMAP (65.3 mg, 0.53 mmol, 1.0 equiv.). The crude material was purified by flash column chromatography (Pentane/Et_2O 85:15, R_f = 0.22) to give **2e** as a yellowish solid (90.8 mg, 0.39 mmol, 76%). **^{1}H NMR** (400 MHz, $CDCl_3$) δ 7.97 (dd, $^3J_{H-H}$ = 8.7 Hz, $^5J_{H-H}$ = 1.7 Hz, 1H), 7.80 (s, 1H), 6.99 (dd, $^3J_{H-H}$ = 8.8 Hz, $^4J_{H-H}$ = 2.2 Hz, 1H), 6.83 (d, $^4J_{H-H}$ = 2.2 Hz, 1H), 6.01 (ddt, $^3J_{H-H\ trans}$ = 17.1 Hz, $^3J_{H-H\ cis}$ = 10.2 Hz, $^3J_{H-H}$ = 5.5 Hz, 1H), 5.34 (dq, $^3J_{H-H\ cis}$ = 10.4 Hz, $^4J_{H-H}$ = 1.3 Hz, 1H), 5.24 – 5.16 (m, 1H), 4.74 (dt, $^3J_{H-H}$ = 5.5 Hz, $^3J_{H-H}$ = 1.6 Hz, 2H), 3.87 (s, 3H). **^{13}C{^{1}H} NMR** (126 MHz, $CDCl_3$) δ 157.7, 154.8 (d, $^1J_{C-F}$ = 327 Hz), 137.9 (d, $^4J_{C-F}$ = 3.0 Hz), 136.2, 131.5, 122.1, 121.1 (d, $^3J_{C-F}$ = 6.0 Hz), 119.4, 112.6, 101.7 (d, $^2J_{C-F}$ = 69.9 Hz), 94.6, 55.8, 49.8. **^{19}F{^{1}H} NMR** (377 MHz, $CDCl_3$) δ 22.4. **IR** (ATR) 3112 (w), 2961 (w), 1769 (s), 1618 (m), 1575 (w), 1526 (m), 1501 (m), 1452 (m), 1403 (m), 1378 (m), 1249 (s), 1206 (m), 1178 (m), 1071 (m), 1034 (s), 994 (m), 948 (s), 911 (m), 837 (m), 813 (s), 757 (m), 730 (m). **HRMS-ESI** *(m/z)* $[M]^+$ calculated for $C_{13}H_{12}FNO_2$, 233.0847; found, 233.0843. **mp**: 78.6 °C.

1-Benzyl-6-methoxy-1*H*-indole-3-carbonyl fluoride 2f

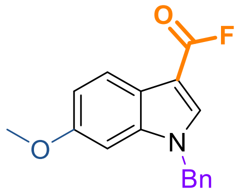

According to OP1, 1-benzyl-6-methoxy-1*H*-indole-3-carbonyl fluoride **2f** was obtained from 1-benzyl-6-methoxy-1*H*-indole **1f** (120 mg, 0.51 mmol, 1.0 equiv.), DNTFB (0.16 mL, 1.03 mmol, 2.0 equiv.) and DMAP (61.7 mg, 0.51 mmol, 1.0 equiv.). The crude material was purified by flash column chromatography (Pentane/Et_2O 85:15, R_f = 0.24) to give **2f** as a light orange solid (108 mg, 0.38 mmol, 75%). **^{1}H NMR** (400 MHz, $CDCl_3$) δ 7.98 (dd, $^3J_{H-H}$ = 8.8 Hz, $^4J_{H-H}$ = 1.6 Hz, 1H), 7.79 (s, 1H), 7.44 – 7.30 (m, 3H), 7.22 – 7.14 (m, 2H), 6.99 (dd, $^3J_{H-H}$ = 8.8 Hz, $^4J_{H-H}$ = 2.2 Hz, 1H), 6.79 (d, $^4J_{H-H}$ = 2.2 Hz, 1H), 5.31 (s, 2H), 3.82 (s, 3H). **^{13}C{^{1}H} NMR** (126 MHz, $CDCl_3$) δ 157.8, 154.8 (d, $^1J_{C-F}$ = 328 Hz), 138.1 (d,

$^4J_{C\text{-}F}$ = 3.1 Hz), 136.4, 135.1, 129.4, 128.7, 127.4, 122.2, 121.1 (d, $^3J_{C\text{-}F}$ = 5.8 Hz), 112.6, 101.9 (d, $^2J_{C\text{-}F}$ = 70.0 Hz), 94.8, 55.8, 51.2. **^{19}F{^{1}H} NMR** (377 MHz, $CDCl_3$) δ 22.5. **IR** (ATR) 3120 (w), 3033 (w), 2939 (w), 1776 (s), 1530 (w), 1501 (w), 1468 (w), 1450 (w), 1403 (m), 1356 (w), 1327 (m), 1236 (m), 1077 (m), 1048 (s), 979 (s), 884 (m), 754 (m), 725 (s), 696 (m). **HRMS-ESI** *(m/z)* $[M]^+$ calculated for $C_{17}H_{14}FNO_2$, 283.1003; found, 283.1011. **mp**: 110.7 °C.

6-Methoxy-1-[(4-methoxyphenyl)methyl]-1*H*-indole-3-carbonyl fluoride 2g

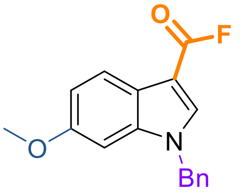

According to OP1, 6-methoxy-1-[(4-methoxyphenyl)methyl]-1*H*-indole-3-carbonyl fluoride **2g** was obtained from 6-methoxy-1-[(4-methoxyphenyl)methyl]-1*H*-indole **1g** (137 mg, 0.51 mmol, 1.0 equiv.), DNTFB (0.16 mL, 1.03 mmol, 2.0 equiv.) and DMAP (61.2 mg, 0.50 mmol, 1.0 equiv.). The crude material was purified by flash column chromatography (Pentane/Et_2O 70:30, R_f = 0.32) to give **2g** as an off-white solid (129 mg, 0.41 mmol, 80%). **^{1}H NMR** (400 MHz, $CDCl_3$) δ 7.97 (dd, $^3J_{H\text{-}H}$ = 8.7 Hz, $^4J_{H\text{-}H}$ = 1.6 Hz, 1H), 7.75 (s, 1H), 7.18 – 7.11 (m, 2H), 6.98 (dd, $^3J_{H\text{-}H}$ = 8.7 Hz, $^4J_{H\text{-}H}$ = 2.2 Hz, 1H), 6.93 – 6.87 (m, 2H), 6.82 (d, $^4J_{H\text{-}H}$ = 2.2 Hz, 1H), 5.23 (s, 2H), 3.83 (s, 3H), 3.80 (s, 3H). **^{13}C{^{1}H} NMR** (126 MHz, $CDCl_3$) δ 159.8, 154.7 (d, $^1J_{C\text{-}F}$ = 327 Hz), 137.9 (d, $^4J_{C\text{-}F}$ = 2.8 Hz), 136.2, 128.9, 126.7, 122.0, 121.1 (d, $^3J_{C\text{-}F}$ = 5.6 Hz), 114.6, 112.5, 101.6 (d, $^2J_{C\text{-}F}$ = 69.9 Hz), 94.7, 55.7, 55.4, 50.6. **^{19}F{^{1}H} NMR** (377 MHz, $CDCl_3$) δ 22.4. **IR** (ATR) 3120 (w), 3011 (w), 2921 (br w), 2848 (w) 1769 (m), 1613 (m), 1537 (m), 1522 (m), 1504 (m), 1443 (m), 1396 (m), 1381 (m), 1290 (m), 1270 (m), 1247 (s), 1178 (m), 1059 (s), 1026 (m), 954 (s), 925 (m), 827(m), 809 (s), 780 (m), 734 (m), 736 (m), 704 (m). **HRMS-ESI** *(m/z)* $[M+Na]^+$ calculated for $C_{18}H_{16}FNO_3Na$, 336.1006; found, 336.1002. **mp**: 98.1 °C.

6-Methoxy-1-{[2-(trimethylsilyl)ethoxy]methyl}-1*H*-indole-3-carbonyl fluoride 2h

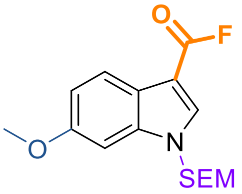

According to OP1, 6-methoxy-1-{[2-(trimethylsilyl)ethoxy]methyl}-1*H*-indole-3-carbonyl fluoride **2h** was obtained from 6-methoxy-1-{[2-(trimethylsilyl)ethoxy]methyl}-1*H*-indole **1h** (143 mg, 0.52 mmol, 1.0 equiv.), DNTFB (0.16 mL, 1.03 mmol, 2.0 equiv.) and DMAP (64.5 mg, 0.53 mmol, 1.0 equiv.). The crude material was purified by flash column chromatography (Pentane/Et_2O 85:15, R_f = 0.27) to give **2h** as a colourless oil (52.4 mg, 0.16 mmol, 32%). **^{1}H NMR** (400 MHz, $CDCl_3$) δ 7.95 (dd, $^3J_{H\text{-}H}$ = 9.5 Hz, $^4J_{H\text{-}H}$ = 1.7 Hz, 1H), 7.84 (s, 1H), 7.01 (s, 1H), 7.00 (dd, $^3J_{H\text{-}H}$ = 7.7 Hz, $^4J_{H\text{-}H}$ = 2.3 Hz, 1H), 5.48 (s, 2H), 3.88 (s, 3H), 3.56 – 3.47 (m, 2H), 0.96 – 0.86 (m, 2H), -0.04 (s, 9H). **^{13}C{^{1}H} NMR** (126 MHz, $CDCl_3$) δ 157.9, 154.7 (d, $^1J_{C\text{-}F}$ = 328 Hz), 137.8 (d, $^4J_{C\text{-}F}$ = 2.7 Hz), 136.0, 122.0, 121.0 (d, $^3J_{C\text{-}F}$ = 5.9 Hz), 113.3, 102.5 (d, $^2J_{C\text{-}F}$ = 69.9 Hz), 94.8, 76.8, 66.8, 55.8, 17.8, -1.3. **^{19}F{^{1}H} NMR** (377 MHz, $CDCl_3$) δ 23.1. **IR** (ATR) 2955 (br w), 1779 (s), 1621 (w), 1578 (w), 1538 (m), 1501 (m), 1449 (w), 1375 (w), 1234 (s), 1191 (w), 1166 (w), 1123 (w), 1074 (s), 1049 (s), 948 (m), 917 (m), 856 (m), 934 (s), 816 (s), 782 (m), 757 (m), 733 (m), 708 (m), 692 (m). **HRMS-ESI** *(m/z)* $[M+K]^+$ calculated for $C_{16}H_{22}FNO_3SiK$, 362.0985; found, 362.0991.

4-Methoxy-1-methyl-1*H*-indole-3-carbonyl fluoride 2l

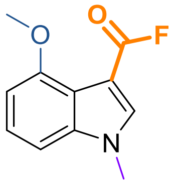

According to OP1, 4-methoxy-1-methyl-1*H*-indole-3-carbonyl fluoride **2l** was obtained from 4-methoxy-1-methyl-1*H*-indole **1l** (81.1 mg, 0.50 mmol, 1.0 equiv.), DNTFB (0.16 mL, 1.03 mmol, 2.0 equiv.) and DMAP (61.4 mg, 0.50 mmol, 1.0 equiv.) with a 2-hour reaction time. The crude material was purified by flash column chromatography (Pentane/Et_2O 70:30, R_f = 0.28) to give **2l** as a colourless solid (31.0 mg, 0.15 mmol, 55%). **^{1}H NMR** (400 MHz, $CDCl_3$) δ 7.78 (s, 1H), 7.28 (t, $^3J_{H-H}$ = 8.1 Hz, 1H), 6.97 (d, $^3J_{H-H}$ = 8.3 Hz, 1H), 6.75 (d, $^3J_{H-H}$ = 8.0 Hz, 1H), 3.97 (s, 3H), 3.80 (s, 3H). **^{13}C{^{1}H} NMR** (126 MHz, $CDCl_3$) δ 154.3, 153.5 (d, $^1J_{C-F}$ = 325 Hz), 139.6 (d, $^4J_{C-F}$ = 2.6 Hz), 138.4, 125.0, 116.1 (d, $^3J_{C-F}$ = 7.8 Hz), 103.5 (d, $^2J_{C-F}$ = 60.9 Hz), 101.3, 100.7, 55.9, 33.9. **^{19}F{^{1}H} NMR** (377 MHz, $CDCl_3$) δ 24.7. **IR** (ATR): 2921 (br w), 1793 (m), 1710 (w), 1532 (m), 1507 (m), 1452 (m), 1357 (m), 1270 (m), 907 (s), 730 (s). The product was too unstable to perform HRMS analysis and melting point measurement.

5-Methoxy-1-methyl-1*H*-indole-3-carbonyl fluoride 2m

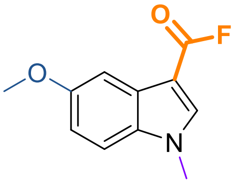

According to OP1, 5-methoxy-1-methyl-1*H*-indole-3-carbonyl fluoride **2m** was obtained from 5-methoxy-1-methyl-1*H*-indole **1m** (80.8 mg, 0.50 mmol, 1.0 equiv.), DNTFB (0.16 mL, 1.03 mmol, 2.0 equiv.) and DMAP (60.9 mg, 0.50 mmol, 1.0 equiv.). The crude material was purified by flash column chromatography (Pentane/Et_2O 80:20, R_f = 0.18) to give **2m** as a colourless solid (77.9 mg, 0.38 mmol, 75%). **^{1}H NMR** (400 MHz, $CDCl_3$) δ 7.78 (s, 1H), 7.53 (app. t, $^xJ_{H-H}$ = 2.1 Hz, 1H), 7.28 (d, $^3J_{H-H}$ = 8.9 Hz, 1H), 6.99 (dd, $^3J_{H-H}$ = 8.9 Hz, $^4J_{H-H}$ = 2.5 Hz, 1H), 3.90 (s, 3H), 3.86 (s, 3H). **^{13}C{^{1}H} NMR** (126 MHz, $CDCl_3$) δ 156.9, 154.8 (d, $^1J_{C-F}$ = 326 Hz), 137.6, 132.5 (d, $^4J_{C-F}$ = 2.9 Hz), 128.1 (d, $^3J_{C-F}$ = 6.8 Hz), 114.6, 111.4, 102.6, 100.7 (d, $^2J_{C-F}$ = 69.5 Hz), 56.0, 34.2. **^{19}F{^{1}H} NMR** (377 MHz, $CDCl_3$) δ 21.8. **IR** (ATR) 3136 (w), 3053 (w), 3004 (w), 2934 (br w), 1769 (s), 1621 (w), 1532 (s), 1477 (m), 1452 (m), 1424 (m), 1400 (w), 1369 (m), 1265 (m), 1234 (s), 1215 (s), 1182 (m), 1135 (m), 1080 (s), 1049 (m), 1019 (m), 954 (s), 868 (s), 853 (s), 807 (s), 757 (m), 733 (s), 708 (m). **HRMS-ESI** *(m/z)* $[M+Na]^+$ calculated for $C_{11}H_{10}FNO_2Na$, 230.0588; found, 230.0589. **mp**: 105.4 °C.

7-Methoxy-1-methyl-1*H*-indole-3-carbonyl fluoride 2n

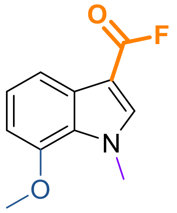

According to OP1, 7-methoxy-1-methyl-1*H*-indole-3-carbonyl fluoride **2n** was obtained from 7-methoxy-1-methyl-1*H*-indole **1n** (81.4 mg, 0.51 mmol, 1.0 equiv.), DNTFB (0.16 mL, 1.03 mmol,

2.0 equiv.) and DMAP (61.8 mg, 0.51 mmol, 1.0 equiv.). The crude material was purified by flash column chromatography (Toluene/Pentane 70:30, R_f = 0.31) to give **2n** as a yellowish solid (63.9 mg, 0.31 mmol, 61%). **^{1}H NMR** (400 MHz, $CDCl_3$) δ 7.69 (s, 1H), 7.66 (app. ddd, $^3J_{H\text{-}H}$ = 8.1 Hz, $^4J_{H\text{-}H}$ = 1.9 Hz, $^4J_{H\text{-}H}$ = 0.9 Hz, 1H), 7.21 (t, $^3J_{H\text{-}H}$ = 8.0 Hz, 1H), 6.74 (dd, $^3J_{H\text{-}H}$ = 7.9 Hz, $^4J_{H\text{-}H}$ = 0.9 Hz, 1H), 4.11 (s, 3H), 3.94 (s, 3H). **^{13}C{^{1}H} NMR** (126 MHz, $CDCl_3$) δ 154.8 (d, $^1J_{C\text{-}F}$ = 326 Hz), 148.2, 138.5, 129.6 (d, $^3J_{C\text{-}F}$ = 6.3 Hz), 127.3 (d, $^4J_{C\text{-}F}$ = 2.7 Hz*)*, 124.0, 113.7, 104.6, 101.0 (d, $^2J_{C\text{-}F}$ = 69.6 Hz), 55.7, 38.1. **^{19}F{^{1}H} NMR** (377 MHz, $CDCl_3$) δ 22.6. **IR** (ATR) 3127 (w), 2924 (br w), 285 (w), 1765 (s), 1656 (w), 1613 (w), 1580 (w), 1533 (m), 1501 (m), 1457 (m), 1396 (m), 1377 (m), 1328 (m), 1261 (m), 1236 (m), 1016 (m), 1066 (m), 964 (s), 870 (m), 783 (s), 725 (s). **HRMS-ESI** *(m/z)* $[M+K]^+$ calculated for $C_{11}H_{10}FKNO_2$, 246.0327; found, 230.00325. **mp**: 132.5 °C.

tert-Butyl (3-(fluorocarbonyl)-1-methyl-1*H*-indol-5-yl)carbamate 2o

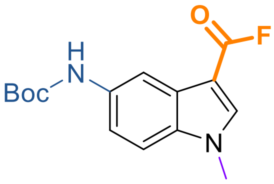

According to OP1, *tert*-butyl (3-(fluorocarbonyl)-1-methyl-1*H*-indol-5-yl)-carbamate **2n** was obtained from *tert*-butyl (1-methyl-1*H*-indol-5-yl)carbamate **1o** (123.1 mg, 0.50 mmol, 1.0 equiv.), DNTFB (0.16 mL, 1.03 mmol, 2.0 equiv.) and DMAP (61.2 mg, 0.50 mmol, 1.0 equiv.). The crude material was purified by flash column chromatography (PE/Et_2O 40:60, R_f = 0.30) to give a first pure fraction of **2o** as an off-white solid (59.3 mg). A second unpure light orange fraction was isolated as a solid (46.1 mg). It was purified by preparative TLC (PE/Et_2O 20:80, R_f = 0.60) to give pure **2n** as a light orange solid (31.4 mg). At the end, 90.7 mg of **2o** were isolated (0.31 mmol, 61%). **^{1}H NMR** (400 MHz, $CDCl_3$) δ 7.86 (br s, 1H), 7.80 (s, 1H), 7.62 (br s, 1H), 7.32 (d, $^3J_{H\text{-}H}$ = 8.9 Hz, 1H), 6.58 (s, 1H), 3.86 (s, 3H), 1.54 (s, 9H). **^{13}C{^{1}H} NMR** (126 MHz, $CDCl_3$) δ 154.5 (d, $^1J_{C\text{-}F}$ = 326. Hz), 156.2, 138.0, 134.4, 134.1 (d, $^4J_{C\text{-}F}$ = 2.5 Hz), 127.3 (d, $^3J_{C\text{-}F}$ = 5.9 Hz), 116.8, 110.8, 110.6, 100.6 (d, $^2J_{C\text{-}F}$ = 70.0 Hz), 80.5, 33.9, 28.4 (3C). **^{19}F{^{1}H} NMR** (377 MHz, $CDCl_3$) δ 22.4. **IR** (ATR) 3256 (br w), 3118 (w), 3060 (w), 2927 (w), 1781 (m), 1759 (s), 1694 (m), 1679 (m), 1587 (m), 1535 (s), 1489 (m), 1449 (m), 1391 (m), 1363 (m), 1311 (m), 1271 (m), 1249 (m), 1234 (m), 1209 (m), 1166 (s), 1077 (m), 1046 (s), 1026 (m), 979 (m), 908 (m), 877 (m), 859 (m), 797 (m), 782 (m), 751 (m), 732 (m). **HRMS-ESI** *(m/z)* $[M+Na]^+$ calculated for $C_{15}H_{17}FN_2NaO_3$, 315.1115; found, 315.1119. **mp**: 161.4 °C.

tert-Butyl *N*-{[3-(fluorocarbonyl)-1-methyl-*1H*-indol-4-yl]methyl}-carbamate 2p and 4-({[(*tert*-butoxy)carbonyl](fluorocarbonyl)amino}methyl)-1-methyl-*1H*-indole-3-carbonyl fluoride 2p'.

According to OP1, *tert*-butyl *N*-[(1-methyl-*1H*-indol-4-yl)methyl]-carbamate **1p** (131 mg, 0.50 mmol, 1.0 equiv.), DNTFB (0.16 mL, 1.03 mmol, 2.0 equiv.) and DMAP (61.2 mg, 0.50 mmol, 1.0 equiv.) were mixed in MeCN (1.5 mL) for 1 h at 80 °C. The crude material was purified by flash column chromatography (*c*-hexane/EtOAc 70:30, R_f = 0.33) to give 100 mg (corresponding to a 60% combined yield of a mixture of *tert*-butyl *N*-{[3-(fluorocarbonyl)-1-methyl-*1H*-indol-4-yl]methyl}carbamate **2p** (38.2w%, determined by ^{1}H NMR) and 4-({[(*tert*-butoxy)carbonyl](fluorocarbonyl)amino}methyl)-1-methyl-*1H*-indole-3-carbonyl fluoride **2p'** (61.8w%, determined by ^{1}H NMR) as a dark red sticky oil.

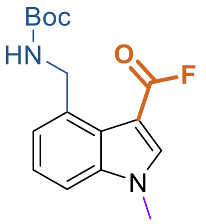

2p (38.2w%, 38.2 mg, 0.12 mmol, 25%). **^{1}H NMR** (400 MHz, $CDCl_3$) δ 7.95 (s, 1H), 7.47 – 7.43 (m, 1H), 7.41 – 7.29 (d, $^3J_{H\text{-}H}$ = 6.4 Hz, 2H), 5.62 (s, 1H), 4.76 (d, $^3J_{H\text{-}H}$ = 6.7 Hz, 2H), 3.88 (s, 3H), 1.40 (s, 9H). **^{13}C{^{1}H} NMR** (126 MHz, $CDCl_3$) δ 155.9, 155.2 (d, $^1J_{C\text{-}F}$ = 324 Hz), 139.9, 138.7 (d, $^4J_{C\text{-}F}$ = 3.5 Hz), 133.1, 125.7, 124.6 (d, $^3J_{C\text{-}F}$ = 10.7 Hz), 124.3, 109.9, 100.7 (d, $^2J_{C\text{-}F}$ = 67.6 Hz), 78.9, 43.8, 28.4. **^{19}F{^{1}H} NMR** (377 MHz, $CDCl_3$) δ 20.3. **HRMS-ESI** *(m/z)* $[M+Na]^+$ calculated for $C_{16}H_{19}FN_2NaO_3$, 329.1272; found, 329.1282.

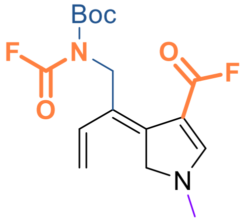

2p' (61.8w%, 61.8 mg, 0.18 mmol, 35%). **^{1}H NMR** (400 MHz, $CDCl_3$) δ 7.95 (s, 1H), 7.41 – 7.29 (m, 2H), 7.09 (dd, $^3J_{H\text{-}H}$ = 7.1 Hz, $^4J_{H\text{-}H}$ = 1.3 Hz, 1H), 5.68 (s, 2H), 3.88 (s, 3H), 1.42 (s, 9H). **^{13}C{^{1}H} NMR** (126 MHz, $CDCl_3$) δ 154.62 (d, $^1J_{C\text{-}F}$ = 323 Hz, C-COF), 150.4, 144.7 (d, $^1J_{C\text{-}F}$ = 304 Hz, N-COF), 139.9, 138.5 (d, $^4J_{C\text{-}F}$ = 3.2 Hz), 130.6, 124.4, 123.6 (d, $^3J_{C\text{-}F}$ = 10.5 Hz), 118.8, 109.7, 101.2 (d, $^2J_{C\text{-}F}$ = 69.5 Hz), 84.9, 49.3 (d, $^4J_{C\text{-}F}$ = 3.8 Hz), 34.0, 27.7. **^{19}F{^{1}H} NMR** δ 20.3 (s, 1F), -1.5 (s, 1F). **HRMS-ESI** *(m/z)* $[M+Na]^+$ calculated for $C_{17}H_{18}F_2N_2NaO_4$, 375.1127; found, 375.1135.

5-Bromo-1-methyl-1*H*-indole-3-carbonyl fluoride 2q

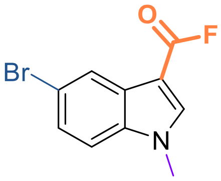

According to OP1, 5-bromo-1-methyl-1*H*-indole-3-carbonyl fluoride **2q** was obtained from 5-bromo-1-methyl-1*H*-indole **1q** (106 mg, 0.51 mmol, 1.0 equiv.), DNTFB (0.16 mL, 1.03 mmol, 2.0 equiv.) and DMAP (63.5 mg, 0.52 mmol, 1.0 equiv.) with a 2-hour reaction time. The crude material was purified by flash column chromatography (Petroleum ether/Et_2O 50:50, R_f = 0.24) to give **2q** as a light orange solid (99.8 mg, 0.39 mmol, 77%). Also synthetized on 1.0 g scale with DNTFB (4.44 mL, 25.6 mmol, 6.0 equiv.), DMAP (1.75 g, 14.3 mmol, 3.0 equiv.) and MeCN (14 mL) with a 2-hour reaction time, giving **2p** as a pink to orange solid (987 mg, 3.85 mmol, 80%). **^{1}H NMR** (400 MHz, $CDCl_3$) δ 8.23 (app. t, $^4J_{H\text{-}H}$ = 1.8 Hz, 1H), 7.82 (s, 1H), 7.46 (dd, $^3J_{H\text{-}H}$ = 8.7 Hz, $^4J_{H\text{-}H}$ = 1.9 Hz, 1H), 7.26 (d, $^3J_{H\text{-}H}$ = 8.7 Hz, 1H), 3.88 (s, 3H).**^{13}C{^{1}H} NMR** (126 MHz, $CDCl_3$) δ 154.2 (d, $^1J_{C\text{-}F}$ = 327.1 Hz), 138.5, 136.3, 128.6 (d, $^3J_{C\text{-}F}$ = 6.1 Hz), 127.1, 124.0 (d, $^4J_{C\text{-}F}$ = 1.6 Hz), 117.1, 111.9, 101.0 (d, $^2J_{C\text{-}F}$ = 70.9 Hz), 34.2. **^{19}F{^{1}H} NMR** (377 MHz, $CDCl_3$) δ 23.2. **IR** (ATR) 3090 (w), 2924 (w), 1778 (s), 1606 (w), 1529 (m), 1446 (m), 1384 (m), 1360 (m), 1301 (w), 1246 (m), 1209 (m), 1147 (m), 1092 (m), 1043 (m), 959 (m), 936 (m), 874 (m), 840 (m), 800 (s), 773 (m), 754 (m), 727 (m). **HRMS-ESI** *(m/z)* $[M+K]^+$ calculated for $C_{10}H_7ON^{79}BrFKN$, 277.9587; found, 277.9587. **mp**: 150.1 °C.

5-Formyl-1-methyl-1*H*-indole-3-carbonyl fluoride 2r

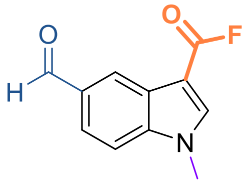

According to OP1, 5-formyl-1-methyl-1*H*-indole-3-carbonyl fluoride **2r** was obtained from 5-formyl-1-methyl-1*H*-indole **1r** (81.8 mg, 0.51 mmol, 1.0 equiv.), DNTFB (0.47 mL, 3.03 mmol, 5.9 equiv.) and DMAP (186 mg, 1.52 mmol, 3.0 equiv.) with a 2-hour reaction time. The crude material was purified by flash column chromatography (Pentane/Et_2O 50:50, R_f = 0.13) to give **2r** as an orange solid (19.8 mg, 0.10 mmol, 19%). **^{1}H NMR** (400 MHz, $CDCl_3$) δ 10.14 (d, $^4J_{H\text{-}H}$ = 0.6 Hz, 1H), 8.61 (m, 1H), 7.97 (dd, $^3J_{H\text{-}H}$ = 8.6 Hz, $^4J_{H\text{-}H}$ = 1.6 Hz, 2H), 7.97 (s, 1H), 7.53 (d, $^3J_{H\text{-}H}$ = 8.6 Hz, 1H), 3.96 (s, 3H). **^{13}C{^{1}H} NMR** (126 MHz, $CDCl_3$) δ 192.2, 154.1 (d, $^1J_{C\text{-}F}$ = 328 Hz), 140.7 (d, $^4J_{C\text{-}F}$ = 2.8 Hz), 139.4, 132.3, 126. (d, $^3J_{C\text{-}F}$ = 6.7 Hz), 126.6, 123.5, 111.3, 103.1 (d, $^2J_{C\text{-}F}$ = 71.0 Hz), 34.3. **^{19}F{^{1}H} NMR** (377 MHz, $CDCl_3$) δ 24.2. **IR** (ATR) 3127 (w), 2917 (w), 2826 (w), 2732 (w), 1776 (s), 1682 (s), 1613 (m), 1569 (w), 1530 (m), 1457 (m), 1392 (w), 1367 (s), 1316 (w), 1265 (w), 1214 (m), 1193 (m), 1124 (m), 1069 (s), 1041 (m), 950 (m), 874 (m), 812 (m), 754 (m), 736 (s). **HRMS-ESI** *(m/z)* $[M+Na]^+$ calculated for $C_{11}H_8FNO_2Na$, 228.0431; found, 228.0431. **mp**: 156.0 °C.

1-Methyl-6-(trifluoromethyl)-1*H*-indole-3-carbonyl fluoride 2s

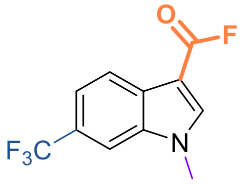

According to OP1, 1-methyl-6-(trifluoromethyl)-1*H*-indole-3-carbonyl fluoride **2s** was obtained from 1-methyl-6-(trifluoromethyl)-1*H*-indole **1s** (99.2 mg, 0.50 mmol, 1.0 equiv.), DNTFB (0.47 mL, 3.03 mmol, 6.1 equiv.) and DMAP (187 mg, 1.53 mmol, 3.0 equiv.) with a 2-hour reaction time. The crude material was purified by flash column chromatography (PE/Et_2O 60:40, R_f = 0.36) to give **2s** as an orange solid (68.9 mg, 0.28 mmol, 56%). **^{1}H NMR** (400 MHz, $CDCl_3$) δ 8.20 (d, $^3J_{H\text{-}H}$ = 8.4 Hz, 1H), 7.99 (s, 1H), 7.70 (s, 1H), 7.60 (dd, $^3J_{H\text{-}H}$ = 8.4 Hz, $^4J_{H\text{-}H}$ = 0.9 Hz, 1H), 3.96 (s, 3H). **^{13}C{^{1}H} NMR** (126 MHz, $CDCl_3$) δ 154.0 (d, $^1J_{C\text{-}F}$ = 328 Hz), 139.6, 136.6 (d, $^4J_{C\text{-}F}$ = 2.9 Hz), 129.3 (d, $^3J_{C\text{-}F}$ = 6.4 Hz), 126.2 (q, $^2J_{C\text{-}F}$ = 32.5 Hz), 124.6 (q, $^1J_{C\text{-}F}$ = 272 Hz), 121.8, 119.7 (q, $^3J_{C\text{-}F}$ = 3.6 Hz), 108.0 (q, $^3J_{C\text{-}F}$ = 4.3 Hz), 101.6 (d, $^2J_{C\text{-}F}$ = 71.2 Hz), 34.1. **^{19}F{^{1}H} NMR** (377 MHz, $CDCl_3$) δ 23.9 (br s, 1F), -61.1 (s, 3F). **IR** (ATR) 3142 (w), 3076 (w), 2924 (w), 1772 (s), 1623 (w), 1533 (m), 1457 (m), 1403 (m), 1385 (m), 1330 (s), 1290 (m), 1211 (s), 1171 (m), 1109 (s), 1084 (m), 1066 (m), 1037 (m), 943 (s), 881 (m), 827 (s), 768,8 (m), 736 (m). **HRMS-ESI** not found. **LCMS** *(m/z)* = $[M]^{\bullet +}$ 245.11 (100.0%), $[M - F]^+$ 226.09 (43.4%), $[M - CF_3]^+$ 176.04 (10.0%). **mp**: 120.2 °C.

Methyl 3-(fluorocarbonyl)-1-methyl-1*H*-indole-7-carboxylate 2t

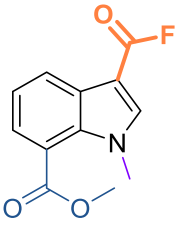

According to OP1, methyl 3-(fluorocarbonyl)-1-methyl-1*H*-indole-7-carboxylate **2s** was obtained from methyl 1-methyl-1*H*-indole-7-carboxylate **1t** (114 mg, 0.60 mmol, 1.0 equiv.), DNTFB (0.56 mL, 3.61 mmol, 6.0 equiv.) and DMAP (220 mg, 1.80 mmol, 3.0 equiv.) with a 2-hour reaction time. The crude material was purified by flash column chromatography (PE/Et_2O 70:30, R_f = 0.27) to give **2t** as a light orange solid (79.6 mg, 0.34 mmol, 56%). **^{1}H NMR** (400 MHz, $CDCl_3$) δ 8.31 (dt, $^3J_{H\text{-}H}$ = 8.0 Hz, $^4J_{H\text{-}H}$ = 1.5 Hz, 1H), 7.86 (s, 1H), 7.78 (dd, $^3J_{H\text{-}H}$ = 7.5 Hz, $^4J_{H\text{-}H}$ = 1.2 Hz, 1H), 7.35 (dd, $^3J_{H\text{-}H}$ = 8.0 Hz, $^3J_{H\text{-}H}$ = 7.6 Hz, 1H), 3.99 (s, 6H).**^{13}C{^{1}H} NMR** (126 MHz, $CDCl_3$) δ 167.3, 154.2 (d, $^1J_{C\text{-}F}$ = 328 Hz), 140.7, 134.8 (d, $^4J_{C\text{-}F}$ = 3.0 Hz), 129.3 (d, $^3J_{C\text{-}F}$ = 6.9 Hz), 127.0, 125.5, 122.4, 117.8, 101.6 (d, $^2J_{C\text{-}F}$ = 70.1 Hz), 52.7, 38.9. **^{19}F{^{1}H} NMR** (377 MHz, $CDCl_3$) δ 23.9. **IR** (ATR) 3128 (w), 2956 (w), 1777 (m), 1712 (m), 1540 (m), 1448 (m), 1433 (m), 1411 (m), 1377 (m), 1267 (s), 1218 (s), 1187 (m), 1144 (m), 1092 (s), 1052 (m), 1009 (m), 941 (s), 926 (m), 868 (m), 819 (m), 782 (m), 754 (s), 736 (s). **HRMS-ESI** *(m/z)* $[M]^+$ calculated for $C_{12}H_{10}FNO_3$, 274.0276; found, 274.0273. **mp**: 158.7 °C.

Methyl 3-(fluorocarbonyl)-1*H*-indole-7-carboxylate 2v and methyl 1-(fluorocarbonyl)-1*H*-indole-7-carboxylate 2v'

According to OP1, methyl 1*H*-indole-7-carboxylate **1v** (86.1 mg, 0.49 mmol, 1.0 equiv.) was mixed with DNTFB (0.47 mL, 3.03 mmol, 6.2 equiv.) and DMAP (181 mg, 1.48 mmol, 3.0 equiv.) in MeCN (1.5 mL) for 2 h at 80 °C.

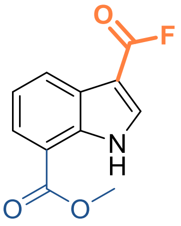

2v was obtained after purification of the reaction crude by flash column chromatography (pentane/Et_2O 80:20, R_f = 0.22) as a light-yellow solid (36.7 mg, 0.17, 34%). **^{1}H NMR** (400 MHz, $CDCl_3$) δ 10.52 (br s, 1H), 8.34 (d, $^3J_{H\text{-}H}$ = 8.0 Hz, 1H), 8.09 (d, $^3J_{H\text{-}H}$ = 3.1 Hz, 1H), 8.00 (dd, $^3J_{H\text{-}H}$ = 7.6 Hz, $^4J_{H\text{-}H}$ = 1.1 Hz, 1H), 7.40 (t, $^3J_{H\text{-}H}$ = 7.8 Hz, 1H), 4.02 (s, 3H). **^{13}C{^{1}H} NMR** (126 MHz, $CDCl_3$) δ 167.3, 154.4 (d, $^1J_{C\text{-}F}$ = 329 Hz), 136.2 (d, $^4J_{C\text{-}F}$ = 3.0 Hz), 134.6, 126.9 (d, $^3J_{C\text{-}F}$ = 6.4 Hz), 126.6, 126.2, 122.3, 113.9, 103.3 (d, $^2J_{C\text{-}F}$ = 70.6 Hz), 52.3. **^{19}F{^{1}H} NMR** (377 MHz, $CDCl_3$) δ 24.3. **IR** (ATR): 3337 (br m), 2961 (w), 2921 (w), 2847 (w), 1796 (s), 1692 (m), 1590 (w), 1532 (m), 1440 (m), 1418 (w), 1375 (w), 1351 (w), 1317 (w), 1286 (s), 1234 (w), 1212 (m), 1185 (w), 1135 (m), 1108 (m), 1071 (w), 1028 (w), 973 (w), 948 (m), 902 w), 853 (w), 757 (m), 733 (m), 628 (w). **HRMS-ESI** *(m/z)* $[M]^+$ calculated for $C_{11}H_9FNO_3$, 222.0561; found, 222.0571. **mp**: 170.6 °C.

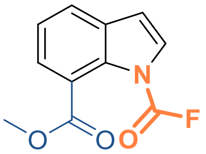 **2v′** was not isolated by flash column chromatography. It was only observed in quantitative ^{1}H NMR with a 37% yield and in the crude mixture after work-up. Some NMR signals could be attributed to **2v′**. **^{1}H NMR** (400 MHz, $CDCl_3$) δ 6.77 (d, $^{3}J_{H\text{-}H}$ = 3.8 Hz, 1H), 3.94 (s, 3H). **^{19}F{^{1}H} NMR** (377 MHz, $CDCl_3$) δ -4.17.

2-*tert*-Butyl-1-methyl-1*H*-indole-3-carbonyl fluoride 2w

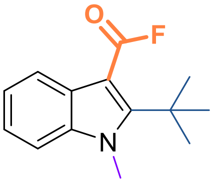 According to OP1, 2-*tert*-butyl-1-methyl-1*H*-indole-3-carbonyl fluoride **2w** was obtained from 2-*tert*-butyl-1-methyl-1*H*-indole **1w** (94.0 mg, 50 mmol, 1.0 equiv.), DNTFB (0.47 mL, 3.03 mmol, 6.0 equiv.) and DMAP (183 mg, 1.50 mmol, 3.0 equiv.) with a 2-hour reaction time. The crude material was purified by flash column chromatography (PE/Et_2O 90:10, R_f = 0.34) to give **2w** as a brown oil (46.6 mg, 0.20 mmol, 40%). **^{1}H NMR** (400 MHz, $CDCl_3$) δ 7.93 – 7.84 (m, 1H), 7.35 – 7.20 (m, 3H), 3.97 (s, 3H), 1.66 (s, 9H). **^{13}C{^{1}H} NMR** (126 MHz, $CDCl_3$) δ 157.2 (d, $^{3}J_{C\text{-}F}$ = 13.6 Hz), 156.3 (d, $^{1}J_{C\text{-}F}$ = 328 Hz), 137.2, 127.4 (d, $^{4}J_{C\text{-}F}$ = 3.9 Hz), 123.2, 122.8, 121.1 (d, $^{3}J_{C\text{-}F}$ = 10.5 Hz), 109.9, 100.4 (d, $^{2}J_{C\text{-}F}$ = 70.5 Hz), 35.5, 34.7, 30.9. **^{19}F{^{1}H} NMR** (377 MHz, $CDCl_3$) δ 43.8. The product was too unstable to perform HRMS analyses. GC-MS analyses only gave the mass of the substrate **1w.** We suspect an hydrolysis of acyl fluoride **2w** followed by a decarboxylation reaction leading to the starting indole **1w**.

3-Methyl-1*H*-indole-1-carbonyl fluoride 2x

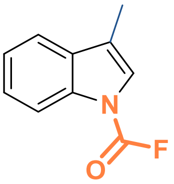 According to OP4, 2-*tert*-butyl-1-methyl-1*H*-indole-3-carbonyl fluoride **2x** was obtained from 2-*tert*-butyl-1-methyl-1*H*-indole **1x** (66.6 mg, 0.51 mmol, 1.0 equiv.), DNTFB (0.47 mL, 3.03 mmol, 6.2 equiv.) and DMAP (181 mg, 1.48 mmol, 3.0 equiv.) with a 2-hour reaction time. The crude material was purified by flash column chromatography (pentane/DCM 90:10, R_f = 0.44) to give **2x** as a yellowish solid (62.0 mg, 0.35 mmol, 69%). **^{1}H NMR** (400 MHz, $CDCl_3$) δ 8.15 (d, $^{3}J_{H\text{-}H}$ = 8.0 Hz, 0.75H), 7.74 (br s, 0.25H) (rotamers signals), 7.54 (dd, $^{3}J_{H\text{-}H}$ = 7.5, $^{4}J_{H\text{-}H}$ = 1.3 Hz, 1H), 7.38 (m, 2.25H), 7.16 (s, 0.75H) (rotamers signals), 2.29 (d, $^{4}J_{H\text{-}H}$ = 1.4 Hz, 3H). **^{13}C{^{1}H} NMR** (126 MHz, $CDCl_3$) δ 140.2 (d, $^{1}J_{C\text{-}F}$ = 301 Hz), 135.6, 131.9, 125.6, 124.4, 121.0, 120.2, 119.5, 114.7, 9.6. **^{19}F{^{1}H} NMR** (377 MHz, $CDCl_3$) δ -5.4 (br s, 0.78F), -7.9 (br s, 0.22F) (rotamers signals). **IR** (ATR): 3133 (w), 3060 (w), 2930 (w), 2847 (w), 1809 (s), 1618 (w), 1758 (w), 1458 (m), 1394 (s), 1375 (m), 1348 (m), 1234 (m), 1172 (w), 1148 (w), 1126 (w), 1071 (m), 1922 (w), 960 (m), 911 (w), 785 (w), 742 (s), 696 (m), 650 (w), 619 (w), 555 (w). **HRMS-ESI *(m/z)*** $[M]^+$ calculated for $C_{10}H_8FNO$, 177.0584; found, 177.0580. **mp**: 38.7 °C.

1-Methyl-4-(trifluoromethoxy)-1*H*-indole-3-carbonyl fluoride 2z[4] and 1-methyl-5-(trifluoromethoxy)-1*H*-indole-3-carbonyl fluoride 2z[5]

In a 10 mL screw-cap tube closed by a septum, DNTFB (1.40 mL, 9.01 mmol, 19.7 equiv.) was added to a solution of DMAP (555 mg, 4.54 mmol, 9.9 equiv.) in anhydrous MeCN (4 mL) under argon. The septum was quickly replaced by a screw-cap and the resulting solution was stirred for 20 min at room temperature. Then, a solution of 1-methyl-4-(trimethylsilyl)-1*H*-indol-5-yl trifluoromethanesulfonate **1z** (161 mg, 0.46 mmol, 1.0 equiv.) in anhydrous MeCN (1 mL) was quickly added in the tube and the latter was tightly closed. The reaction mixture was stirred overnight at 80 °C. The tube was allowed to cool down to room temperature, then 10 mL of distilled water were added to the reaction medium in a separating funnel and the aqueous layer was extracted with pentane (3 x 10 mL). The combined organic layers were washed with brine (3 x 10 mL), dried over $MgSO_4$ and concentrated under vacuum. The product was purified by flash column chromatography.

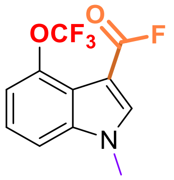

1-Methyl-4-(trifluoromethoxy)-1*H*-indole-3-carbonyl fluoride **2z[4]** was detected by quantitative ^{19}F NMR analysis (with α,α,α-trifluorotoluene as internal standard). Yield = 7%. **$^{19}F\{^1H\}$ NMR** (376 MHz, $CDCl_3$) δ 21.9 (br s, 1F), -58.0 (3F).

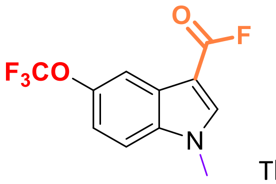

The purification by flash column chromatography (Pentane/Et_2O 80:20, R_f = 0.50) gave 1-methyl-5-(trifluoromethoxy)-1*H*-indole-3-carbonyl fluoride **2z[5]** as a yellow-orange solid (41.3 mg, 0.16 mmol, 34%). **^1H NMR** (400 MHz, $CDCl_3$) δ 7.93 (s, 1H), 7.87 (s, 1H), 7.39 (d, $^3J_{H-H}$ = 8.9 Hz, 1H), 7.23 (dd, $^3J_{H-H}$ = 8.9 Hz, $^4J_{H-H}$ = 2.3 Hz, 1H), 3.90 (s, 3H). **$^{13}C\{^1H\}$ NMR** (101 MHz, $CDCl_3$) δ 154.0 (d, $^1J_{C-F}$ = 327 Hz), 145.5, 139.1, 135.7 (d, $^4J_{C-F}$ = 2.6 Hz), 127.3 (d, $^3J_{C-F}$ = 6.4 Hz), 120.6 (q, $^1J_{C-F}$ = 257 Hz), 117.9, 113.8, 111.5, 101.4 (d, J_{C-F} = 71.1 Hz), 34.1. **$^{19}F\{^1H\}$ NMR** (377 MHz, $CDCl_3$) δ 23.1 (br s, 1F), -58.1 (s, 3F). **IR** (ATR): 3136 (w), 2955 (w), 1780 (s), 1621 (w), 1532 (m), 1480 (w), 1461 (w), 1372 (w), 1298 (m), 1258 (m), 1212 (s), 1151 (s), 1139 (s), 1080 (m), 1049 (w), 976 (w), 917 (w). **HRMS-ESI** *(m/z)* $[M]^+$ calculated for $C_{11}H_7O_2NF_4Na$, 284.0305; found, 284.0302. **mp**: 100.5 °C.

5-Methoxythiophene-2-carbonyl fluoride 2aa

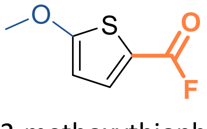

According to OP1, 5-methoxythiophene-2-carbonyl fluoride **2aa** was obtained from 2-methoxythiophene **1aa** (50 µL, 0.50 mmol, 1.0 equiv.), DNTFB (0.47 mL, 3.03 mmol, 6.1 equiv.) and DMAP (182.8 mg, 1.20 mmol, 3.0 equiv.) with a 2-hour reaction time. The crude material was purified by flash column chromatography (PE/Et_2O 90:10, R_f = 0.41) to give **2aa** as a yellow oil (43.1 mg, 0.27 mmol, 54%). **^1H NMR** (400 MHz, $CDCl_3$) δ 7.70 (d, $^3J_{H-H}$ = 4.4 Hz, 1H), 6.33 (dd, $^3J_{H-H}$ = 4.4 Hz, $^4J_{H-F}$ = 1.8 Hz, 1H), 4.01 (s, 3H). **$^{13}C\{^1H\}$ NMR** (126 MHz, $CDCl_3$) δ 176.2 (d, $^4J_{C-F}$ = 1.9 Hz), 153.0 (d, $^1J_{C-F}$ = 327.0 Hz), 138.5 (d, $^3J_{C-F}$ = 2.8 Hz), 111.8 (d, $^2J_{C-F}$ = 78.5 Hz), 107.1, 60.9. **$^{19}F\{^1H\}$ NMR** (377 MHz, $CDCl_3$) δ 18.1. **IR** (ATR) 3112 (w), 2934 (br w), 1779 (s), 1538 (m), 1474 (s), 1412 (s), 1357 (m), 1274 (m), 1240 (m), 1216 (m), 1052 (m), 963 (m), 794 (m), 727 (m). **HRMS-ESI** not found. **LCMS** *(m/z)* = $[M]^{\bullet+}$

160.00 (90.5%), $[M - Me]^+$ 144.96 (17.3%), $[M - Me - CO]^+$ 116.99 (100.0%), $[M - Me - COF]^{\bullet+}$ 97.98 (7.6%).

Methyl 1-methyl-1*H*-imidazole-2-carboxylate 2ab (CAS Number: 62366-53-4)

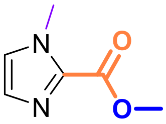

According to OP1, methyl 1-methyl-1*H*-imidazole-2-carboxylate **2ab** was obtained from 1-methylimidazole **1ab** (40 µL, 0.50 mmol, 1.0 equiv.), DNTFB (0.16 mL, 1.03 mmol, 2.1 equiv.) and DMAP (62.5 mg, 0.51 mmol, 1.0 equiv.). After 1 hour, the reaction mixture was concentrated under reduced pressure to dryness. Then MeOH (5 mL) was added and the solution was stirred at room temperature for 30 min. Standard workup led to the crude material that was purified by flash column chromatography (DCM/MeOH 98:2, Rf = 0.34) to give **2ab** as a yellow oil (53.2 mg, 0.39 mmol, 74%). **^{1}H NMR** (400 MHz, $CDCl_3$) δ 7.14 (d, $^3J_{H\text{-}H}$ = 1.0 Hz, 1H), 7.03 (d, $^3J_{H\text{-}H}$ = 1.0 Hz, 1H), 4.01 (s, 3H), 3.93 (s, 3H). **^{13}C{^{1}H} NMR** (126 MHz, $CDCl_3$) δ 159.8, 136.7, 129.6, 126.4, 52.3, 35.9. **IR** (ATR) 3106 (w), 2956 (w), 2222 (w), 1712 (s), 1473 (m), 1442 (m), 1399 (m), 1291 (m), 1261 (s), 1190 (m), 1126 (s), 1055 (m), 963 (w), 917 (m), 815 (m), 779 (m), 763 (m), 729 (m). **HRMS-ESI** *(m/z)* $[M]^+$ calculated for $C_6H_8N_2NaO_2$, 163.0478; found, 163.0468.

In accordance with the literature.[30]

Methyl 1-benzyl-1*H*-imidazole-2-carboxylate 2ac (CAS Number: 1502811-19-9)

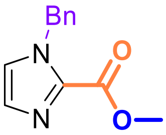

According to OP1, methyl 1-benzyl-1*H*-imidazole-2-carboxylate **2ac** was obtained from 1-benzylimidazole **1ac** (79.2 mg, 0.50 mmol, 1.0 equiv.), DNTFB (0.16 mL, 1.03 mmol, 2.1 equiv.) and DMAP (62.3 mg, 0.51 mmol, 1.0 equiv.). After 1 hour, the reaction mixture was concentrated under reduced pressure to dryness. Then MeOH (5 mL) was added and the solution was stirred at room temperature for 30 min. Standard workup led to the crude material that was purified by flash column chromatography (DCM/MeOH 98:2, Rf = 0.25) to give **2ac** as an orange solid (95.5 mg, 0.44 mmol, 88%). **^{1}H NMR** (400 MHz, $CDCl_3$) δ 7.36 – 7.27 (m, 3H), 7.19 – 7.14 (m, 3H), 7.06 (d, $^3J_{H\text{-}H}$ = 1.1 Hz, 1H), 5.62 (s, 2H), 3.91 (s, 3H). **^{13}C{^{1}H} NMR** (126 MHz, $CDCl_3$) δ 159.7, 136.4, 136.3, 130.0, 129.1 (2C), 128.3, 127.6 (2C), 125.5, 52.4, 51.8. **IR** (ATR) 3134 (w), 2953 (w), 1700 (s), 1493 (m), 1450 (m), 1435 (s), 1410 (m), 1385 (m), 1301 (m), 1258 (s), 1200 (m), 1153 (m), 1109 (s), 1066 (m), 917 (m), 780 (m), 758 (m), 722 (s), 696 (m). **HRMS-ESI** *(m/z)* $[M]^+$ calculated for $C_{12}H_{13}N_2O_2$, 217.0972; found, 217.0984. **mp**: 70.8 °C.

1-Methyl-1*H*-pyrrole-2-carbonyl fluoride 2ad (CAS Number: 38144-27-3)

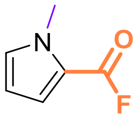

According to OP1, 1-methyl-1*H*-pyrrole-2-carbonyl fluoride **2ad** was obtained from *N*-methylpyrrole **1ad** (44 µL, 0.50 mmol, 1.0 equiv.), DNTFB (0.16 mL, 1.03 mmol, 2.1 equiv.) and DMAP

(60.7 mg, 0.50 mmol, 1.0 equiv.) with a 2-hour reaction time. The crude material was purified by flash column chromatography (Pentane/Et_2O 90:10, R_f = 0.67, revealed with vanillin) to give **2ad** as a red oil (11.2 mg, 0.09 mmol, 18%). **^{1}H NMR** (400 MHz, $CDCl_3$) δ 7.10 (ddd, $^3J_{H-H}$ = 4.2 Hz, $^4J_{H-H}$ = 1.8 Hz, $^4J_{H-F}$ = 0.8 Hz, 1H), 6.98 (dd, $^3J_{H-H}$ = 2.5 Hz, 1H), 6.21 (dd, $^3J_{H-H}$ = 4.2 Hz, $^3J_{H-H}$ = 2.5 Hz, 1H), 3.93 (d, $^{TS}J_{H-F}$ = 1.0 Hz, 3H). **^{13}C{^{1}H} NMR** (126 MHz, $CDCl_3$) δ 151.2 (d, $^1J_{C-F}$ = 323 Hz), 132.9 (d, $^3J_{C-F}$ = 5.6 Hz), 122.3 (d, $^4J_{C-F}$ = 3.2 Hz), 109.3, 36.6. The signal of the carbon bearing the COF group could not be detected. **^{19}F{^{1}H} NMR** (377 MHz, $CDCl_3$) δ 17.7. **IR** (ATR) 2958 (w), 2930 (w), 2857 (w), 2094 (w), 1781 (s), 1707 (w), 1529 (s), 1468 (w), 1406 (m), 1387 (m), 1326 (w), 1246 (w), 1218 (w), 1096 (w), 1062 (w), 1043 (w), 988 (m), 883 (w), 736 (m). **HRMS-ESI** not found. **LCMS** *(m/z)* = $[M]^{\bullet+}$ 127.06 (100.0%), $[M - F]^+$ 108.06 (8.8%), $[M - CO]^+$ 99.06 (37.9%).

In accordance with the literature.[31]

Methyl 1-methyl-1*H*-pyrrole-2-carboxylate 2ad' (CAS Number 37619-24-2)

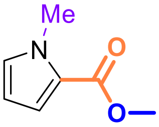

According to OP1, methyl 1-methyl-1H-pyrrole-2-carboxylate **2ad'** was obtained from *N*-methylpyrrole **1ad** (44 µL, 0.50 mmol, 1.0 equiv.), DNTFB (0.16 mL, 1.03 mmol, 2.1 equiv.) and DMAP (61.3 mg, 0.50 mmol, 1.0 equiv.). After 2 hours, the reaction mixture was concentrated under reduced pressure to dryness. Then MeOH (5 mL) and NEt_3 (70 µL, 0.50 mmol, 1.0 equiv.) was added and the solution was stirred at room temperature for 16 hours. Standard workup led to the crude material that was purified by flash column chromatography (pentane/Et_2O 70:30 to 60:40, R_f = 0.83 in pentane/Et_2O 70:30) to give **2ad'** as an orange oil (23.1 mg, 0.17 mmol, 33%). **^{1}H NMR** (400 MHz, $CDCl_3$) δ 6.93 (dd, $^3J_{H-H}$ = 3.9 Hz, $^4J_{H-H}$ = 1.8 Hz, 1H), 6.78 (t, $^3J_{H-H}$ = 2.2 Hz, 1H), 6.11 (dd, $^3J_{H-H}$ = 4.0 Hz, $^3J_{H-H}$ = 1.5 Hz, 1H), 3.93 (s, 3H), 3.81 (s, 3H). **^{13}C{^{1}H} NMR** (126 MHz, $CDCl_3$) δ 161.9, 129.6, 122.5, 117.9, 108.0, 51.1, 36.9. **IR** (ATR): 3115 (w), 2952 (w), 1704 (s), 1529 (m), 1477 (w), 1437 (m), 1412 (m), 1323 (m), 1246 (s), 1188 (w), 1114 (s), 1052 (m), 1022 (w), 954 (w), 890 (w), 803 (w), 739 (m), 690 (w), 607 (w), 505 (w). **HRMS-ESI** *(m/z)* $[M+H]^+$ calculated for $C_9H_8FN_2O$, 140.0706; found, 140.0703.

Data in accordance with the literature.[32]

1-Benzyl-1*H*-pyrrole-2-carbonyl fluoride 2ae

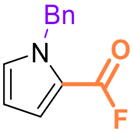

According to OP1, 1-benzyl-1*H*-pyrrole-2-carbonyl fluoride **2ae** was obtained from *N*-benzylpyrrole **1ae** (79.1 mg, 0.50 mmol, 1.0 equiv.), DNTFB (0.47 mL, 3.03 mmol, 6.0 equiv.) and DMAP (185 mg, 1.52 mmol, 3.0 equiv.) with a 2-hour reaction time. The crude material was purified by flash column chromatography (PE/Et_2O 95:5, R_f = 0.48) to give **2ae** in mixture with 4-methoxytrifluorotoluene (internal standard for quantitative NMR) as a yellow oil. This mixture was purified by preparative TLC with PE/DCM 80:20 as eluting system (2 elutions) to give **2ae** as a yellow oil (26.7 mg, 0.13 mmol, 26%). **^{1}H NMR** (400 MHz, $CDCl_3$) δ 7.37 – 7.28 (m, 3H), 7.16 (ddd, $^3J_{H-H}$ = 4.2 Hz, $^4J_{H-H}$ = 1.8 Hz, J_{H-F} = 0.9 Hz, 1H), 7.16 – 7.11 (m, 2H), 7.09 – 7.06 (m, 1H), 6.27 (dd, $^3J_{H-H}$ = 4.1 Hz, $^3J_{H-H}$ = 2.6 Hz, 1H), 5.50 (s, 2H). **^{13}C{^{1}H} NMR** (126 MHz, $CDCl_3$) δ 151.0 (d, $^1J_{C-F}$ = 323 Hz), 136.8, 132.4 (d, $^3J_{C-F}$ = 5.5 Hz), 128.8 (2C), 128.0, 127.2 (2C), 122.9 (d, $^4J_{C-F}$ = 3.3 Hz), 116.5 (d, $^2J_{C-F}$ = 85.3 Hz), 109.8,

52.1. **^{19}F{^{1}H} NMR** (377 MHz, $CDCl_3$) δ 18.6. **IR** (ATR) 3120 (w), 3033 (w), 2939 (w), 1776 (s), 1530 (w), 1501 (w), 1468 (w), 1450 (w), 1403 (m), 1356 (w), 1327 (m), 1236 (m), 1077 (m), 148 (m), 979 (s), 885 (m), 754 (m), 725 (s), 696 (m). **HRMS-ESI** *(m/z)* $[M]^+$ calculated for $C_{12}H_{11}FNO$, 204.0819; found, 204.0811.

1-Methyl-1*H*-pyrrolo[2,3-*b*]pyridine-3-carbonyl fluoride 2af

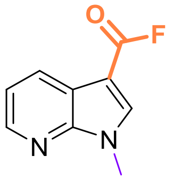

According to OP1, 1-methyl-1*H*-pyrrolo[2,3-*b*]pyridine-3-carbonyl fluoride **2af** was obtained from 1-methyl-1*H*-pyrrolo[2,3-*b*]pyridine **1aa** (60 µL, 0.50 mmol, 1.0 equiv.), DNTFB (0.47 mL, 3.03 mmol, 2.0 equiv.) and DMAP (185.3 mg, 1.52 mmol, 3.0 equiv.) with a 2-hour reaction time. The crude material was purified by flash column chromatography (PE/Et_2O 50:50, R_f = 0.38) to give **2af** as an orange solid (71.9 mg, 0.40 mmol, 80%). **^{1}H NMR** (400 MHz, $CDCl_3$) δ 8.47 (dd, $^{3}J_{H\text{-}H}$ = 4.8 Hz, $^{4}J_{H\text{-}H}$ = 1.6 Hz, 1H), 8.37 (dt, $^{3}J_{H\text{-}H}$ = 7.9 Hz, $^{4}J_{H\text{-}H}$ = 1.7 Hz, 1H), 8.02 (s, 1H), 7.31 (dd, $^{3}J_{H\text{-}H}$ = 7.9 Hz, $^{3}J_{H\text{-}H}$ = 4.7 Hz, 1H), 3.99 (s, 3H). **^{13}C{^{1}H} NMR** (126 MHz, $CDCl_3$) δ 154.2 (d, $^{1}J_{C\text{-}F}$ = 328 Hz), 148.5, 145.3, 137.9, 129.8, 119.5 (d, $^{3}J_{C\text{-}F}$ = 5.7 Hz), 119.1, 99.9 (d, $^{2}J_{C\text{-}F}$ = 72.1 Hz), 32.4. **^{19}F{^{1}H} NMR** (377 MHz, $CDCl_3$) δ 22.7. **IR** (ATR) 3094 (w), 3039 (w), 2944 (w), 1780 (s), 1605 (w), 1580 (m), 1528 (m), 1476 (w), 1445 (m), 1377 (m), 1344 (m), 1304 (m), 1153 (m), 1120 (m), 1086 (m), 1040 (m), 954 (m), 929 (s), 797 (m), 776 (m), 736 (s). **HRMS-ESI** *(m/z)* $[M+H]^+$ calculated for $C_9H_8FN_2O$, 179.0615; found, 179.0620. **mp**: 103.7 °C.

VII. Post-functionalisation reactions

General procedure 2 (GP2) for the one-pot, two-step formation of amide bonds.

This procedure was adapted from the work of Qin *et al.*[33] In a 10 mL screw-cap tube cap, DNTFB (0.16 mL, 1.03 mmol, 2.0 equiv.) was added to a solution of DMAP (61.1 mg, 0.50 mmol, 1.0 equiv.) and 5-bromo-1-methyl-*1H*-indole **1q** (106 mg, 0.50 mmol, 1.0 equiv.) in MeCN (1.5 mL). The tube was closed and the solution was stirred at 80 °C for 120 min. Then the tube was allowed to cool down to room temperature, the solution was degassed by argon bubbling and the desired amine-HCl salt (1.60 mmol, 3.2 equiv.), a base (3.50 or 6.00 mmol, 7.0 or 12.0 equiv.) and MeCN (3.0 mL) were added. The tube was closed and the reaction mixture was stirred at 80 °C for 18 hours. The reaction mixture was then diluted with water (10 mL) and extracted with EtOAc (3 × 10 mL). The combined organic layers were dried over anhydrous $MgSO_4$ and concentrated under vacuum. The residue was purified by column chromatography using the appropriate eluting system (given in V:V).

Ethyl (*2S*)-2-[(5-bromo-1-methyl-*1H*-indol-3-yl)formamido]-3-methylbutanoate 2q1

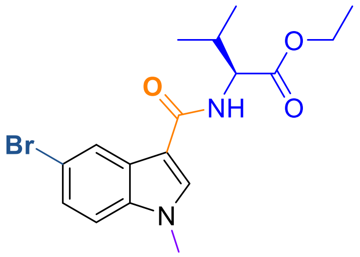

According to GP2, ethyl (2S)-2-[(5-bromo-1-methyl-*1H*-indol-3-yl)formamido]-3-methylbutanoate **2q1** was obtained from 5-bromo-1-methyl-1*H*-indole **1q** (106 mg, 0.50 mmol, 1.0 equiv.), DNTFB (0.16 mL, 1.03 mmol, 2.0 equiv.), DMAP (61.7 mg, 0.51 mmol, 1.0 equiv.), ethyl L-valinate hydrochloride (292 mg, 1.61 mmol, 3.2 equiv.) and KOH (195 mg, 3.48 mmol, 6.9 equiv.). The crude material was purified by flash column chromatography (DCM/PE/Et_2O 60:35:5, R_f = 0.27) to give **2q1** as a dark yellow solid (134 mg, 0.35 mmol, 70%). **^{1}H NMR** (400 MHz, $CDCl_3$) δ 8.24 (dd, $^4J_{H\text{-}H}$ = 2.0 Hz, $^5J_{H\text{-}H}$ = 0.6 Hz, 1H), 7.60 (s, 1H), 7.36 (dt, $^3J_{H\text{-}H}$ = 8.7 Hz, $^4J_{H\text{-}H}$ = 1.5 Hz, 1H), 7.18 (d, $^3J_{H\text{-}H}$ = 8.7 Hz, 1H), 6.39 (d, ^3J = 8.6 Hz, 1H), 4.81 (dd, $^3J_{H\text{-}H}$ = 8.7 Hz, $^3J_{H\text{-}H}$ = 4.8 Hz, 1H), 4.34 – 4.18 (m, 2H), 3.80 (s, 3H), 2.30 (m, 1H), 1.33 (t, $^3J_{H\text{-}H}$ = 7.1 Hz, 3H), 1.03 (dd, $^3J_{H\text{-}H}$ = 11.0 Hz, $^4J_{H\text{-}H}$ = 6.9 Hz, 6H). **^{13}C{^{1}H} NMR** (126 MHz, $CDCl_3$) δ 172.7, 164.3, 135.8, 132.4, 127.4, 125.7, 123.4, 115.2, 111.3, 110.1, 61.4, 56.9, 33.5, 31.7, 19.1, 18.1, 14.3. **IR** (ATR) 3358 (m), 3109 (w), 2967 (w), 2930 (w), 2878 (w), 2289(w), 1731 (s), 1643 (m), 1624 (s), 1535 (s), 1514 (m), 1464 (s), 1424 (m), 1374 (m), 1301 (m), 1268 (m), 1231 (m), 1206 (s), 1151 (s), 1120 (m), 1040 (m), 1019 (m), 929 (m), 914 (m), 896 (m), 862 (m), 819 (m), 788 (m), 770 (s), 754 (s), 733 (s). **HRMS-ESI** *(m/z)* $[M+Na]^+$ calculated for $C_{17}H_{21}{}^{79}BrN_2NaO_3$, 403.0628; found, 403.0643. **mp**: 62.1 °C.$[\alpha]^{20}_{589}$ = -20.53 °. This value is a mean of $[\alpha]^{20}_{589}$ measured on 2 concentrations with a 1 dm cell length. These values were confirmed by the use of a 2 dm cell length as the solution of **2q1** in methanol appeared to be yellow. Measured values are reported in Table S2.

*Table S2: Specific rotation measurements for a solution of **2p1** in MeOH*

Cell length (dm)	[2q1] in MeOH (g/mL)	α (°) measured	$[\alpha]^{20}_{589}$ (°) calculated	$[\alpha]^{20}_{589}$ mean (°)
1	0.00134	-0.026	-19.55	-20.53
	0.00218	-0.047	-21.51	
2	0.00134	-0.057	-21.27	-20.50
	0.00218	-0.086	-19.72	

Chiral HPLC: Chiralcel® OD-H column, Hex/IPA 80/20, 0.5 mL/min, 35 °C, 223 nm, t_R = 22.0 & 35.2 min (*vide infra*).

Ethyl 2-[(5-bromo-1-methyl-*1H*-indol-3-yl)formamido]-3-methylbutanoate rac-2q1

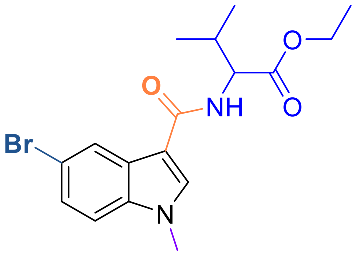

A mixture of ethyl 2-amino-3-methylbutanoate (75.3 mg, 0.52 mmol, 1.0 equiv.), 5-bromo-1-methyl-1*H*-indole-3-carbonyl fluoride **2q** (260 mg, 1.02 mmol, 2.0 equiv.), KOH (92.5 mg, 1.65 mmol, 3.3 equiv.) and MeCN (2.3 mL) was stirred at 80 °C for 18 hours. The mixture was then diluted with water (10 mL) and extracted with ethyl acetate (3 × 10 mL). The extracts were dried over anhydrous $MgSO_4$ and concentrated under vacuum. The residue was purified by column chromatography (*c*-hexane/EtOAc 100:0 to 75:25, R_f = 0.36 *c*-hexane/EtOAc 50:50) to give **rac-2q1** as an orange solid (117 mg, 0.31 mmol, 58%). **^{1}H NMR** (400 MHz, $CDCl_3$) δ 8.24 (d, $^4J_{H\text{-}H}$ = 1.9 Hz, 1H), 7.62 (s, 1H), 7.38 (dd, $^3J_{H\text{-}H}$ = 8.7, $^4J_{H\text{-}H}$ = 1.9 Hz, 1H), 7.21 (d, $^3J_{H\text{-}H}$ = 8.6 Hz, 1H), 6.34 (d, $^3J_{H\text{-}H}$ = 8.6 Hz, 1H), 4.81 (dd, $^3J_{H\text{-}H}$ = 8.6, $^4J_{H\text{-}H}$ 4.8 Hz, 1H), 4.32 – 4.19 (m, 2H), 4.12 (q, $^3J_{H\text{-}H}$ = 7.1 Hz, 1H), 3.81 (s, 3H), 2.30 (m, 1H), 1.33 (t, $^3J_{H\text{-}H}$ = 7.1 Hz, 3H), 1.03 (dd, $^3J_{H\text{-}H}$ = 11.3, $^4J_{H\text{-}H}$ = 6.9 Hz, 6H). **^{13}C{^{1}H} NMR** (126 MHz, $CDCl_3$) δ 172.5, 164.2, 135.9, 132.4, 127.4, 125.7, 123.4, 115.3, 111.3, 110.2, 61.3, 56.9, 33.5, 31.7, 19.1, 18.0, 14.3. **Chiral HPLC**: Chiralcel® OD-H column, Hex/IPA 80/20, 0.5 mL/min, 35 °C, 223 nm, t_R = 22.0 & 35.2 min (*vide infra*).

Ethyl 2-[(5-bromo-1-methyl-*1H*-indol-3-yl)formamido]-3-hydroxypropanoate 2q2

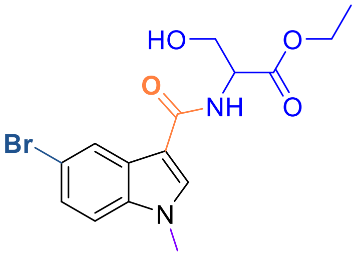

According to GP2, ethyl 2-[(5-bromo-1-methyl-*1H*-indol-3-yl)formamido]-3-hydroxypropanoate **2q2** was obtained from 5-bromo-1-methyl-1*H*-indole **1q** (105 mg, 0.50 mmol, 1.0 equiv.), DNTFB (0.16 mL, 1.03 mmol, 2.0 equiv.), DMAP (62.0 mg, 0.51 mmol, 1.0 equiv.), ethyl 2-amino-3-hydroxypropanoate hydrochloride (269 mg, 1.59 mmol, 3.2 equiv.) and NEt_3 (0.49 mL, 3.53 mmol, 7.1 equiv.). The crude material was purified by flash column chromatography (DCM/MeOH 98:2, R_f = 0.18) to give **2q2** as a dark yellow solid (88.2 mg, 0.24 mmol, 48%). **^{1}H NMR** (400 MHz, $CDCl_3$) δ 8.22 (d, $^4J_{H\text{-}H}$ = 1.8 Hz, 1H), 7.64 (s, 1H), 7.36 (dd, $^3J_{H\text{-}H}$ = 8.7 Hz, $^4J_{H\text{-}H}$ = 1.9 Hz, 1H), 7.17 (d, $^3J_{H\text{-}H}$ = 8.7 Hz, 1H), 6.84 (d, $^3J_{H\text{-}H}$ = 6.8 Hz, 1H), 4.89 (m, 1H), 4.32 (q, $^3J_{H\text{-}H}$ = 7.1 Hz, 2H), 4.09 (app. qd, $^3J_{H\text{-}H}$ = 11.0 Hz, $^{TS}J_{H\text{-}H}$ = 3.7 Hz, 2H), 3.78 (s, 3H), 2.96 (br s, 1H), 1.35 (t, $^3J_{H\text{-}H}$ = 7.1 Hz, 3H). **^{13}C{^{1}H} NMR** (126 MHz, $CDCl_3$) δ 171.1, 165.0, 136.0, 133.1, 127.4, 125.9, 123.4, 115.5, 111.5, 109.6, 64.4, 62.3, 55.4, 33.7, 14.6. **IR** (ATR) 3337 (br w), 3109 (w), 2986 (w), 2942 (w), 1736 (m), 1624 (m), 1540 (s), 1468 (m), 1372 (m), 1189 (s), 1146 (m), 1069 (m), 1019 (m), 856 (m), 765

(m). **HRMS-ESI** *(m/z)* $[M+Na]^+$ calculated for $C_{15}H_{17}{}^{79}BrN_2NaO_4$, 391.0239; found, 391.0261. **mp**: 55.3 °C.

5-Bromo-*N*-methoxy-*N*,1-dimethyl-*1H*-indole-3-carboxamide 2q3

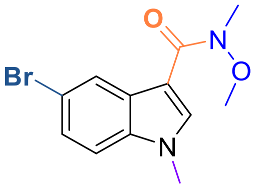

According to GP2, 5-bromo-*N*-methoxy-*N*,1-dimethyl-*1H*-indole-3-carboxamide **2q3** was obtained from 5-bromo-1-methyl-1*H*-indole **1q** (106 mg, 0.50 mmol, 1.0 equiv.), DNTFB (0.16 mL, 1.03 mmol, 2.0 equiv.), DMAP (63.6 mg, 0.52 mmol, 1.0 equiv.), *N*-methoxymethylamine hydrochloride (157 mg, 1.61 mmol, 3.2 equiv.) and KOH (338 mg, 1.61 mmol, 12.0 equiv.). The crude material was purified by flash column chromatography (DCM/PE/Et_2O 60:35:5, R_f = 0.27) to give **2q3** as an orange oil (96.3 mg pure at 97.9w%, 0.24 mmol, 62%). **^{1}H NMR** (400 MHz, $CDCl_3$) δ 8.59 (dd, $^4J_{H-H}$ = 2.0 Hz, $^5J_{H-H}$ = 0.6 Hz, 1H), 7.80 (s, 1H), 7.37 (dd, $^3J_{H-H}$ = 8.7 Hz, $^4J_{H-H}$ = 2.0 Hz, 1H), 7.19 (dd, $^3J_{H-H}$ = 8.7 Hz, 0.6 Hz, 1H), 3.82 (s, 3H), 3.72 (s, 2H), 3.38 (s, 3H). **^{13}C{^{1}H} NMR** (126 MHz, $CDCl_3$) δ 165.3, 135.2, 134.6, 130.2, 125.9, 125.4, 115.5, 110.8, 107.1, 60.9, 33.7, 33.1. **IR** (ATR) 2934 (br w), 2242 (w), 1609 (m), 1563 (w), 1517 (m), 1461 (s), 1415 (m), 1369 (s), 1271 (m), 1243 (m), 1209 (m), 1169 (m), 1142 (m), 1049 (m), 1013 (m), 960 (m), 911 (m), 890 (m), 868 (m), 794 (s), 754 (s), 724 (s). **HRMS-ESI** *(m/z)* $[M+Na]^+$ calculated for $C_{12}H_{13}{}^{79}BrN_2NaO_2$, 319.0053; found, 319.0049.

A. Chiral-HPLC analyses of 2q1 and *rac*-2q1

Ethyl 2-[(5-bromo-1-methyl-*1H*-indol-3-yl)formamido]-3-methylbutanoate **rac-2q1**: Chiralcel® OD-H column, Hex/IPA 80/20, 0.5 mL/min, 35 °C, 223 nm.

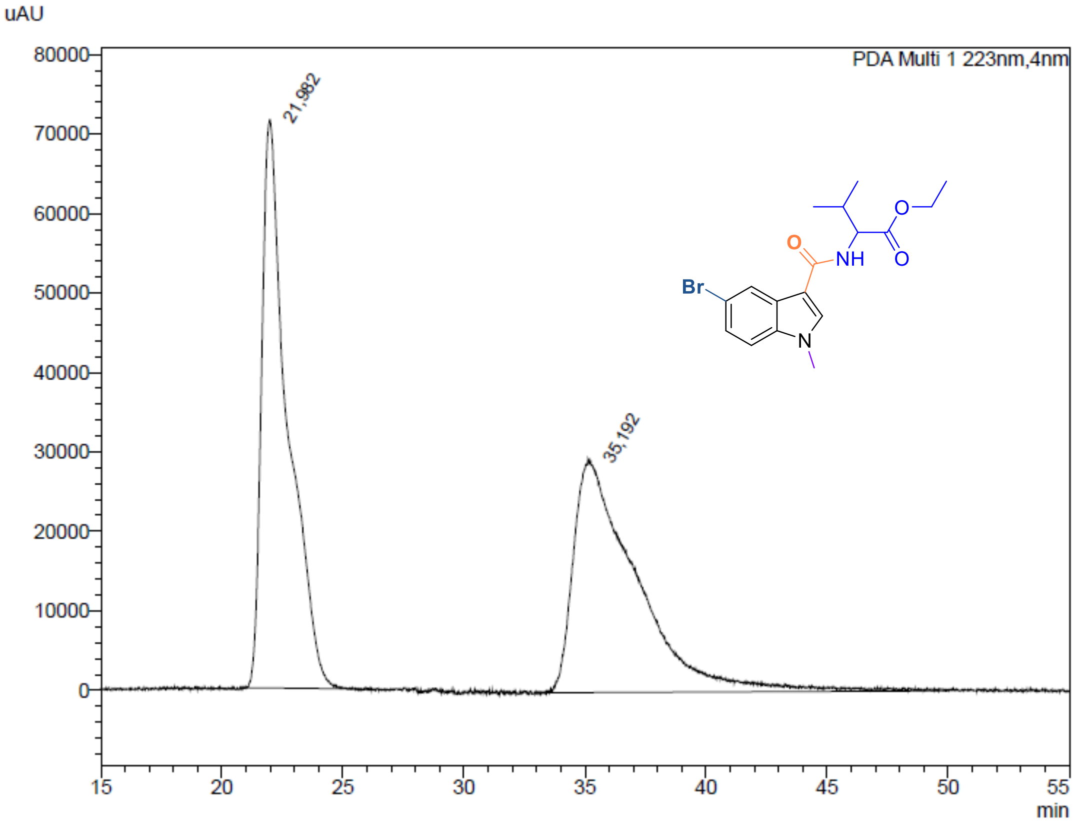

<PDA Chromatogram>

Peak Table

PDA Ch1 223nm

Peak#	Ret. Time	Area	Height	Area%	Capacity Factor(k')	Resolution(USP)	Lambda max
1	21,982	5362708	71510	50,078	--	--	223/200/293/660
2	35,192	5346086	29234	49,922	0,601	3,818	223/200/294/488
Total		10708794	100745	100,000			

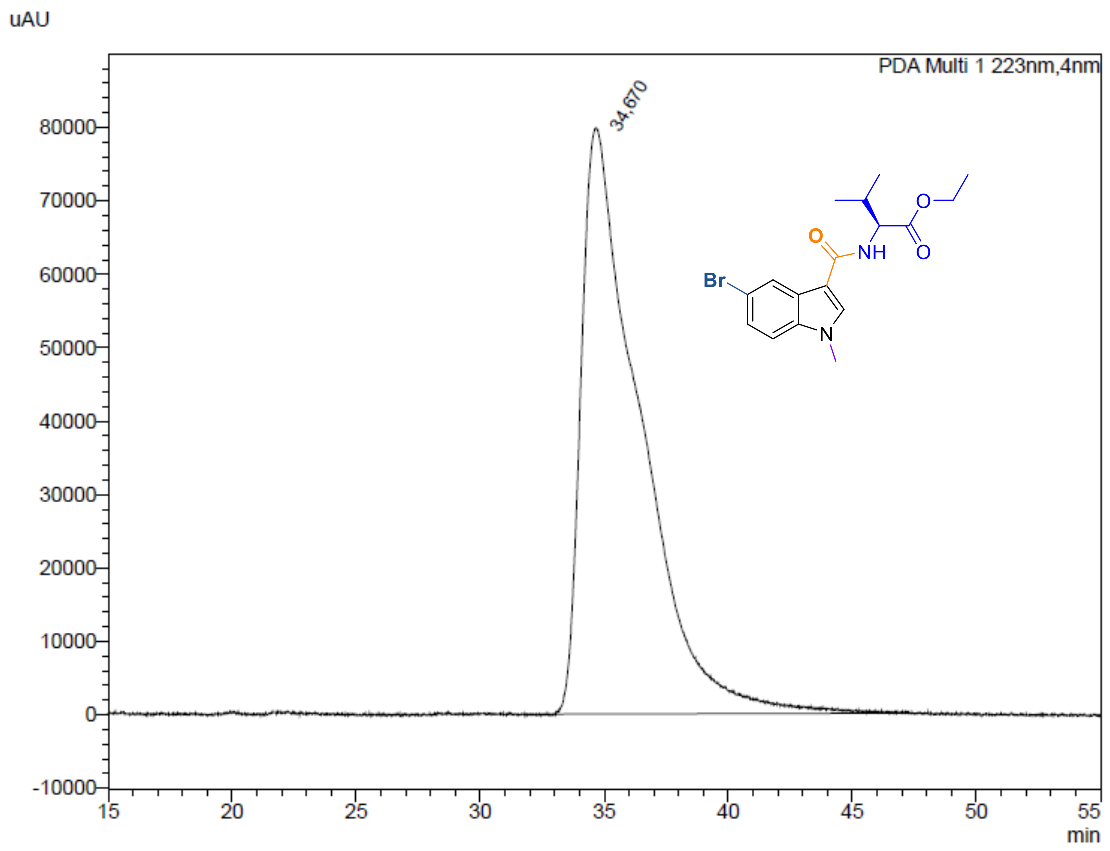

<PDA Chromatogram>

Peak Table

PDA Ch1 223nm

Peak#	Ret. Time	Area	Height	Area%	Capacity Factor(k')	Resolution(USP)	Lambda max
1	34.670	13233644	79846	100.000	--	--	223/200/293/488/400
Total		13233644	79846	100.000			

VIII. Bibliography

[1] J. F. Stadlwieser, M. E. Dambaur, *Helv. Chim. Acta* **2006**, *89*, 936–946.

[2] E. G. L. Robert, V. Pirenne, M. D. Wodrich, J. Waser, *Angew. Chem. Int. Ed.* **2023**, *62*, e202302420.

[3] E. M. Galathri, L. D. Terlizzi, M. Fagnoni, S. Protti, C. G. Kokotos, *Org. Biomol. Chem.* **2023**, *21*, 365–369.

[4] S. Sar, A. Tripathi, K. D. Dubey, S. Sen, *J. Org. Chem.* **2020**, *85*, 3748–3756.

[5] H. Ishikawa, D. A. Colby, S. Seto, P. Va, A. Tam, H. Kakei, T. J. Rayl, I. Hwang, D. L. Boger, *J. Am. Chem. Soc.* **2009**, *131*, 4904–4916.

[6] S. Potavathri, K. C. Pereira, S. I. Gorelsky, A. Pike, A. P. LeBris, B. DeBoef, *J. Am. Chem. Soc.* **2010**, *132*, 14676–14681.

[7] A. D. Yamaguchi, D. Mandal, J. Yamaguchi, K. Itami, *Chem. Lett.* **2011**, *40*, 555–557.

[8] R. R. Reddy, K. Adlak, P. Ghorai, *J. Org. Chem.* **2017**, *82*, 8426–8437.

[9] C. Wei, J. Wu, L. Zhang, Z. Xia, *Org. Lett.* **2022**, *24*, 4689–4693.

[10] A. Kasahara, T. Izumi, S. Murakami, K. Miyamoto, T. Hino, *J. Heterocycl. Chem.* **1989**, *26*, 1405–1413.

[11] J.-L. Zhou, M.-C. Ye, X.-L. Sun, Y. Tang, *Tetrahedron* **2009**, *65*, 6877–6881.

[12] W. Zhuang, J. Zhang, C. Ma, J. S. Wright, X. Zhang, S.-F. Ni, Q. Huang, *Org. Lett.* **2022**, *24*, 4229–4233.

[13] S. Choi, J. Park, E. Yu, J. Sim, C.-M. Park, *Angew. Chem. Int. Ed.* **2020**, *59*, 11886–11891.

[14] S. Maiti, J. S. Kim, J. Kim, *New J. Chem.* **2024**, *48*, 3342–3346.

[15] J. G. Allen, K. Briner, M. P. Cohen, C. S. Galka, S. L. Hellman, M. A. Martinez-Grau, M. R. Reinhard, M. J. Rodriguez, R. R. Rothhaar, M. W. Tidwell, F. Victor, A. C. Williams, D. Zhang, S. A. Boyd, R. G. Conway, A. S. Deo, W.-M. Lee, C. S. Siedem, A. Singh, *2,3,4,5-Tetrahydro-1h-Benzo[d]Azepines Substitues En Position 6 En Tant Qu'agonistes De Recepteur 5-Ht2c*, **2005**, WO2005082859A1.

[16] C. Ostacolo, V. Di Sarno, G. Lauro, G. Pepe, S. Musella, T. Ciaglia, V. Vestuto, G. Autore, G. Bifulco, S. Marzocco, P. Campiglia, I. M. Gomez-Monterrey, A. Bertamino, *Eur. J. Med. Chem.* **2019**, *167*, 61–75.

[17] N. Jacob, Y. Zaid, J. C. A. Oliveira, L. Ackermann, J. Wencel-Delord, *J. Am. Chem. Soc.* **2022**, *144*, 798–806.

[18] S. Liu, Y. Huang, J. Wang, F.-L. Qing, X.-H. Xu, *J. Am. Chem. Soc.* **2022**, *144*, 1962–1970.

[19] M. Kitano, A. Kojima, K. Nakano, A. Miyagishi, T. Noguchi, N. Ohashi, *Chem. Pharm. Bull.* **1999**, *47*, 1538–1548.

[20] L. Xiong, X.-L. Zhu, H.-W. Gao, Y. Fu, S.-Q. Hu, L.-N. Jiang, W.-C. Yang, G.-F. Yang, *J. Agric. Food Chem.* **2016**, *64*, 4830–4837.

[21] S. Zhou, J. Wang, L. Wang, K. Chen, C. Song, J. Zhu, *Org. Lett.* **2016**, *18*, 3806–3809.

[22] Y.-Z. Ji, H.-J. Li, Y.-R. Wang, Z.-Y. Zhang, Y.-C. Wu, *Adv. Synth. Catal.* **2020**, *362*, 1039–1045.

[23] S. M. Bronner, K. B. Bahnck, N. K. Garg, *Org. Lett.* **2009**, *11*, 1007–1010.

[24] F. J. Lundevall, H.-R. Bjørsvik, *Eur. J. Org. Chem.* **2023**, *26*, e202201504.

[25] J. K. Laha, M. K. Hunjan, T. Maity, A. Hazra, *Adv. Synth. Catal.* **2023**, *365*, 1238–1246.

[26] B. G. Nangunuri, R. P. Shirke, M. Kim, *Org. Biomol. Chem.* **2023**, *21*, 960–965.

[27] A. V. R. D. Lisboa, G. Duran-Camacho, A. K. Ehrlacher, M. R. Lasky, M. S. Sanford, *Org. Lett.* **2023**, *25*, 9025–9029.

[28] N. Ree, A. H. Göller, J. H. Jensen, *Digital Discovery* **2024**, *3*, 347–354.

[29] Y. Ogiwara, Y. Sakurai, H. Hattori, N. Sakai, *Org. Lett.* **2018**, *20*, 4204–4208.

[30] Y. Du, Y. Wang, X. Li, Y. Shao, G. Li, R. D. Webster, Y. R. Chi, *Org. Lett.* **2014**, *16*, 5678–5681.

[31] S. Zhao, Y. Guo, Z. Su, C. Wu, W. Chen, Q.-Y. Chen, *Chin. J. Chem.* **2021**, *39*, 1225–1232.

[32] M. G. Darnowski, T. D. Lanosky, A. R. Paquette, C. N. Boddy, *J. Org. Chem.* **2022**, *87*, 15634–15643.

[33] Q.-X. Wu, T. Shu, W.-Y. Fang, H.-L. Qin, *Eur. J. Org. Chem.* **2022**, *2022*, e202200719.

IX. Crystal description of 2z[5]

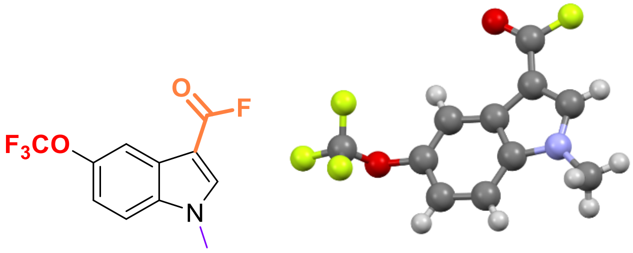

CCDC Deposition number: 2321178

Table S2: Crystal data and structure refinement for ***2z*****[5]**

Empirical formula	$C_{11} H_7 F_4 N O_2$
Formula weight	261.18
Temperature	173(2) K
Wavelength	0.71073 Å
Crystal system, space group	Triclinic, P -1
Unit cell dimensions	a = 7.4681(9) Å alpha = 67.968(4)°
	b = 8.5179(11) Å beta = 89.286(4)°
	c = 10.1439(13) Å gamma = 65.402(5)°
Volume	535.91(12) $Å^3$
Z, Calculated density	2, 1.619 Mg/m^3
Absorption coefficient	0.157 mm^{-1}
F(000)	264
Crystal size	0.240 x 0.200 x 0.180 mm
Theta range for data collection	2.198 to 28.016°
Limiting indices	-9<=h<=9, -8<=k<=11, -13<=l<=13
Reflections collected / unique	9498 / 2590 [R(int) = 0.0186]
Completeness to theta = 25.242	99.9%
Absorption correction	Semi-empirical from equivalents
Max. and min. transmission	0.7456 and 0.7068
Refinement method	Full-matrix least-squares on F^2
Data / restraints / parameters	2590 / 0 / 164
Goodness-of-fit on F^2	1.044
Final R indices [I>2sigma(I)]	R1 = 0.0416, wR2 = 0.1031
R indices (all data)	R1 = 0.0520, wR2 = 0.1116
Extinction coefficient	n/a

Largest diff. peak and hole	0.390 and -0.351 e.Å^{-3}

*Table S3: Atomic coordinates (x 10^4) and equivalent isotropic displacement parameters (Å^2 x 10^3) for **2z^5**. U(eq) is defined as one third of the trace of the orthogonalized Uij tensor.*

	x	y	z	U(eq)
N(1)	7893(2)	3444(2)	6849(1)	28(1)
C(1)	6492(2)	4898(2)	7097(2)	29(1)
C(2)	5720(2)	6514(2)	5838(2)	26(1)
C(3)	6720(2)	6031(2)	4726(2)	24(1)
C(4)	6651(2)	7032(2)	3262(2)	27(1)
C(5)	7919(2)	6022(2)	2560(2)	29(1)
C(6)	9249(2)	4107(2)	3228(2)	32(1)
C(7)	9343(2)	3109(2)	4675(2)	30(1)
C(8)	8072(2)	4094(2)	5404(2)	25(1)
C(9)	8984(3)	1499(2)	7893(2)	39(1)
C(10)	4120(2)	8307(2)	5646(2)	33(1)
O(1)	3303(2)	9656(2)	4486(1)	37(1)
F(1)	3499(2)	8441(2)	6853(1)	49(1)
O(2)	7992(2)	7024(2)	1092(1)	38(1)
C(11)	6795(3)	7101(3)	88(2)	43(1)
F(2)	7036(3)	5424(2)	206(1)	81(1)
F(3)	7135(2)	7989(2)	-1193(1)	56(1)
F(4)	4861(2)	8041(2)	119(1)	71(1)

*Table S4: Bond lengths [Å] and angles [°] for **2z^5**.*

N(1)-C(1)	1.3567(19)
N(1)-C(8)	1.3866(18)
N(1)-C(9)	1.453(2)
C(1)-C(2)	1.377(2)
C(1)-H(1)	0.9500
C(2)-C(10)	1.436(2)
C(2)-C(3)	1.4393(19)
C(3)-C(4)	1.397(2)
C(3)-C(8)	1.412(2)
C(4)-C(5)	1.381(2)
C(4)-H(4)	0.9500
C(5)-C(6)	1.395(2)
C(5)-O(2)	1.4250(18)
C(6)-C(7)	1.378(2)
C(6)-H(6)	0.9500
C(7)-C(8)	1.391(2)
C(7)-H(7)	0.9500
C(9)-H(9A)	0.9800
C(9)-H(9B)	0.9800
C(9)-H(9C)	0.9800
C(10)-O(1)	1.224(2)
C(10)-F(1)	1.3317(19)

O(2)-C(11)	1.329(2)
C(11)-F(3)	1.3185(19)
C(11)-F(2)	1.320(2)
C(11)-F(4)	1.335(2)
C(1)-N(1)-C(8)	108.75(12)
C(1)-N(1)-C(9)	126.50(14)
C(8)-N(1)-C(9)	124.70(13)
N(1)-C(1)-C(2)	110.08(13)
N(1)-C(1)-H(1)	125.0
C(2)-C(1)-H(1)	125.0
C(1)-C(2)-C(10)	126.53(14)
C(1)-C(2)-C(3)	107.05(12)
C(10)-C(2)-C(3)	126.29(14)
C(4)-C(3)-C(8)	119.32(13)
C(4)-C(3)-C(2)	134.76(13)
C(8)-C(3)-C(2)	105.89(12)
C(5)-C(4)-C(3)	116.63(13)
C(5)-C(4)-H(4)	121.7
C(3)-C(4)-H(4)	121.7
C(4)-C(5)-C(6)	124.05(14)
C(4)-C(5)-O(2)	118.15(14)
C(6)-C(5)-O(2)	117.61(13)
C(7)-C(6)-C(5)	119.83(13)
C(7)-C(6)-H(6)	120.1
C(5)-C(6)-H(6)	120.1
C(6)-C(7)-C(8)	117.16(14)
C(6)-C(7)-H(7)	121.4
C(8)-C(7)-H(7)	121.4
N(1)-C(8)-C(7)	128.76(14)
N(1)-C(8)-C(3)	108.23(12)
C(7)-C(8)-C(3)	123.01(14)
N(1)-C(9)-H(9A)	109.5
N(1)-C(9)-H(9B)	109.5
H(9A)-C(9)-H(9B)	109.5
N(1)-C(9)-H(9C)	109.5
H(9A)-C(9)-H(9C)	109.5
H(9B)-C(9)-H(9C)	109.5
O(1)-C(10)-F(1)	119.50(14)
O(1)-C(10)-C(2)	125.28(14)
F(1)-C(10)-C(2)	115.21(14)
C(11)-O(2)-C(5)	117.04(12)
F(3)-C(11)-F(2)	108.66(15)
F(3)-C(11)-O(2)	108.69(15)
F(2)-C(11)-O(2)	113.69(16)
F(3)-C(11)-F(4)	107.51(16)
F(2)-C(11)-F(4)	105.29(16)
O(2)-C(11)-F(4)	112.75(15)

*Table S5: Anisotropic displacement parameters ($Å^2 \times 10^3$) for **2z⁵**. The anisotropic displacement factor exponent takes the form: $-2 pi^2 [h^2 a^{*2} U11 + ... + 2 h k a^* b^* U12]$*

U11	U22	U33	U23	U13	U12	U11
N(1)	27(1)	29(1)	26(1)	-9(1)	1(1)	-12(1)
C(1)	28(1)	36(1)	29(1)	-17(1)	7(1)	-18(1)
C(2)	24(1)	30(1)	29(1)	-16(1)	6(1)	-13(1)
C(3)	20(1)	27(1)	27(1)	-14(1)	4(1)	-11(1)
C(4)	25(1)	28(1)	28(1)	-12(1)	3(1)	-13(1)
C(5)	30(1)	39(1)	26(1)	-15(1)	7(1)	-20(1)
C(6)	26(1)	41(1)	38(1)	-25(1)	11(1)	-16(1)
C(7)	23(1)	30(1)	38(1)	-18(1)	4(1)	-10(1)
C(8)	21(1)	29(1)	28(1)	-13(1)	2(1)	-12(1)
C(9)	38(1)	32(1)	34(1)	-4(1)	-2(1)	-13(1)
C(10)	28(1)	38(1)	45(1)	-26(1)	11(1)	-16(1)
O(1)	33(1)	28(1)	42(1)	-13(1)	2(1)	-6(1)
F(1)	48(1)	59(1)	51(1)	-37(1)	21(1)	-21(1)
O(2)	44(1)	51(1)	28(1)	-17(1)	13(1)	-30(1)
C(11)	55(1)	53(1)	28(1)	-17(1)	12(1)	-31(1)
F(2)	150(2)	72(1)	43(1)	-24(1)	-1(1)	-66(1)
F(3)	69(1)	73(1)	27(1)	-16(1)	17(1)	-37(1)
F(4)	48(1)	116(1)	41(1)	-23(1)	7(1)	-38(1)

*Table S6: Hydrogen coordinates ($\times 10^4$) and isotropic displacement parameters ($Å^2 \times 10^3$) for **2z⁵**.*

	X	y	z	U(eq)
H(1)	6102	4815	8007	34
H(4)	5775	8340	2773	32
H(6)	10088	3492	2686	38
H(7)	10237	1804	5155	36
H(9A)	8673	1408	8853	58
H(9B)	10423	1093	7911	58
H(9C)	8593	683	7619	58

*Table S7: Torsion angles [°] for **2z⁵**.*

C(8)-N(1)-C(1)-C(2)	0.30(16)
C(9)-N(1)-C(1)-C(2)	-177.32(13)
N(1)-C(1)-C(2)-C(10)	175.70(13)
N(1)-C(1)-C(2)-C(3)	-0.27(16)
C(1)-C(2)-C(3)-C(4)	-177.81(15)
C(10)-C(2)-C(3)-C(4)	6.2(3)
C(1)-C(2)-C(3)-C(8)	0.14(15)
C(10)-C(2)-C(3)-C(8)	-175.84(13)
C(8)-C(3)-C(4)-C(5)	0.98(19)
C(2)-C(3)-C(4)-C(5)	178.72(14)

C(3)-C(4)-C(5)-C(6)	-0.8(2)
C(3)-C(4)-C(5)-O(2)	-175.61(12)
C(4)-C(5)-C(6)-C(7)	0.2(2)
O(2)-C(5)-C(6)-C(7)	175.10(12)
C(5)-C(6)-C(7)-C(8)	0.1(2)
C(1)-N(1)-C(8)-C(7)	178.83(14)
C(9)-N(1)-C(8)-C(7)	-3.5(2)
C(1)-N(1)-C(8)-C(3)	-0.21(15)
C(9)-N(1)-C(8)-C(3)	177.46(13)
C(6)-C(7)-C(8)-N(1)	-178.73(13)
C(6)-C(7)-C(8)-C(3)	0.2(2)
C(4)-C(3)-C(8)-N(1)	178.37(12)
C(2)-C(3)-C(8)-N(1)	0.04(15)
C(4)-C(3)-C(8)-C(7)	-0.7(2)
C(2)-C(3)-C(8)-C(7)	-179.06(13)
C(1)-C(2)-C(10)-O(1)	-170.97(15)
C(3)-C(2)-C(10)-O(1)	4.2(2)
C(1)-C(2)-C(10)-F(1)	7.8(2)
C(3)-C(2)-C(10)-F(1)	-176.95(13)
C(4)-C(5)-O(2)-C(11)	-93.26(18)
C(6)-C(5)-O(2)-C(11)	91.56(18)
C(5)-O(2)-C(11)-F(3)	-176.76(13)
C(5)-O(2)-C(11)-F(2)	-55.6(2)
C(5)-O(2)-C(11)-F(4)	64.2(2)

X. NMR spectra

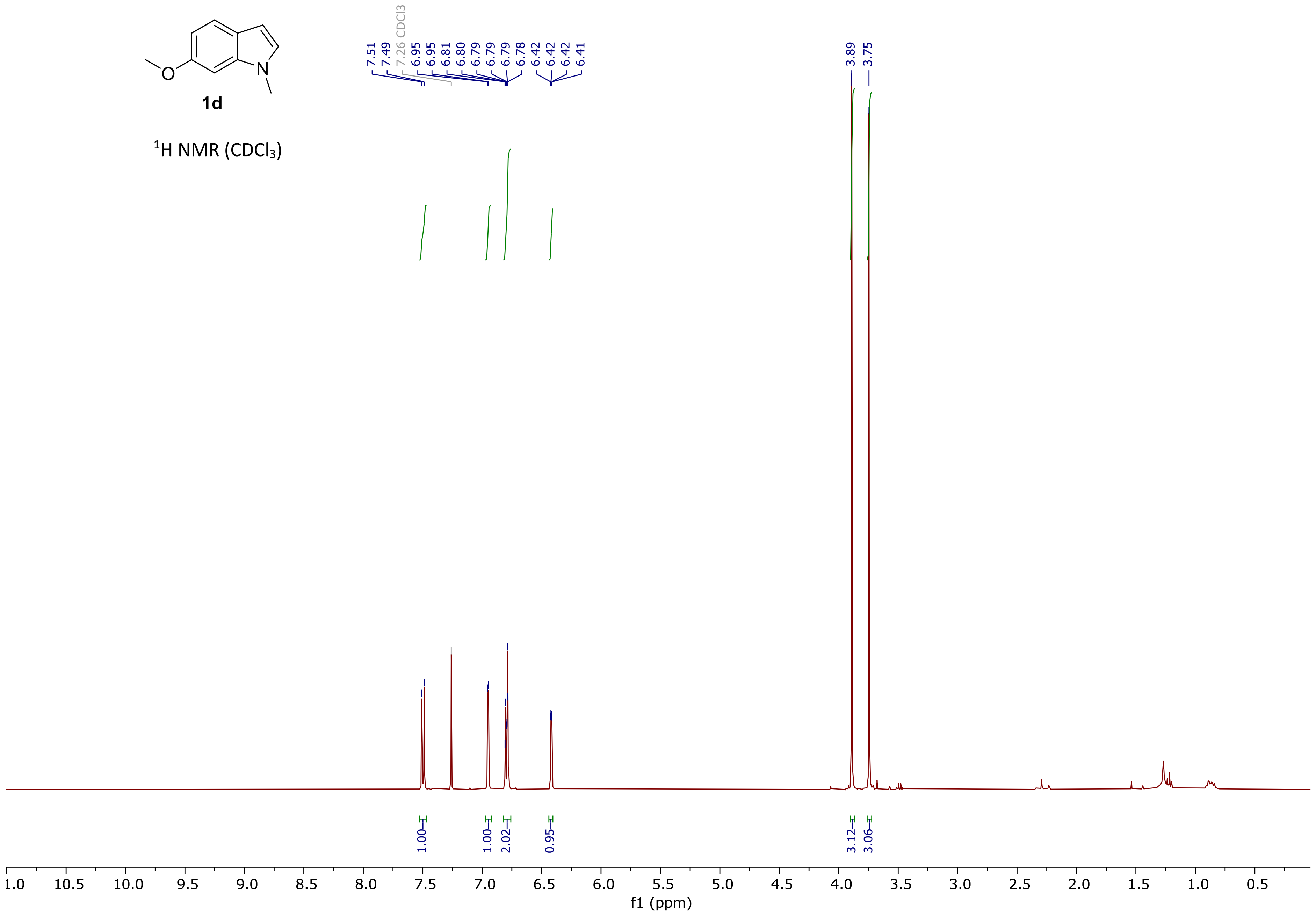
O
N
1d
1H NMR (CDCl3)
7.51
7.49
7.26 CDCl3
6.95
6.95
6.81
6.80
6.79
6.79
6.79
6.78
6.42
6.42
6.42
6.41
3.89
3.75
1.00
1.00
2.02
0.95
3.12
3.06
1.0
10.5
10.0
9.5
9.0
8.5
8.0
7.5
7.0
6.5
6.0
5.5
5.0
4.5
4.0
3.5
3.0
2.5
2.0
1.5
1.0
0.5
f1 (ppm)

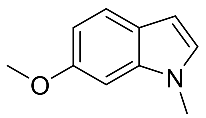

1d

$^{13}C\{^{1}H\}$ NMR ($CDCl_3$)

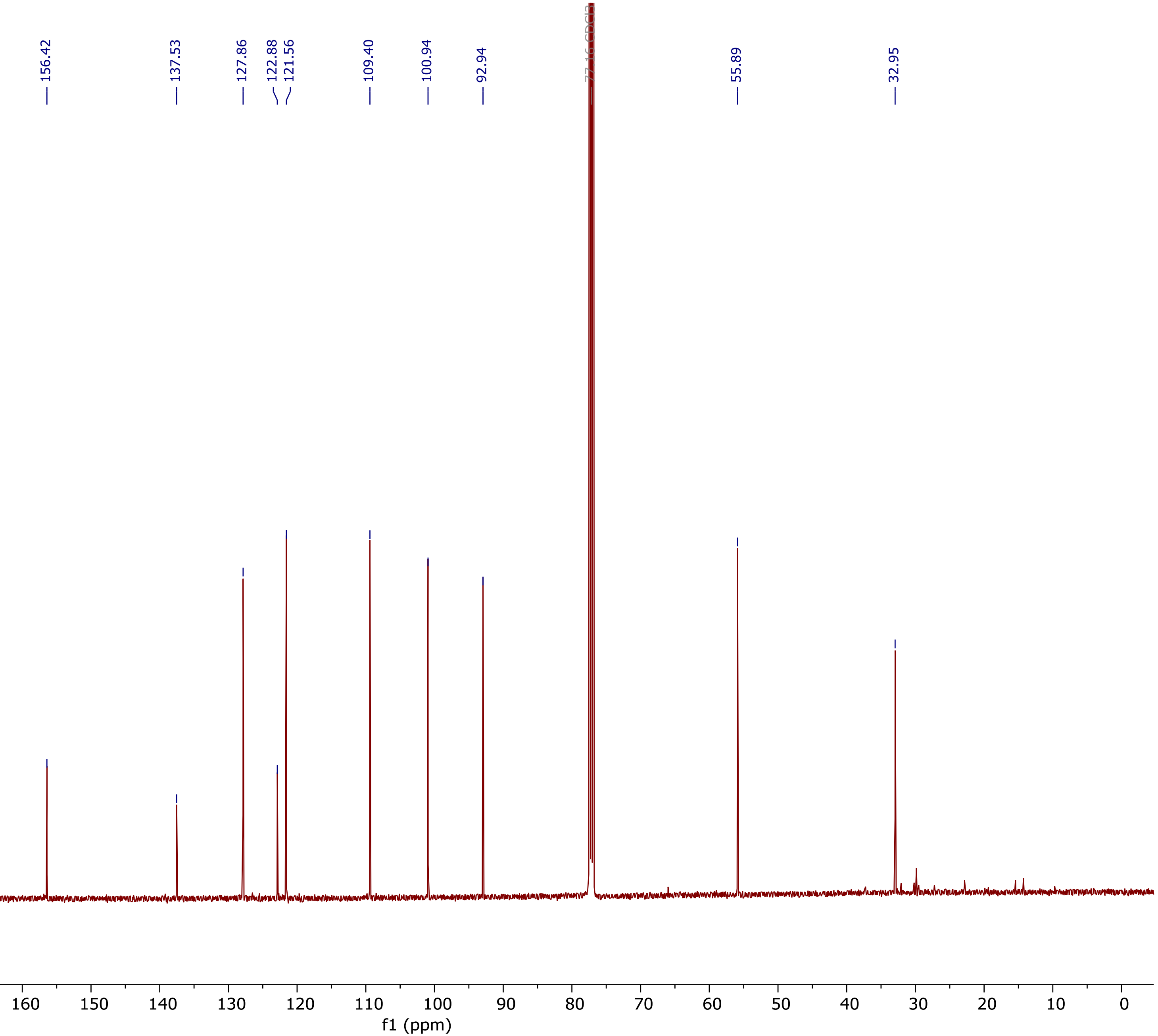

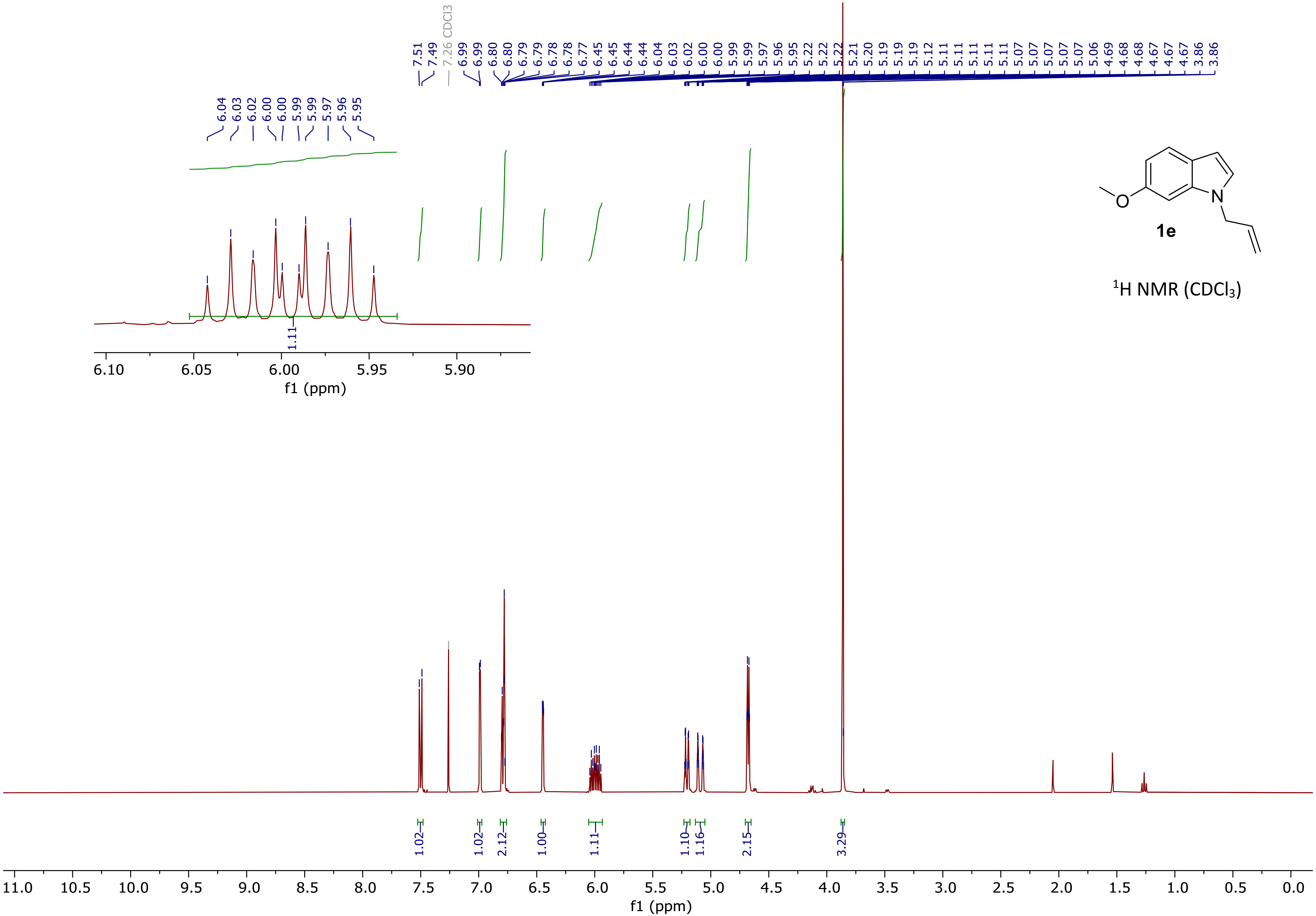

1e

^{1}H NMR ($CDCl_3$)

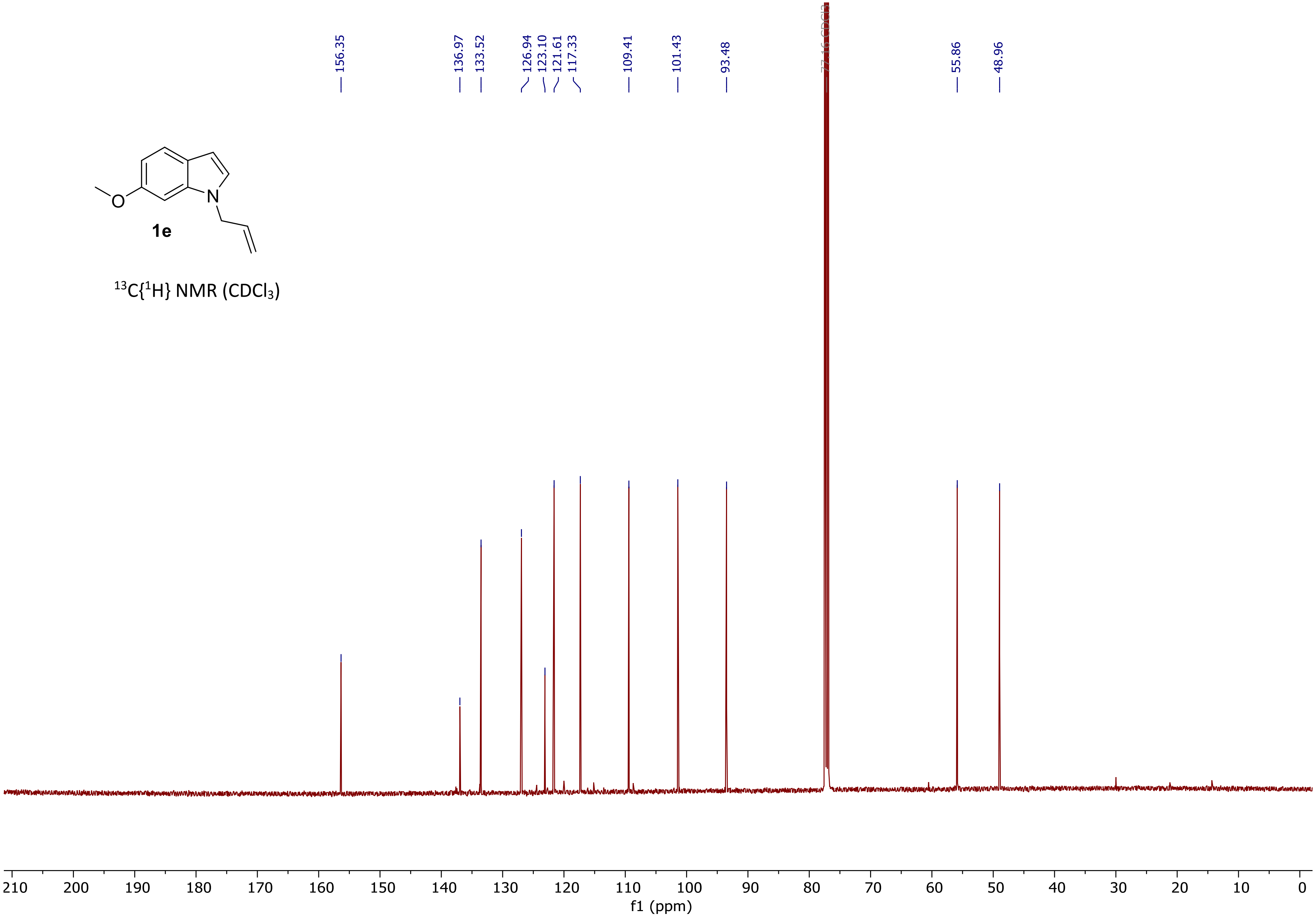

1e

$^{13}C\{^{1}H\}$ NMR ($CDCl_3$)

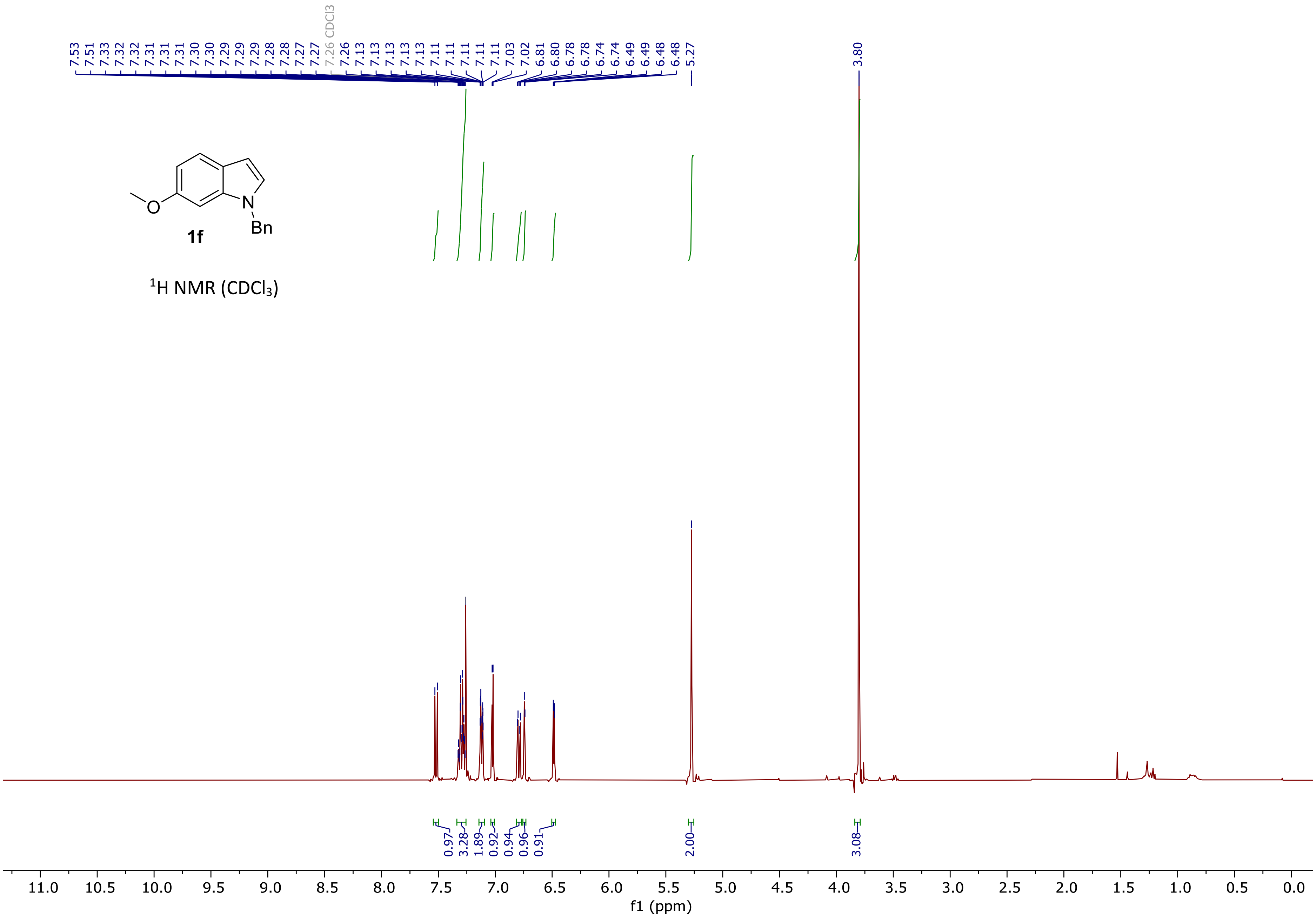
7.53
7.51
7.33
7.32
7.31
7.31
7.31
7.30
7.29
7.29
7.29
7.28
7.28
7.27
7.27
7.26 CDCl3
7.26
7.13
7.13
7.13
7.13
7.13
7.11
7.11
7.11
7.11
7.11
7.03
7.02
6.81
6.80
6.78
6.78
6.74
6.74
6.49
6.49
6.48
6.48
5.27
3.80
O
N
Bn
1f
1H NMR (CDCl3)
0.97
3.28
1.89
0.92
0.94
0.96
0.91
2.00
3.08
11.0
10.5
10.0
9.5
9.0
8.5
8.0
7.5
7.0
6.5
6.0
5.5
5.0
4.5
4.0
3.5
3.0
2.5
2.0
1.5
1.0
0.5
0.0
f1 (ppm)

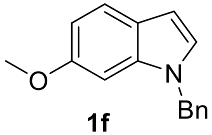

1f

$^{13}C\{^{1}H\}$ NMR ($CDCl_3$)

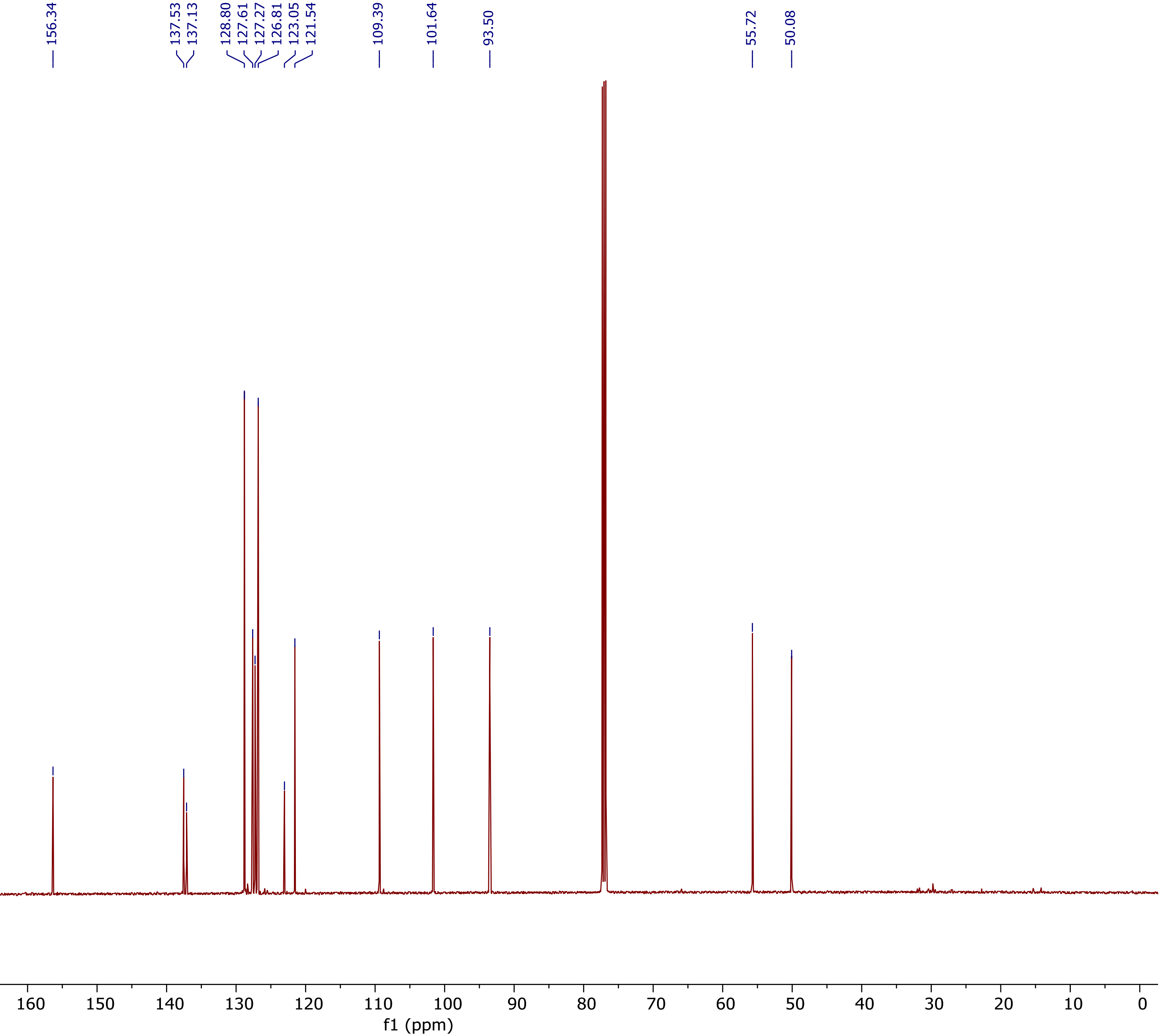

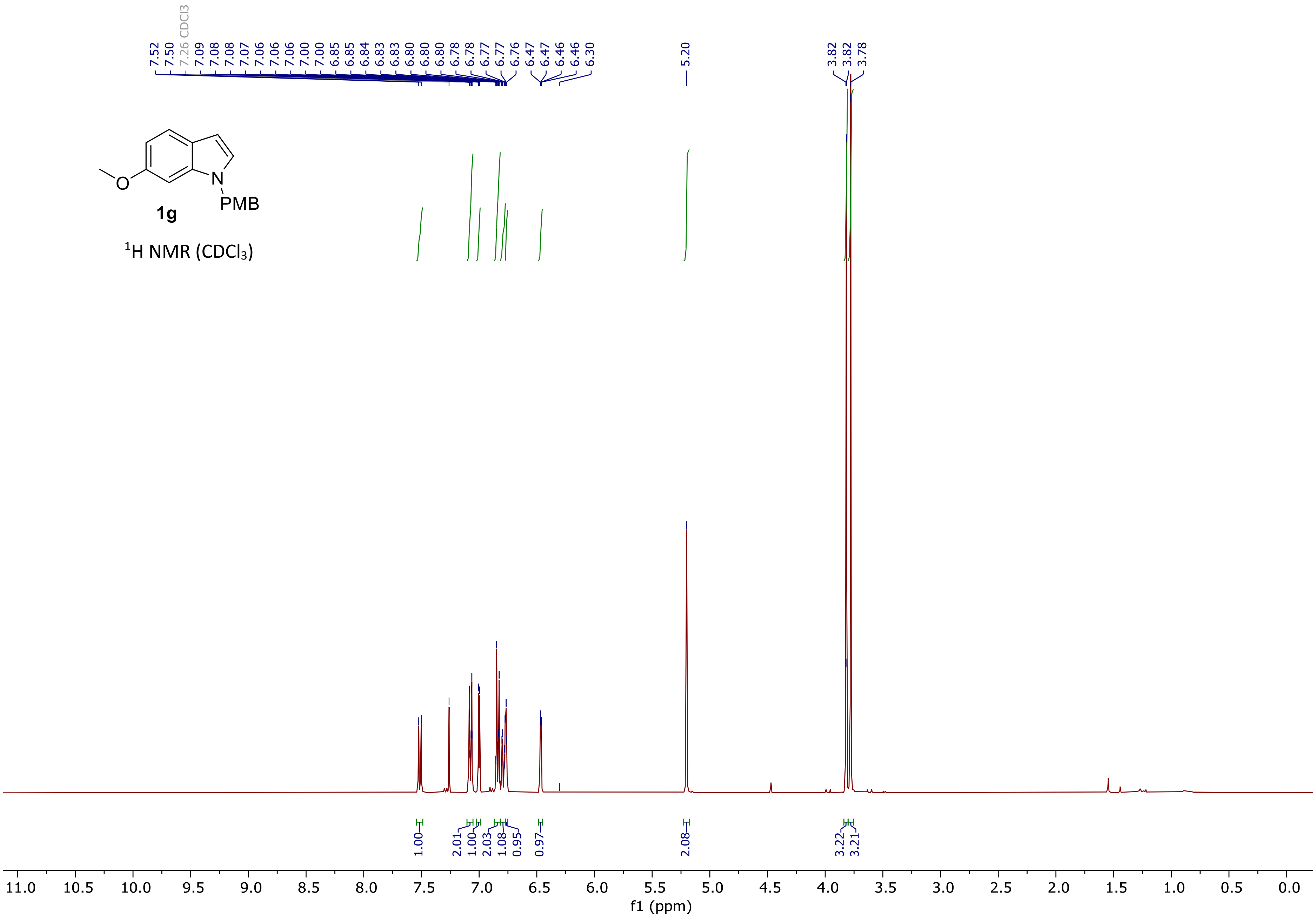
7.52
7.50
7.26 CDCl3
7.09
7.08
7.08
7.07
7.06
7.06
7.06
7.00
7.00
6.85
6.85
6.84
6.83
6.83
6.80
6.80
6.80
6.78
6.78
6.77
6.77
6.76
6.47
6.47
6.46
6.30
5.20
3.82
3.82
3.78
O
N
PMB
1g
^{1}H NMR (CDCl$_3$)
1.00
2.01
1.00
2.03
1.08
0.95
0.97
2.08
3.22
3.21
11.0
10.5
10.0
9.5
9.0
8.5
8.0
7.5
7.0
6.5
6.0
5.5
5.0
4.5
4.0
3.5
3.0
2.5
2.0
1.5
1.0
0.5
0.0
f1 (ppm)

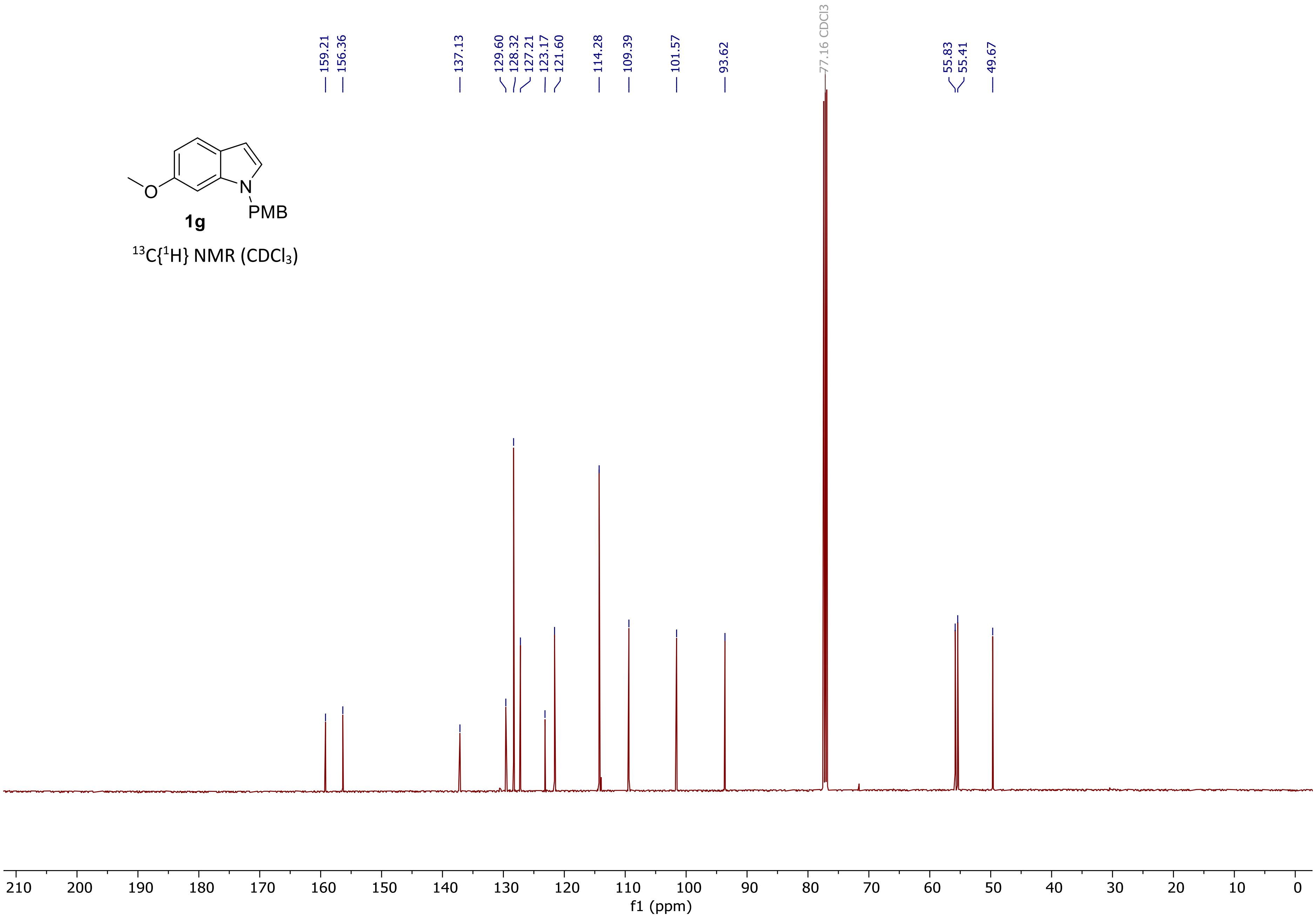

$^{13}C\{^{1}H\}$ NMR ($CDCl_3$)

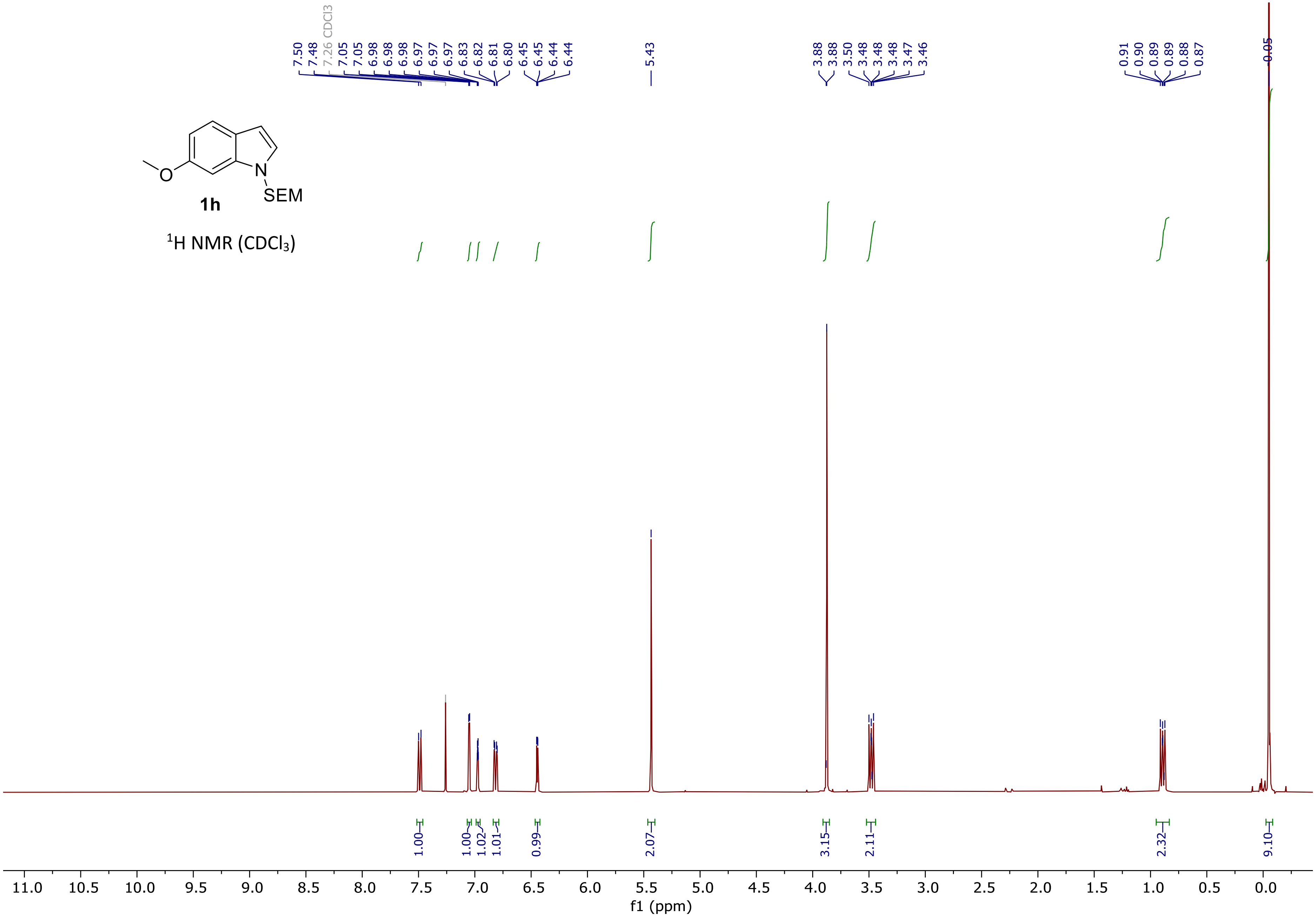
7.50
7.48
7.26 CDCl3
7.05
7.05
6.98
6.98
6.98
6.97
6.97
6.97
6.83
6.82
6.81
6.80
6.45
6.45
6.44
6.44
5.43
3.88
3.88
3.50
3.48
3.48
3.48
3.47
3.46
0.91
0.90
0.89
0.89
0.88
0.87
-0.05
O
N
SEM
1h
1H NMR (CDCl3)
1.00
1.00
1.02
1.01
0.99
2.07
3.15
2.11
2.32
9.10
11.0
10.5
10.0
9.5
9.0
8.5
8.0
7.5
7.0
6.5
6.0
5.5
5.0
4.5
4.0
3.5
3.0
2.5
2.0
1.5
1.0
0.5
0.0
f1 (ppm)

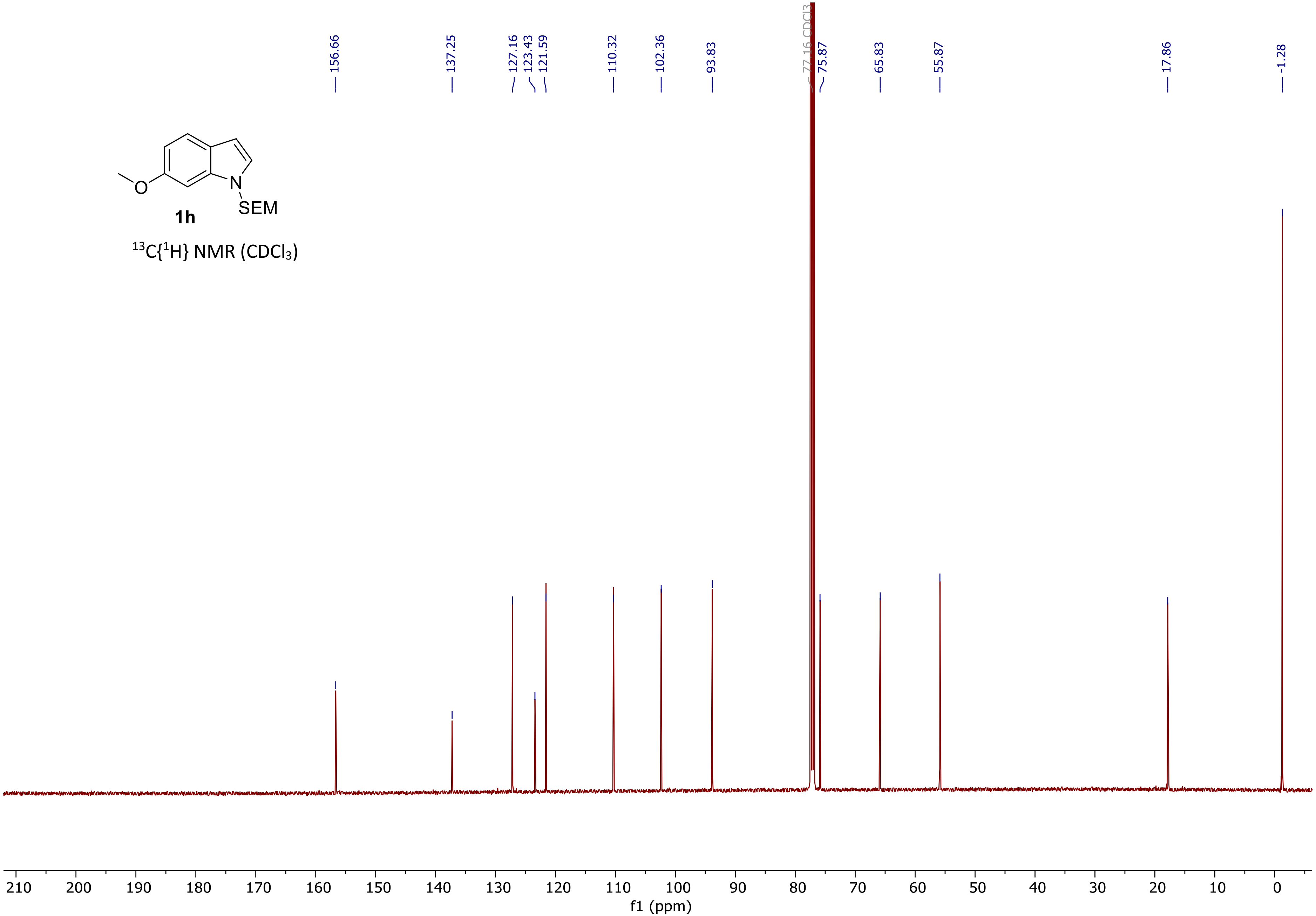
1h
SEM
$^{13}C\{^1H\}$ NMR ($CDCl_3$)
156.66
137.25
127.16
123.43
121.59
110.32
102.36
93.83
77.16 CDCl3
75.87
65.83
55.87
17.86
-1.28
f1 (ppm)

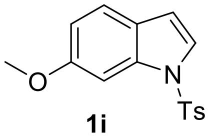

1i

^{1}H NMR ($CDCl_3$)

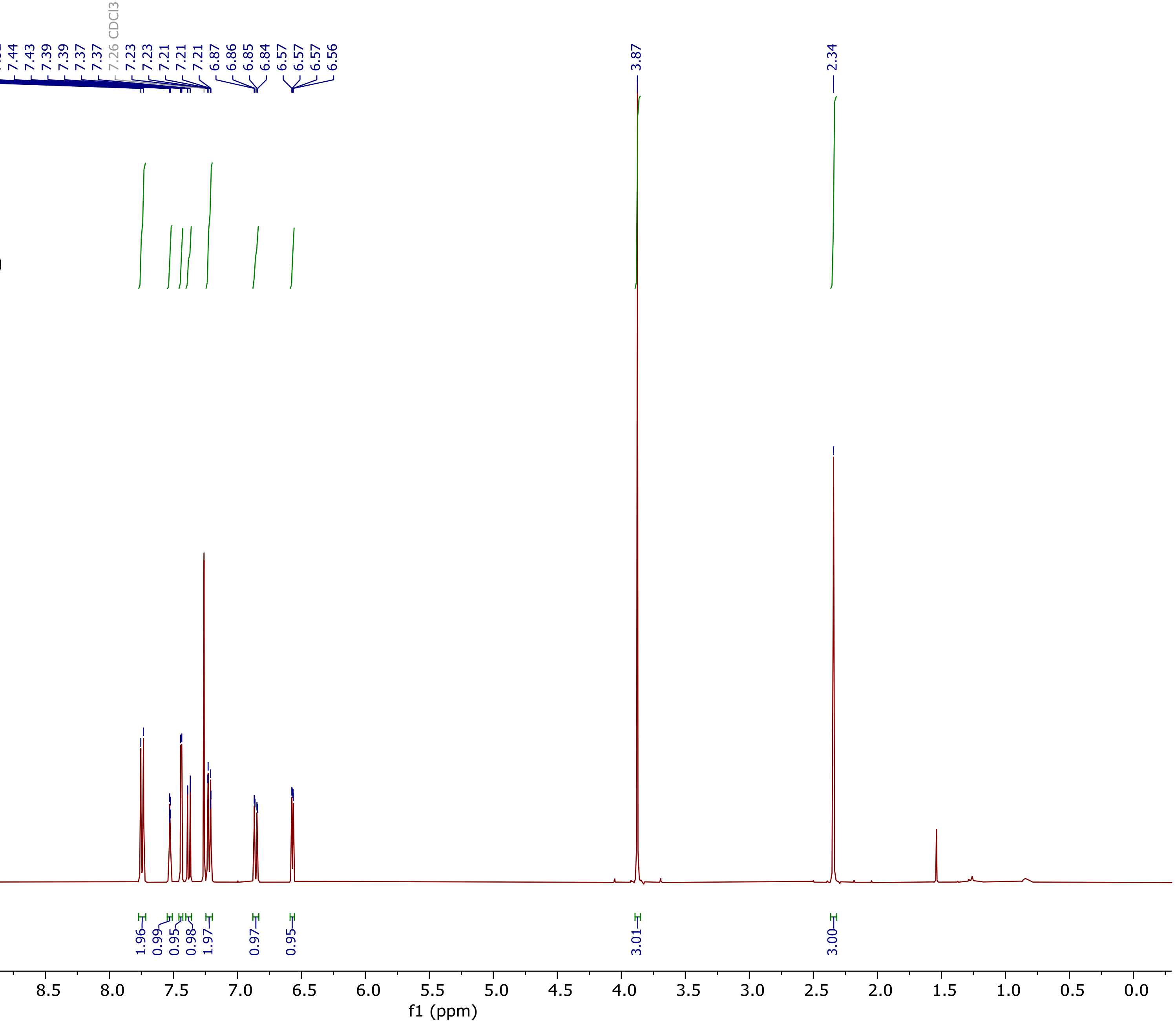

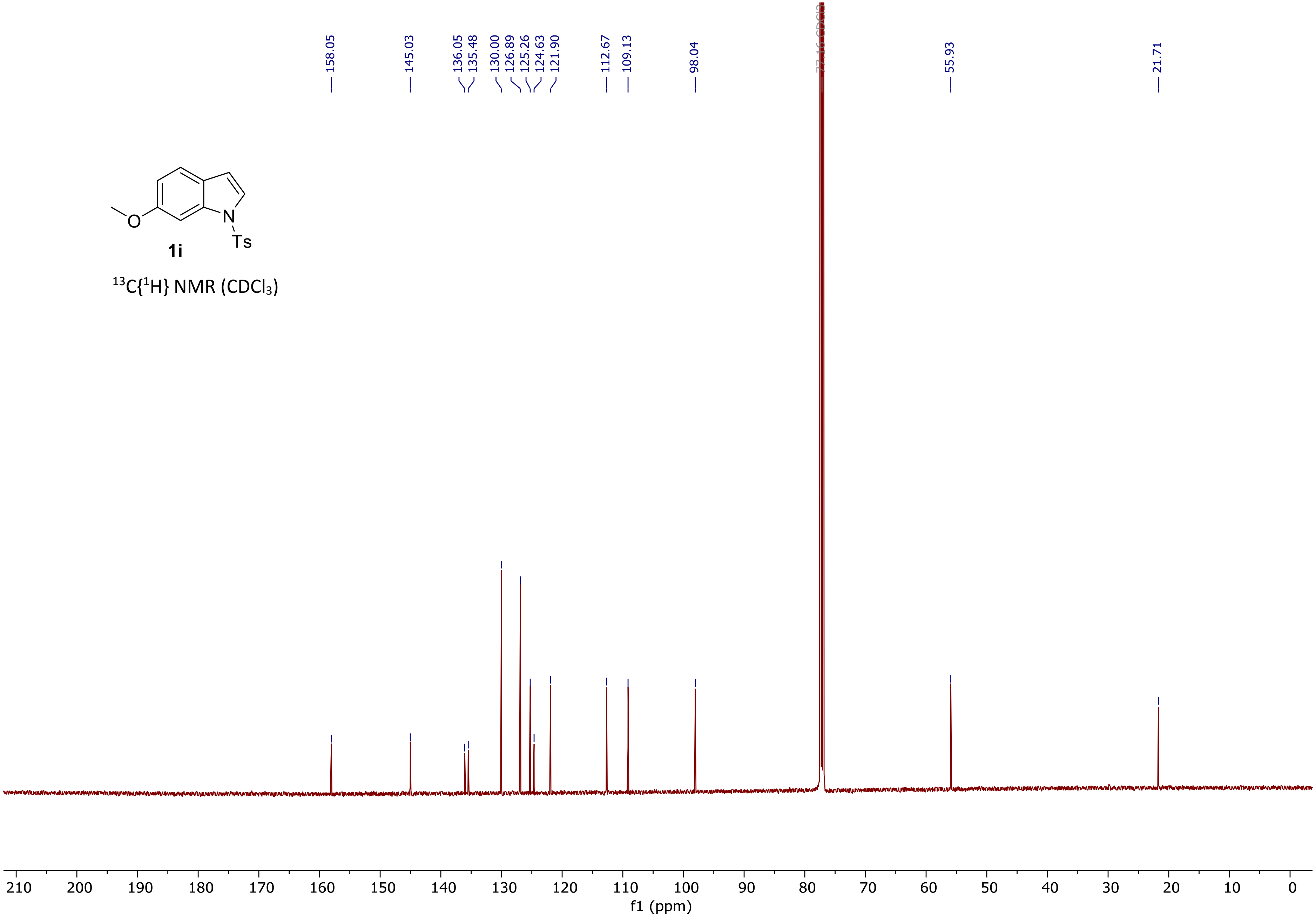

1i

$^{13}C\{^{1}H\}$ NMR ($CDCl_3$)

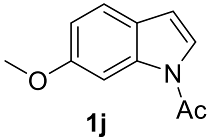

1j

^{1}H NMR ($CDCl_3$)

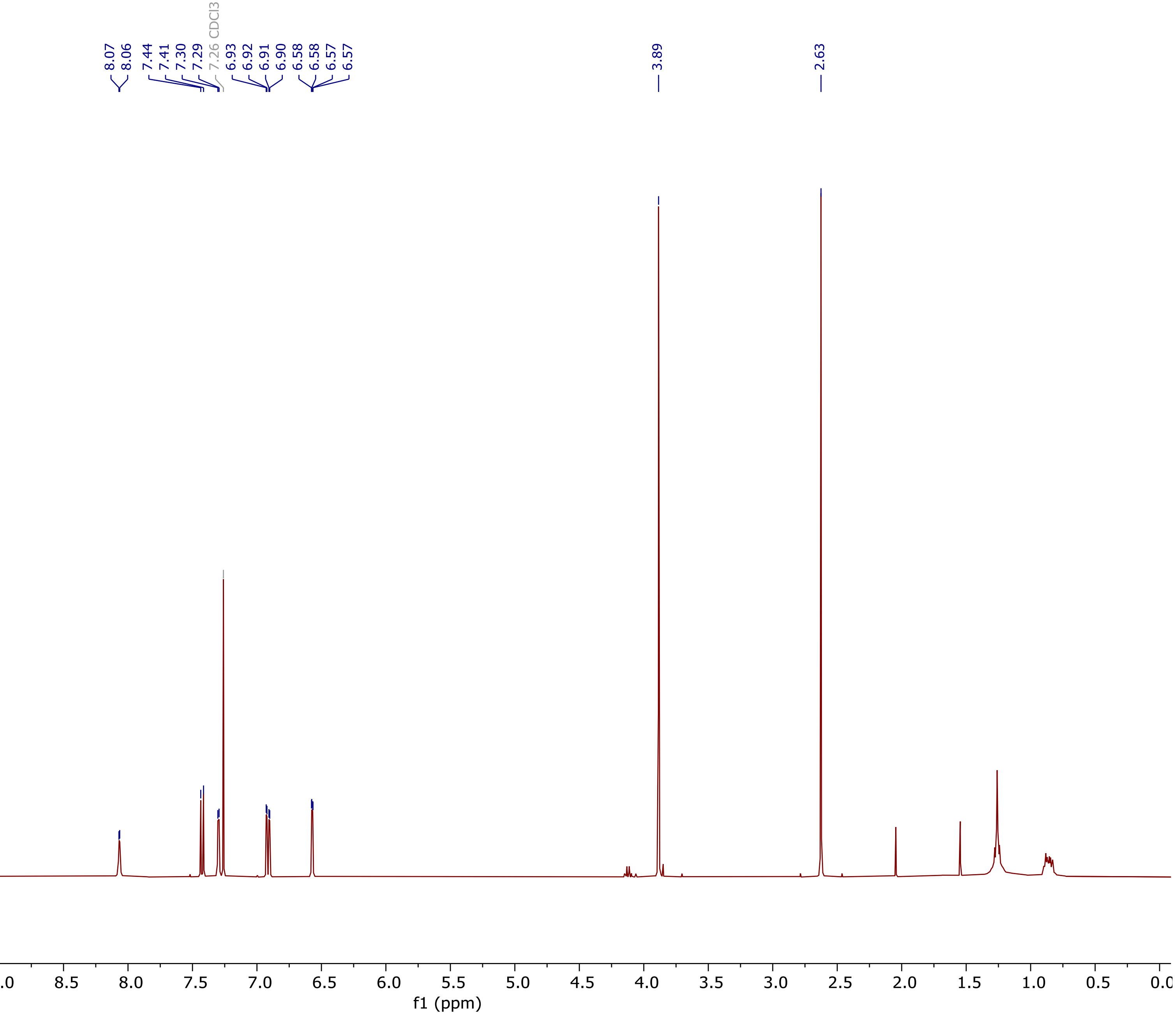

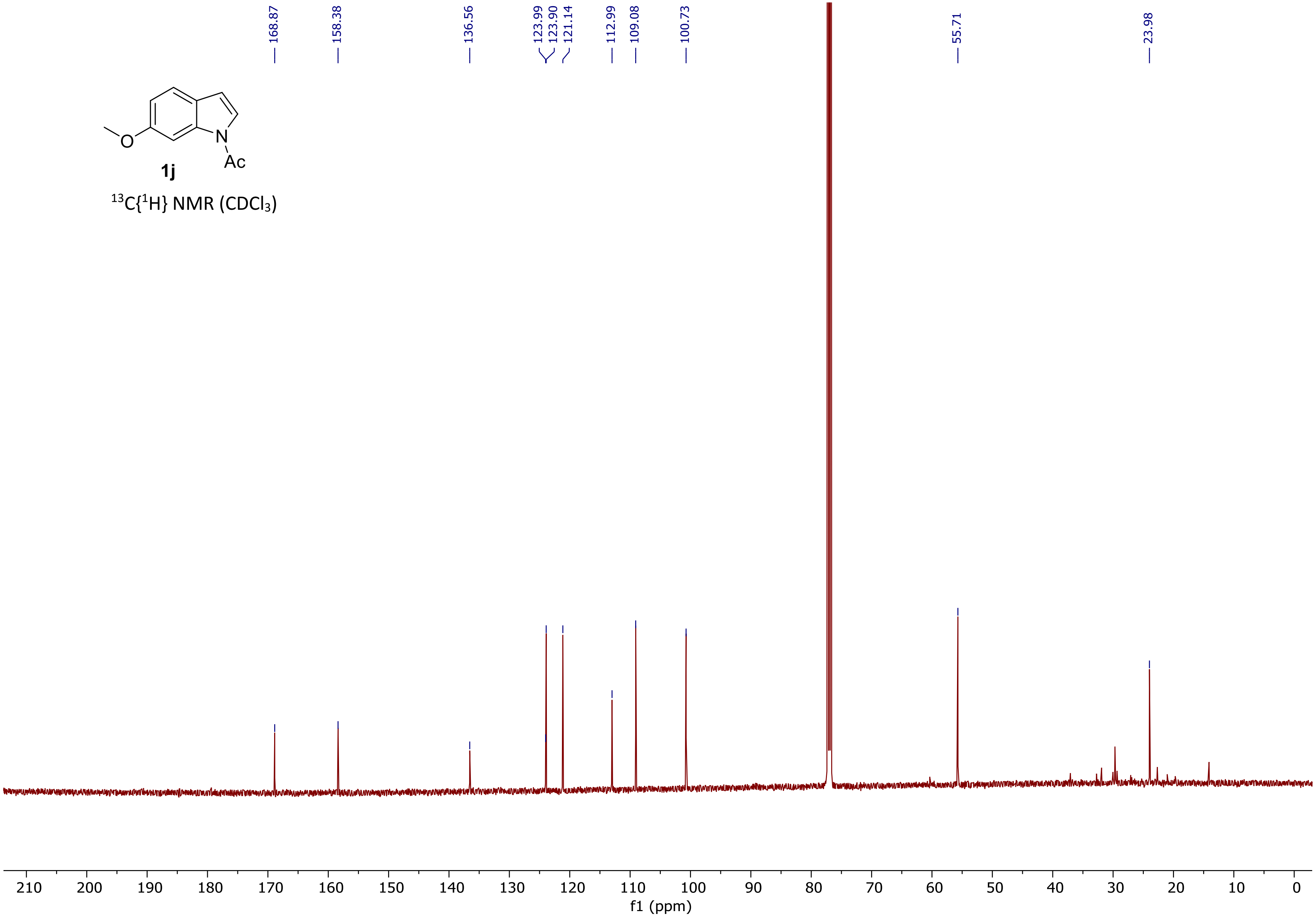
168.87
158.38
136.56
123.99
123.90
121.14
112.99
109.08
100.73
55.71
23.98
Ac
N
O
1j
$^{13}C\{^{1}H\}$ NMR ($CDCl_3$)
210 200 190 180 170 160 150 140 130 120 110 100 90 80 70 60 50 40 30 20 10 0
f1 (ppm)

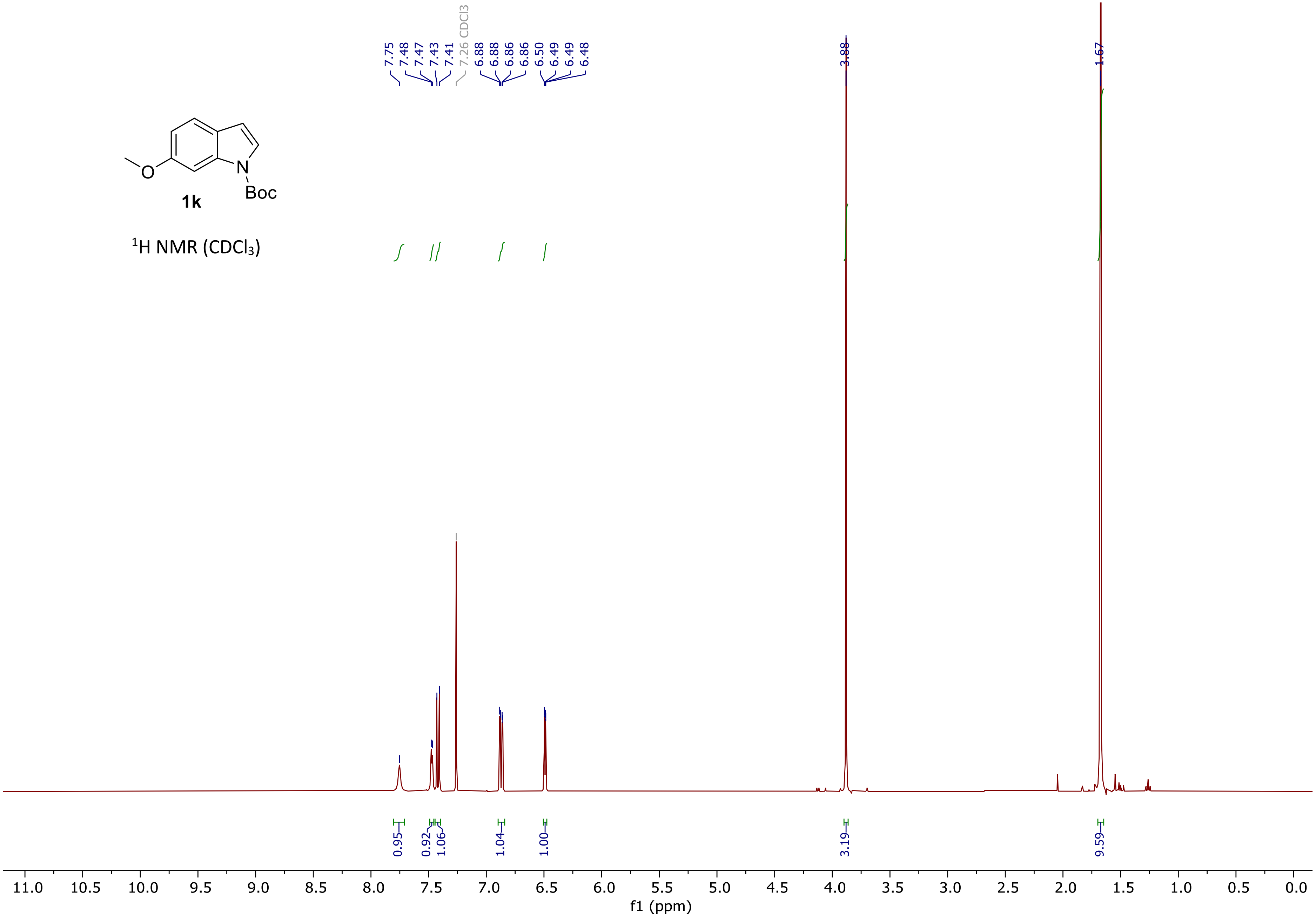

1k
Boc
N
O
^{1}H NMR ($CDCl_3$)
7.75
7.48
7.47
7.43
7.41
7.26 CDCl3
6.88
6.88
6.86
6.86
6.50
6.49
6.49
6.48
3.88
1.67
0.95
0.92
1.06
1.04
1.00
3.19
9.59
11.0 10.5 10.0 9.5 9.0 8.5 8.0 7.5 7.0 6.5 6.0 5.5 5.0 4.5 4.0 3.5 3.0 2.5 2.0 1.5 1.0 0.5 0.0
f1 (ppm)

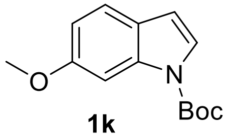

1k

$^{13}C\{^{1}H\}$ NMR ($CDCl_3$)

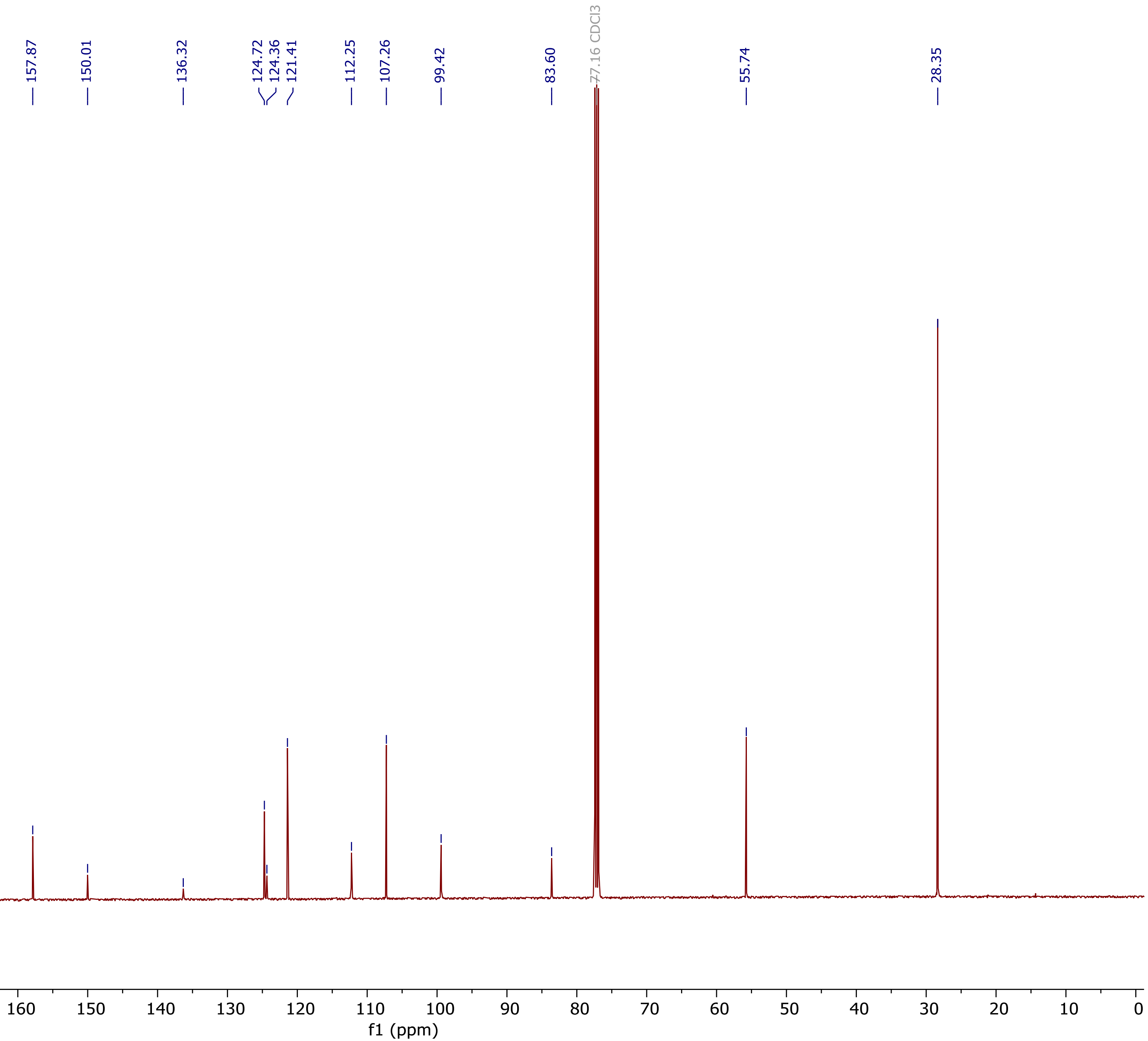

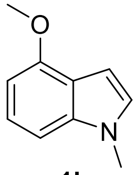

1l

^{1}H NMR ($CDCl_3$)

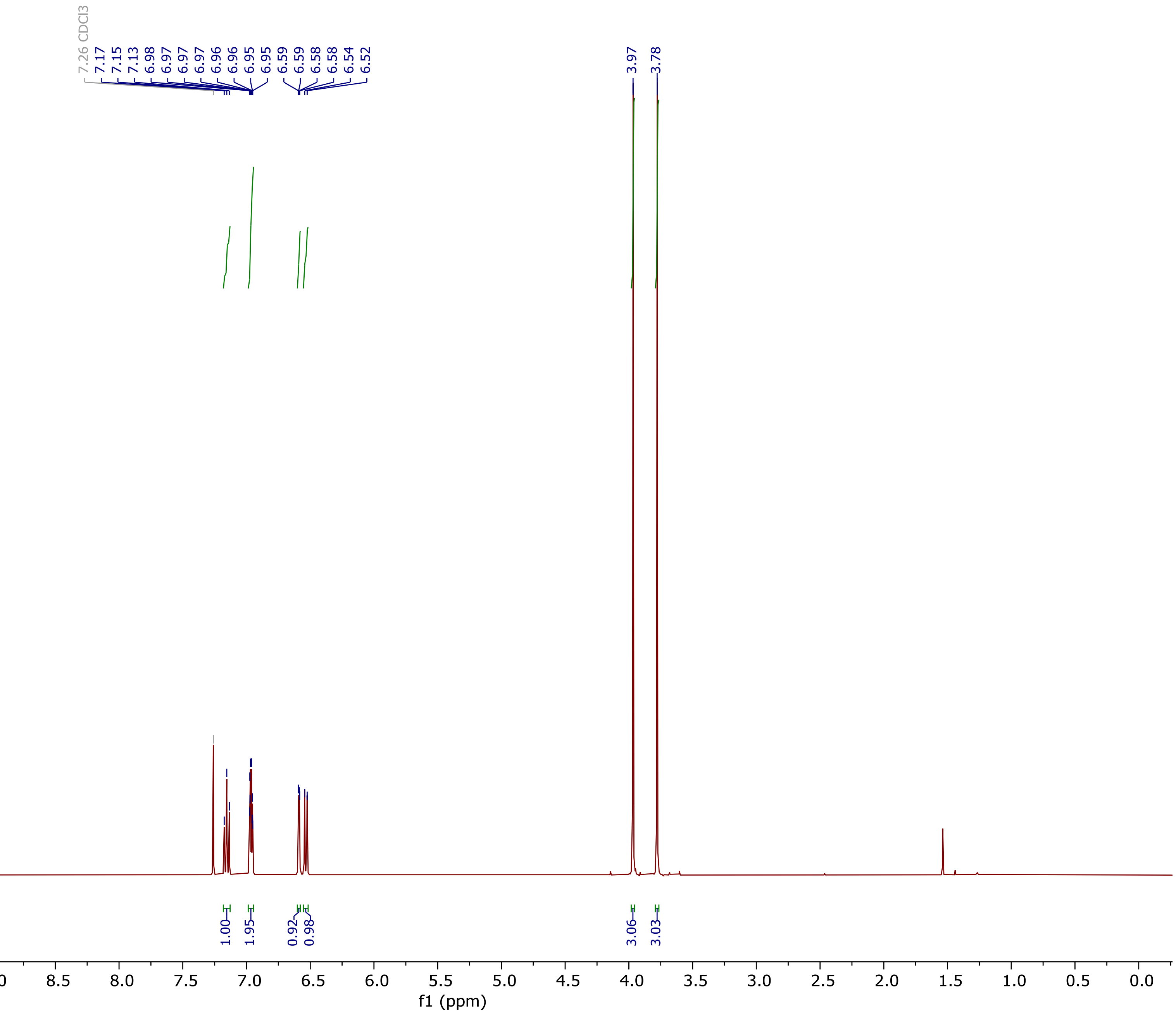

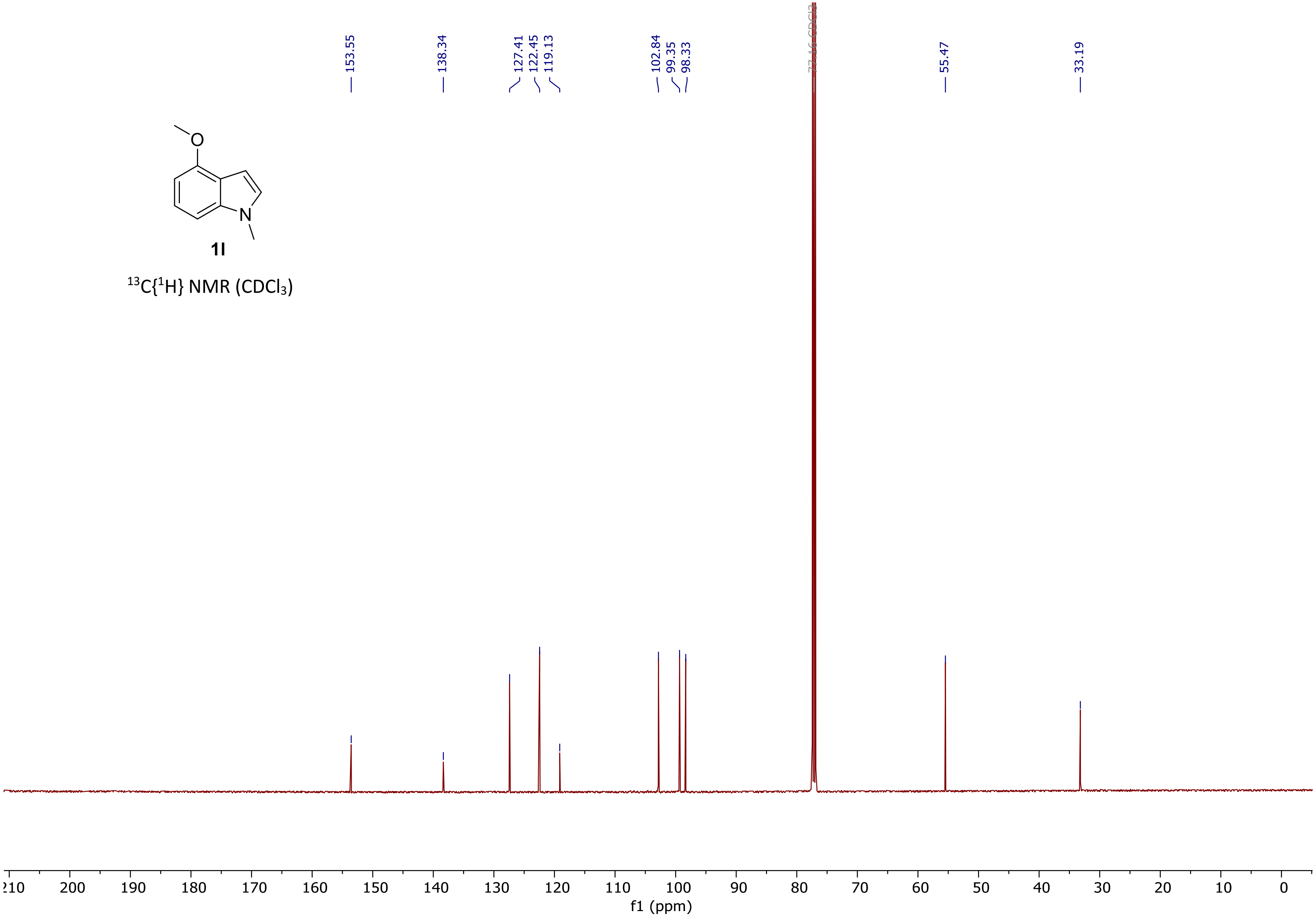

153.55
138.34
127.41
122.45
119.13
102.84
99.35
98.33
77.16 CDCl3
55.47
33.19
1l
13C{1H} NMR (CDCl3)
210
200
190
180
170
160
150
140
130
120
110
100
90
80
70
60
50
40
30
20
10
0
f1 (ppm)

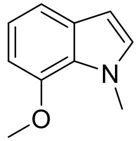

1n

^{1}H NMR ($CDCl_3$)

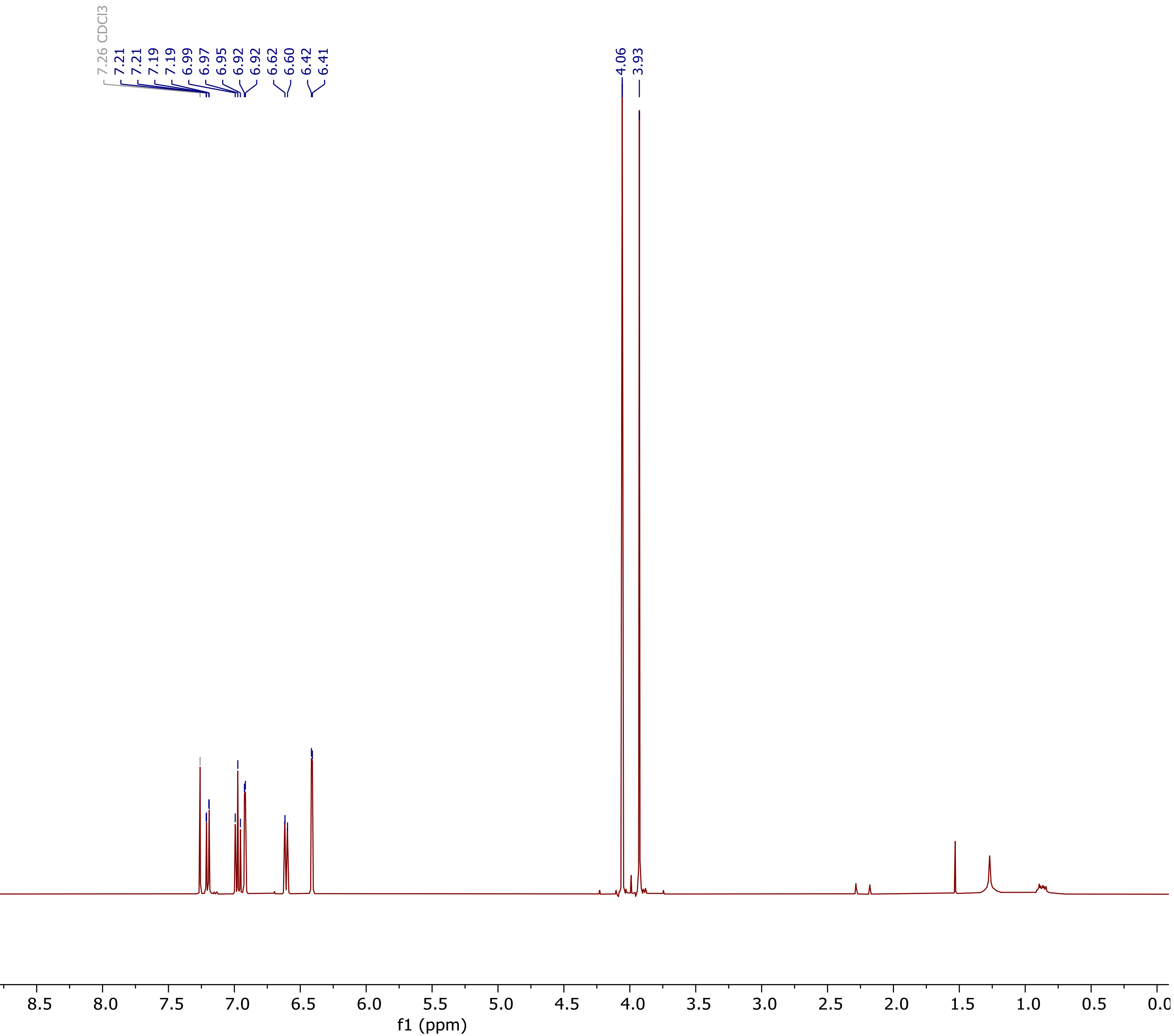

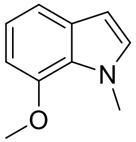

1n

$^{13}C\{^{1}H\}$ NMR ($CDCl_3$)

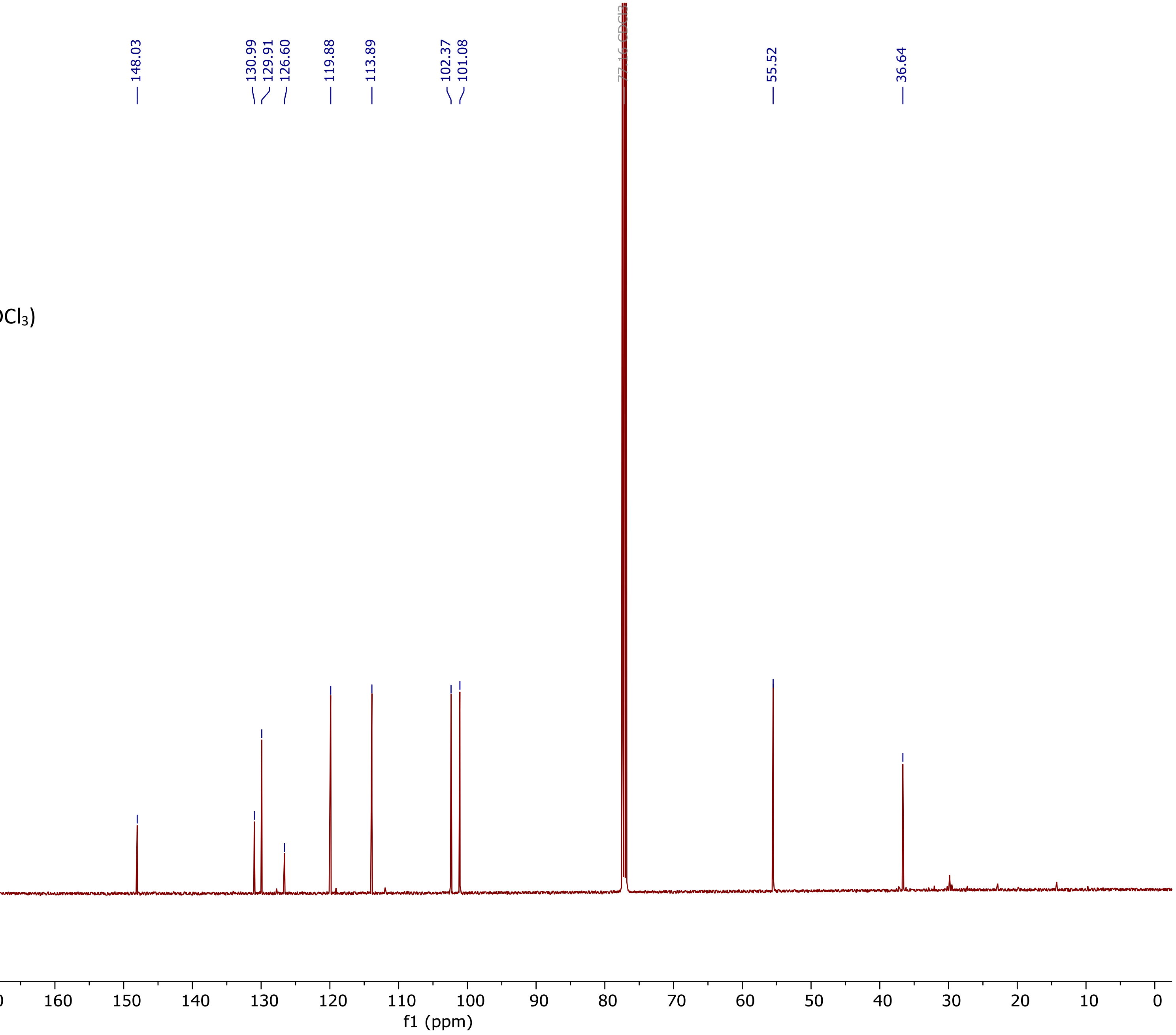

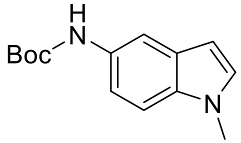

1o

^{1}H NMR ($CDCl_3$)

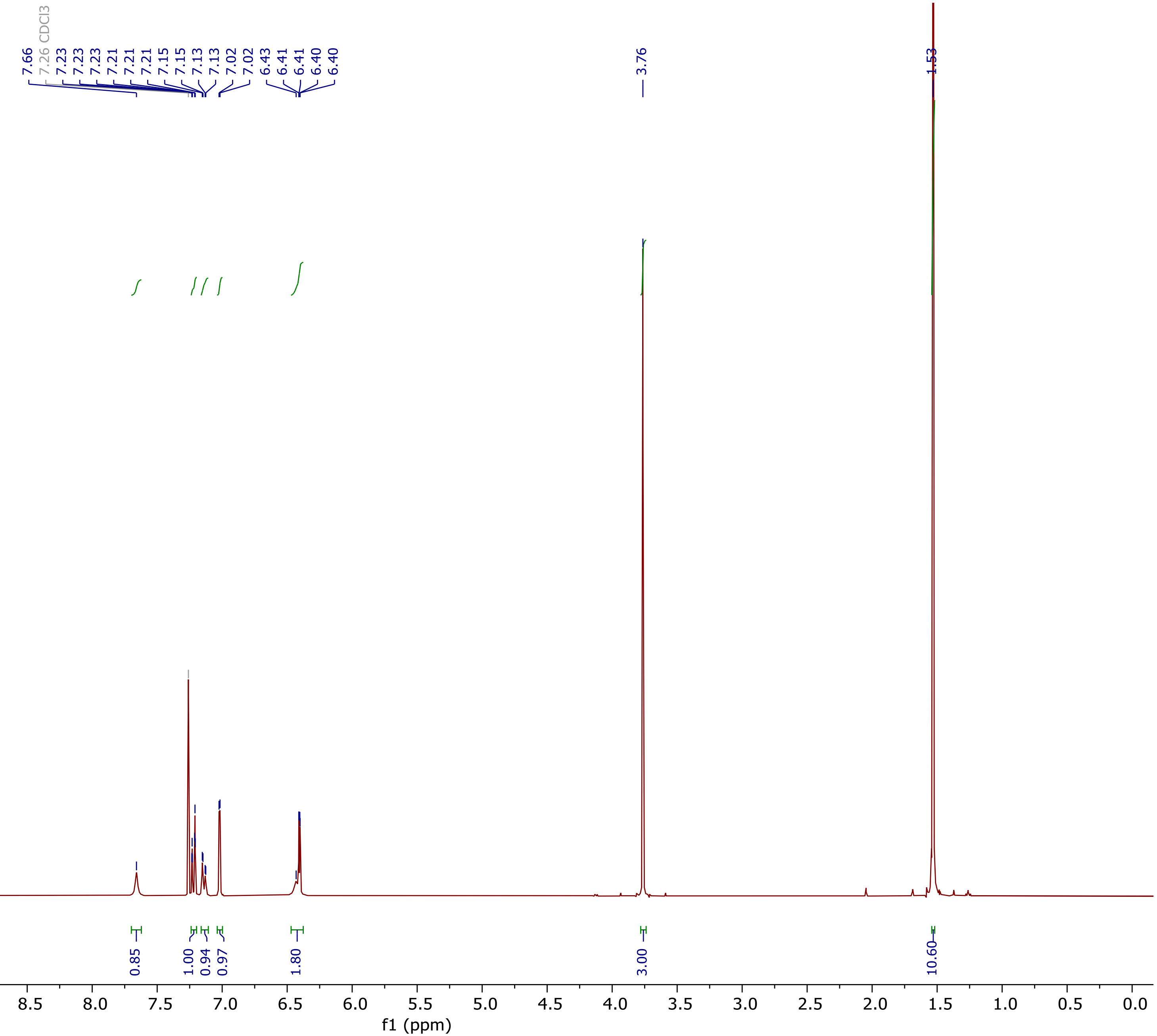

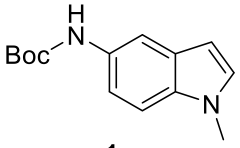

1o

$^{13}C\{^{1}H\}$ NMR ($CDCl_3$)

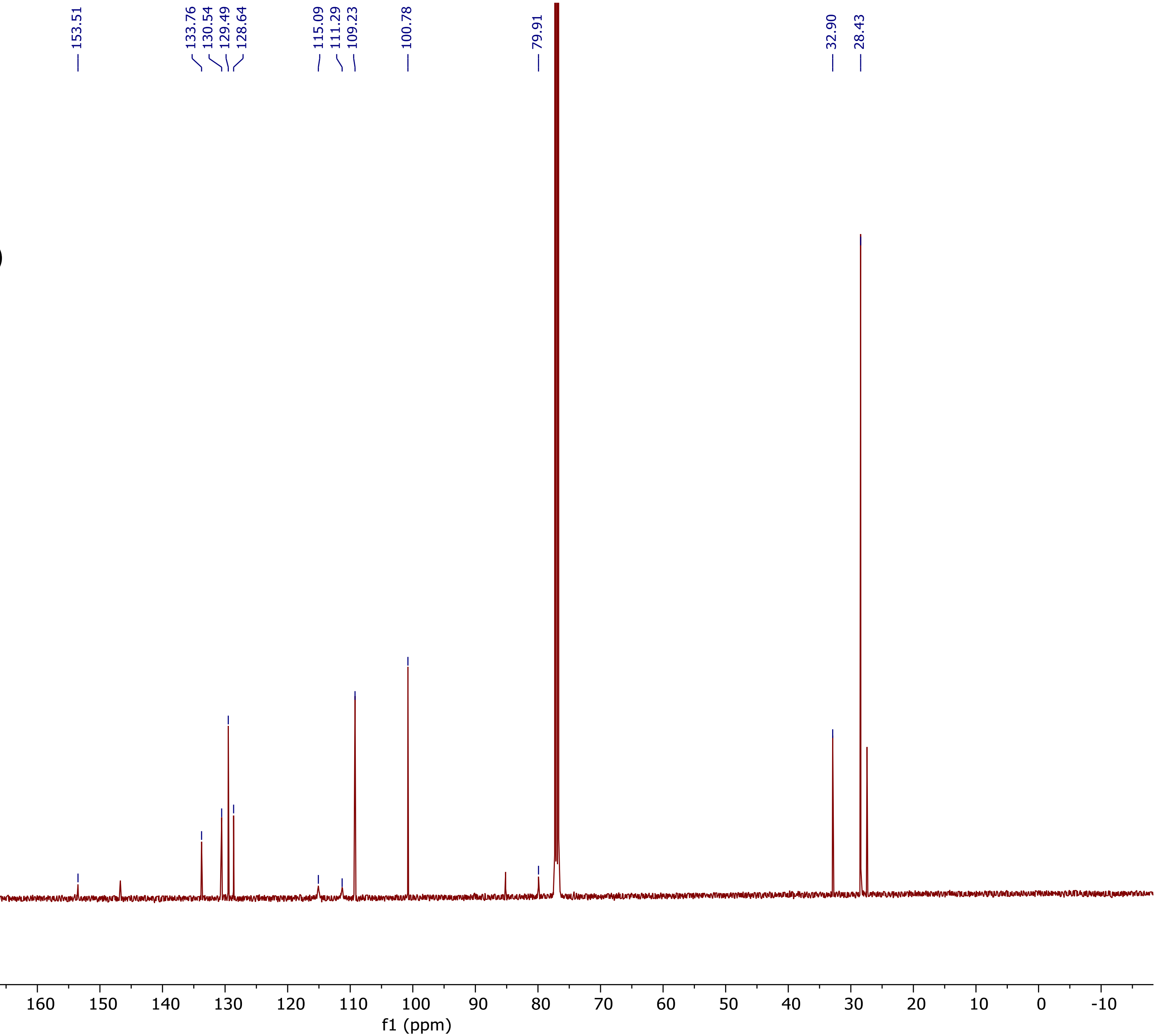

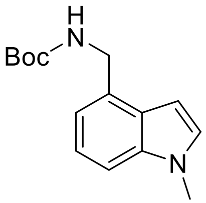

1p

^1H NMR ($CDCl_3$)

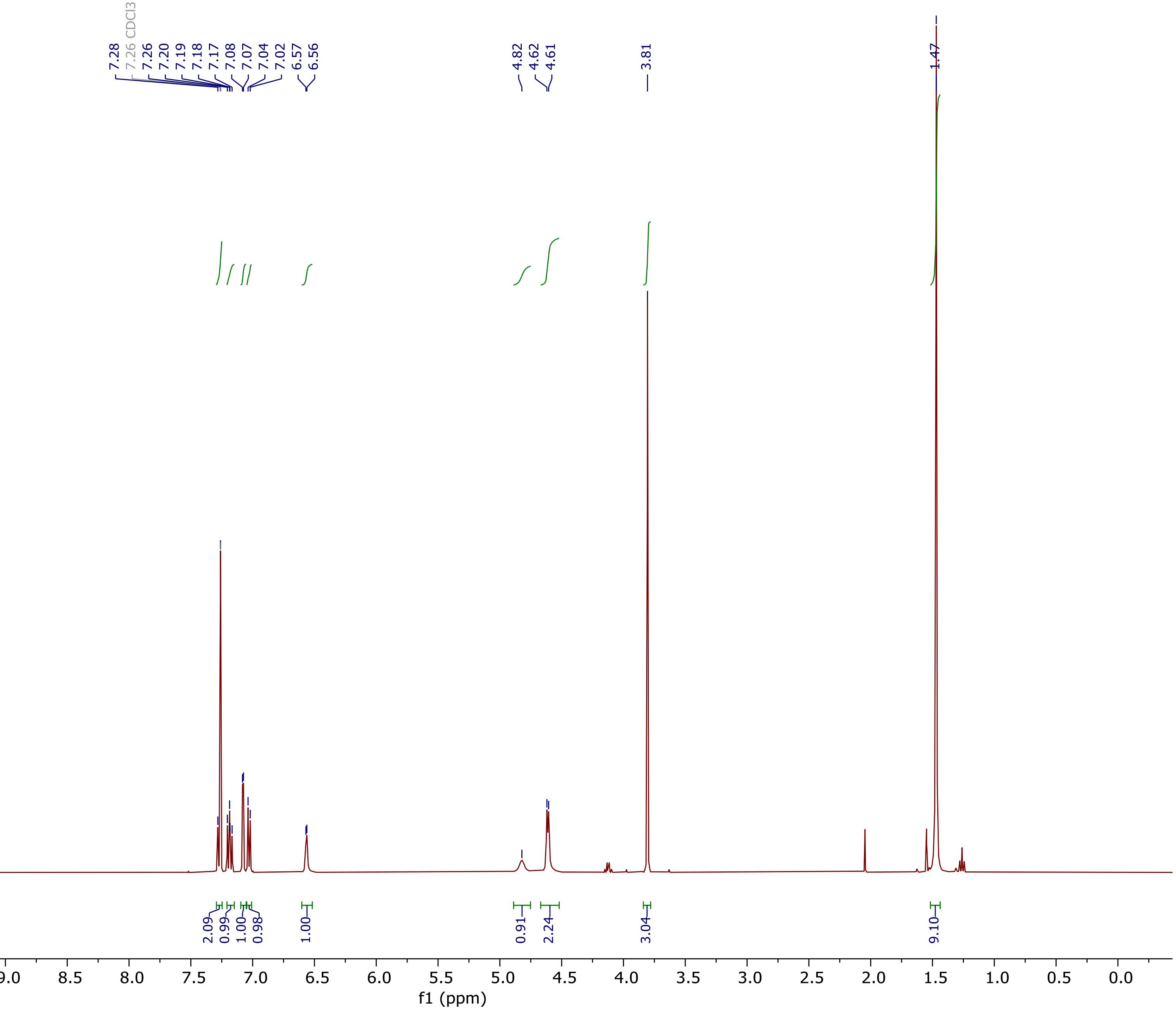

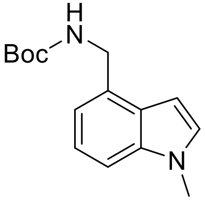

1p

$^{13}C\{^{1}H\}$ NMR ($CDCl_3$)

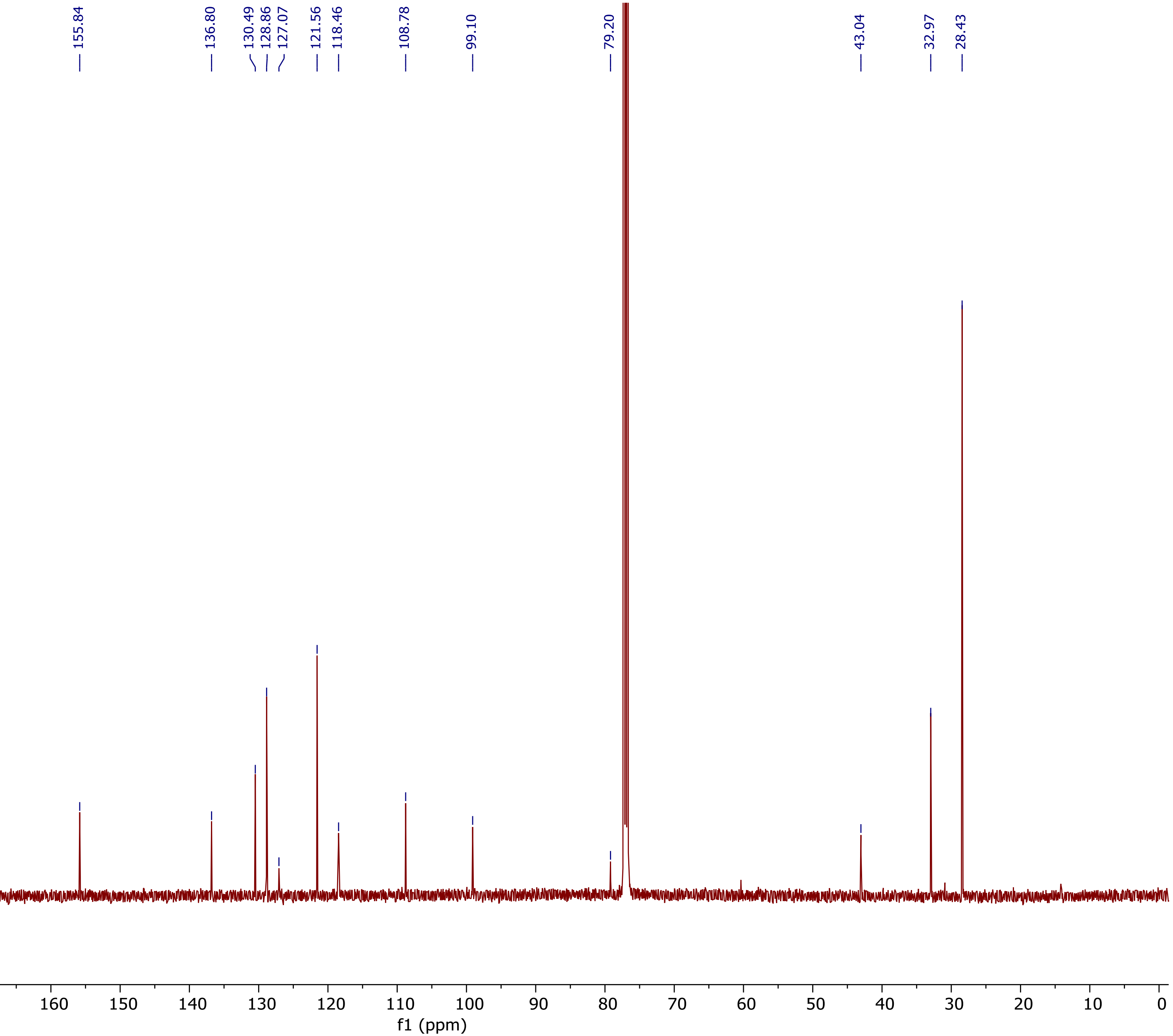

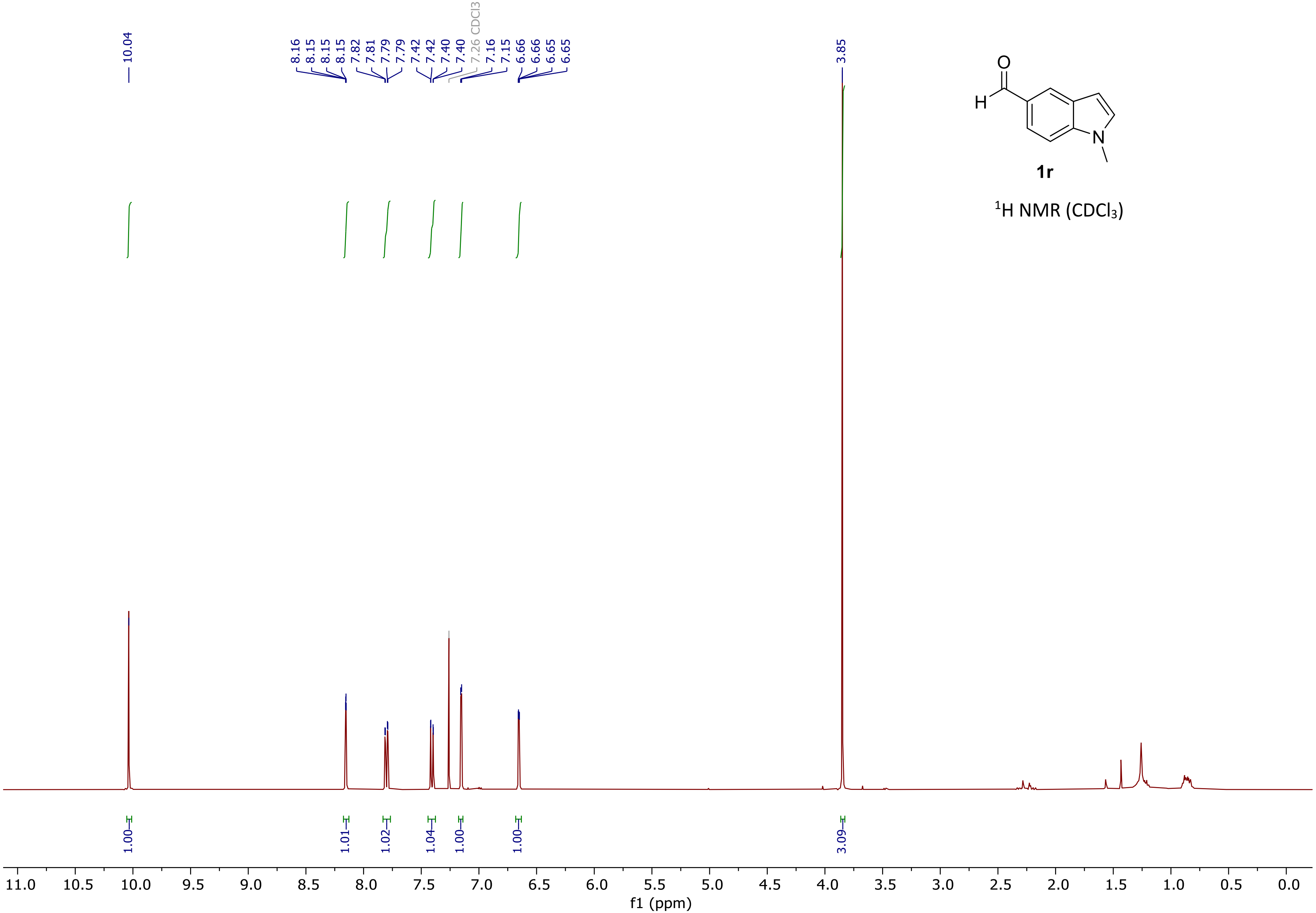

1r

^{1}H NMR ($CDCl_3$)

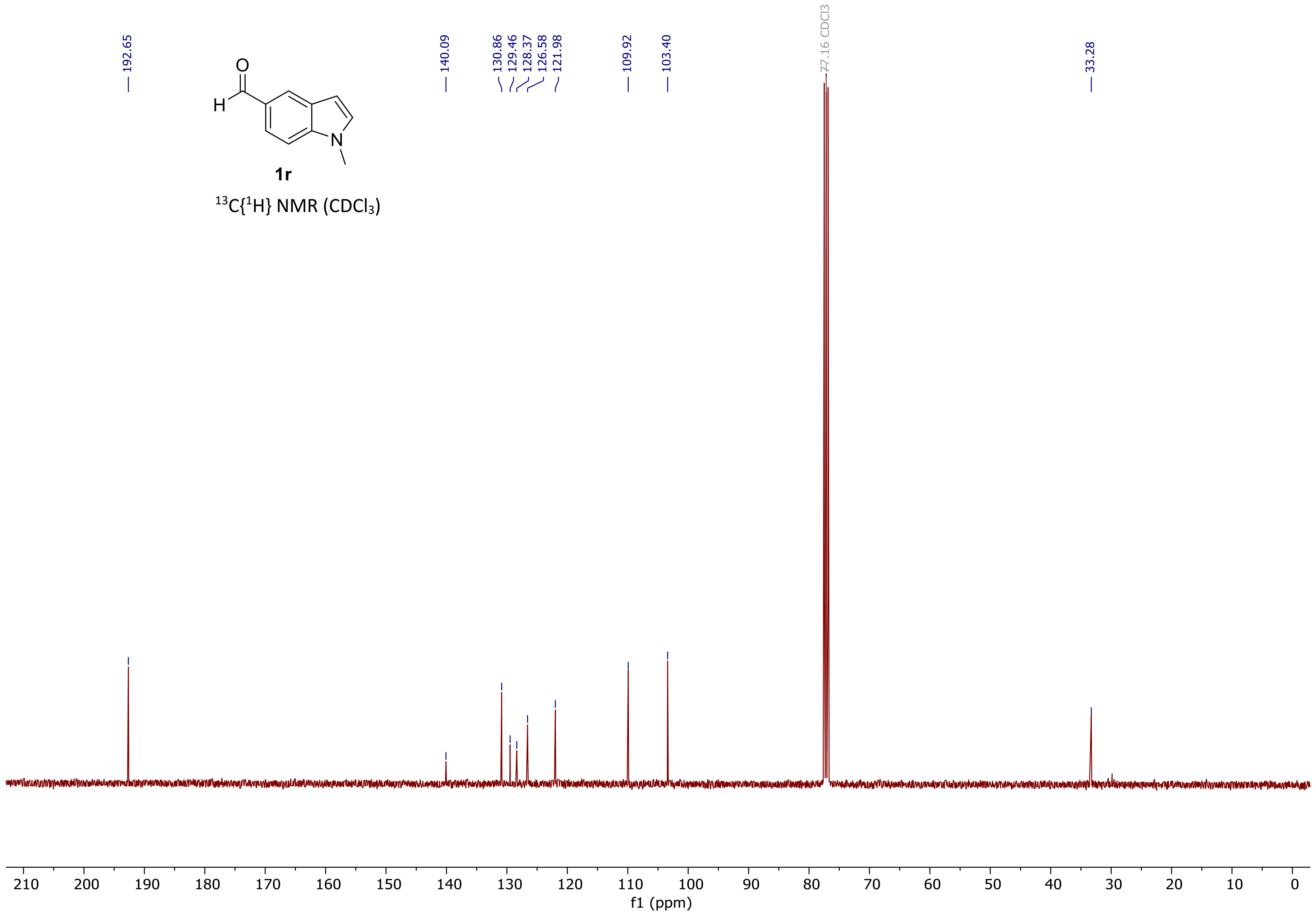
192.65
140.09
130.86
129.46
128.37
126.58
121.98
109.92
103.40
77.16 CDCl3
33.28
O
H
N
1r
13C{1H} NMR (CDCl3)
210 200 190 180 170 160 150 140 130 120 110 100 90 80 70 60 50 40 30 20 10 0
f1 (ppm)

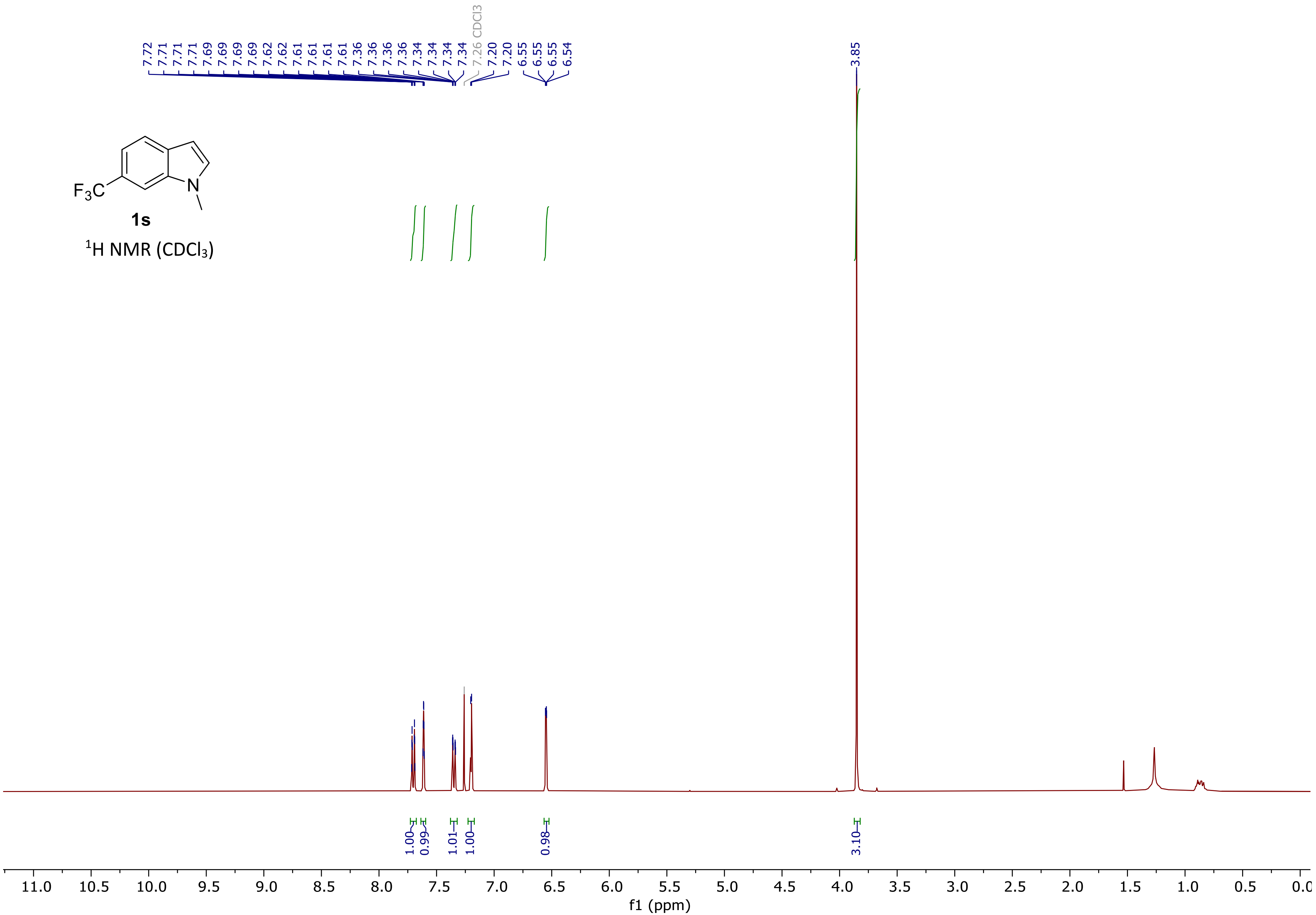

1s

^{1}H NMR ($CDCl_3$)

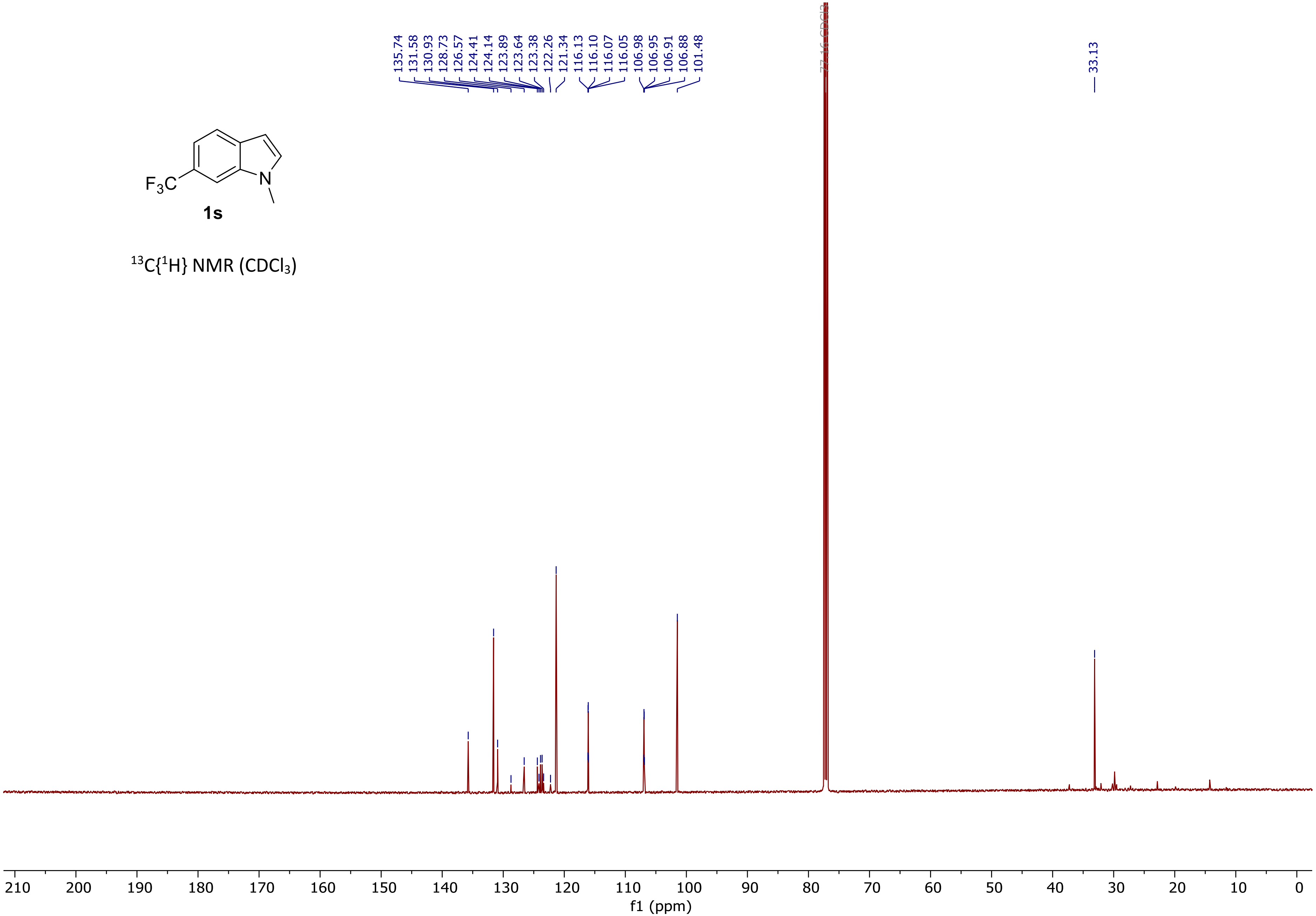
135.74
131.58
130.93
128.73
126.57
124.41
124.14
123.89
123.64
123.38
122.26
121.34
116.13
116.10
116.07
116.05
106.98
106.95
106.91
106.88
101.48
77.16 CDCl3
33.13
F3C
N
1s
13C{1H} NMR (CDCl3)
210
200
190
180
170
160
150
140
130
120
110
100
90
80
70
60
50
40
30
20
10
0
f1 (ppm)

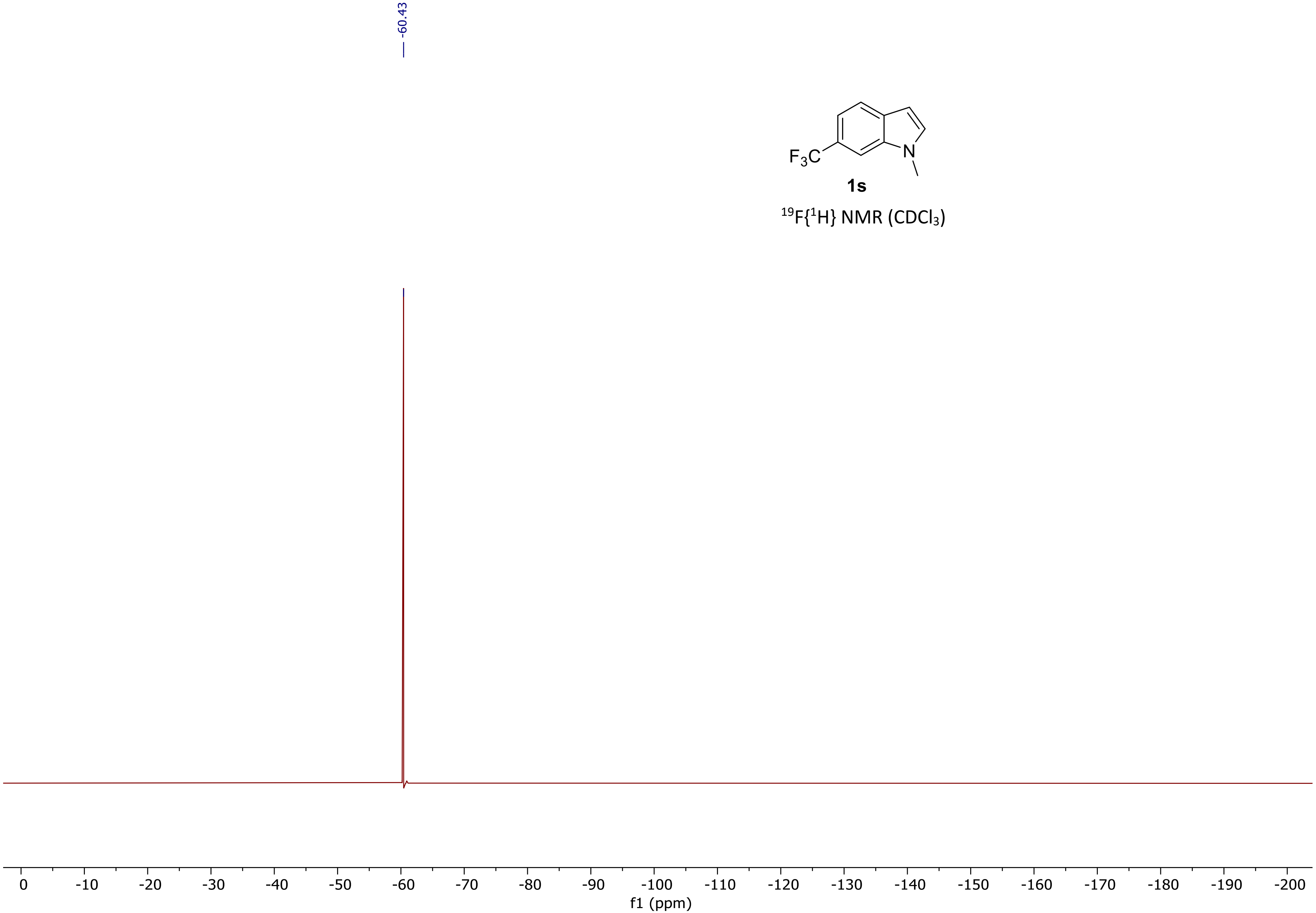

-60.43
F3C
N
1s
19F{1H} NMR (CDCl3)
0
-10
-20
-30
-40
-50
-60
-70
-80
-90
-100
-110
-120
-130
-140
-150
-160
-170
-180
-190
-200
f1 (ppm)

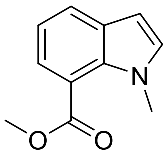

1t

^{1}H NMR ($CDCl_3$)

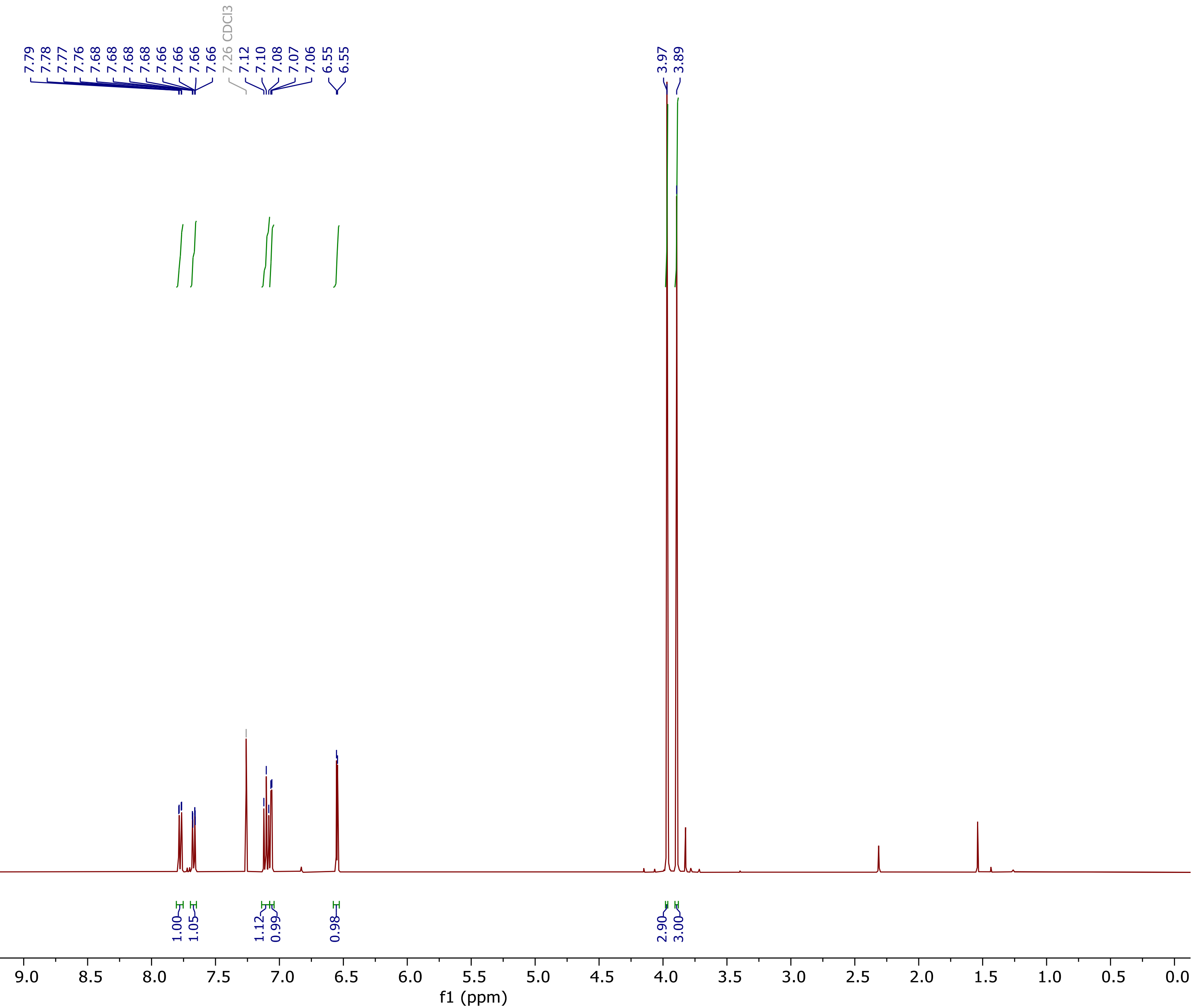

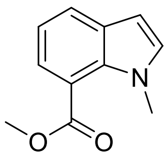

1t

$^{13}C\{^1H\}$ NMR ($CDCl_3$)

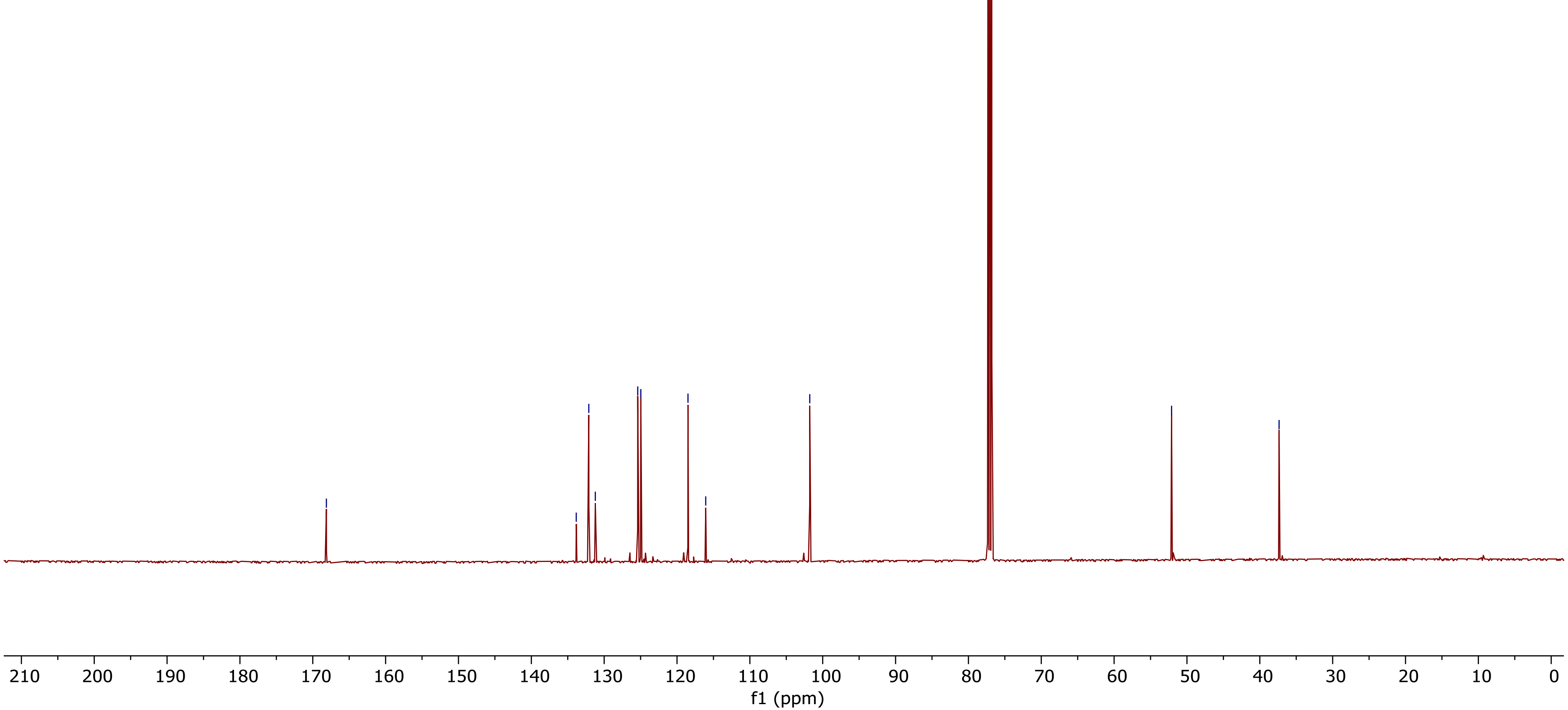

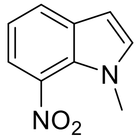

1u

^1H NMR ($CDCl_3$)

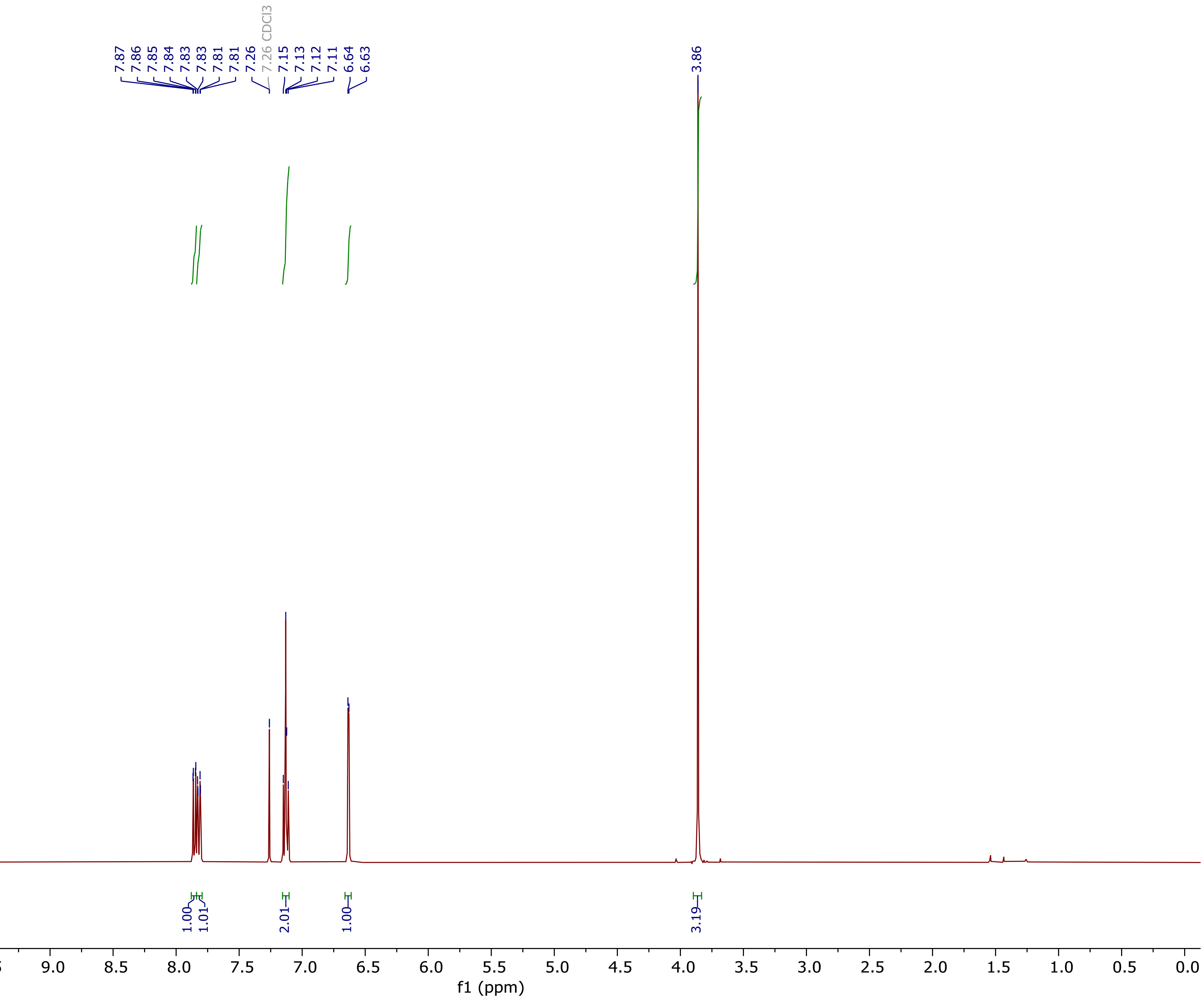

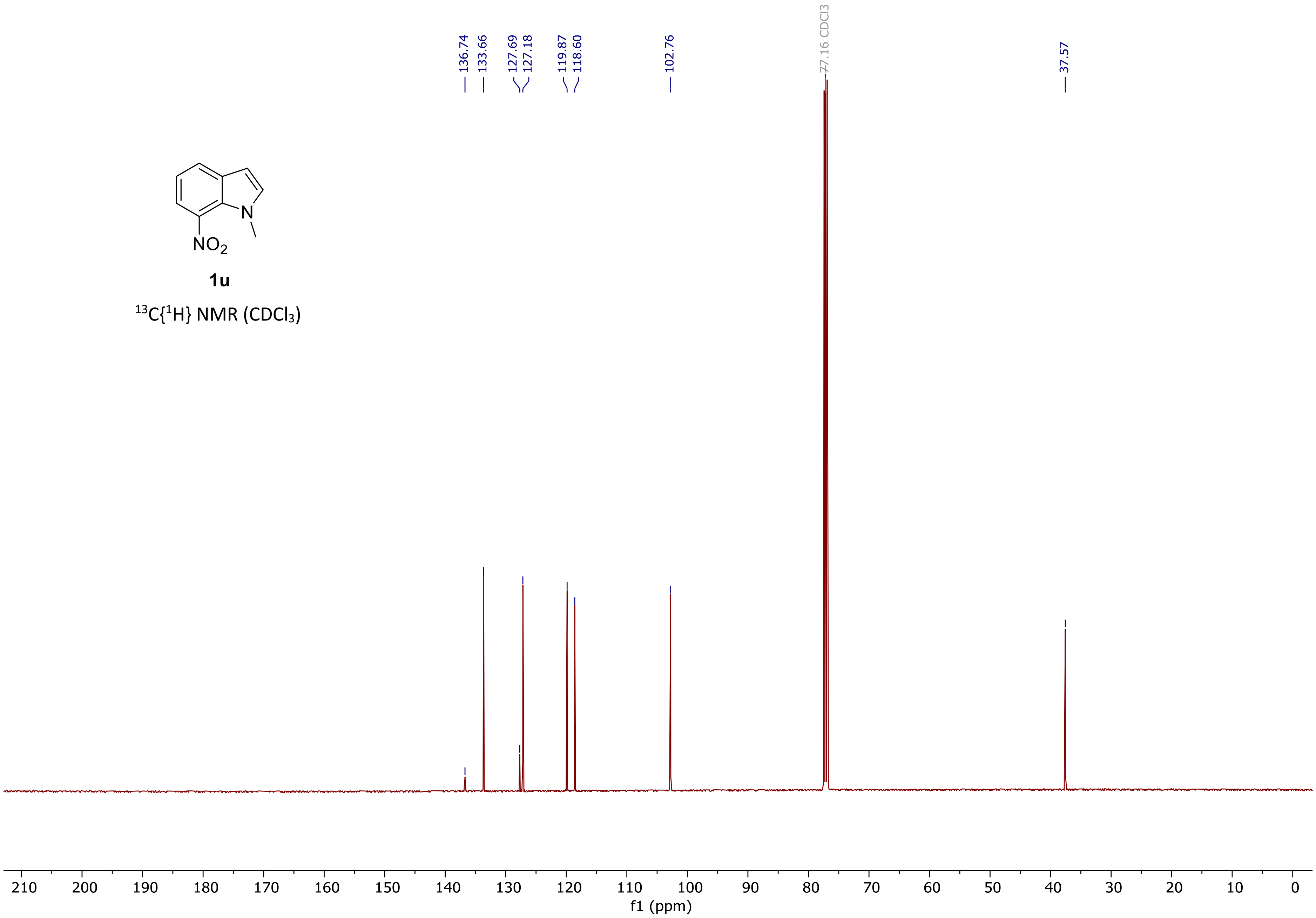

1u

$^{13}C\{^{1}H\}$ NMR ($CDCl_3$)

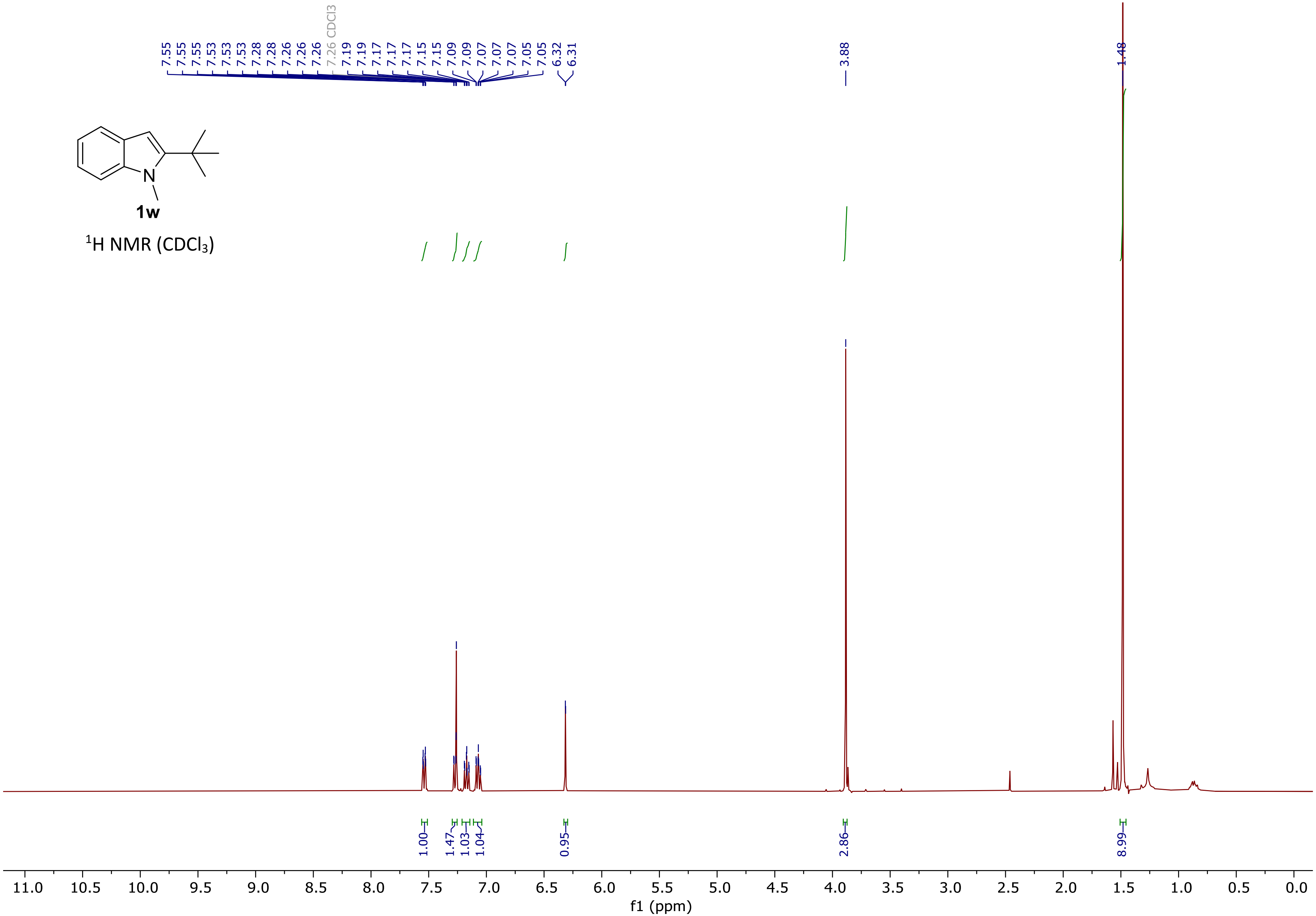

7.55
7.55
7.55
7.53
7.53
7.53
7.28
7.28
7.26
7.26
7.26
7.26 CDCl3
7.19
7.19
7.17
7.17
7.17
7.15
7.15
7.09
7.09
7.07
7.07
7.07
7.05
7.05
6.32
6.31
3.88
1.48
1w
^{1}H NMR ($CDCl_3$)
1.00
1.47
1.03
1.04
0.95
2.86
8.99
11.0
10.5
10.0
9.5
9.0
8.5
8.0
7.5
7.0
6.5
6.0
5.5
5.0
4.5
4.0
3.5
3.0
2.5
2.0
1.5
1.0
0.5
0.0
f1 (ppm)

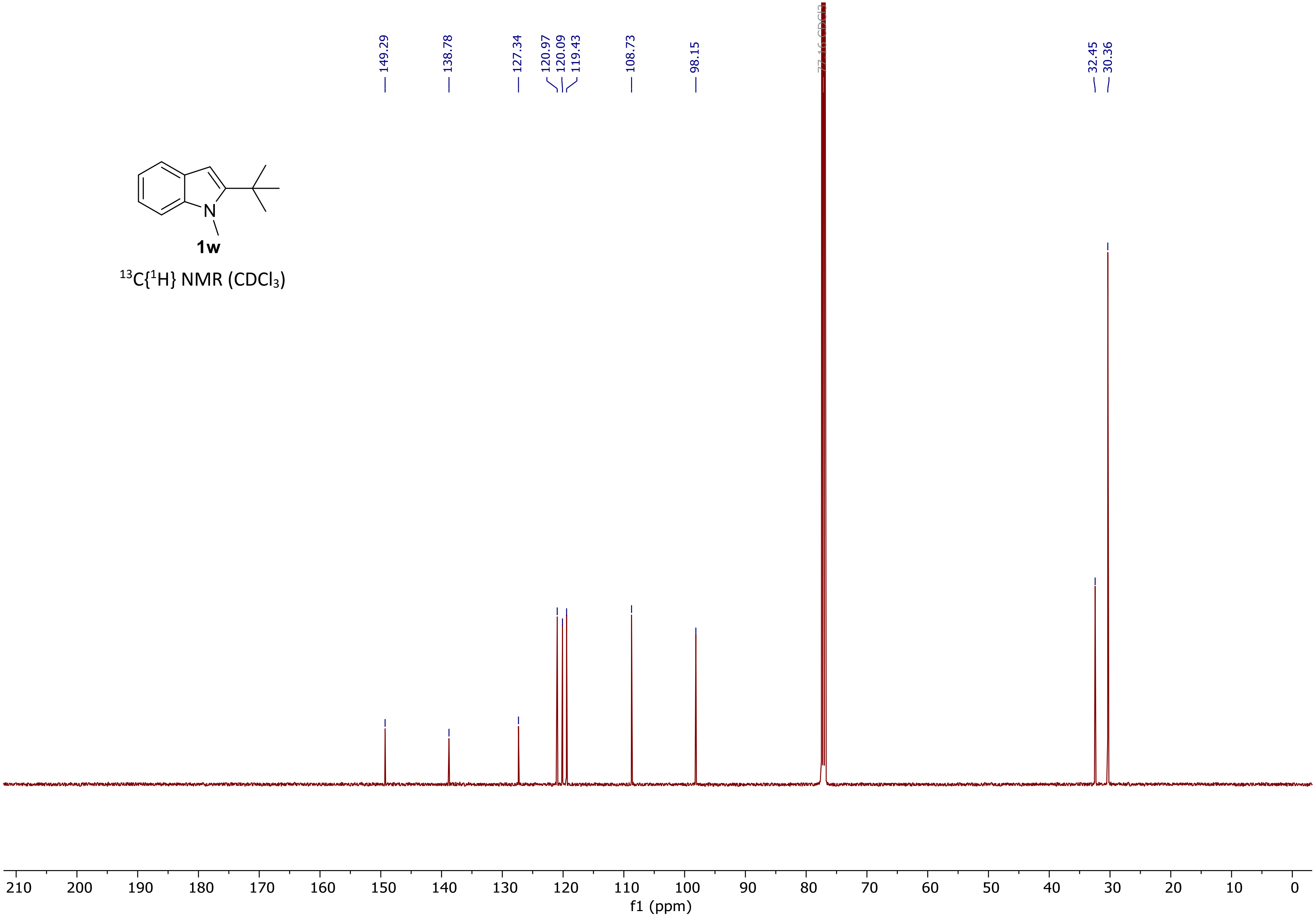
149.29
138.78
127.34
120.97
120.09
119.43
108.73
98.15
77.16 CDCl3
32.45
30.36
1w
$^{13}C\{^{1}H\}$ NMR ($CDCl_3$)
210 200 190 180 170 160 150 140 130 120 110 100 90 80 70 60 50 40 30 20 10 0
f1 (ppm)

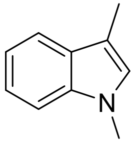

1y

^{1}H NMR ($CDCl_3$)

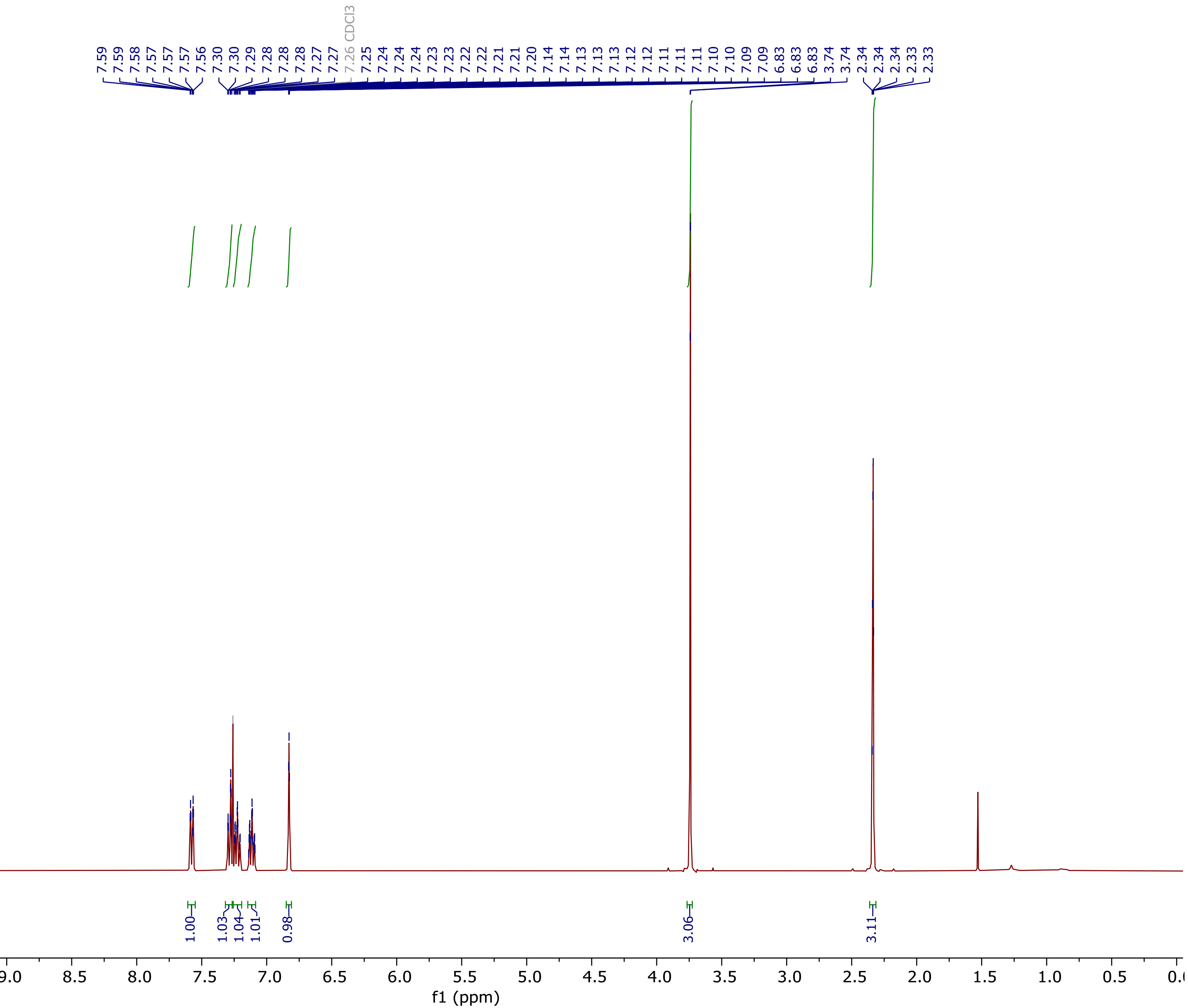

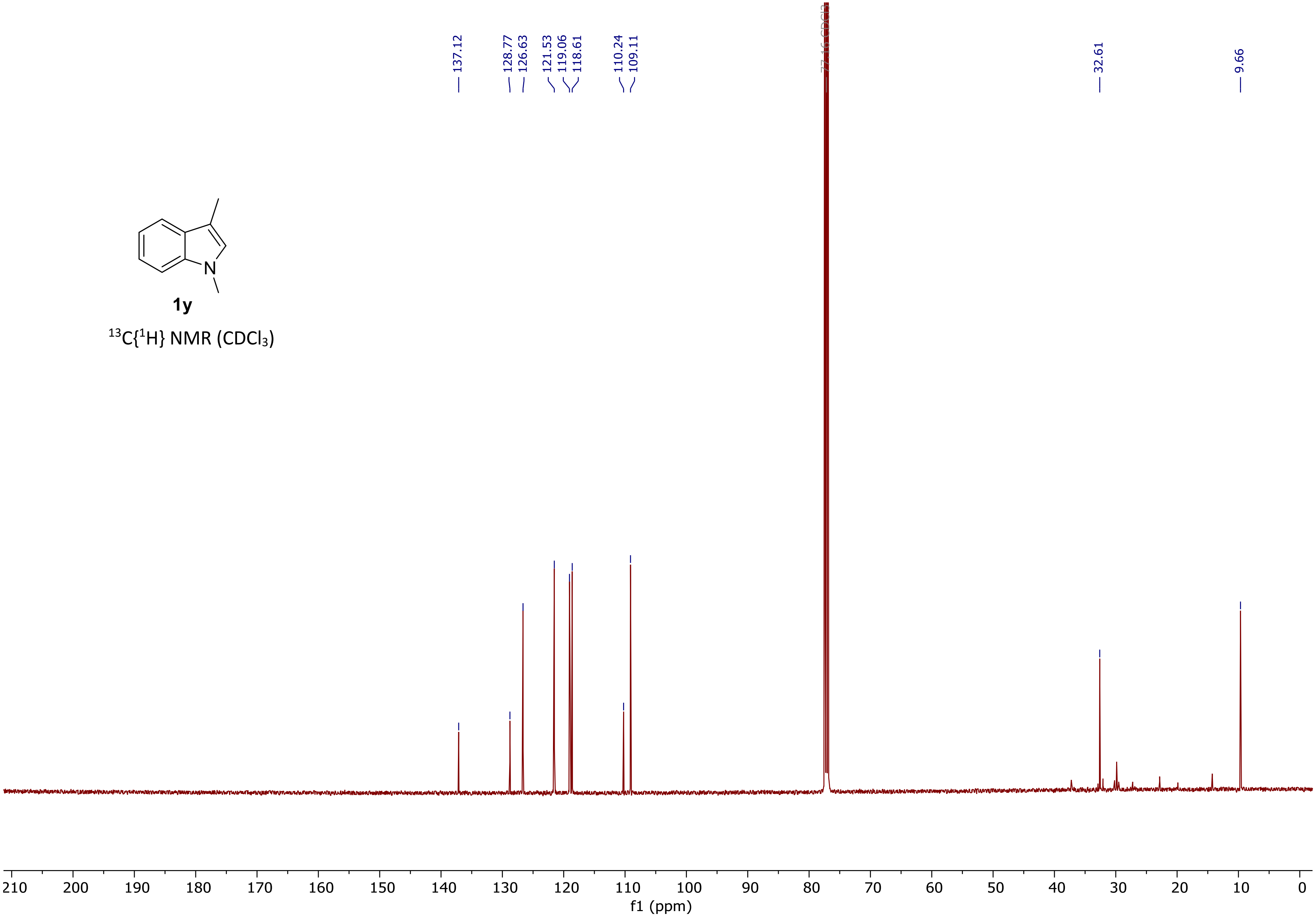

1y

$^{13}C\{^{1}H\}$ NMR ($CDCl_3$)

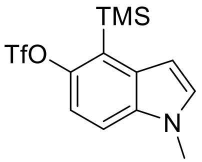

1z

^{1}H NMR ($CDCl_3$)

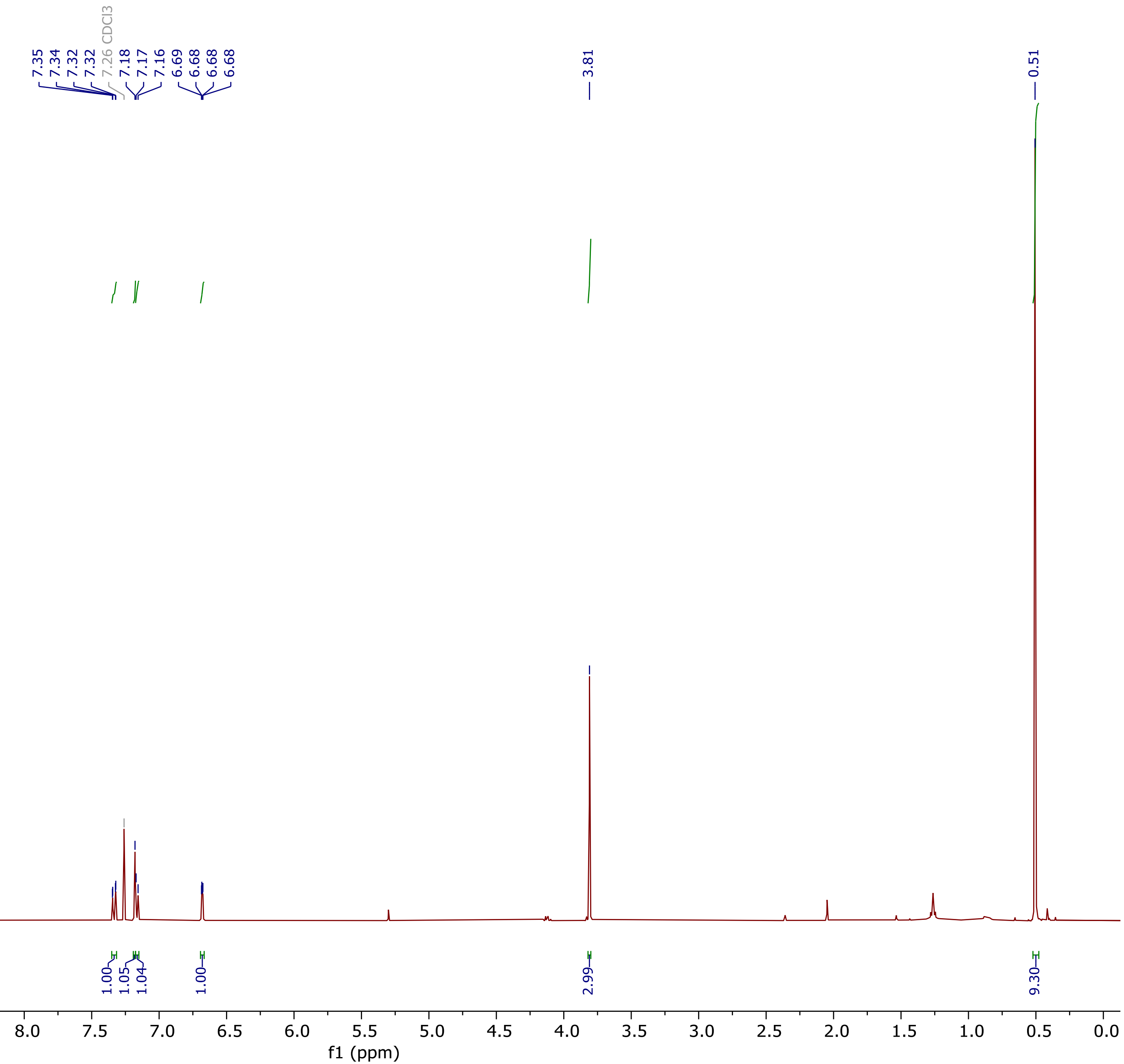

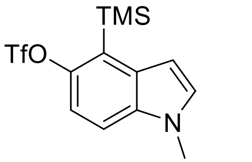

1z

$^{13}C\{^1H\}$ NMR ($CDCl_3$)

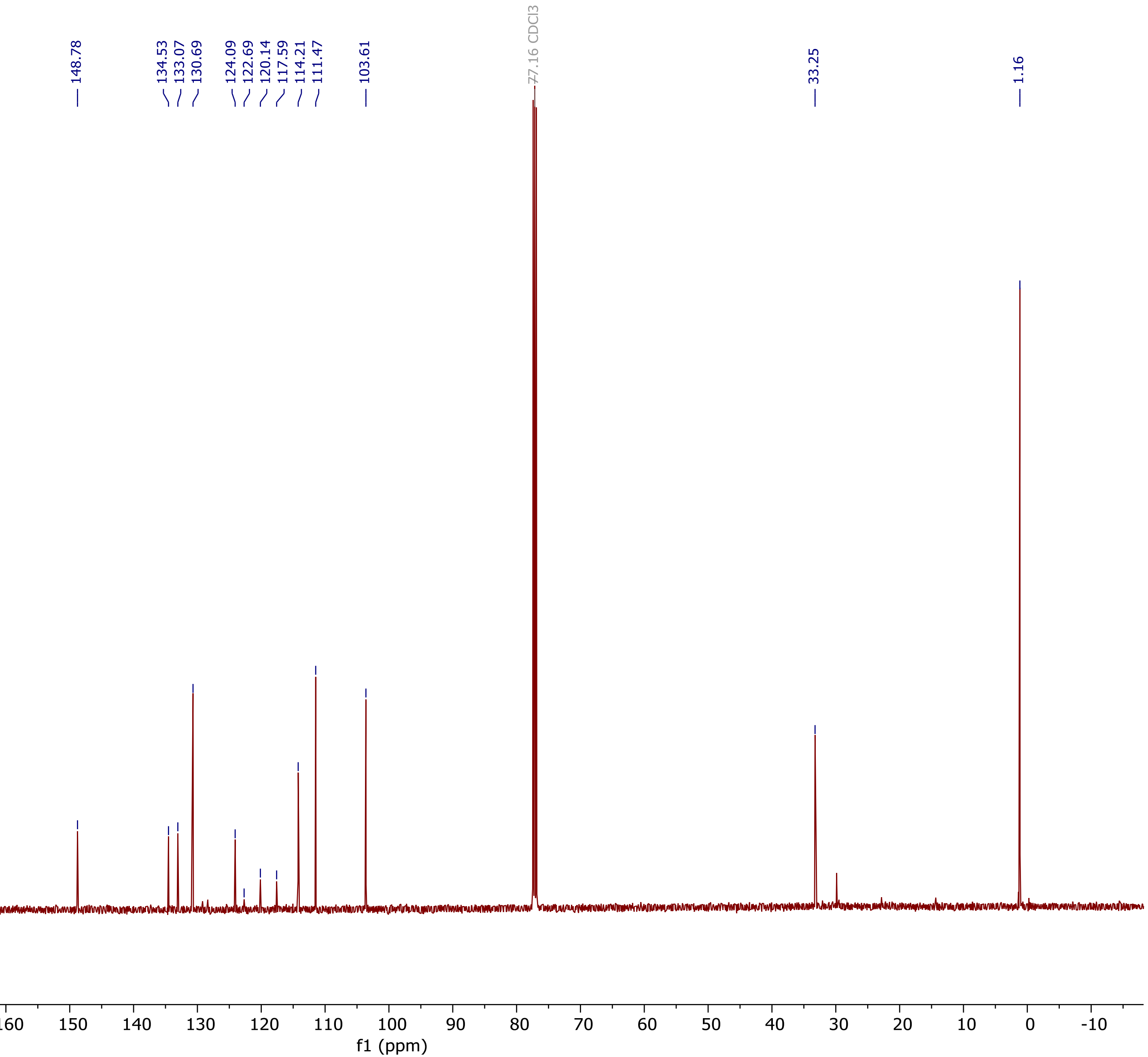

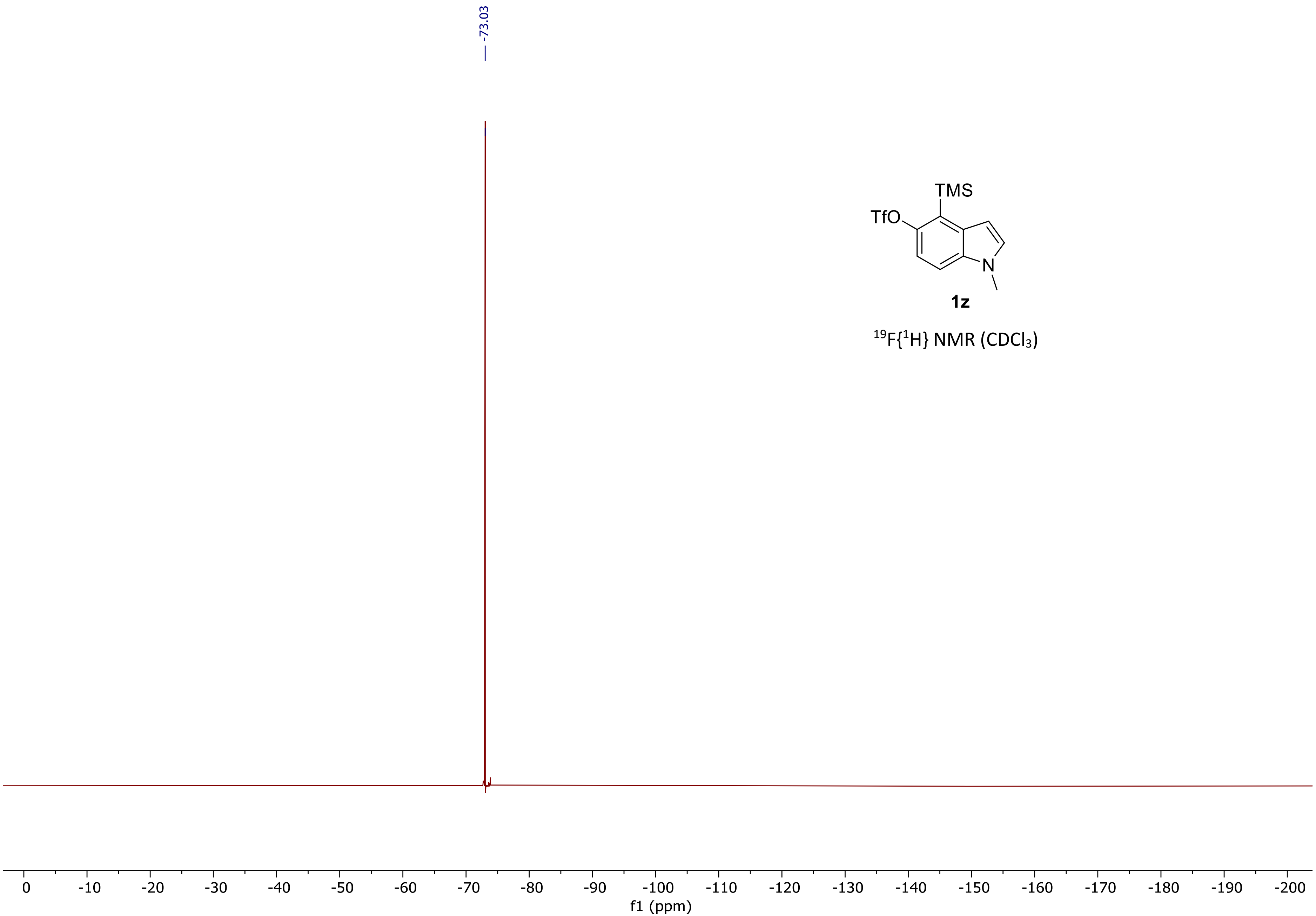

1z

$^{19}F\{^{1}H\}$ NMR ($CDCl_3$)

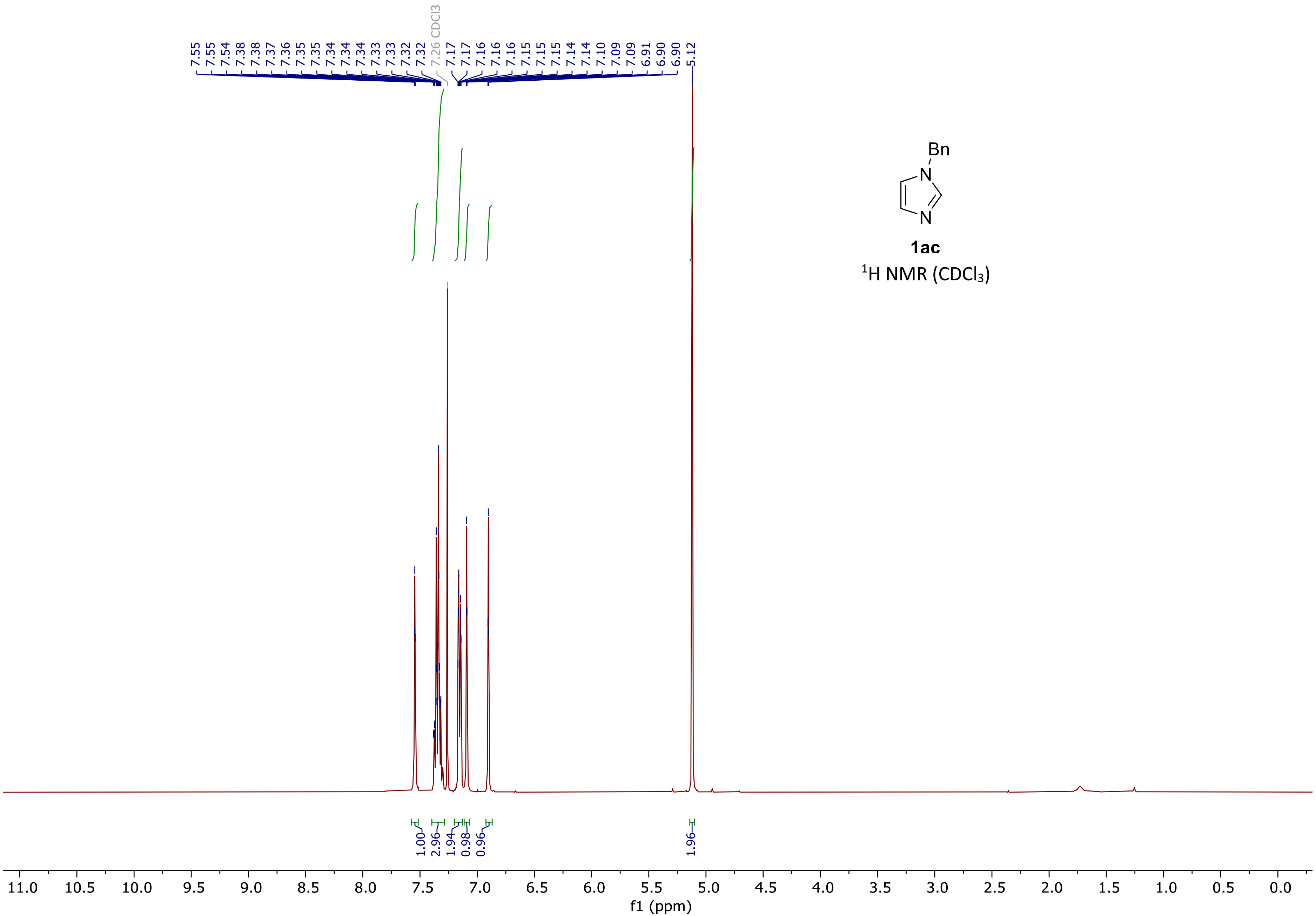
7.55
7.55
7.54
7.38
7.38
7.37
7.36
7.35
7.35
7.34
7.34
7.34
7.33
7.33
7.32
7.32
7.26 CDCl3
7.17
7.17
7.16
7.16
7.16
7.15
7.15
7.15
7.14
7.14
7.10
7.09
7.09
6.91
6.90
6.90
5.12
Bn
N
N
1ac
1H NMR (CDCl3)
1.00
2.96
1.94
0.98
0.96
1.96
11.0
10.5
10.0
9.5
9.0
8.5
8.0
7.5
7.0
6.5
6.0
5.5
5.0
4.5
4.0
3.5
3.0
2.5
2.0
1.5
1.0
0.5
0.0
f1 (ppm)

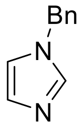

1ac

$^{13}C\{^{1}H\}$ NMR ($CDCl_3$)

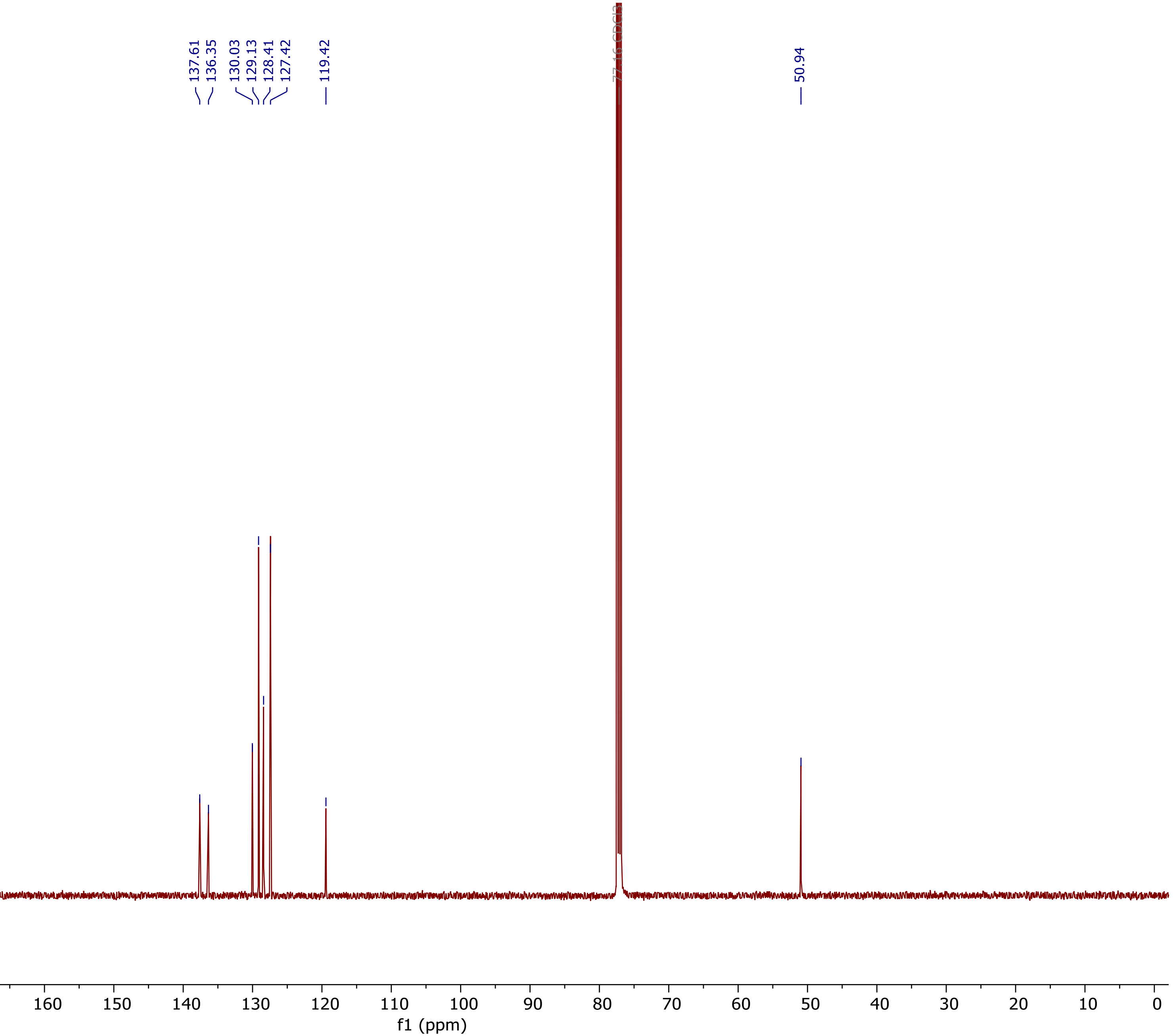

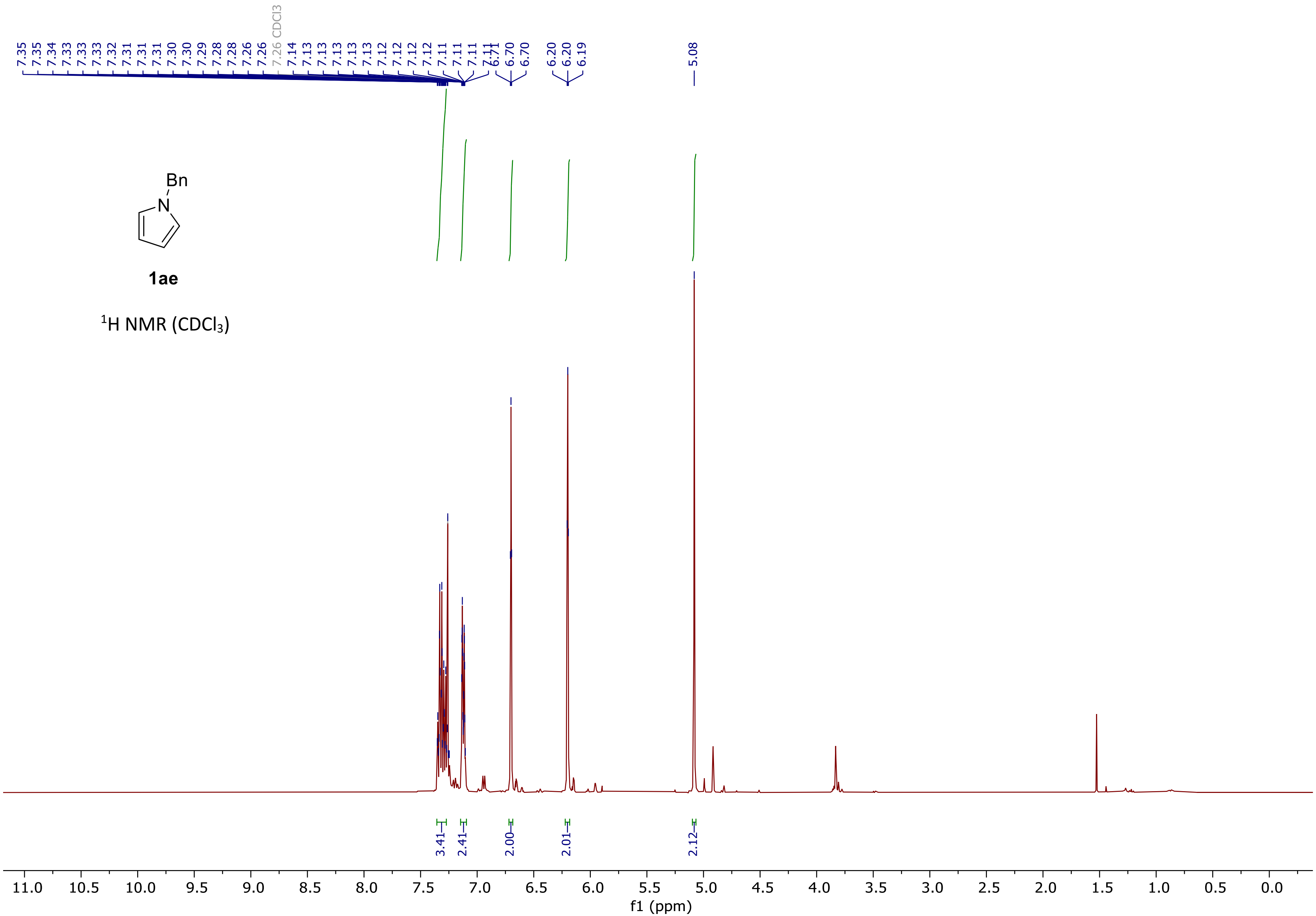
Bn
N
1ae
^{1}H NMR ($CDCl_3$)
7.35 7.35 7.34 7.33 7.33 7.33 7.32 7.31 7.31 7.31 7.30 7.30 7.29 7.28 7.28 7.26 7.26 7.26 CDCl3 7.14 7.13 7.13 7.13 7.13 7.13 7.12 7.12 7.12 7.12 7.11 7.11 7.11 7.11
6.71 6.70 6.70
6.20 6.20 6.19
5.08
3.41 2.41 2.00 2.01 2.12
11.0 10.5 10.0 9.5 9.0 8.5 8.0 7.5 7.0 6.5 6.0 5.5 5.0 4.5 4.0 3.5 3.0 2.5 2.0 1.5 1.0 0.5 0.0
f1 (ppm)

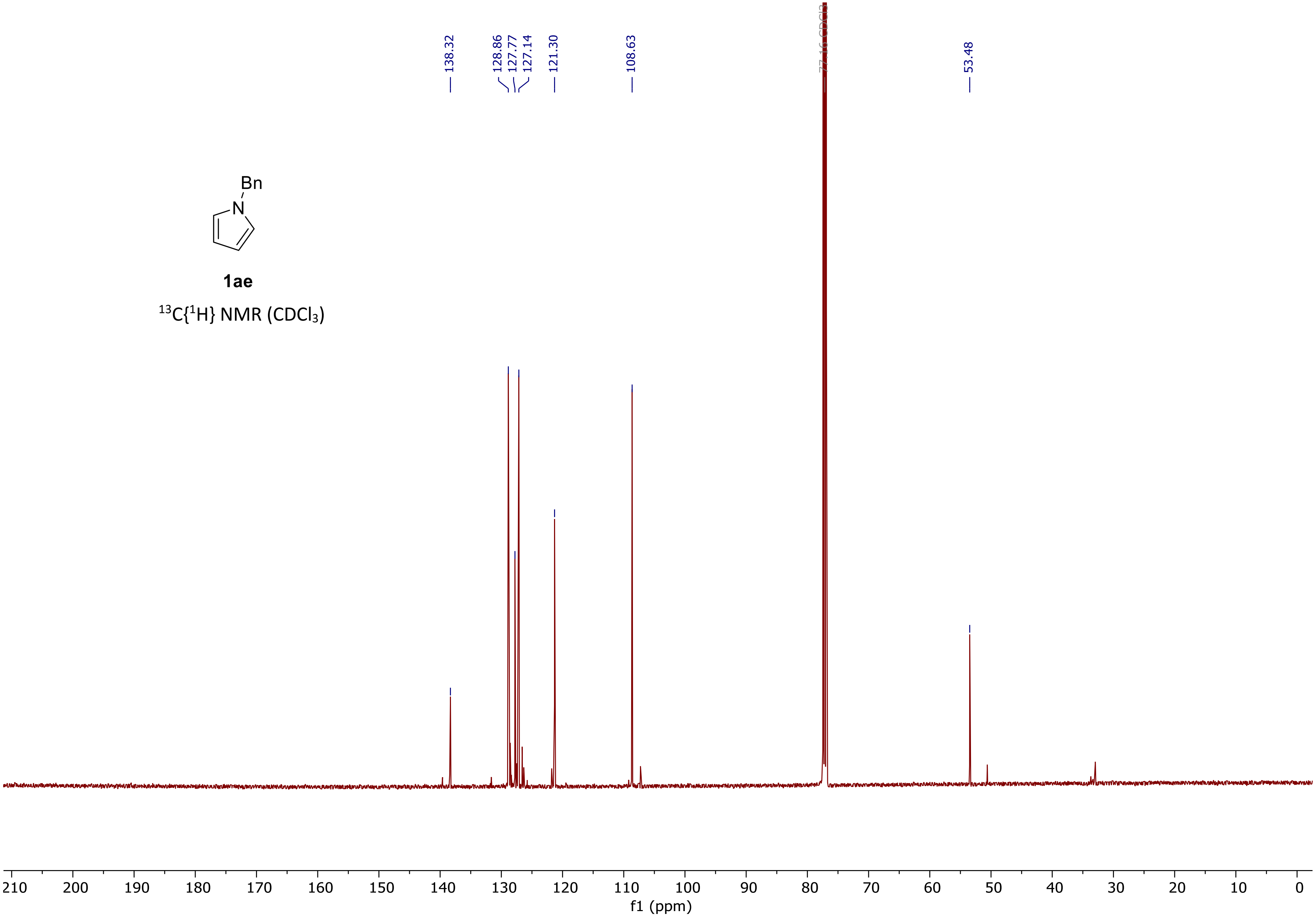

138.32
128.86
127.77
127.14
121.30
108.63
77.16 CDCl3
53.48
Bn
N
1ae
$^{13}C\{^{1}H\}$ NMR ($CDCl_3$)
210 200 190 180 170 160 150 140 130 120 110 100 90 80 70 60 50 40 30 20 10 0
f1 (ppm)

2a

^{1}H NMR ($CDCl_3$)

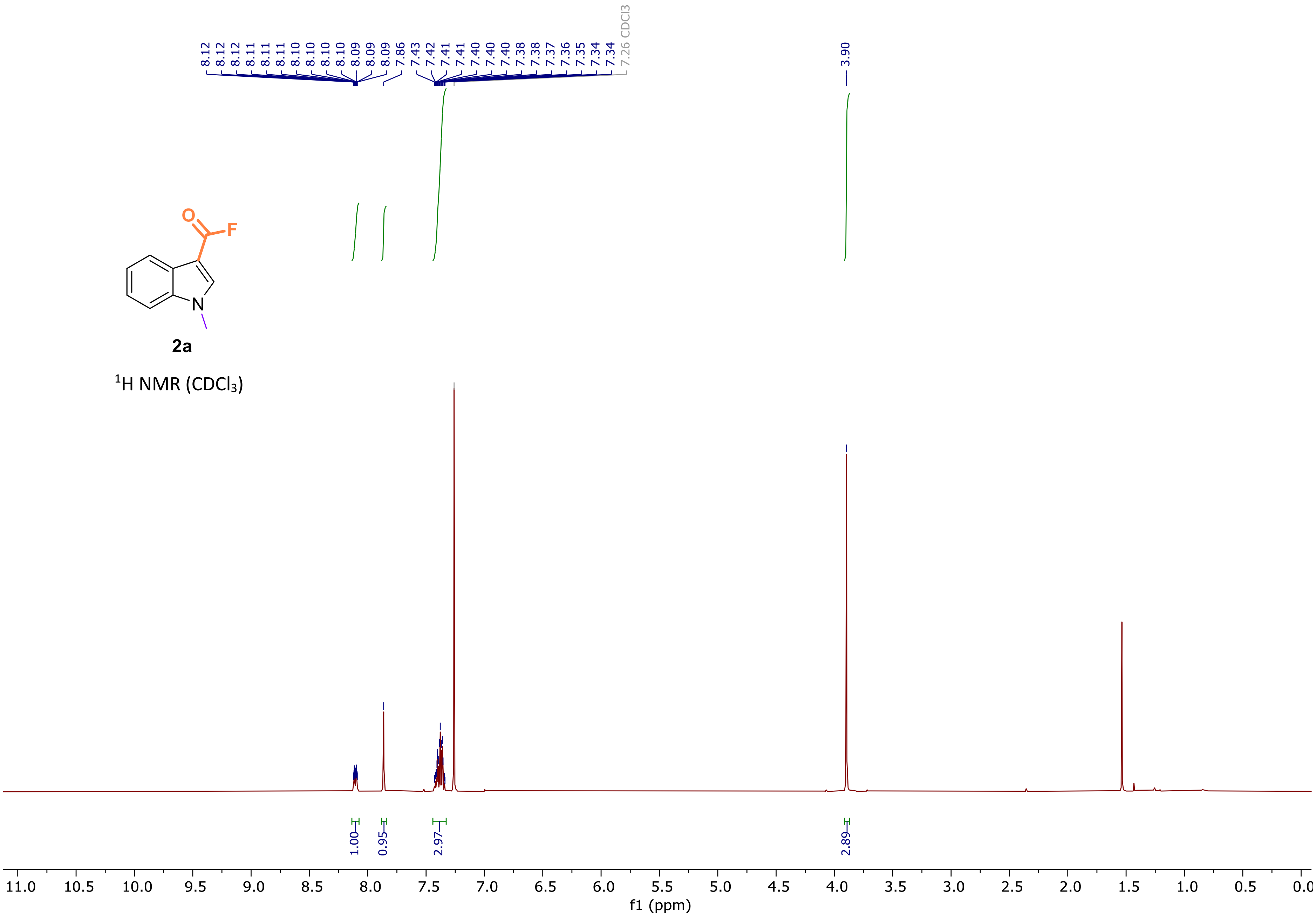

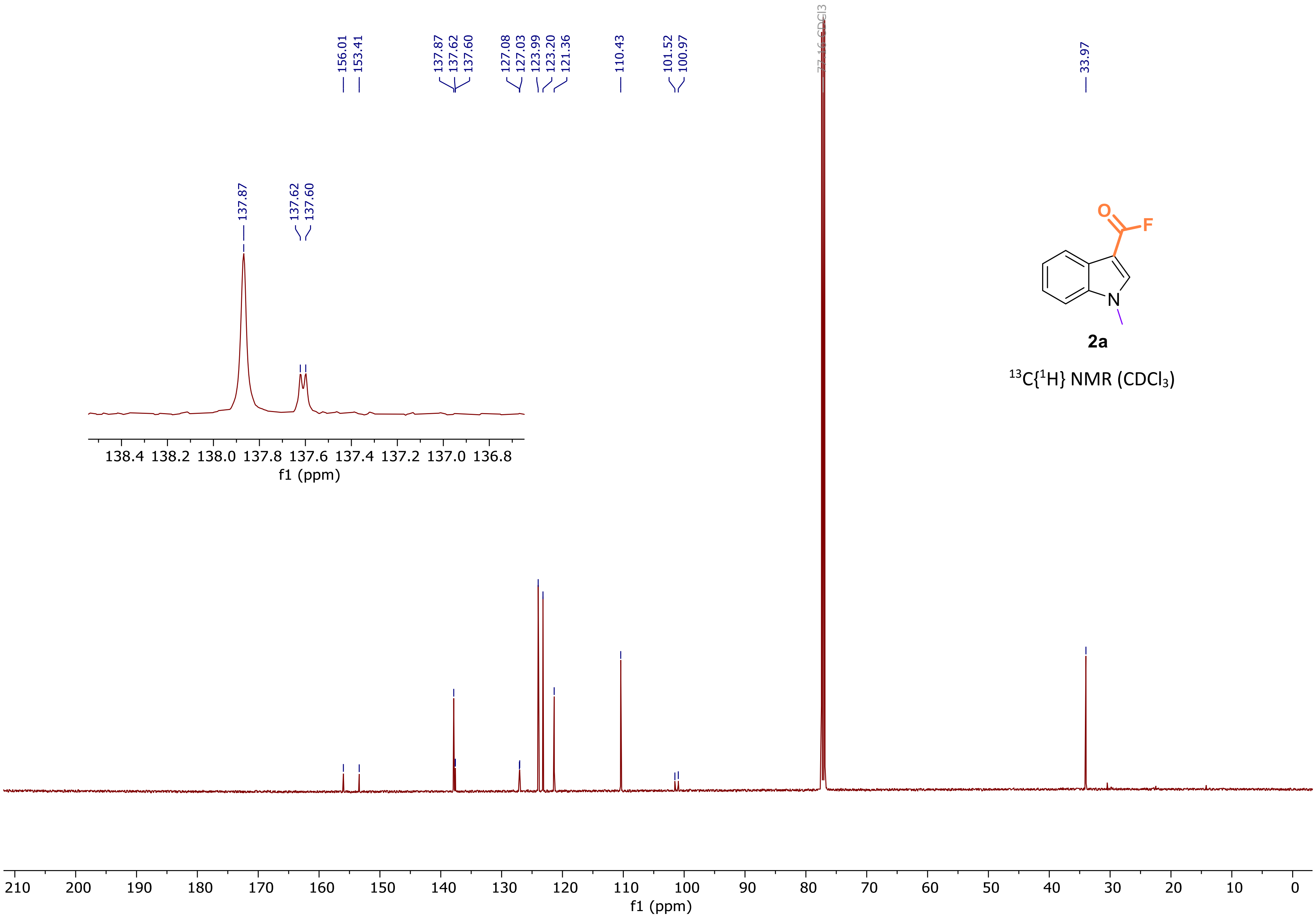
156.01
153.41
137.87
137.62
137.60
127.08
127.03
123.99
123.20
121.36
110.43
101.52
100.97
77.16 CDCl3
33.97
138.4 138.2 138.0 137.8 137.6 137.4 137.2 137.0 136.8
f1 (ppm)
O
F
N
2a
$^{13}C\{^{1}H\}$ NMR ($CDCl_3$)
210 200 190 180 170 160 150 140 130 120 110 100 90 80 70 60 50 40 30 20 10 0
f1 (ppm)

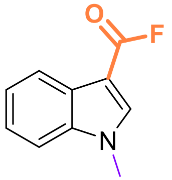

2a

$^{19}F\{^1H\}$ NMR ($CDCl_3$)

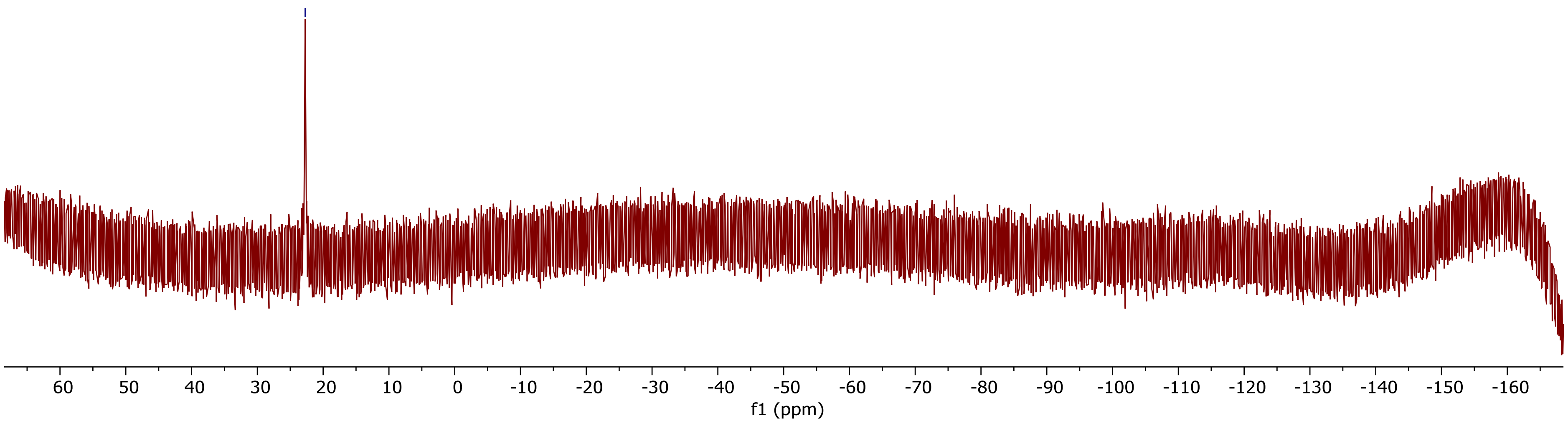

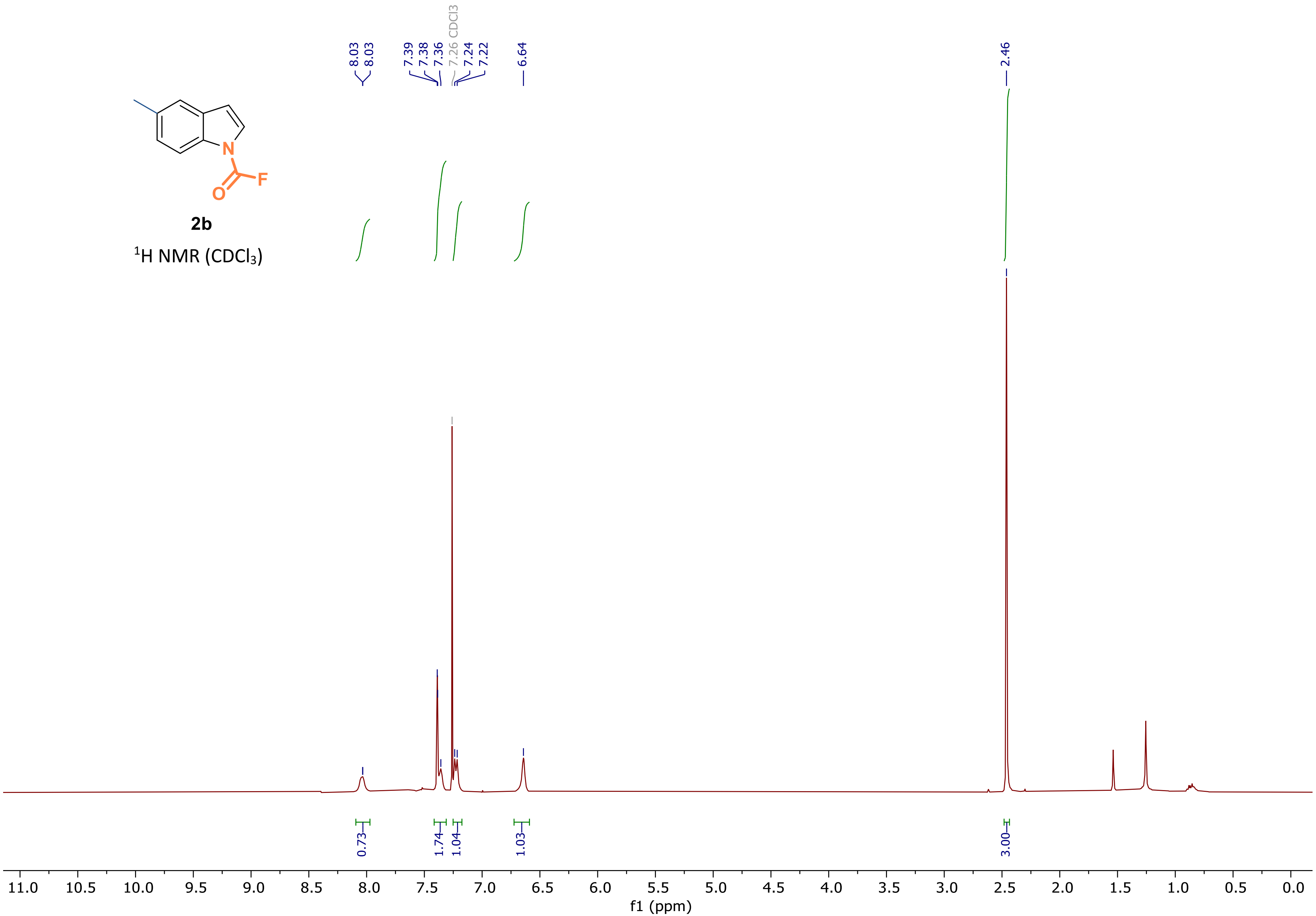

N
F
O
2b
^{1}H NMR (CDCl$_3$)
8.03
8.03
7.39
7.38
7.36
7.26 CDCl3
7.24
7.22
6.64
2.46
0.73
1.74
1.04
1.03
3.00
11.0 10.5 10.0 9.5 9.0 8.5 8.0 7.5 7.0 6.5 6.0 5.5 5.0 4.5 4.0 3.5 3.0 2.5 2.0 1.5 1.0 0.5 0.0
f1 (ppm)

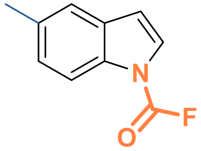

2b

$^{13}C\{^{1}H\}$ NMR ($CDCl_3$)

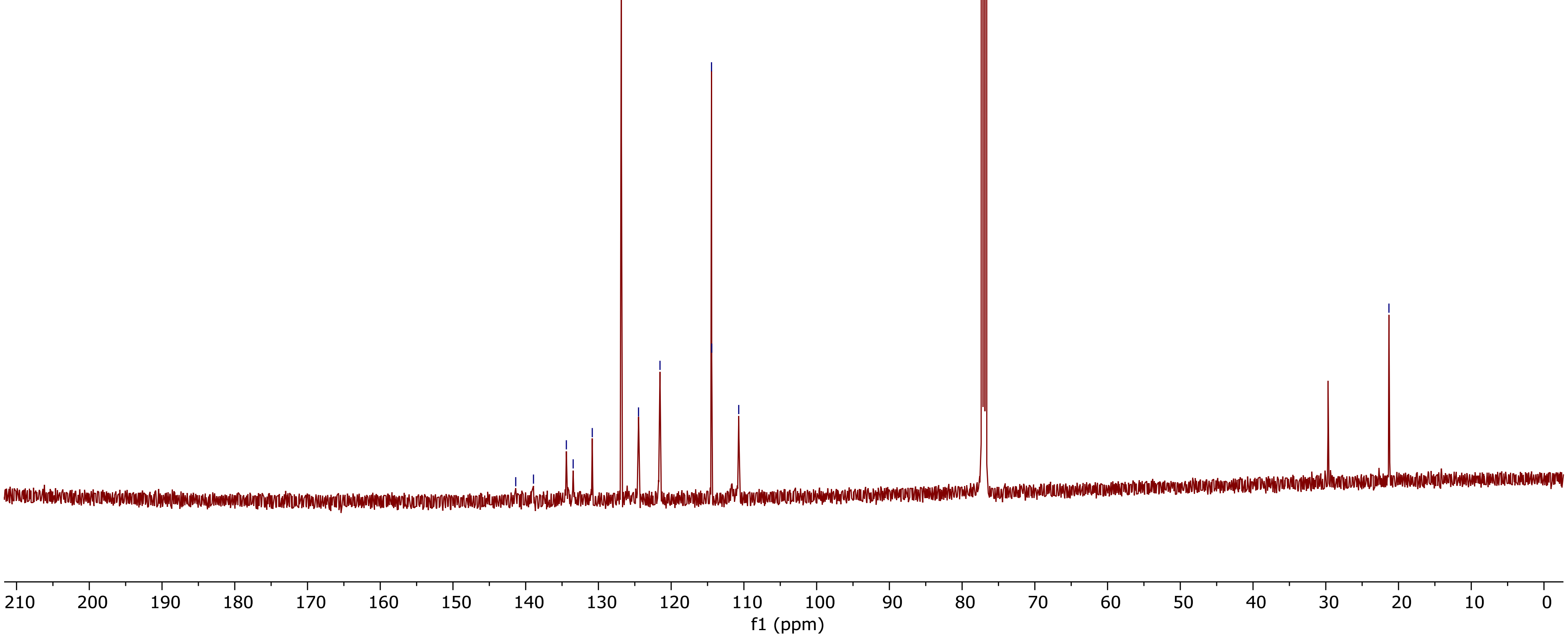

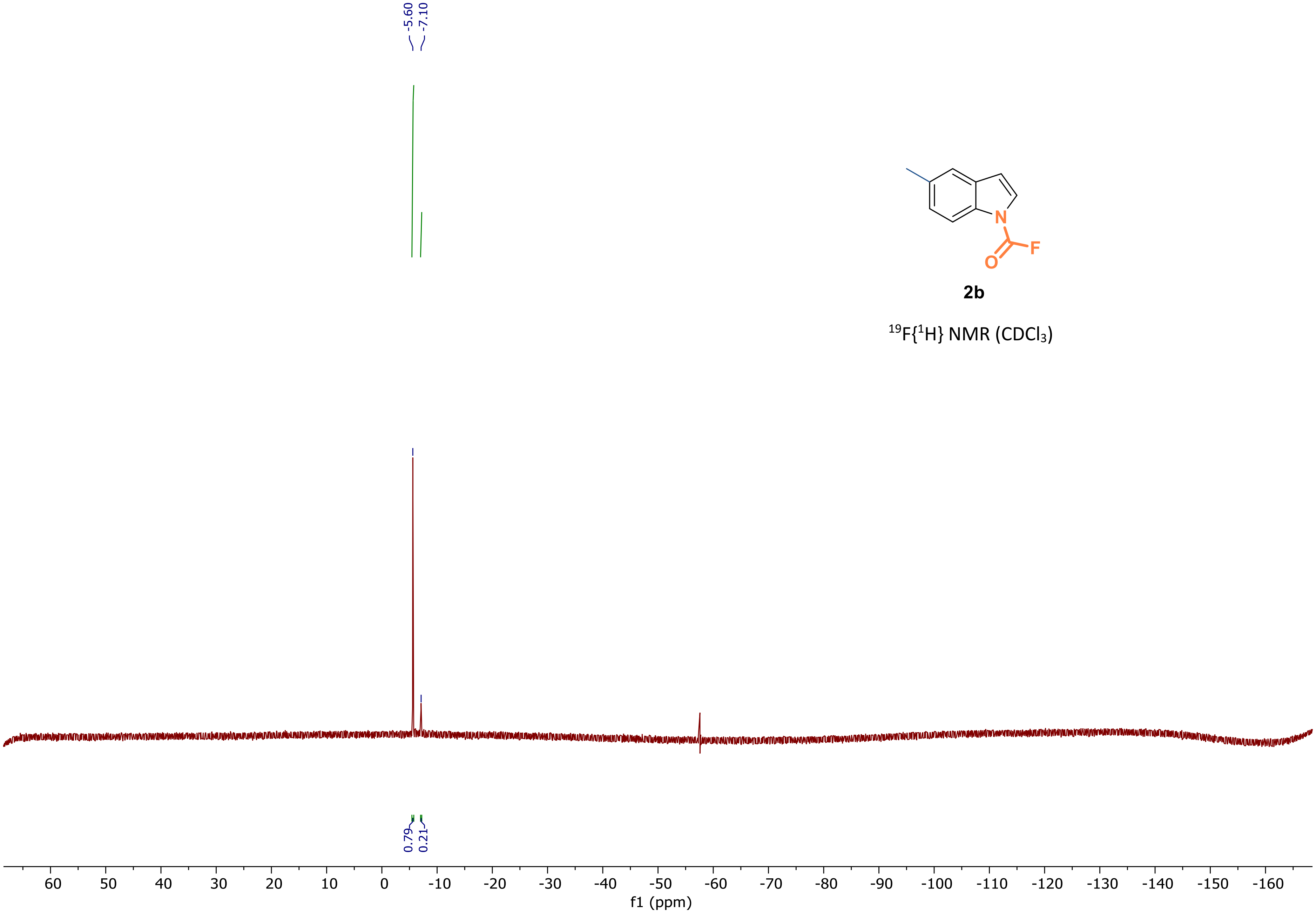

2b

$^{19}F\{^{1}H\}$ NMR ($CDCl_3$)

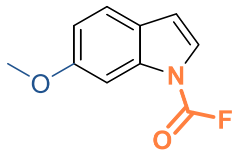

2c

^{1}H NMR ($CDCl_3$)

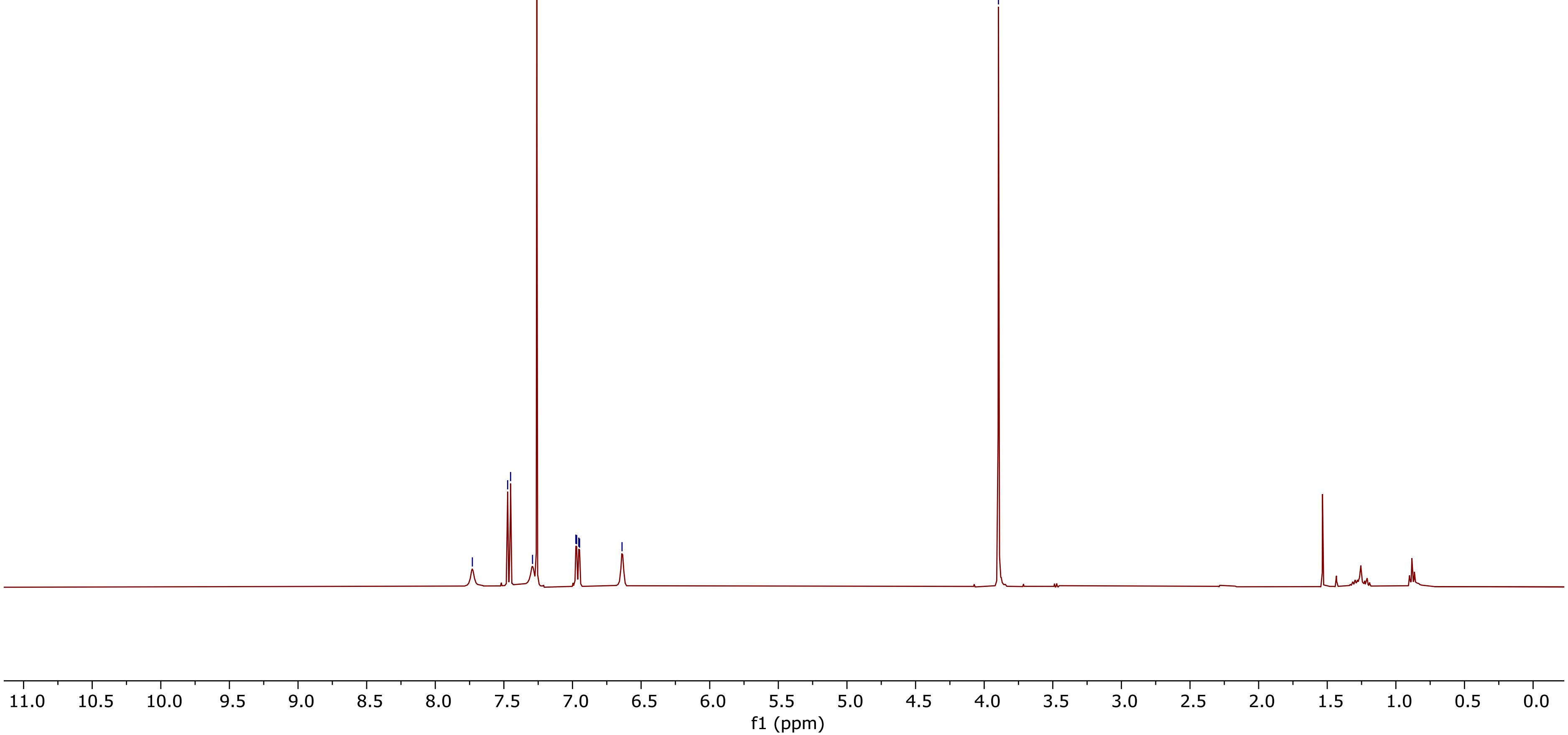

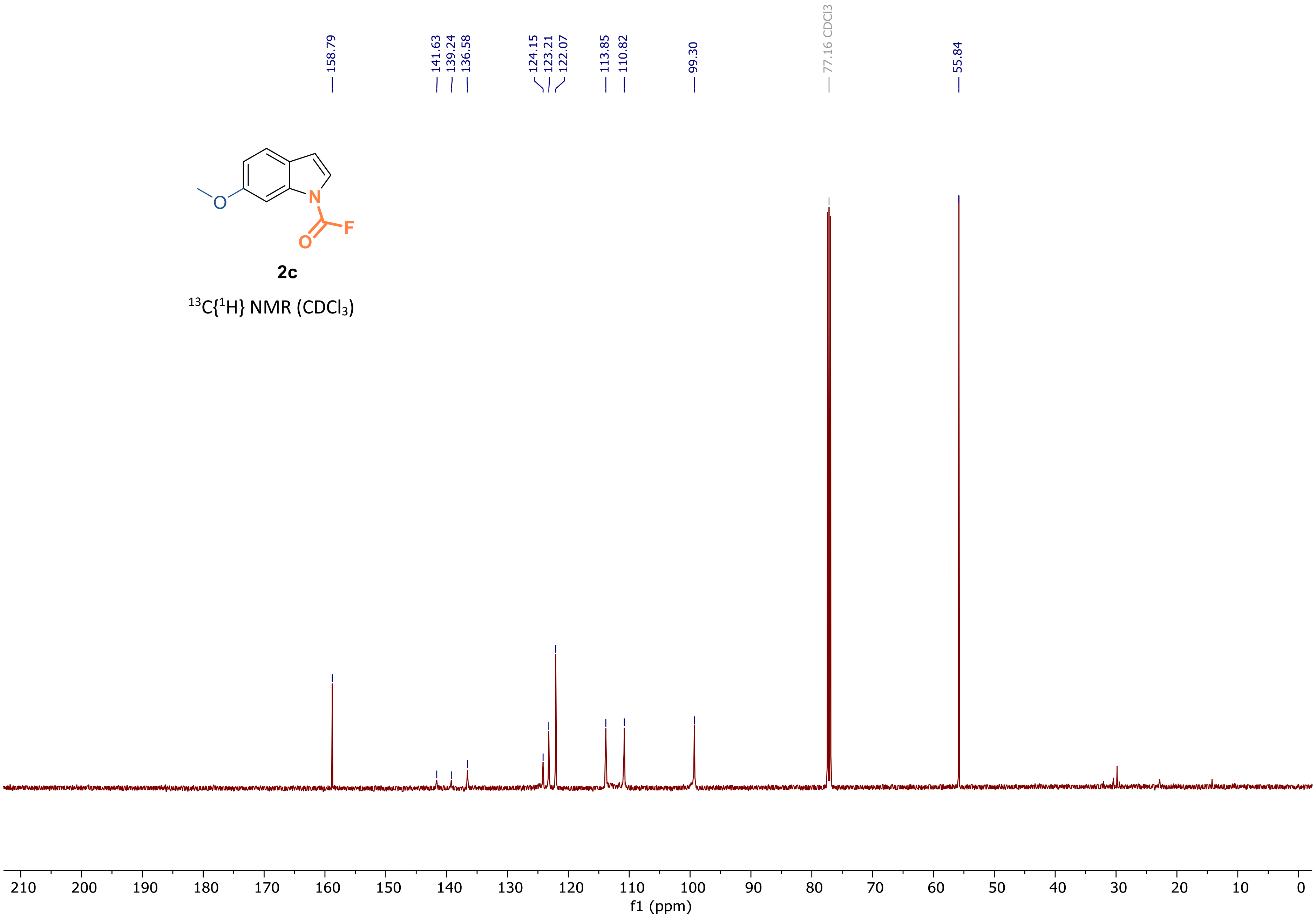
158.79
141.63
139.24
136.58
124.15
123.21
122.07
113.85
110.82
99.30
77.16 CDCl3
55.84
O
N
F
O
2c
$^{13}C\{^{1}H\}$ NMR ($CDCl_3$)
210 200 190 180 170 160 150 140 130 120 110 100 90 80 70 60 50 40 30 20 10 0
f1 (ppm)

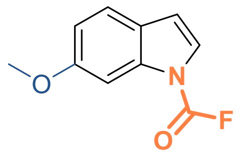

2c

$^{19}F\{^{1}H\}$ NMR ($CDCl_3$)

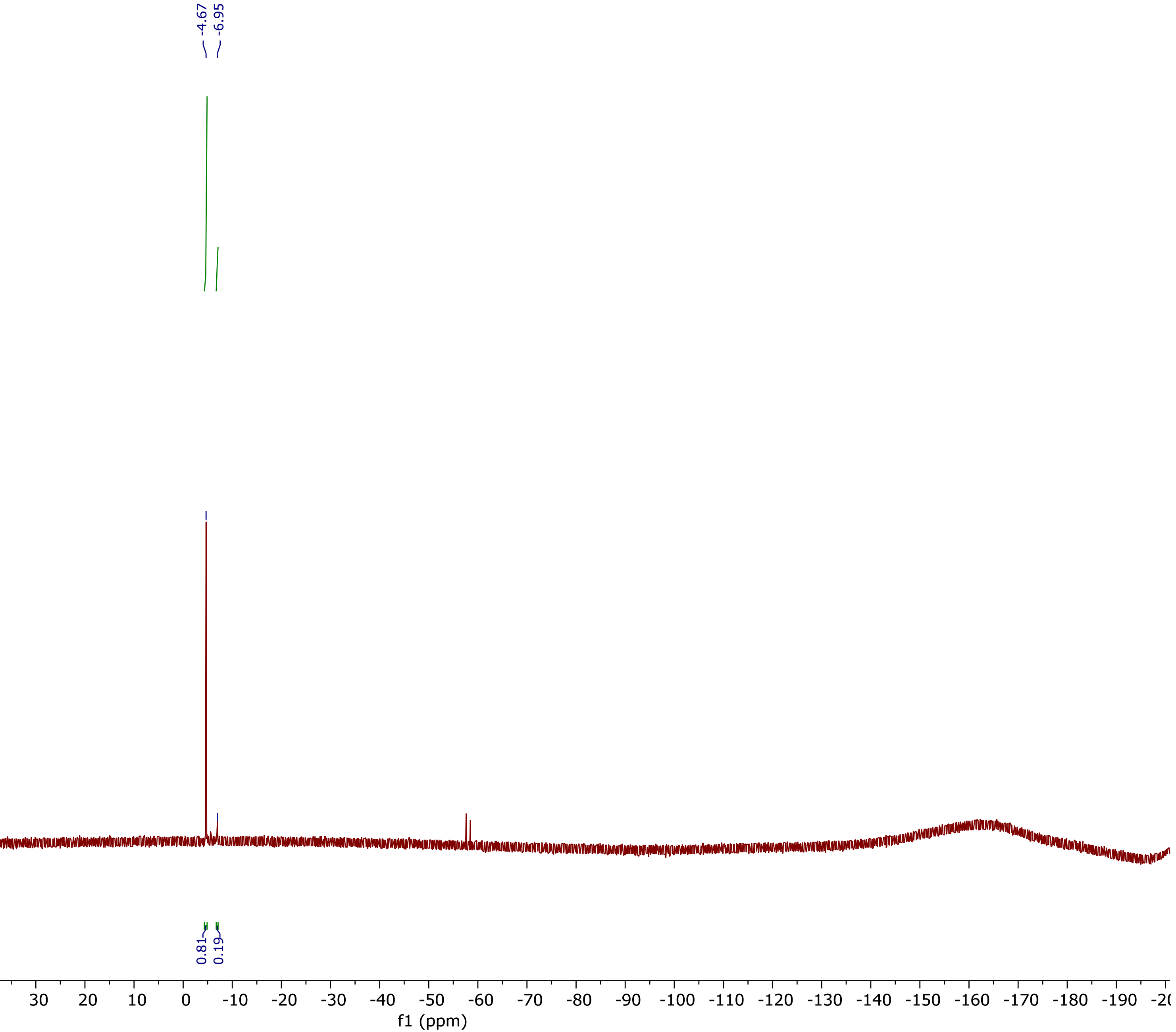

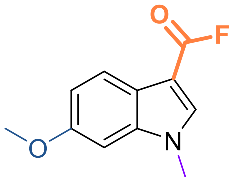

2d

^{1}H NMR ($CDCl_3$)

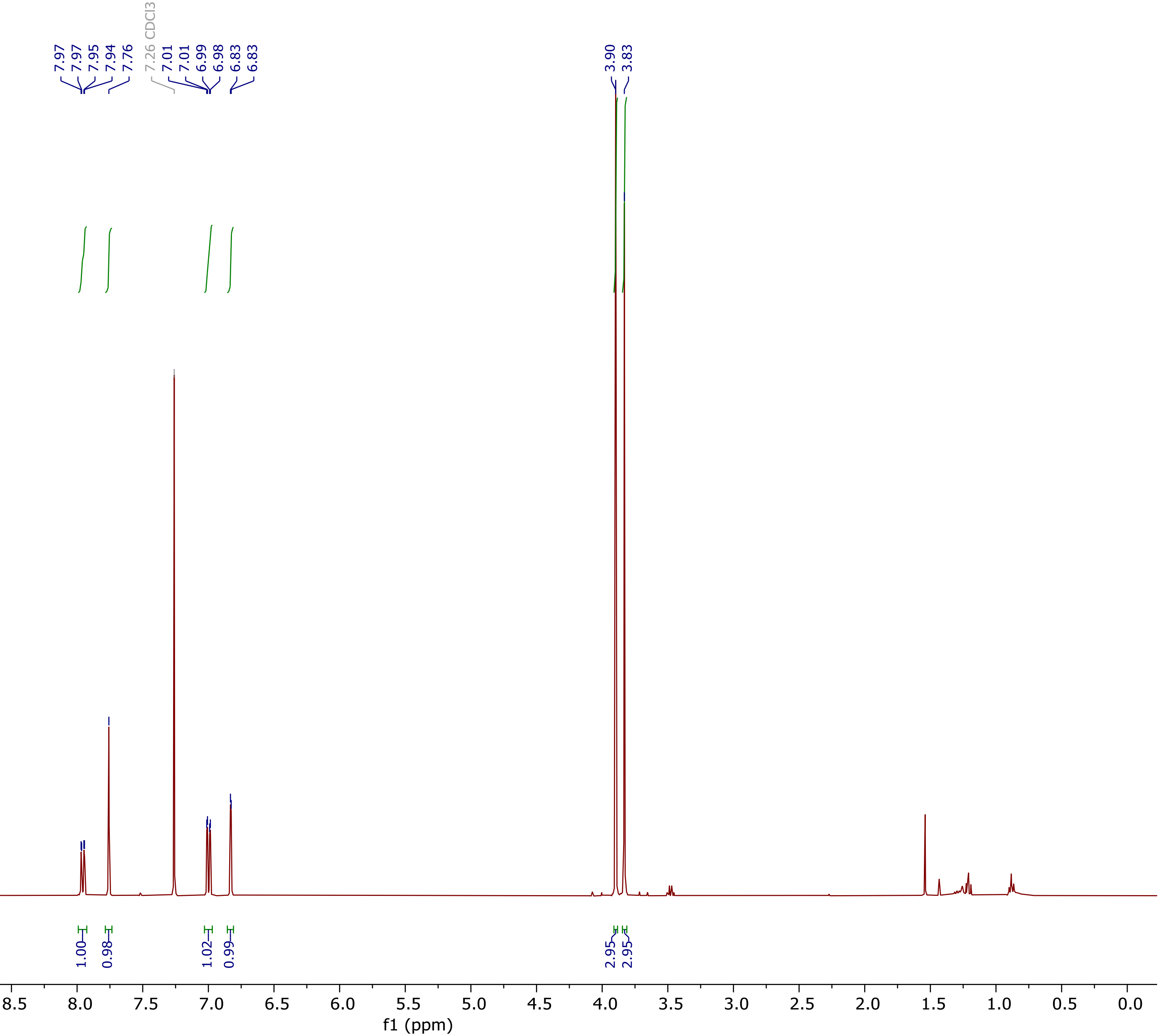

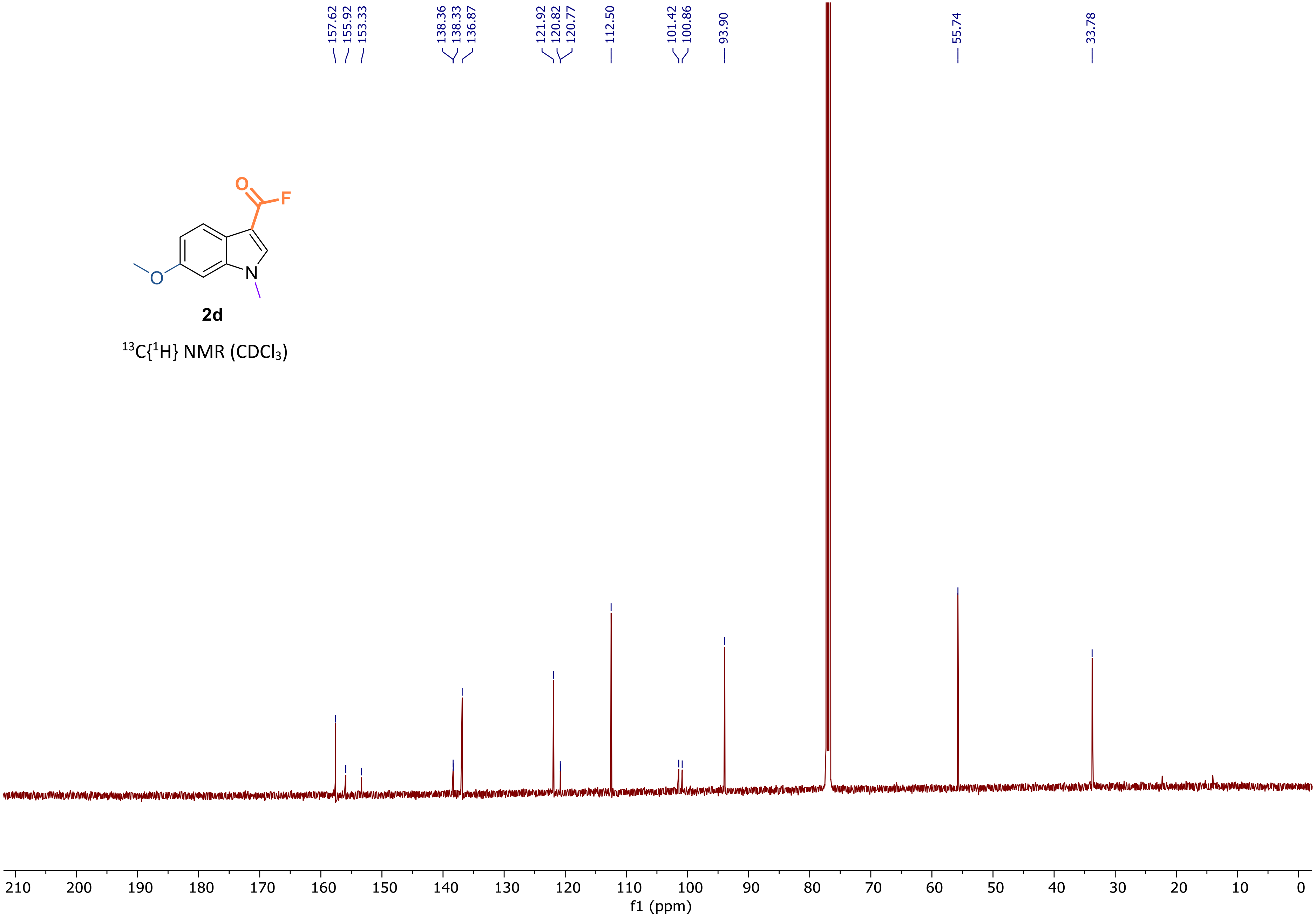
157.62
155.92
153.33
138.36
138.33
136.87
121.92
120.82
120.77
112.50
101.42
100.86
93.90
55.74
33.78
O
F
O
N
2d
$^{13}C\{^1H\}$ NMR ($CDCl_3$)
210 200 190 180 170 160 150 140 130 120 110 100 90 80 70 60 50 40 30 20 10 0
f1 (ppm)

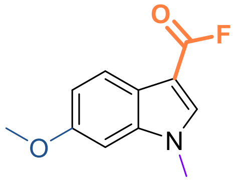

2d

$^{19}F\{^{1}H\}$ NMR ($CDCl_3$)

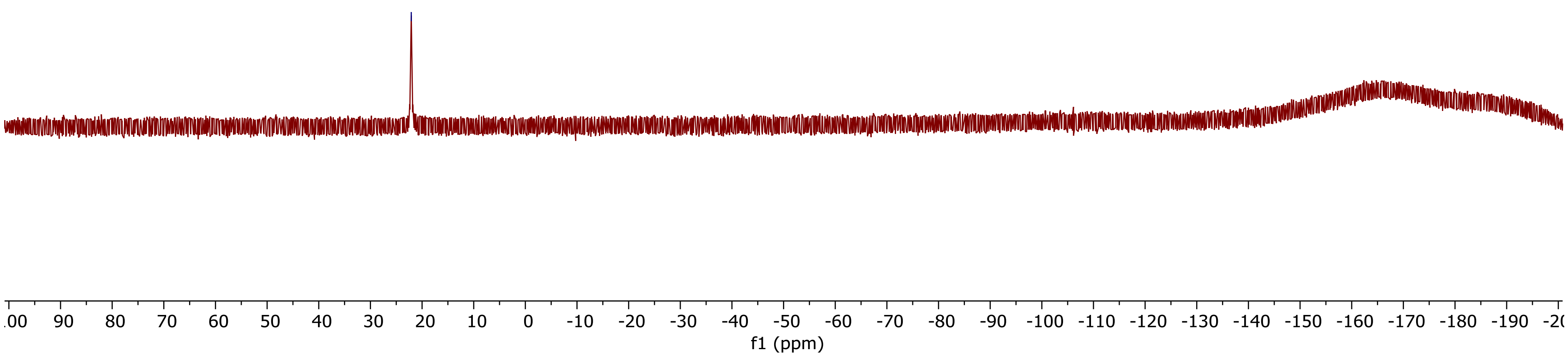

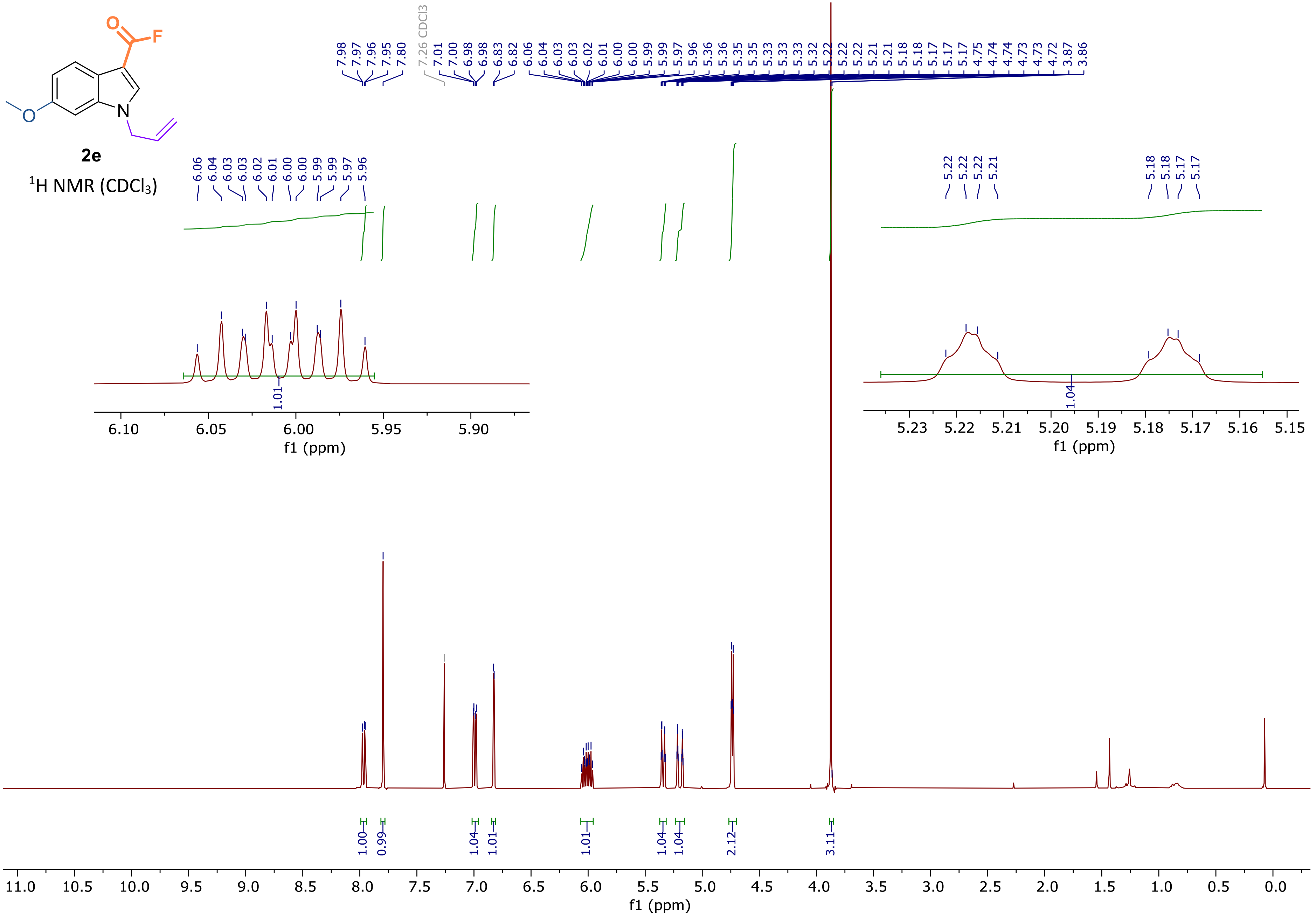

2e
¹H NMR (CDCl₃)
O
F
O
N
7.98 7.97 7.96 7.95 7.80 7.26 CDCl3 7.01 7.00 6.98 6.98 6.83 6.82 6.06 6.04 6.03 6.03 6.02 6.01 6.00 6.00 5.99 5.99 5.97 5.96 5.36 5.36 5.35 5.35 5.33 5.33 5.33 5.32 5.22 5.22 5.22 5.21 5.21 5.18 5.18 5.17 5.17 5.17 4.75 4.74 4.74 4.73 4.73 4.72 3.87 3.86
1.00 0.99 1.04 1.01 1.01 1.04 1.04 2.12 3.11
f1 (ppm)

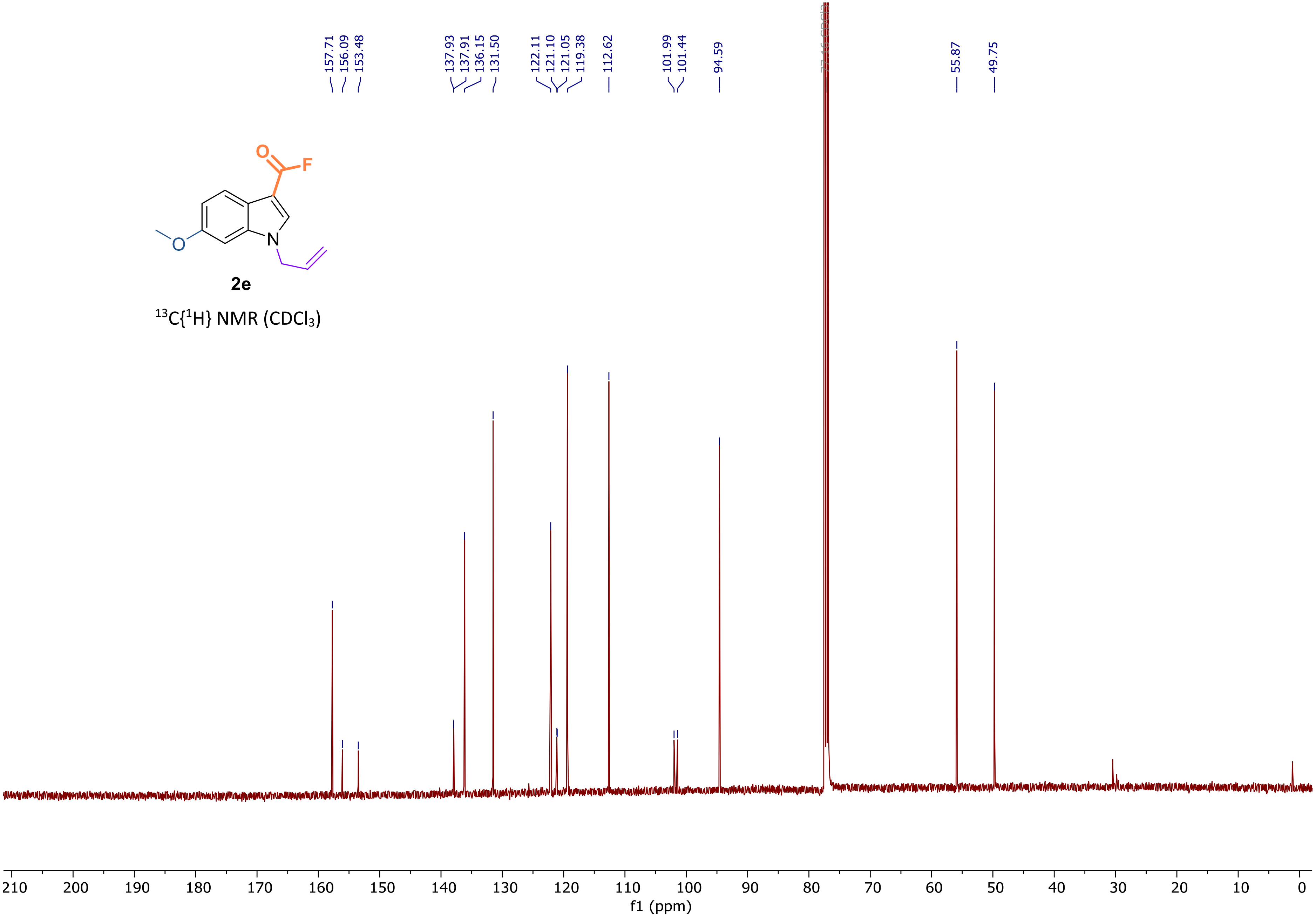
157.71
156.09
153.48
137.93
137.91
136.15
131.50
122.11
121.10
121.05
119.38
112.62
101.99
101.44
94.59
77.16 CDCl3
55.87
49.75
O
F
N
O
2e
$^{13}C\{^{1}H\}$ NMR ($CDCl_3$)
210 200 190 180 170 160 150 140 130 120 110 100 90 80 70 60 50 40 30 20 10 0
f1 (ppm)

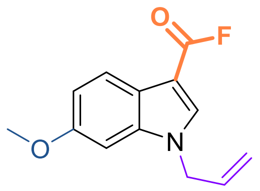

2e

$^{19}F\{^1H\}$ NMR ($CDCl_3$)

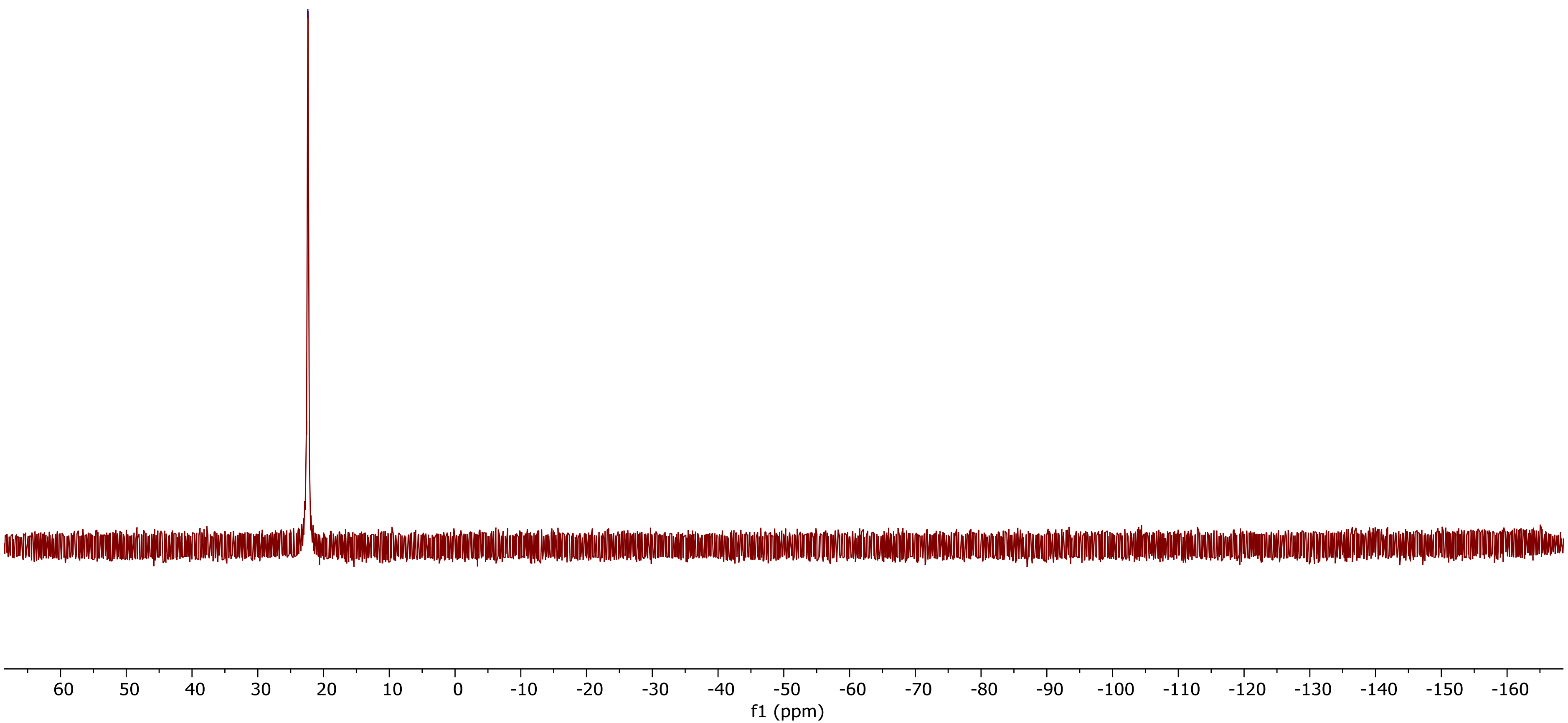

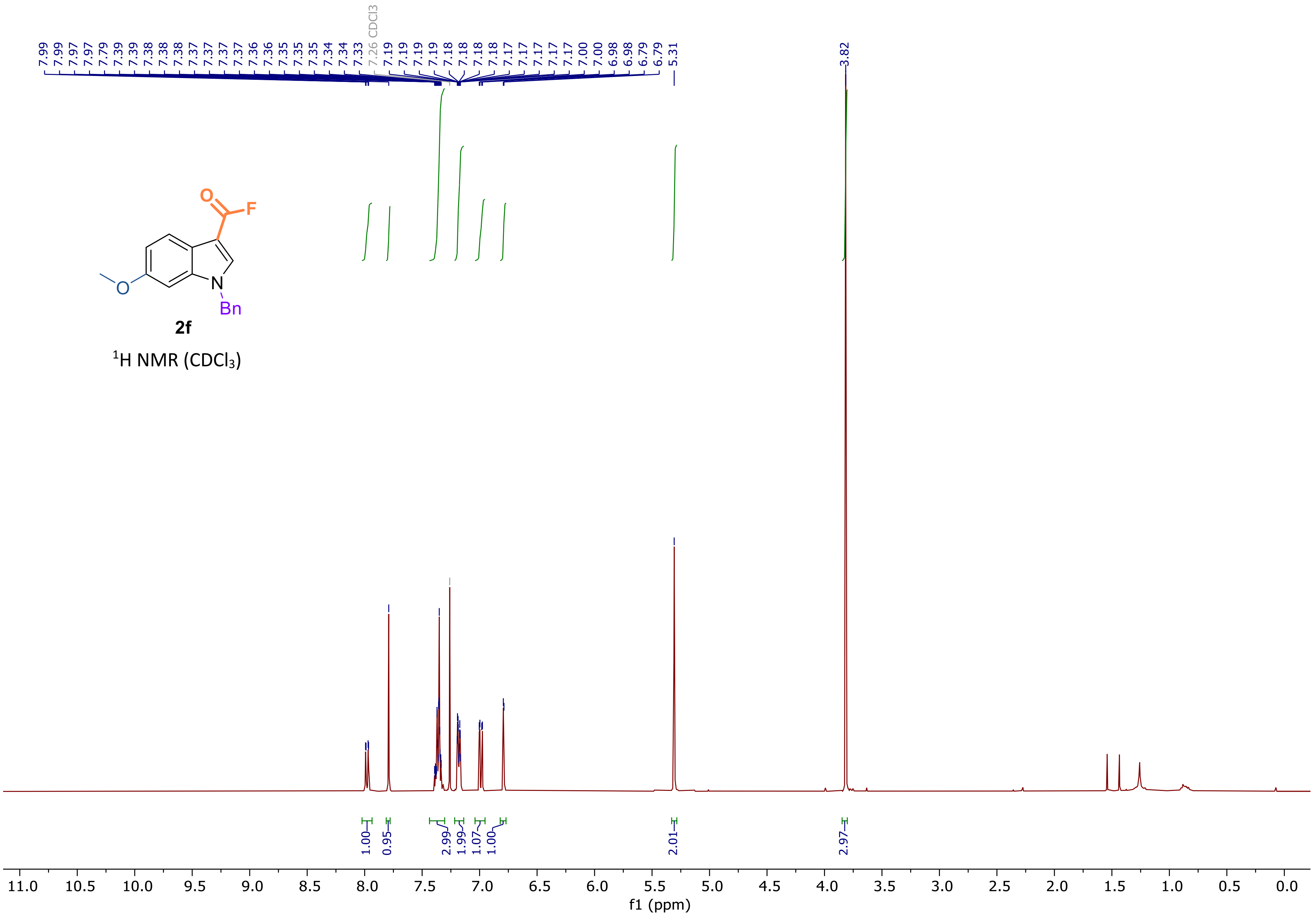
7.99 7.99 7.97 7.97 7.79 7.39 7.39 7.38 7.38 7.38 7.37 7.37 7.37 7.37 7.36 7.36 7.35 7.35 7.35 7.34 7.34 7.33 7.26 CDCl3 7.19 7.19 7.19 7.19 7.18 7.18 7.18 7.18 7.17 7.17 7.17 7.17 7.17 7.00 7.00 6.98 6.98 6.79 6.79 5.31
3.82
O
F
O
N
Bn
2f
1H NMR (CDCl3)
1.00 0.95 2.99 1.99 1.07 1.00 2.01 2.97
11.0 10.5 10.0 9.5 9.0 8.5 8.0 7.5 7.0 6.5 6.0 5.5 5.0 4.5 4.0 3.5 3.0 2.5 2.0 1.5 1.0 0.5 0.0
f1 (ppm)

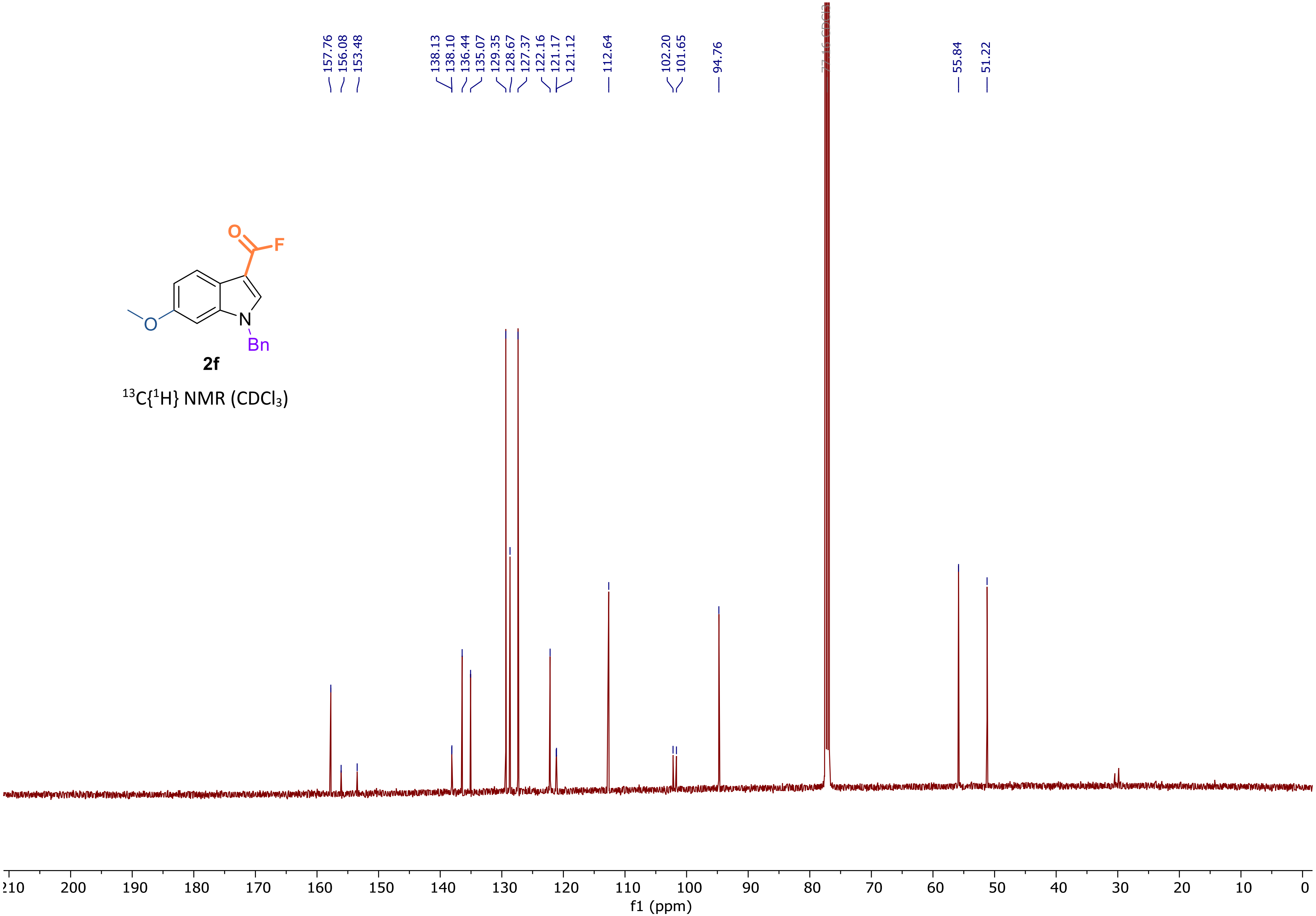

157.76
156.08
153.48
138.13
138.10
136.44
135.07
129.35
128.67
127.37
122.16
121.17
121.12
112.64
102.20
101.65
94.76
55.84
51.22
O
F
N
Bn
2f
$^{13}C\{^1H\}$ NMR ($CDCl_3$)
210 200 190 180 170 160 150 140 130 120 110 100 90 80 70 60 50 40 30 20 10 0
f1 (ppm)

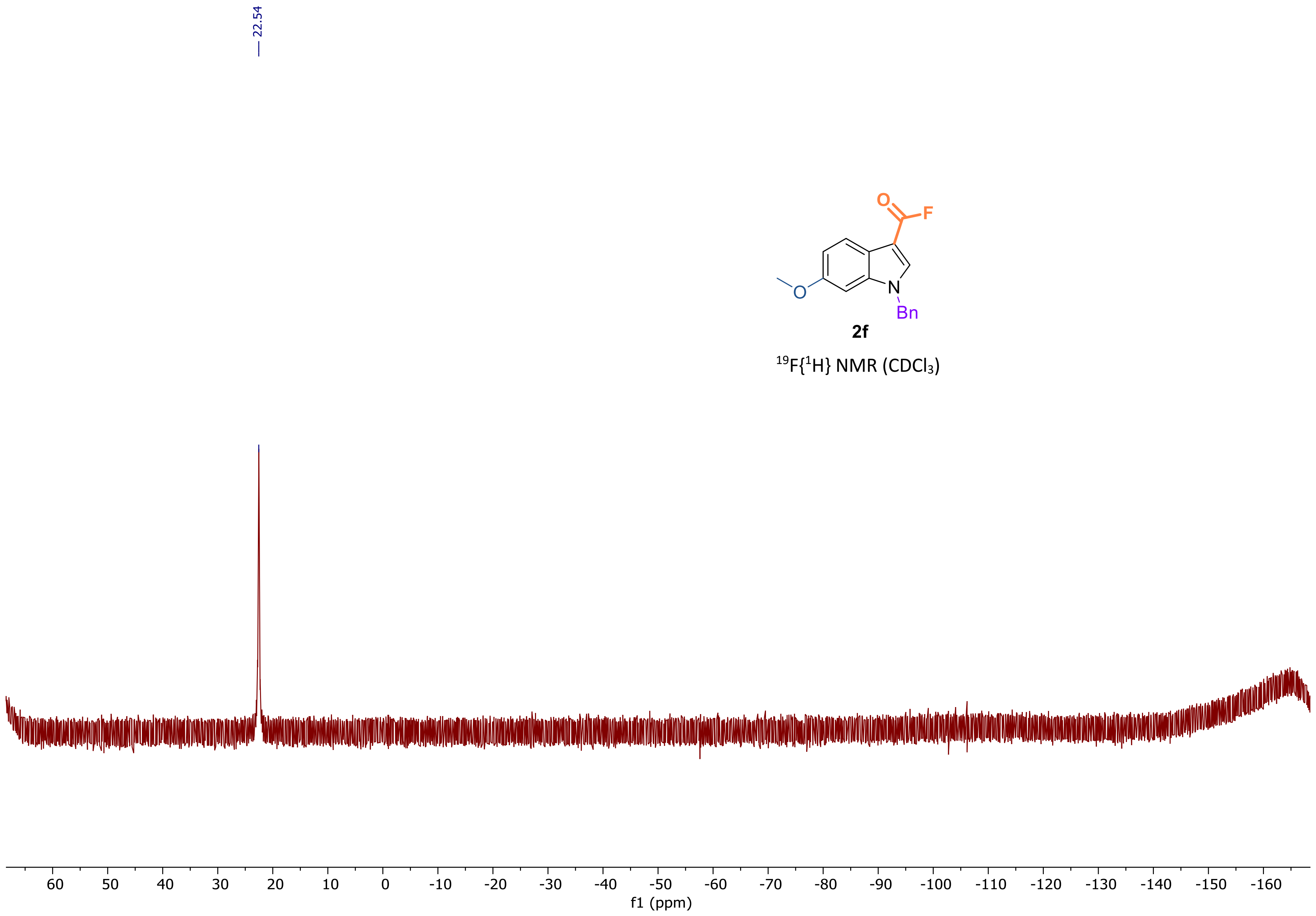
22.54
O
F
O
N
Bn
2f
19F{1H} NMR (CDCl3)
60 50 40 30 20 10 0 -10 -20 -30 -40 -50 -60 -70 -80 -90 -100 -110 -120 -130 -140 -150 -160
f1 (ppm)

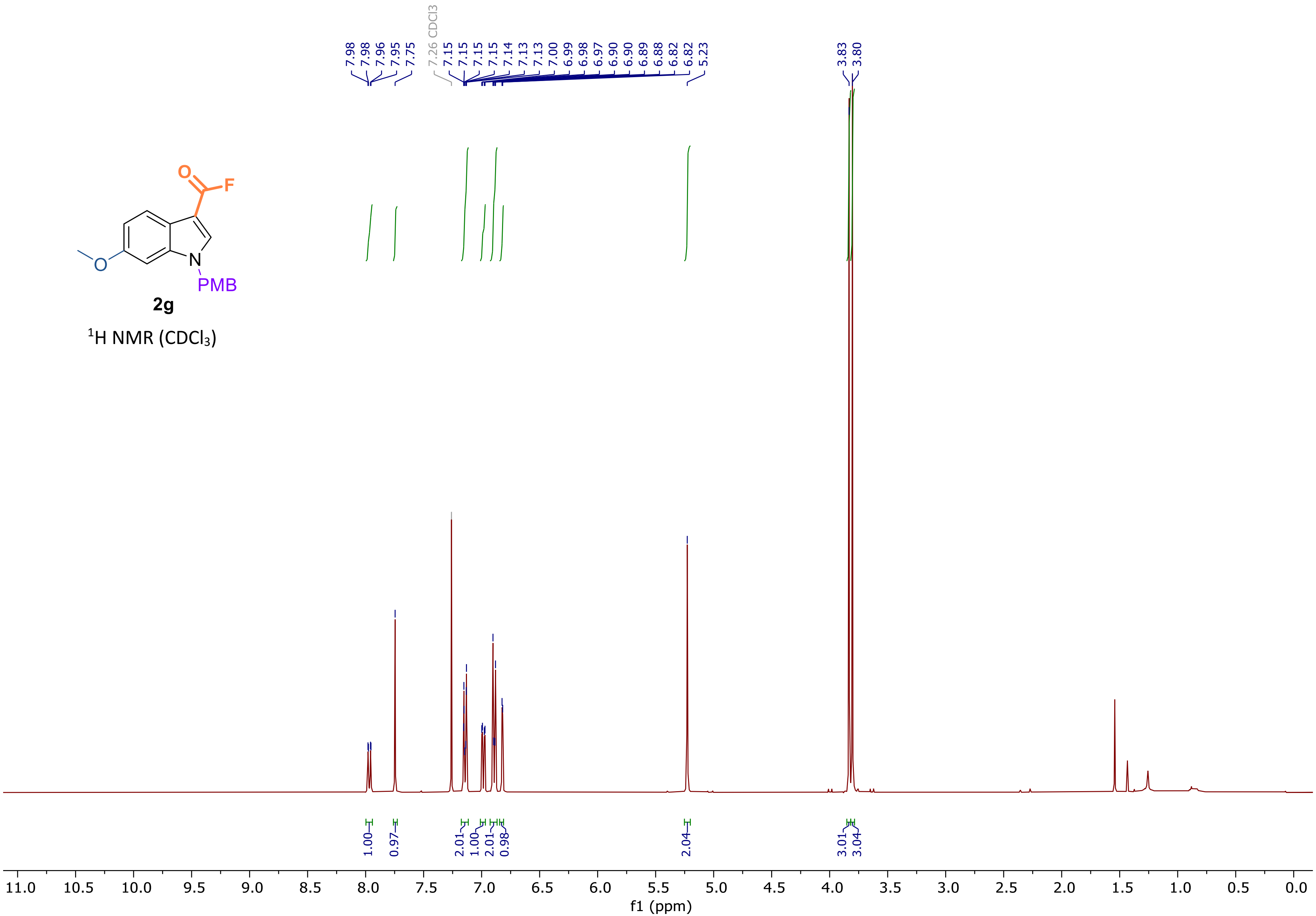
7.98 7.98 7.96 7.95 7.75 7.26 CDCl3 7.15 7.15 7.15 7.15 7.14 7.13 7.00 6.99 6.98 6.97 6.90 6.90 6.89 6.88 6.82 6.82 5.23 3.83 3.80
O
F
N
O
PMB
2g
^{1}H NMR ($CDCl_3$)
1.00 0.97 2.01 1.00 2.01 0.98 2.04 3.01 3.04
11.0 10.5 10.0 9.5 9.0 8.5 8.0 7.5 7.0 6.5 6.0 5.5 5.0 4.5 4.0 3.5 3.0 2.5 2.0 1.5 1.0 0.5 0.0
f1 (ppm)

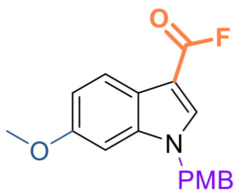

2g

$^{13}C\{^{1}H\}$ NMR ($CDCl_3$)

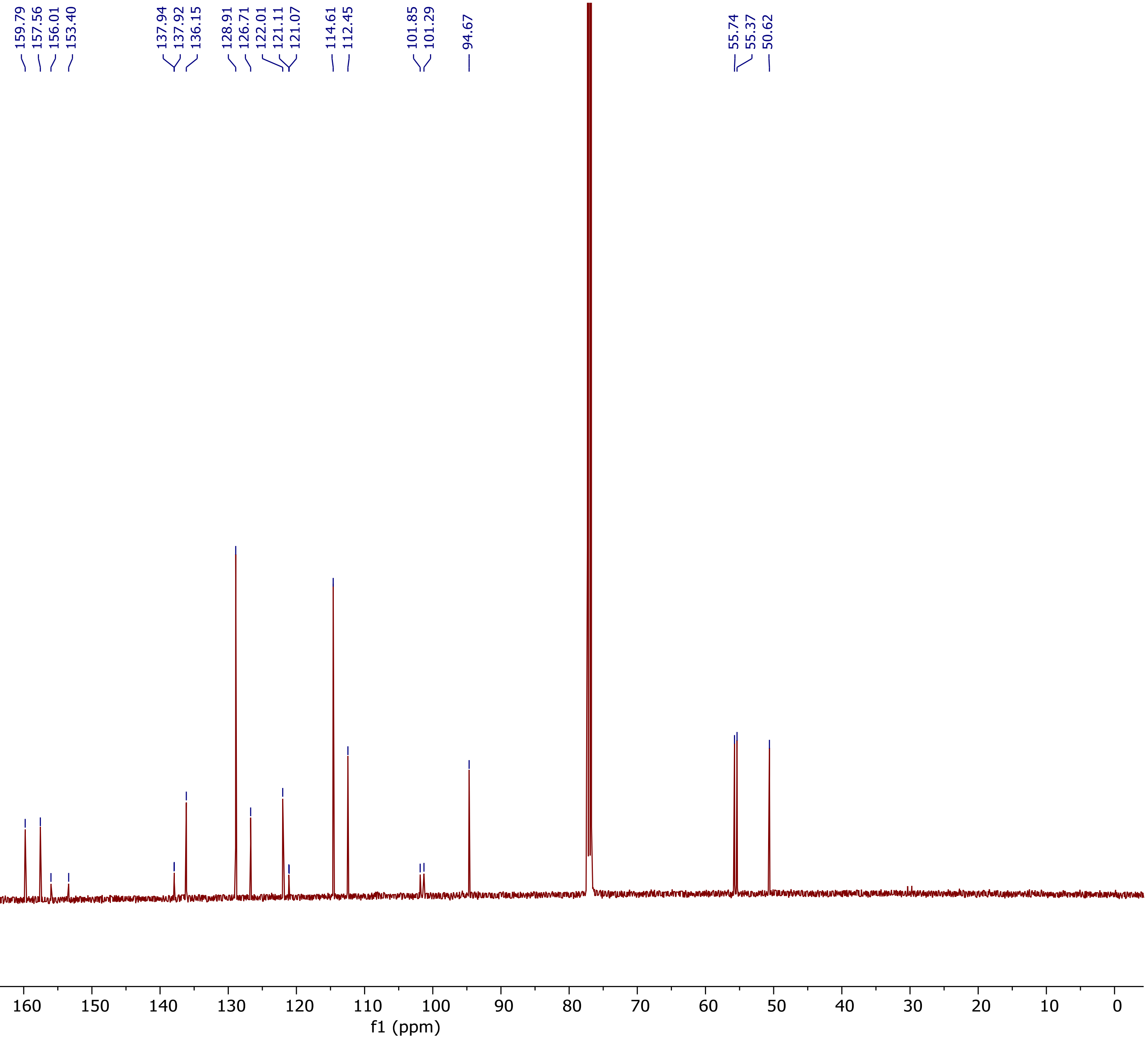

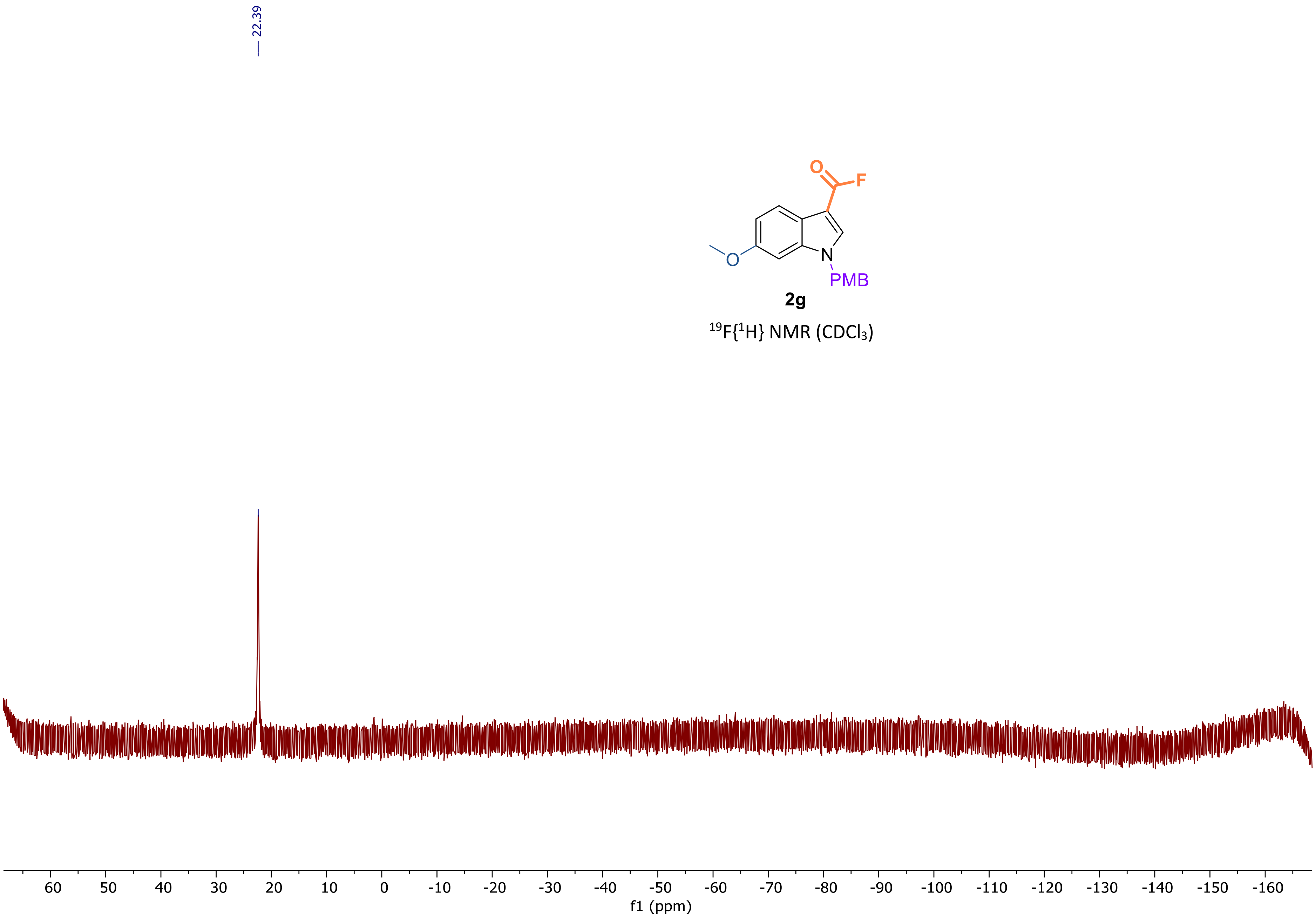

22.39
O
F
N
O
PMB
2g
19F{1H} NMR (CDCl3)
60 50 40 30 20 10 0 -10 -20 -30 -40 -50 -60 -70 -80 -90 -100 -110 -120 -130 -140 -150 -160
f1 (ppm)

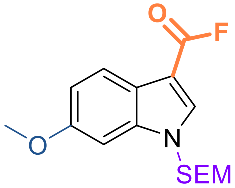

2h

^{1}H NMR ($CDCl_3$)

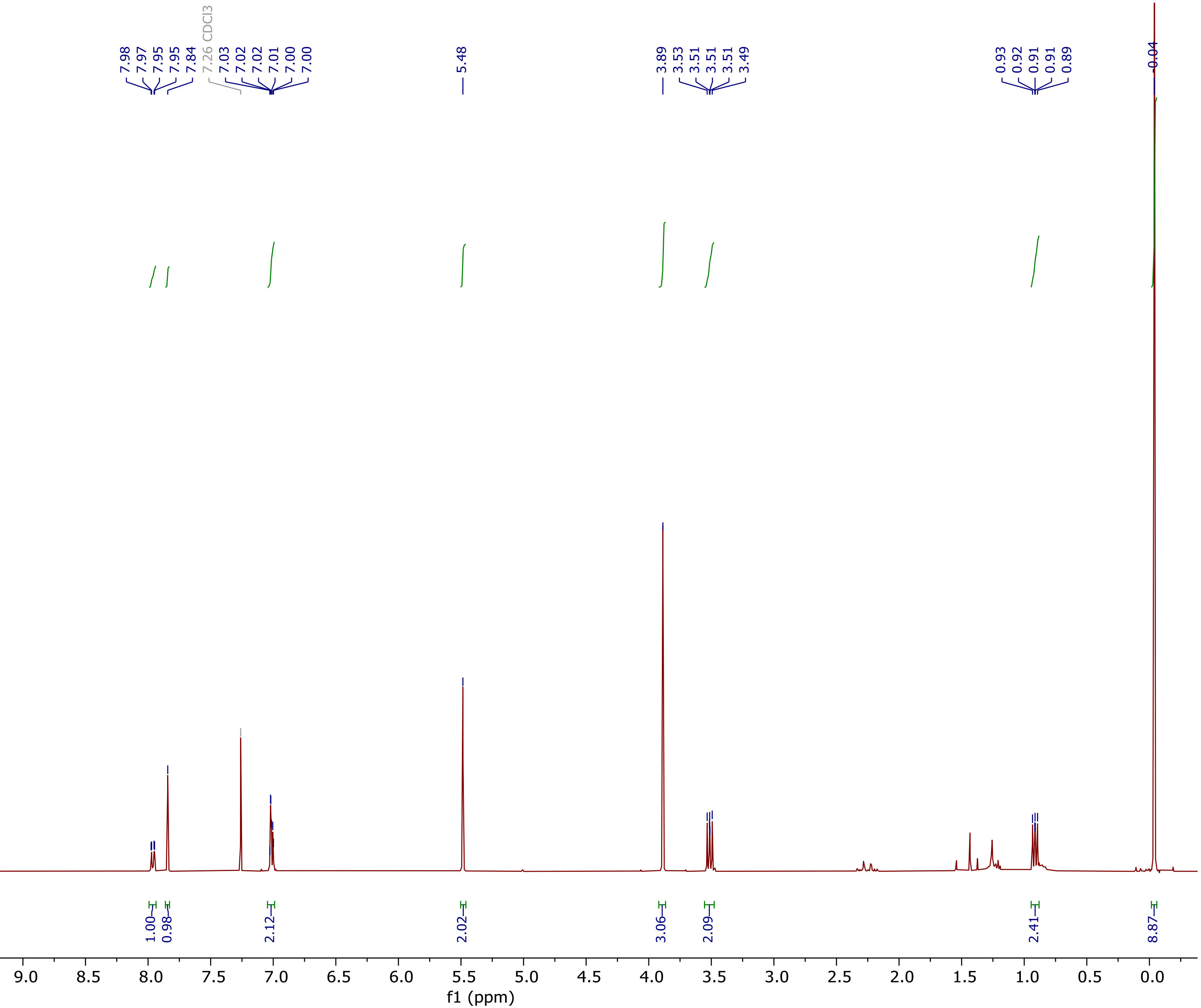

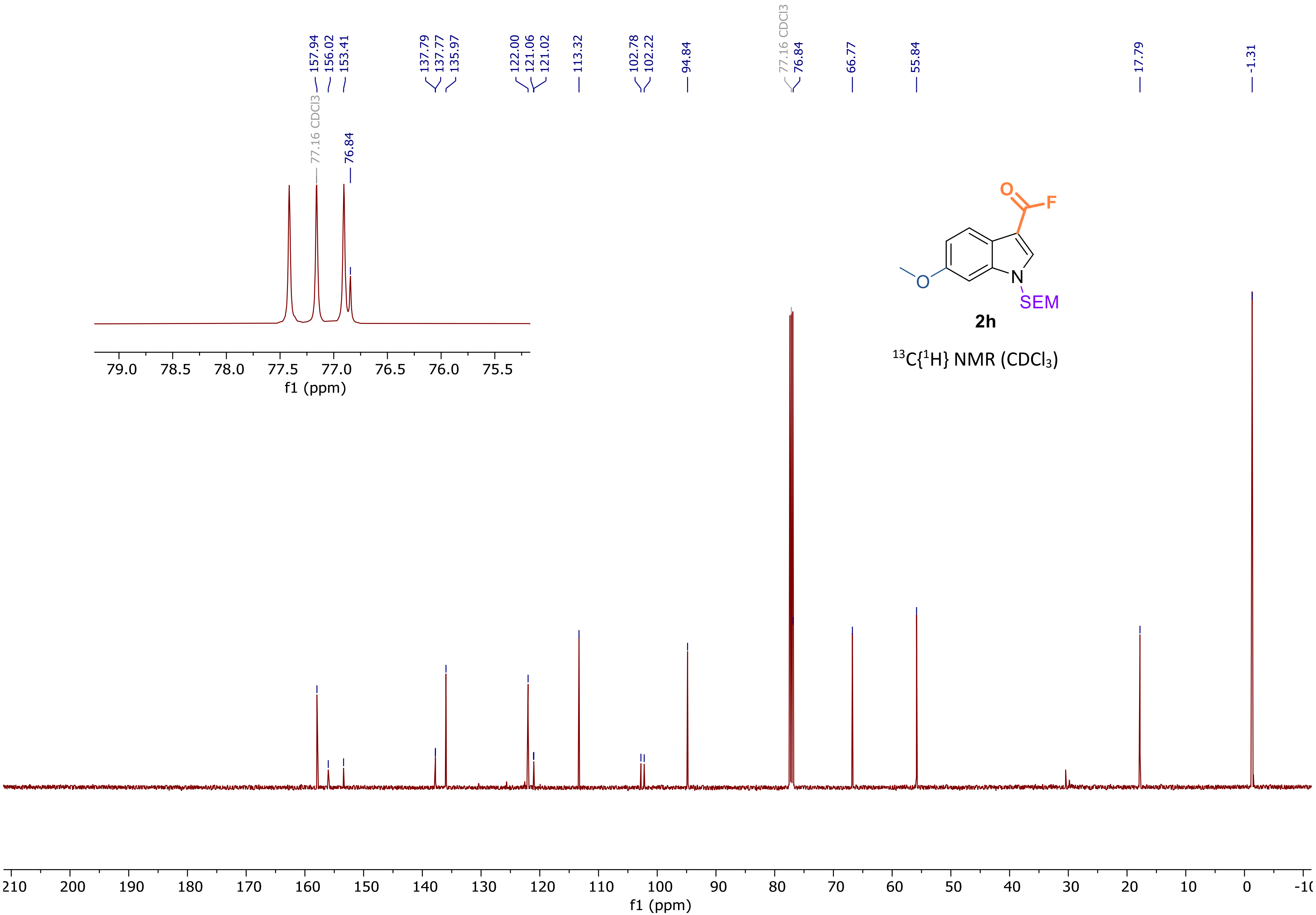
157.94
156.02
153.41
137.79
137.77
135.97
122.00
121.06
121.02
113.32
102.78
102.22
94.84
77.16 CDCl3
76.84
66.77
55.84
17.79
-1.31
77.16 CDCl3
76.84
79.0 78.5 78.0 77.5 77.0 76.5 76.0 75.5
f1 (ppm)
O
F
O
N
SEM
2h
13C{1H} NMR (CDCl3)
210 200 190 180 170 160 150 140 130 120 110 100 90 80 70 60 50 40 30 20 10 0 -10
f1 (ppm)

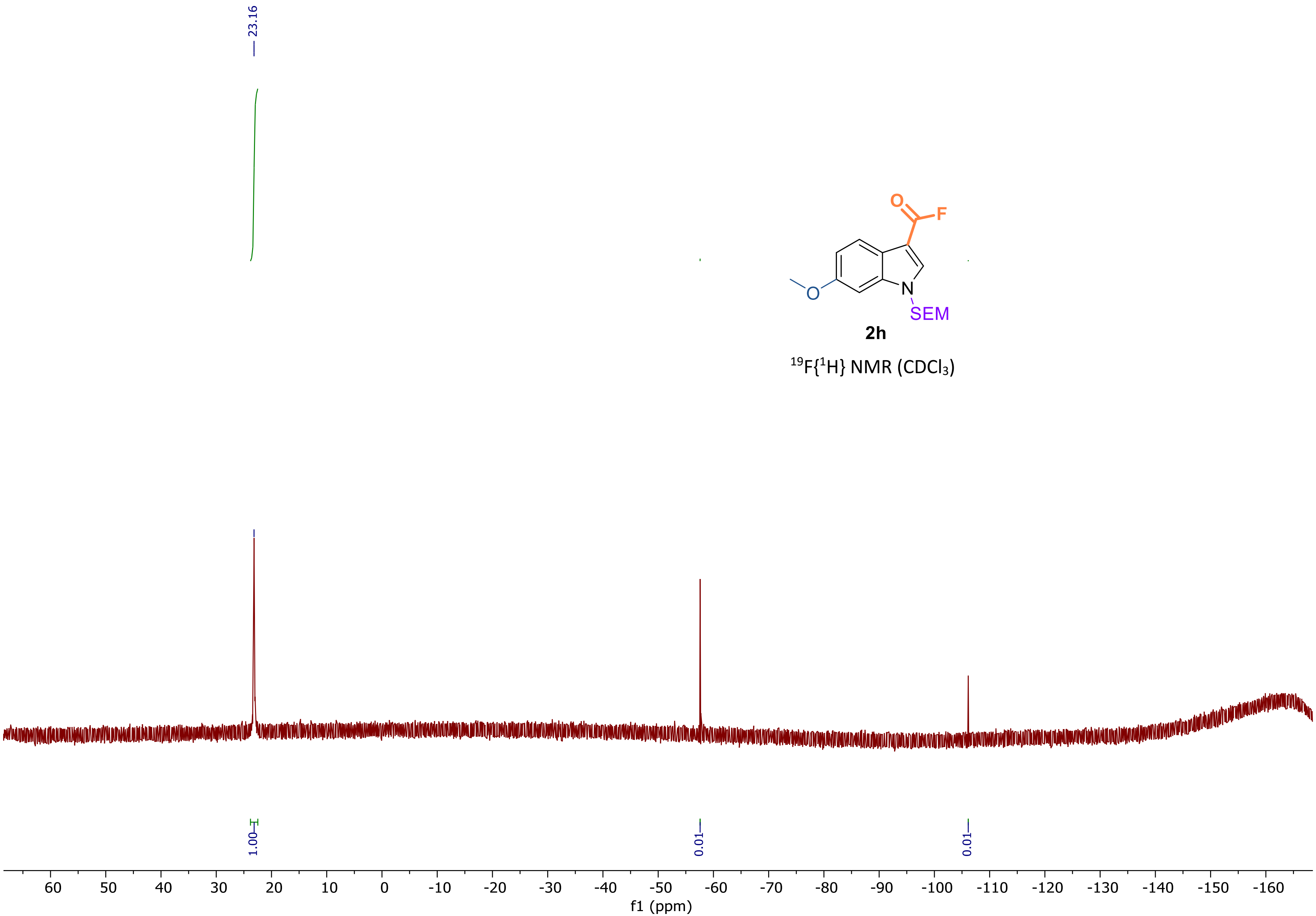
23.16
O
F
O
N
SEM
2h
19F{1H} NMR (CDCl3)
1.00
0.01
0.01
60 50 40 30 20 10 0 -10 -20 -30 -40 -50 -60 -70 -80 -90 -100 -110 -120 -130 -140 -150 -160
f1 (ppm)

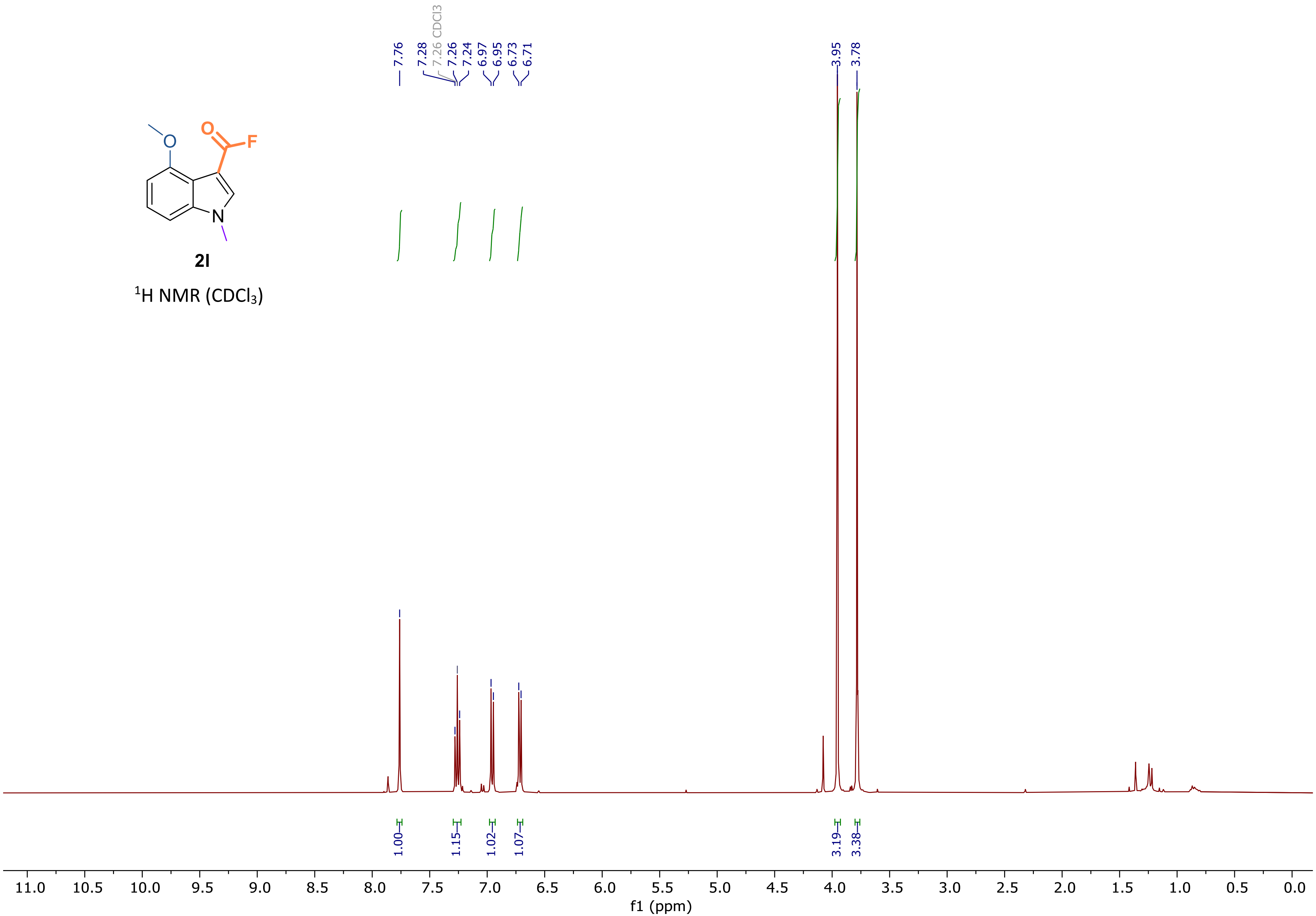

7.76
7.28
7.26 CDCl3
7.26
7.24
6.97
6.95
6.73
6.71
3.95
3.78
2l
^{1}H NMR ($CDCl_3$)
1.00
1.15
1.02
1.07
3.19
3.38
11.0 10.5 10.0 9.5 9.0 8.5 8.0 7.5 7.0 6.5 6.0 5.5 5.0 4.5 4.0 3.5 3.0 2.5 2.0 1.5 1.0 0.5 0.0
f1 (ppm)

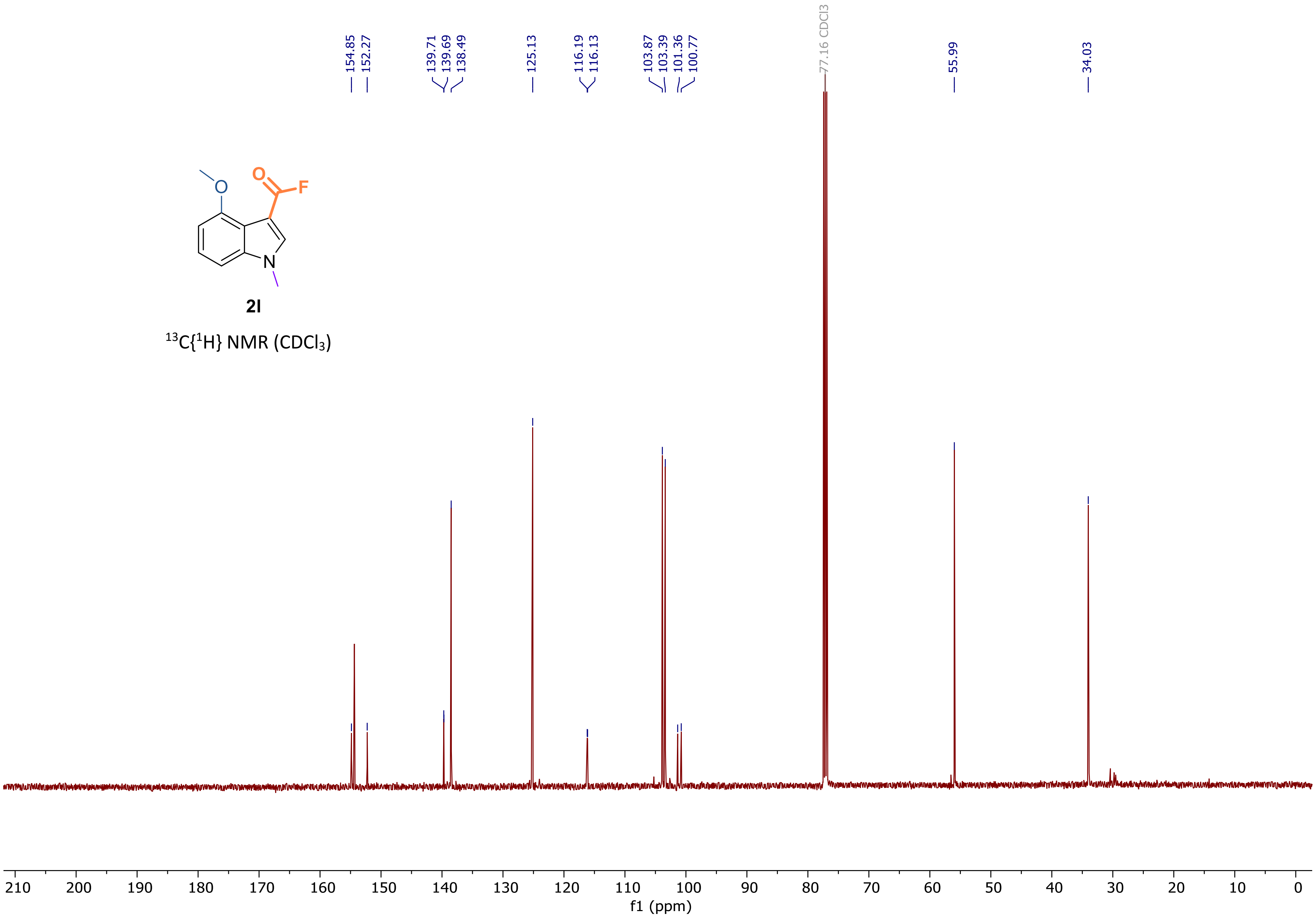

154.85
152.27
139.71
139.69
138.49
125.13
116.19
116.13
103.87
103.39
101.36
100.77
77.16 CDCl3
55.99
34.03
O
F
O
N
2l
$^{13}C\{^{1}H\}$ NMR ($CDCl_3$)
210 200 190 180 170 160 150 140 130 120 110 100 90 80 70 60 50 40 30 20 10 0
f1 (ppm)

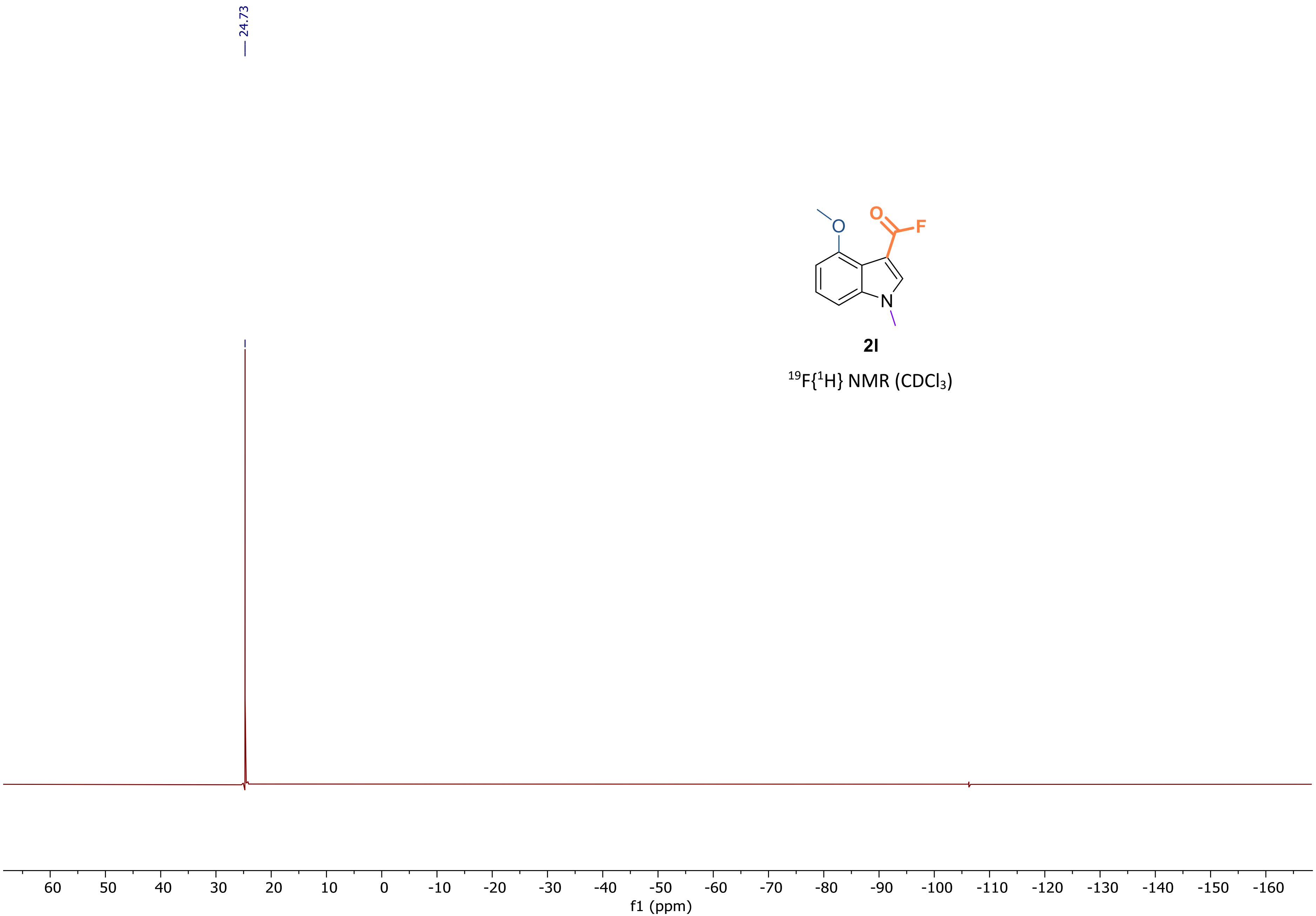

2l

$^{19}F\{^{1}H\}$ NMR ($CDCl_3$)

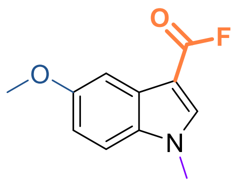

2m

^{1}H NMR ($CDCl_3$)

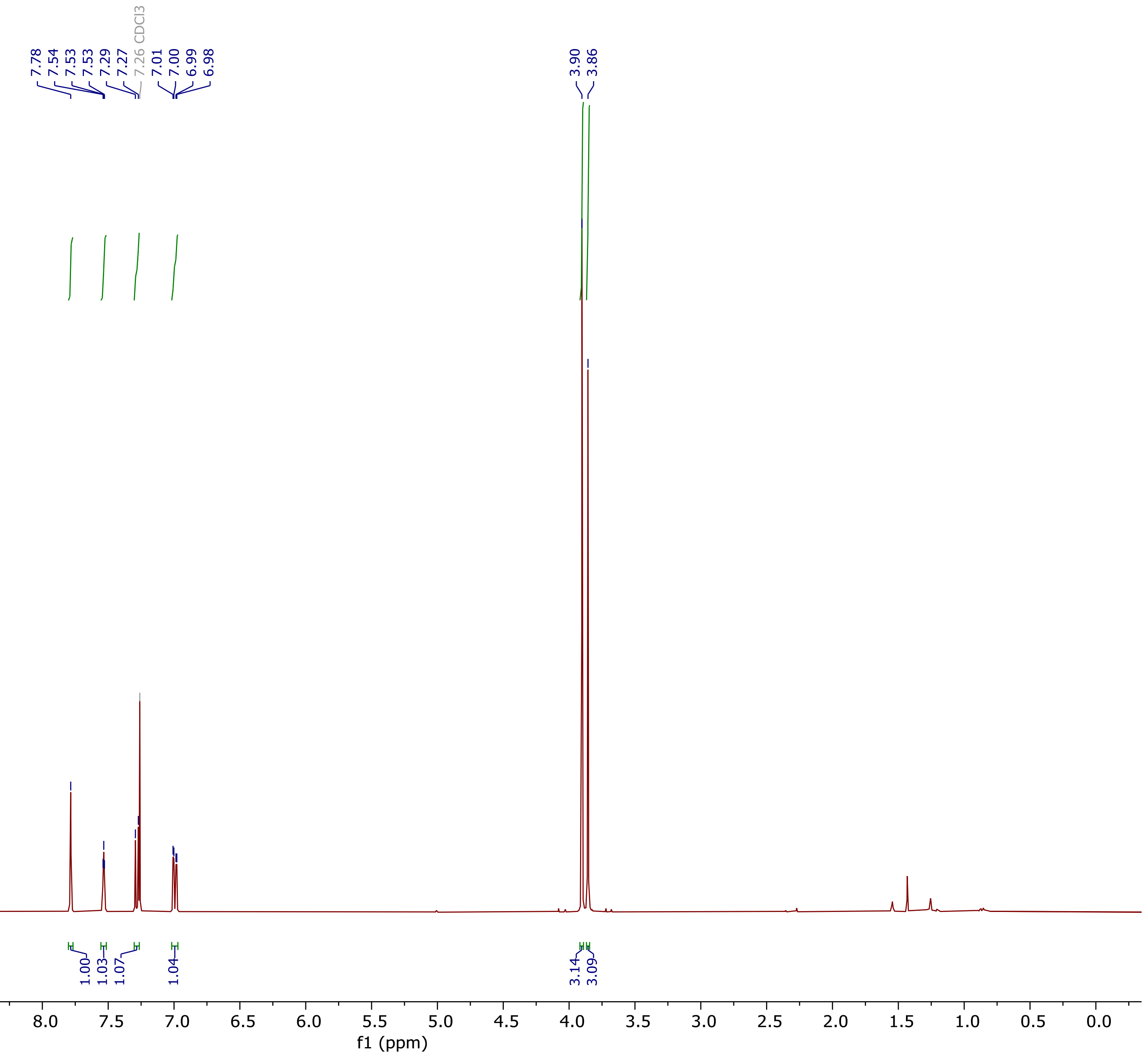

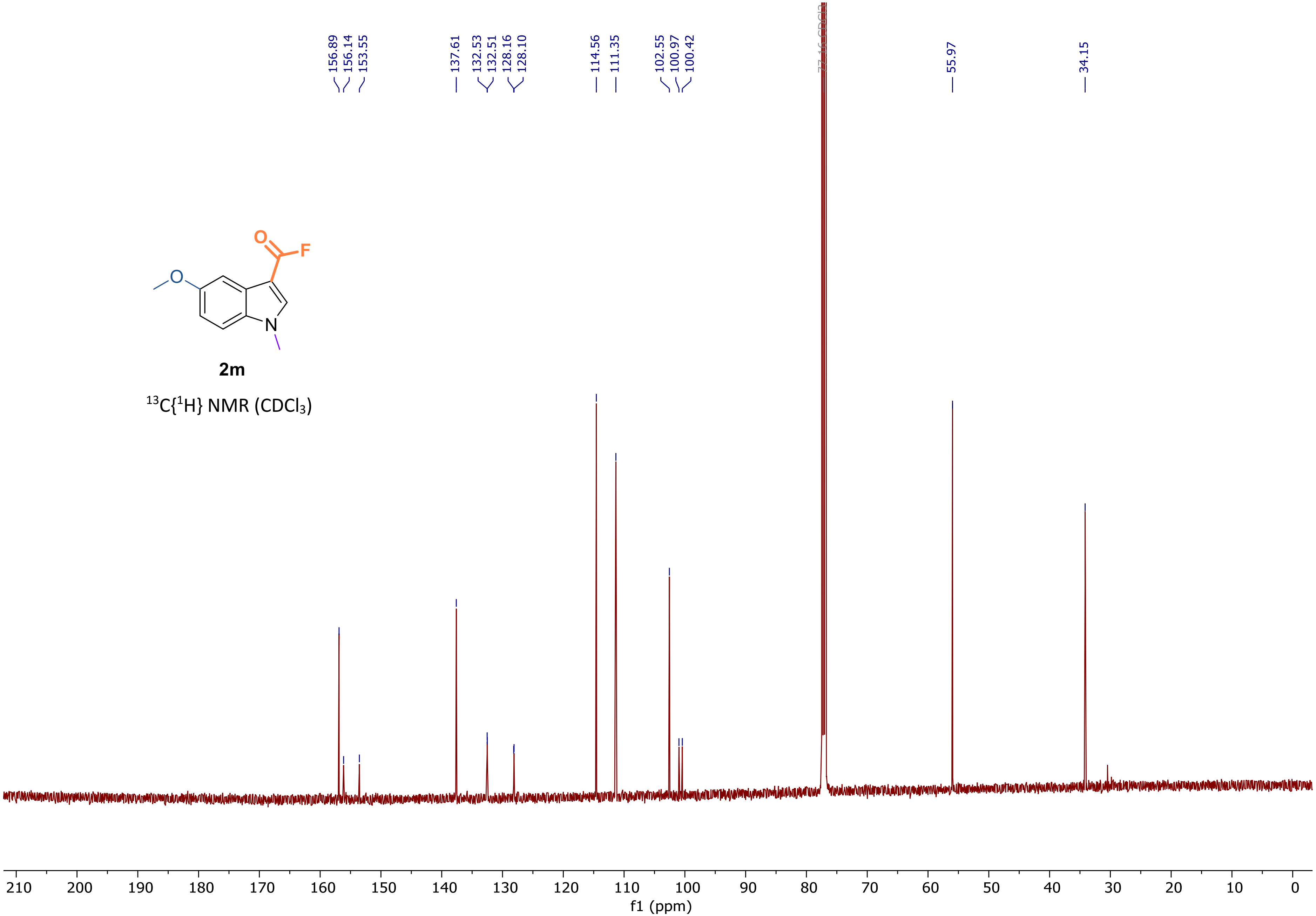
156.89
156.14
153.55
137.61
132.53
132.51
128.16
128.10
114.56
111.35
102.55
100.97
100.42
77.16 CDCl3
55.97
34.15
O
F
O
N
2m
$^{13}C\{^{1}H\}$ NMR ($CDCl_3$)
210 200 190 180 170 160 150 140 130 120 110 100 90 80 70 60 50 40 30 20 10 0
f1 (ppm)

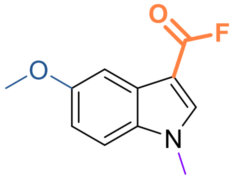

2m

$^{19}F\{^{1}H\}$ NMR ($CDCl_3$)

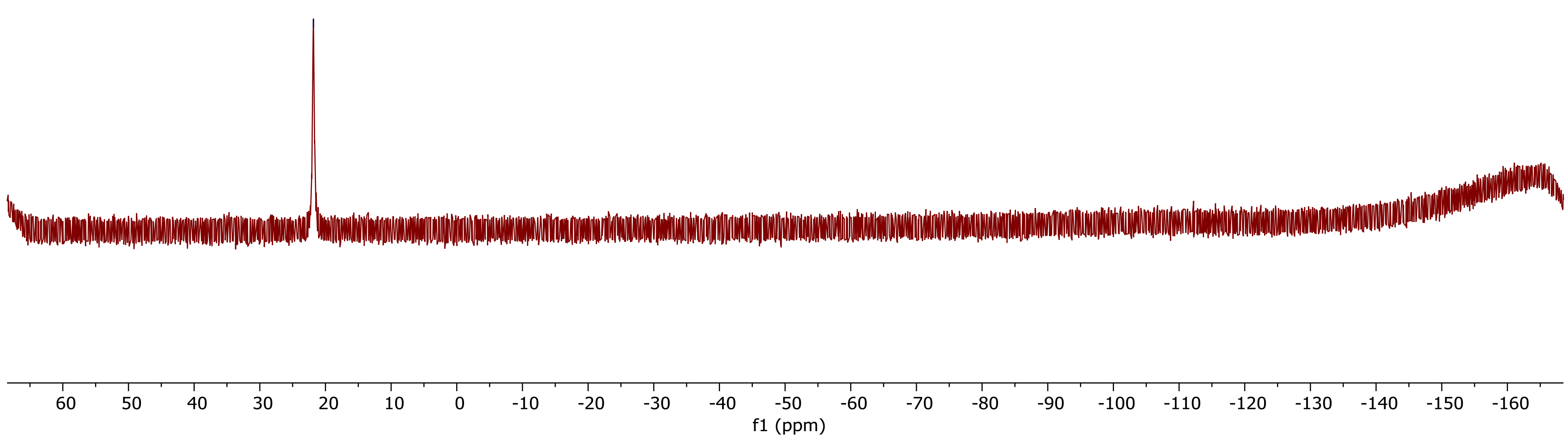

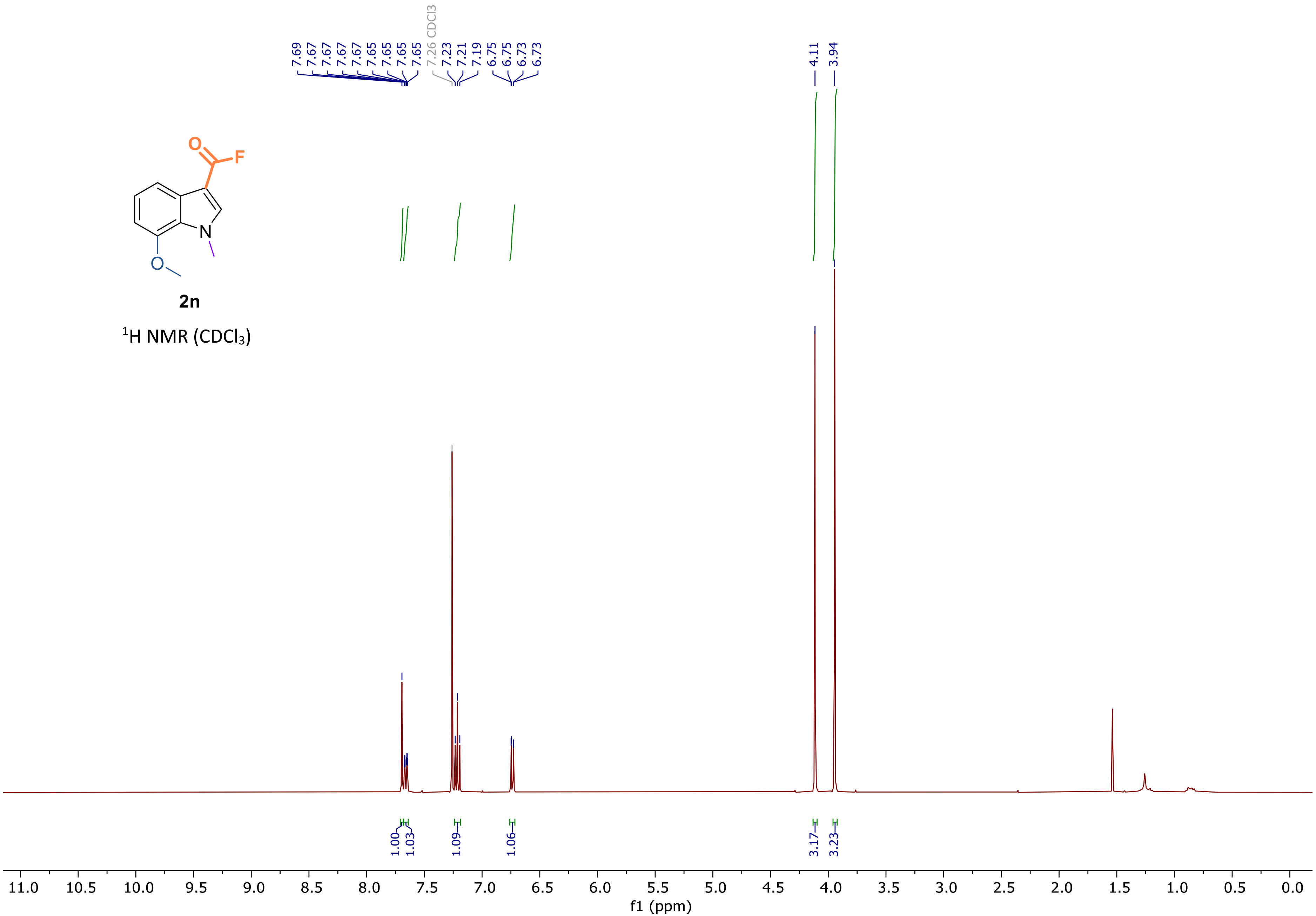
7.69
7.67
7.67
7.67
7.67
7.65
7.65
7.65
7.65
7.26 CDCl3
7.23
7.21
7.21
7.19
6.75
6.75
6.73
6.73
4.11
3.94
O
F
N
O
2n
1H NMR (CDCl3)
1.00
1.03
1.09
1.06
3.17
3.23
11.0
10.5
10.0
9.5
9.0
8.5
8.0
7.5
7.0
6.5
6.0
5.5
5.0
4.5
4.0
3.5
3.0
2.5
2.0
1.5
1.0
0.5
0.0
f1 (ppm)

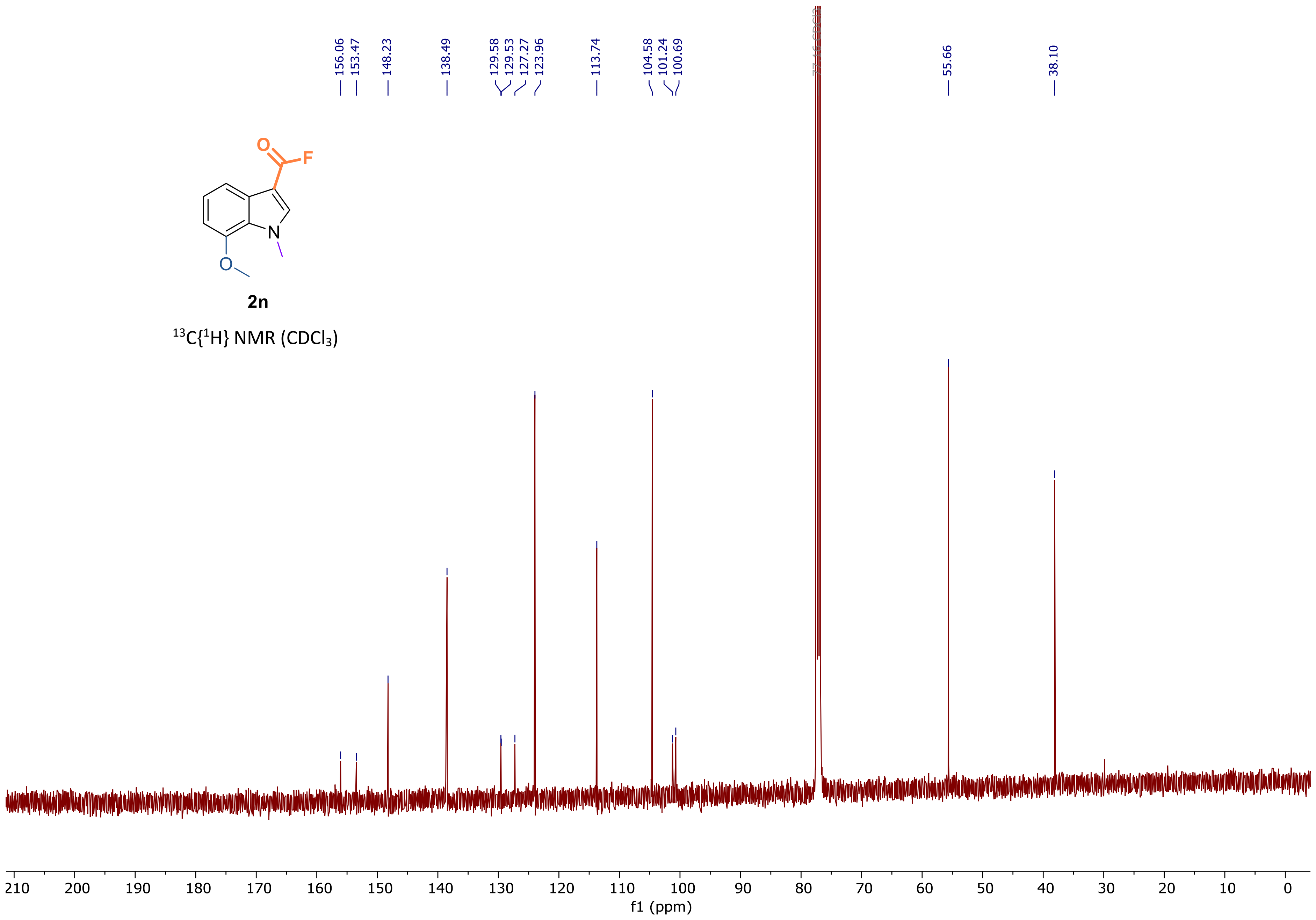
156.06
153.47
148.23
138.49
129.58
129.53
127.27
123.96
113.74
104.58
101.24
100.69
55.66
38.10
O
F
N
O
2n
$^{13}C\{^1H\}$ NMR ($CDCl_3$)
210 200 190 180 170 160 150 140 130 120 110 100 90 80 70 60 50 40 30 20 10 0
f1 (ppm)

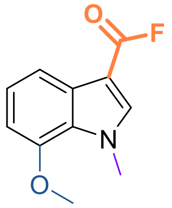

2n

$^{19}F\{^{1}H\}$ NMR ($CDCl_3$)

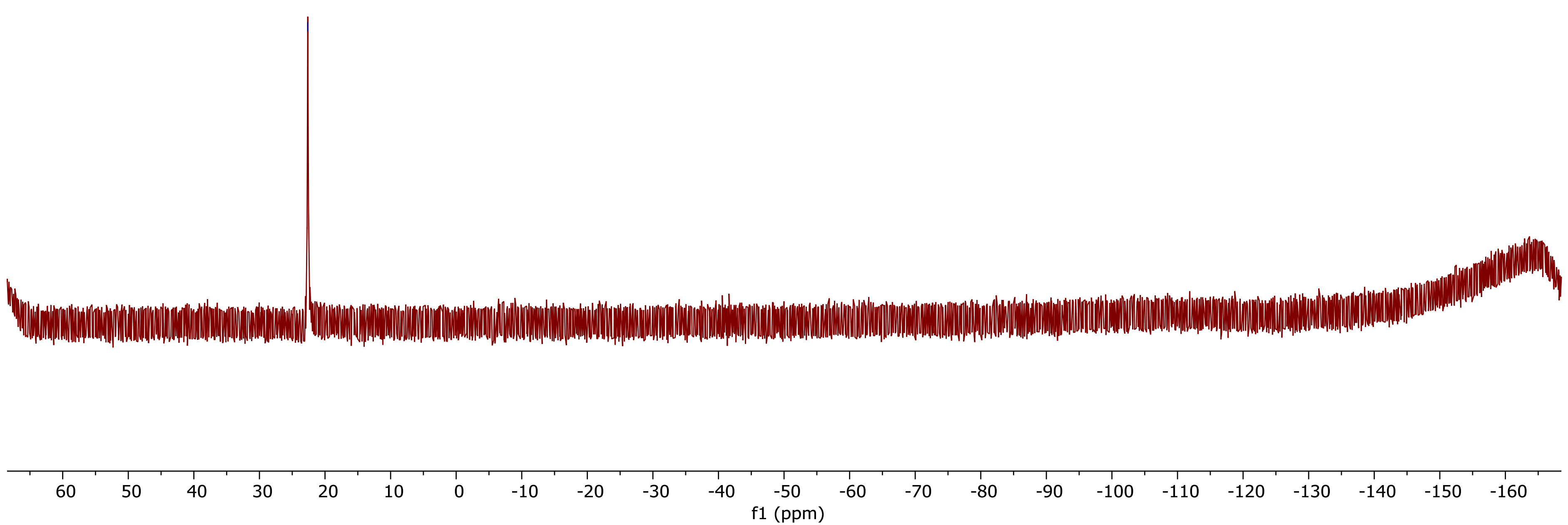

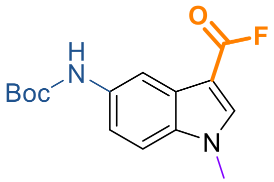

2o

^{1}H NMR ($CDCl_3$)

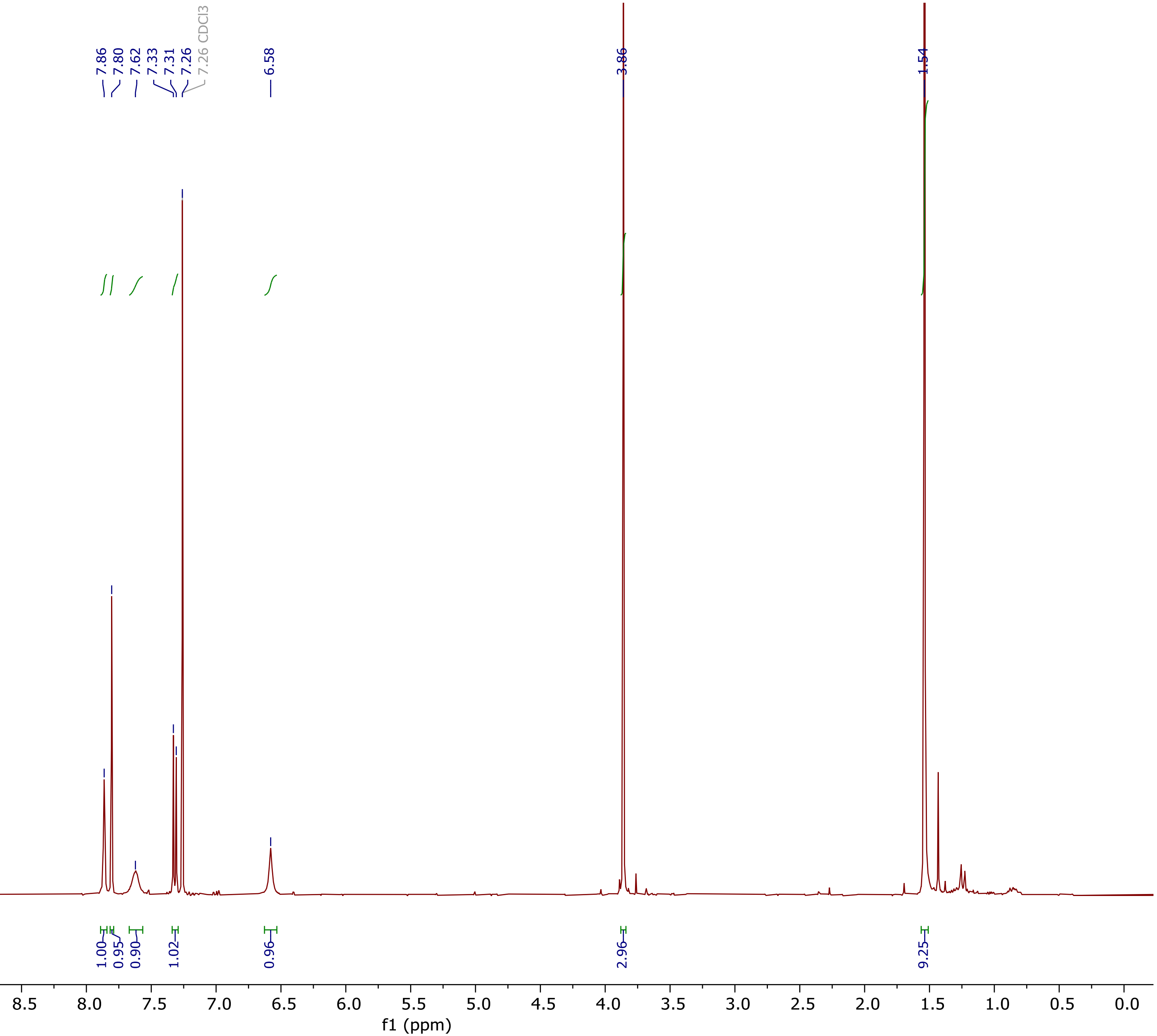

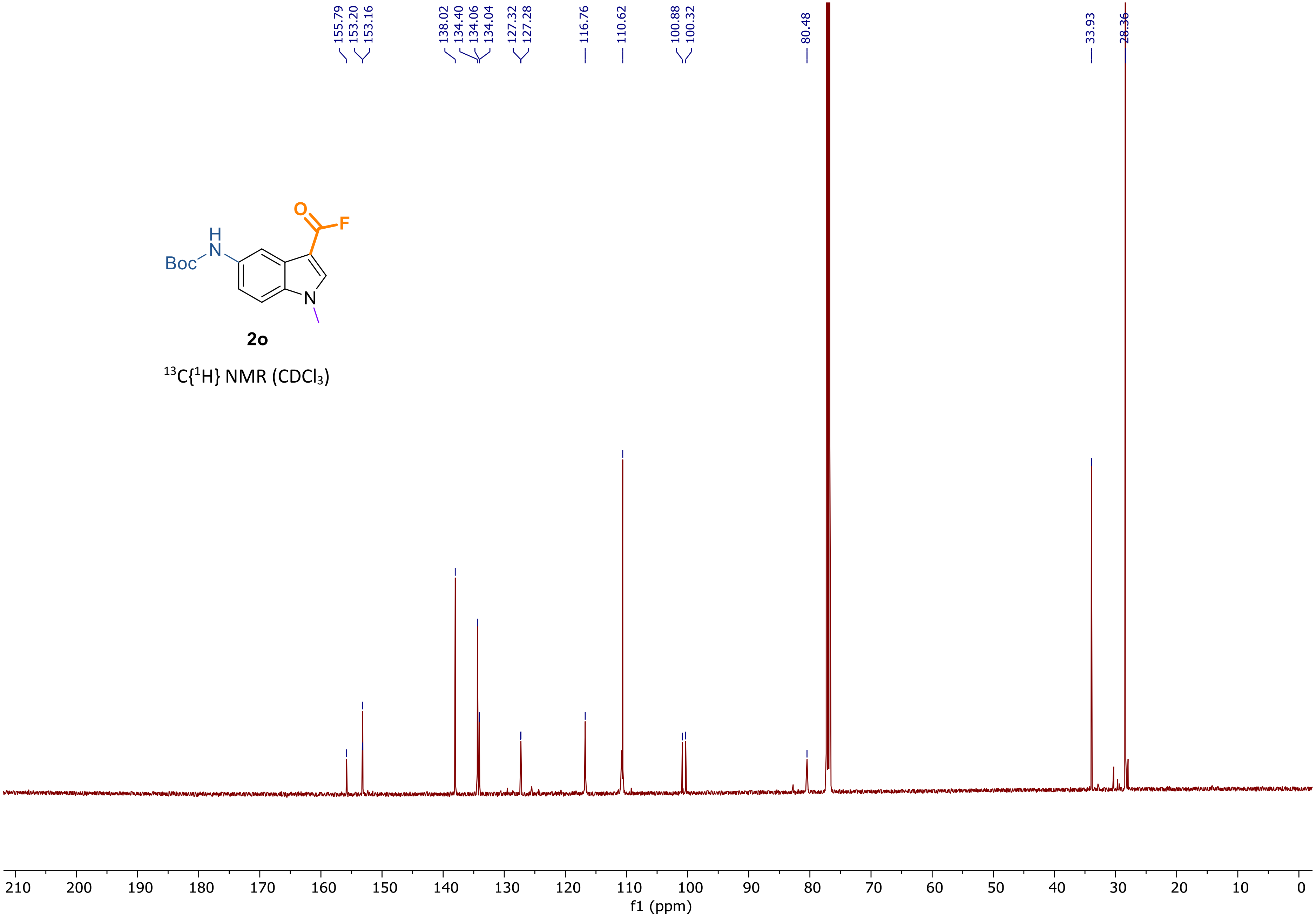
155.79
153.20
153.16
138.02
134.40
134.06
134.04
127.32
127.28
116.76
110.62
100.88
100.32
80.48
33.93
28.36
Boc
H
N
O
F
N
2o
$^{13}C\{^{1}H\}$ NMR ($CDCl_3$)
210
200
190
180
170
160
150
140
130
120
110
100
90
80
70
60
50
40
30
20
10
0
f1 (ppm)

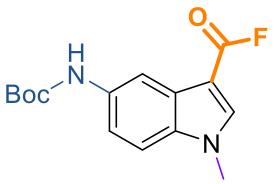

2o

$^{19}F\{^{1}H\}$ NMR ($CDCl_3$)

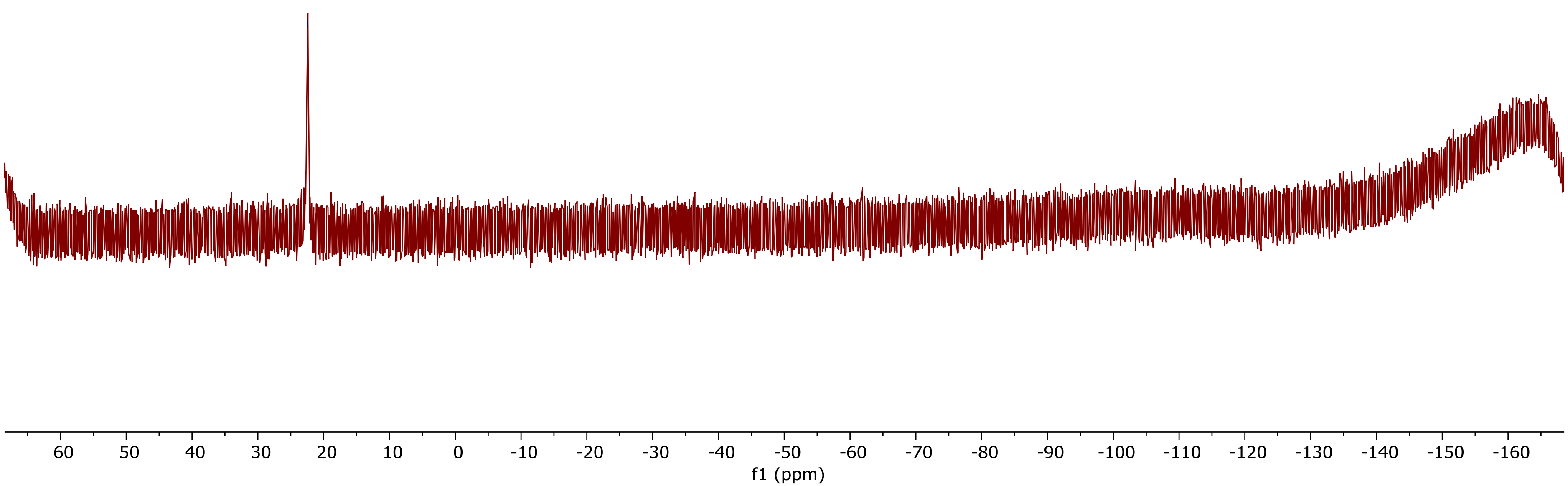

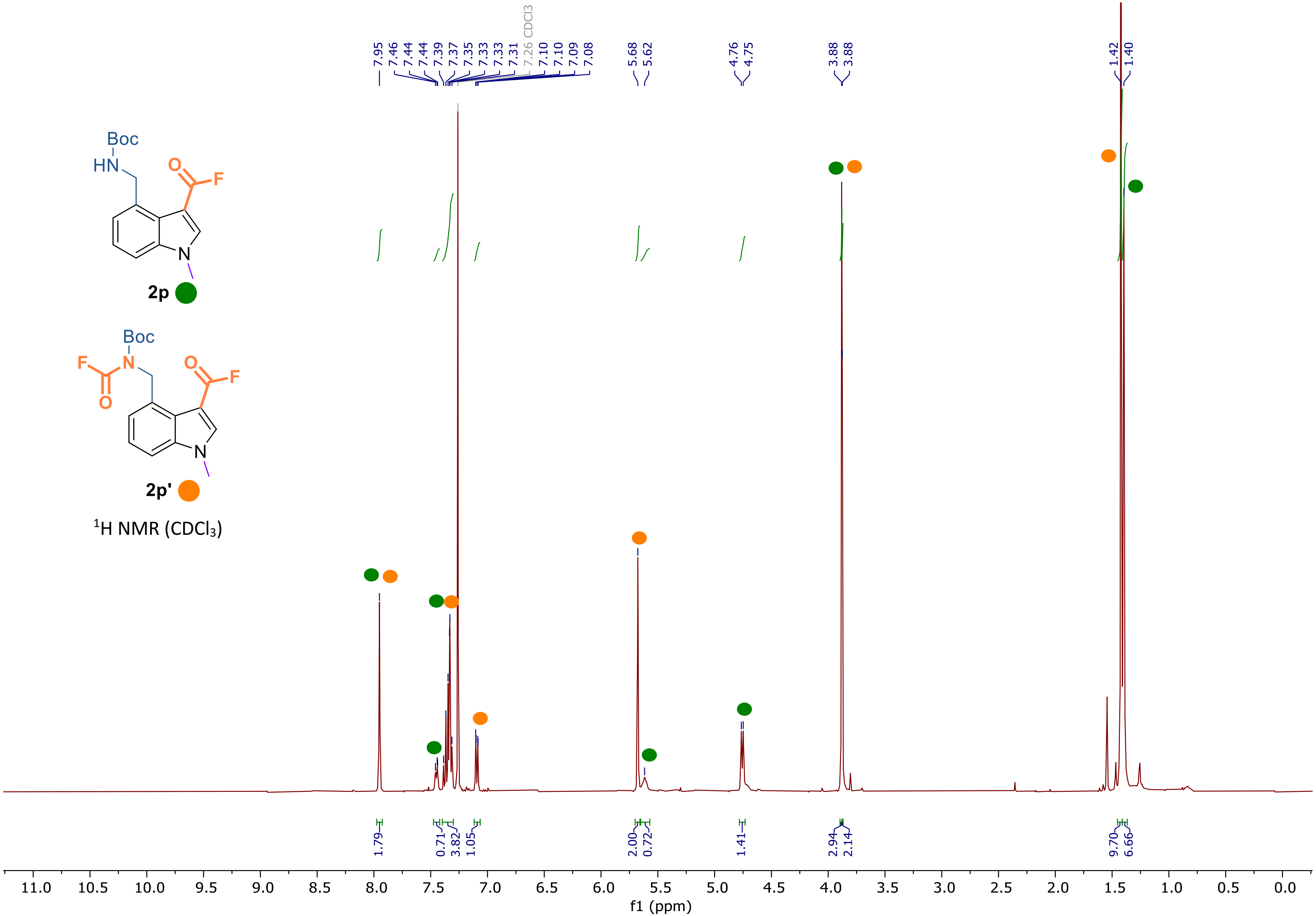

Boc
HN
O
F
N
2p
Boc
F
N
O
O
F
N
2p'
1H NMR (CDCl3)
7.95
7.46
7.44
7.44
7.39
7.37
7.35
7.33
7.33
7.31
7.26 CDCl3
7.10
7.10
7.09
7.08
5.68
5.62
4.76
4.75
3.88
3.88
1.42
1.40
1.79
0.71
3.82
1.05
2.00
0.72
1.41
2.94
2.14
9.70
6.66
11.0
10.5
10.0
9.5
9.0
8.5
8.0
7.5
7.0
6.5
6.0
5.5
5.0
4.5
4.0
3.5
3.0
2.5
2.0
1.5
1.0
0.5
0.0
f1 (ppm)

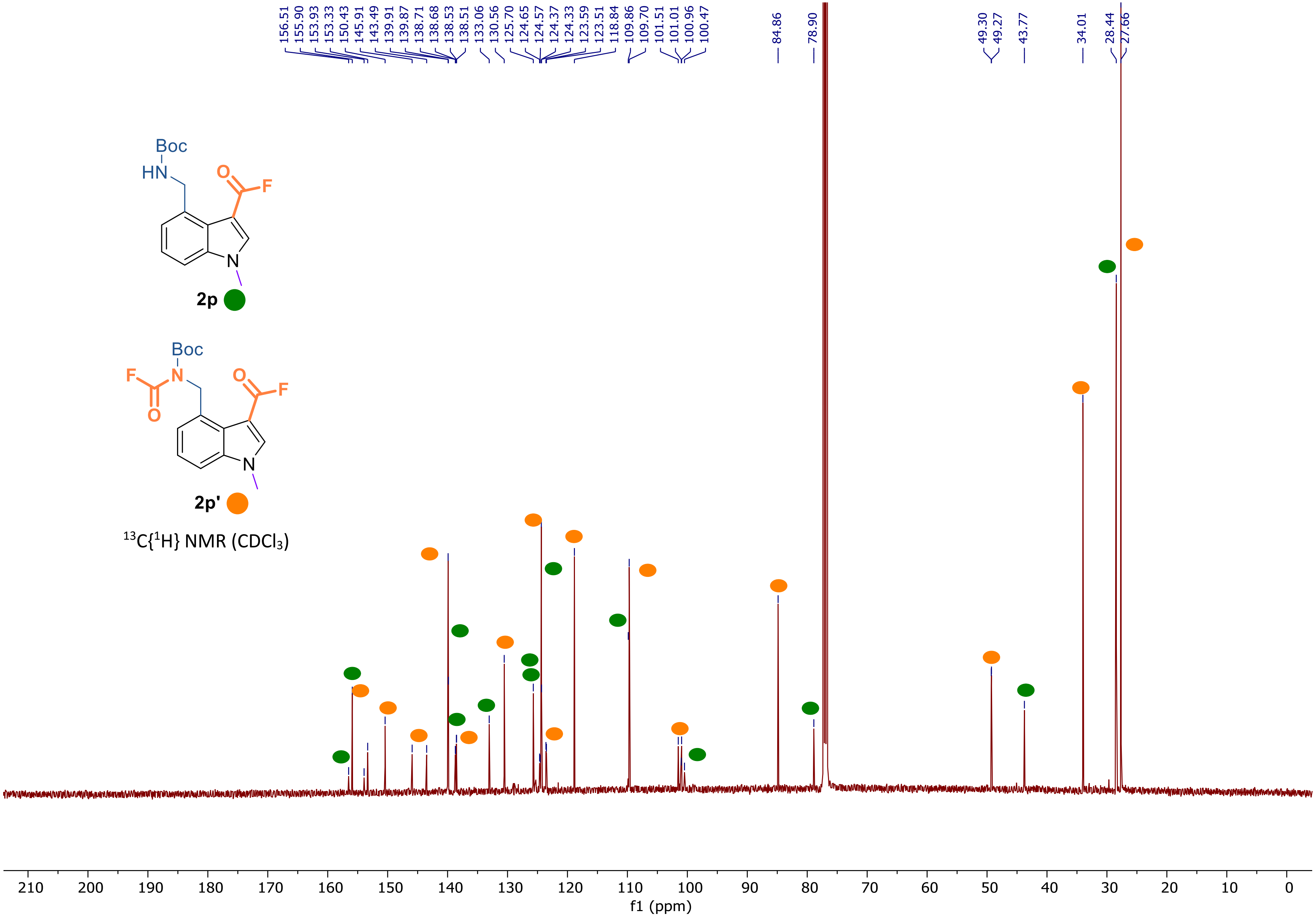

156.51
155.90
153.93
153.33
150.43
145.91
143.49
139.91
139.87
138.71
138.68
138.53
138.51
133.06
130.56
125.70
124.65
124.57
124.37
124.33
123.59
123.51
118.84
109.86
109.70
101.51
101.01
100.96
100.47
84.86
78.90
49.30
49.27
43.77
34.01
28.44
27.66
Boc
HN
O
F
N
2p
Boc
F
N
O
O
F
N
2p'
$^{13}C\{^1H\}$ NMR ($CDCl_3$)
210 200 190 180 170 160 150 140 130 120 110 100 90 80 70 60 50 40 30 20 10 0
f1 (ppm)

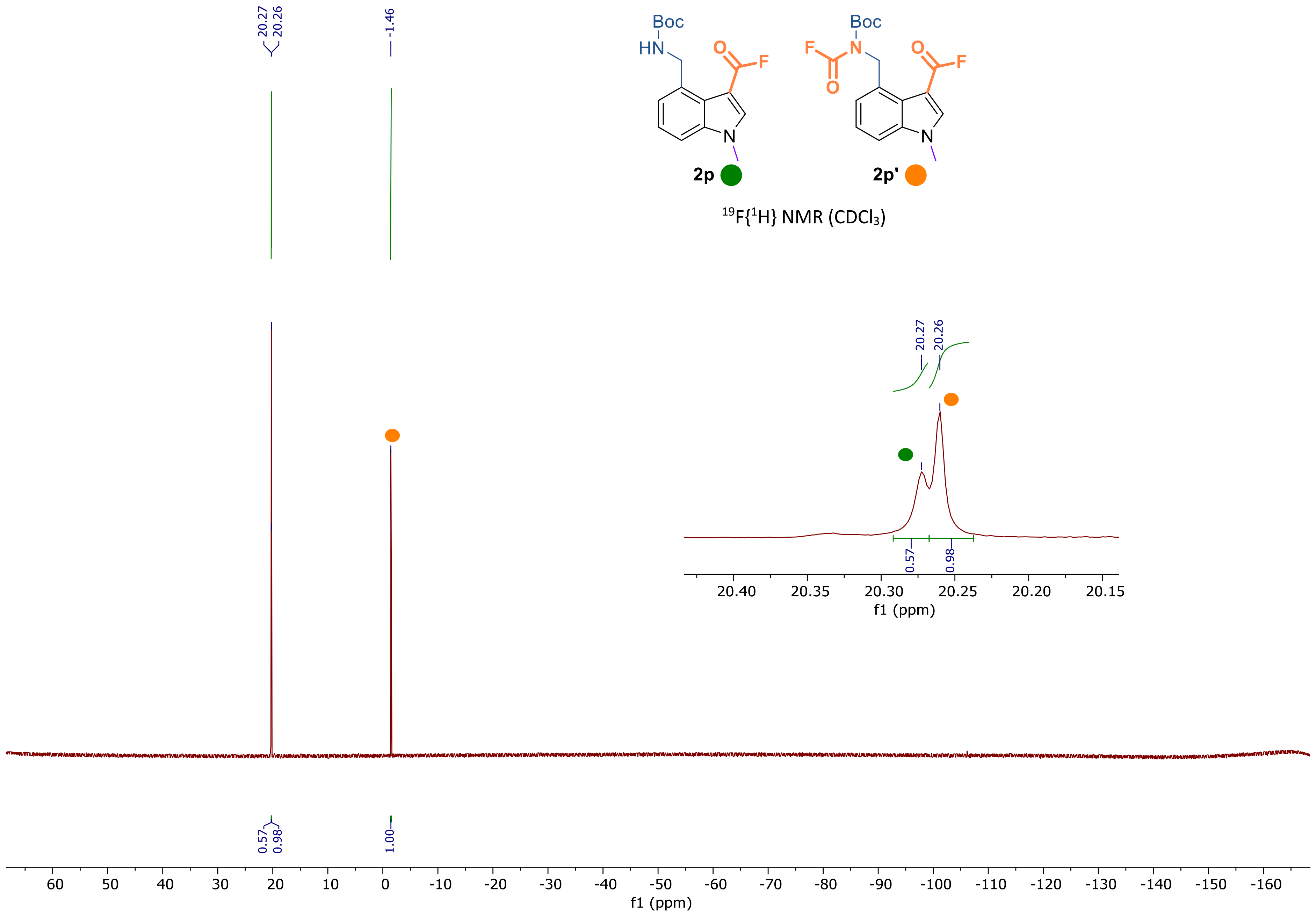

20.27
20.26
-1.46
Boc
HN
O
F
N
2p
Boc
F
N
O
O
F
N
2p'
$^{19}F\{^{1}H\}$ NMR ($CDCl_3$)
20.27
20.26
0.57
0.98
20.40
20.35
20.30
20.25
20.20
20.15
f1 (ppm)
0.57
0.98
1.00
60
50
40
30
20
10
0
-10
-20
-30
-40
-50
-60
-70
-80
-90
-100
-110
-120
-130
-140
-150
-160
f1 (ppm)

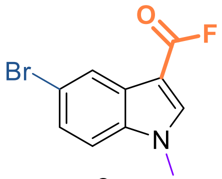

2q

^{1}H NMR ($CDCl_3$)

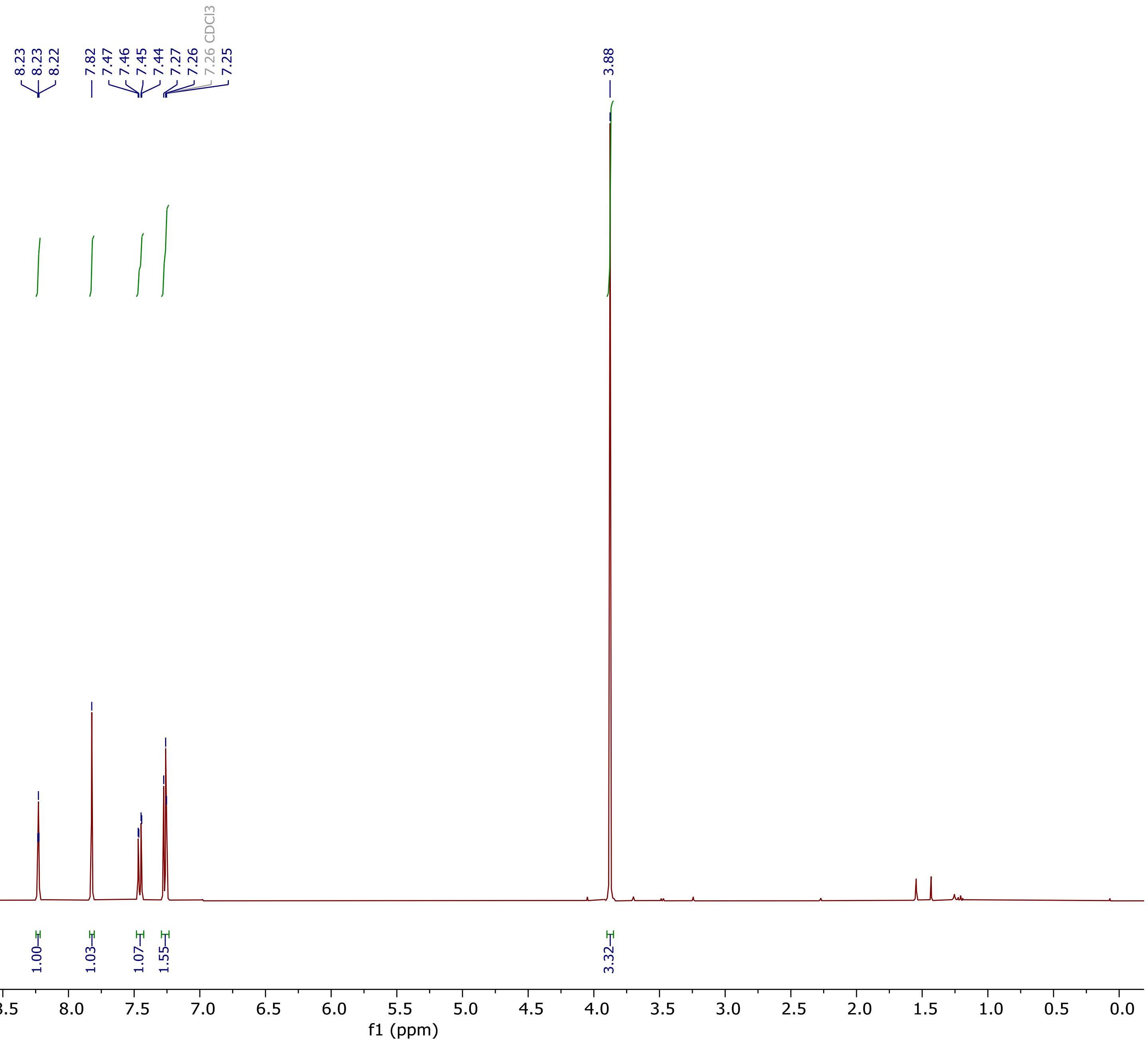

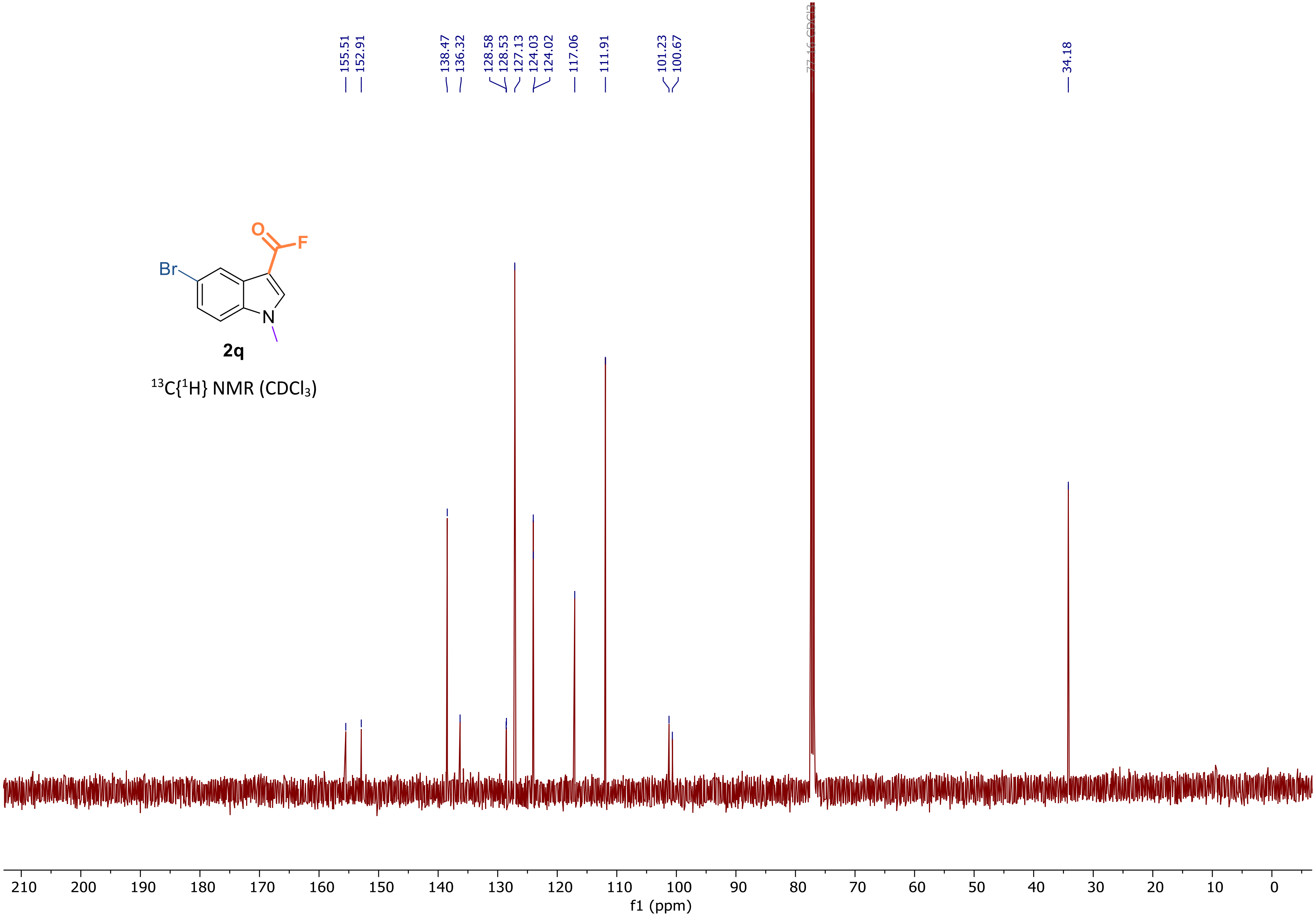
155.51
152.91
138.47
136.32
128.58
128.53
127.13
124.03
124.02
117.06
111.91
101.23
100.67
77.16 CDCl3
34.18
Br
O
F
N
2q
$^{13}C\{^1H\}$ NMR ($CDCl_3$)
210 200 190 180 170 160 150 140 130 120 110 100 90 80 70 60 50 40 30 20 10 0
f1 (ppm)

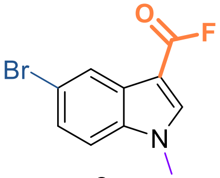

2q

$^{19}F\{^{1}H\}$ NMR ($CDCl_3$)

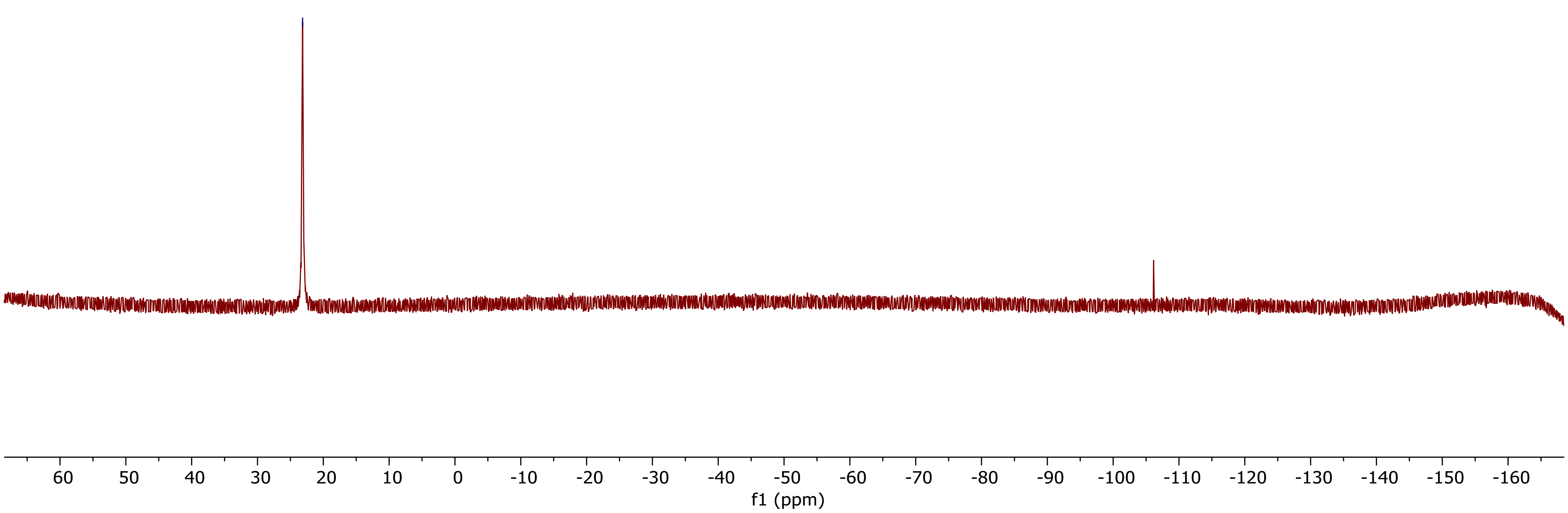

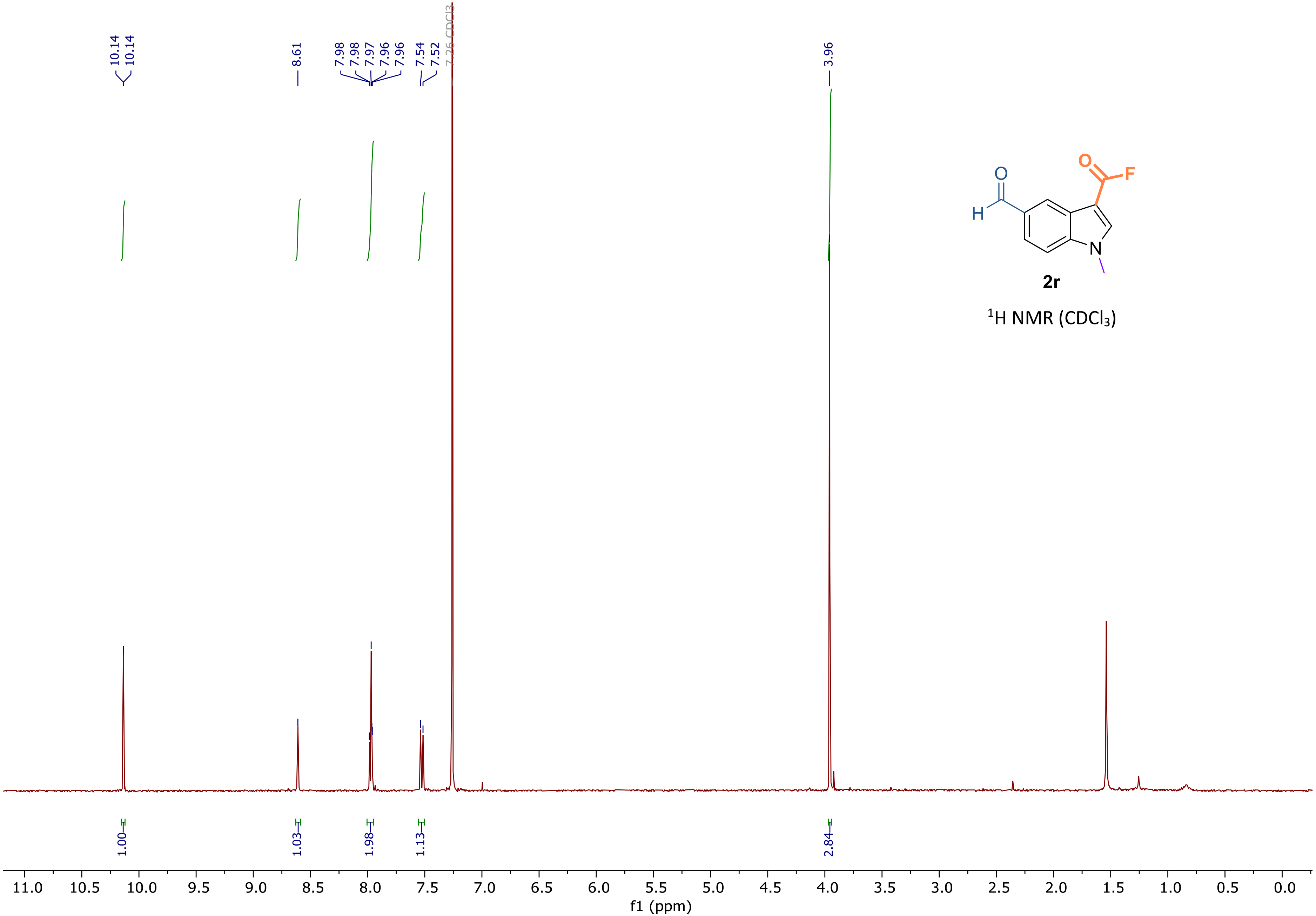

10.14
10.14
8.61
7.98
7.98
7.97
7.96
7.96
7.54
7.52
7.26 CDCl3
3.96
O
O
F
H
N
2r
1H NMR (CDCl3)
1.00
1.03
1.98
1.13
2.84
11.0 10.5 10.0 9.5 9.0 8.5 8.0 7.5 7.0 6.5 6.0 5.5 5.0 4.5 4.0 3.5 3.0 2.5 2.0 1.5 1.0 0.5 0.0
f1 (ppm)

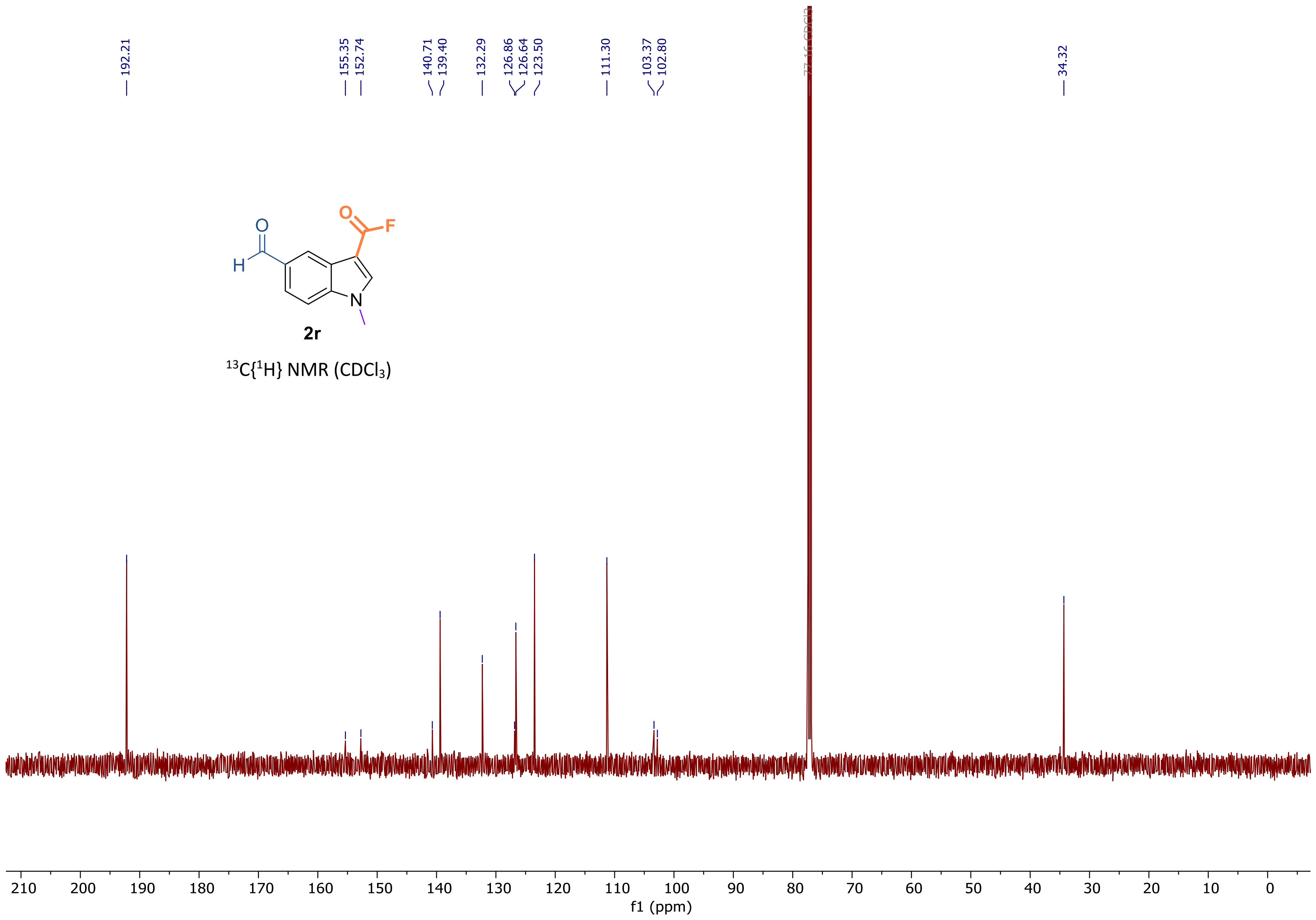

2r

$^{13}C\{^{1}H\}$ NMR ($CDCl_3$)

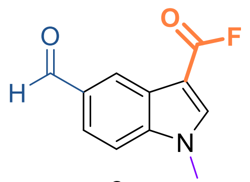

2r

$^{19}F\{^1H\}$ NMR ($CDCl_3$)

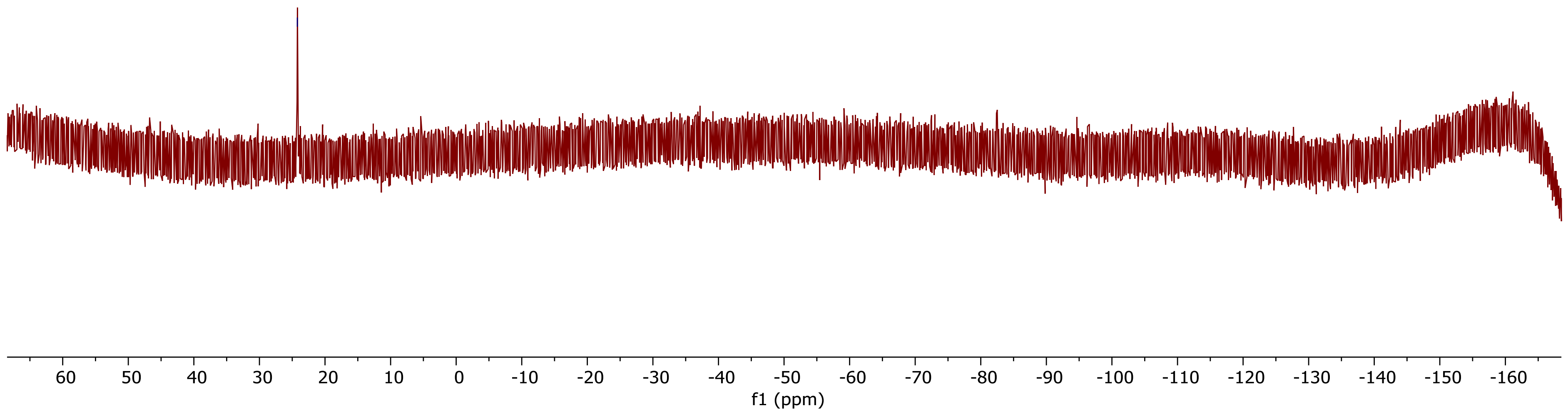

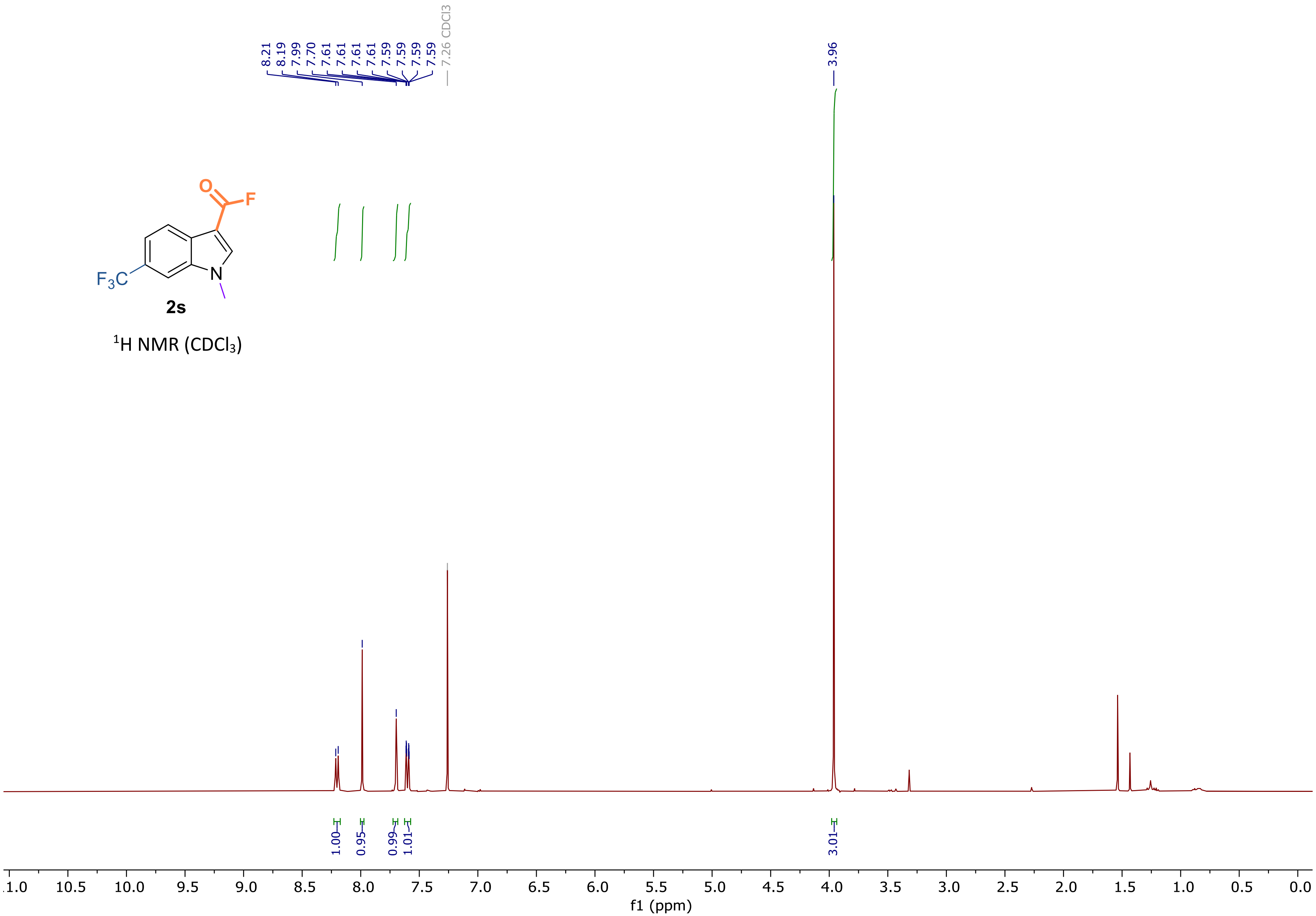
8.21
8.19
7.99
7.70
7.61
7.61
7.61
7.61
7.59
7.59
7.59
7.59
7.26 CDCl3
3.96
O
F
F_3C
N
2s
^1H NMR ($CDCl_3$)
1.00
0.95
0.99
1.01
3.01
1.0
10.5
10.0
9.5
9.0
8.5
8.0
7.5
7.0
6.5
6.0
5.5
5.0
4.5
4.0
3.5
3.0
2.5
2.0
1.5
1.0
0.5
0.0
f1 (ppm)

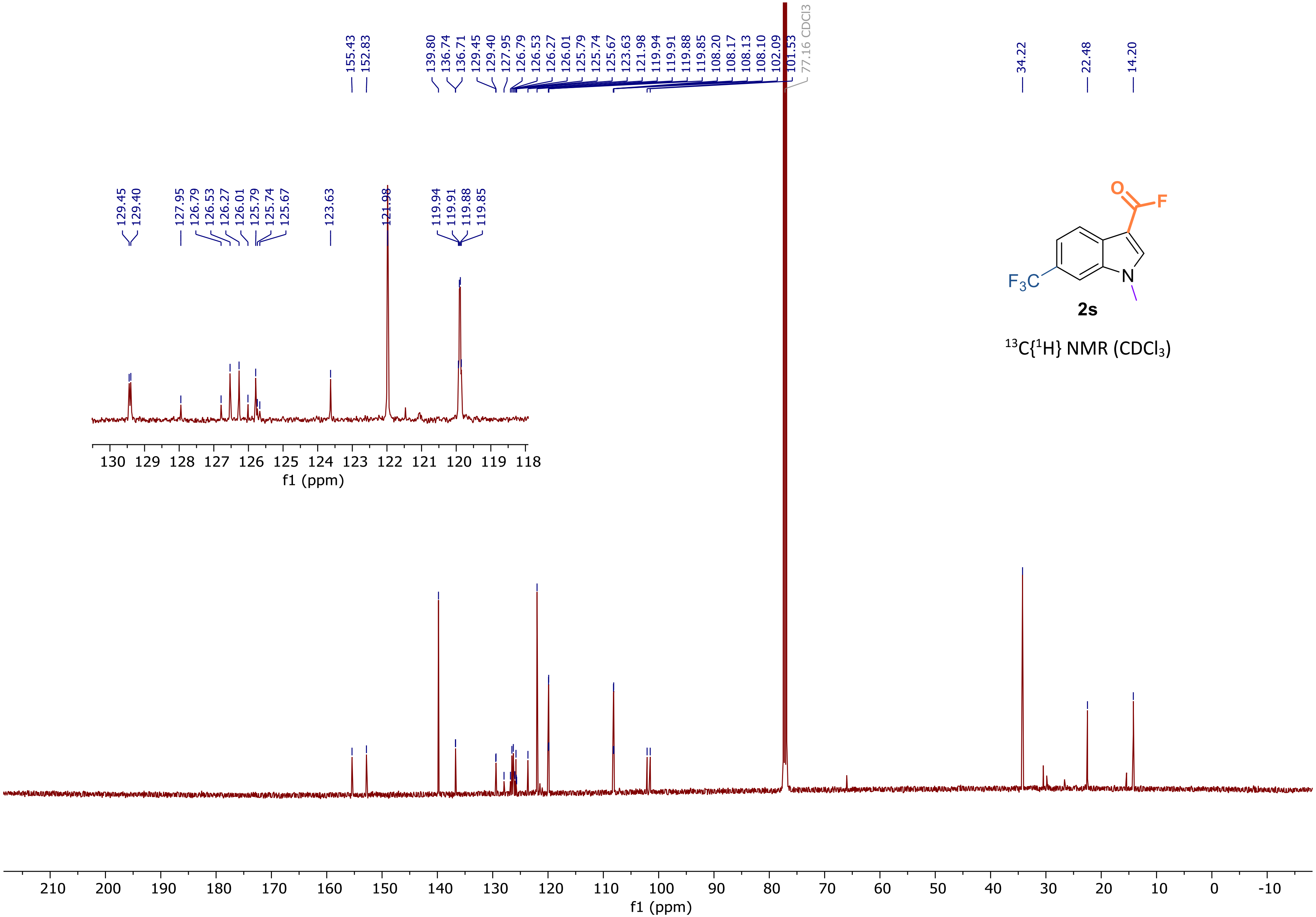
155.43
152.83
139.80
136.74
136.71
129.45
129.40
127.95
126.79
126.53
126.27
126.01
125.79
125.74
125.67
123.63
121.98
119.94
119.91
119.88
119.85
108.20
108.17
108.13
108.10
102.09
101.53
77.16 CDCl3
34.22
22.48
14.20
130 129 128 127 126 125 124 123 122 121 120 119 118
f1 (ppm)
O
F
F_3C
N
2s
$^{13}C\{^1H\}$ NMR ($CDCl_3$)
210 200 190 180 170 160 150 140 130 120 110 100 90 80 70 60 50 40 30 20 10 0 -10
f1 (ppm)

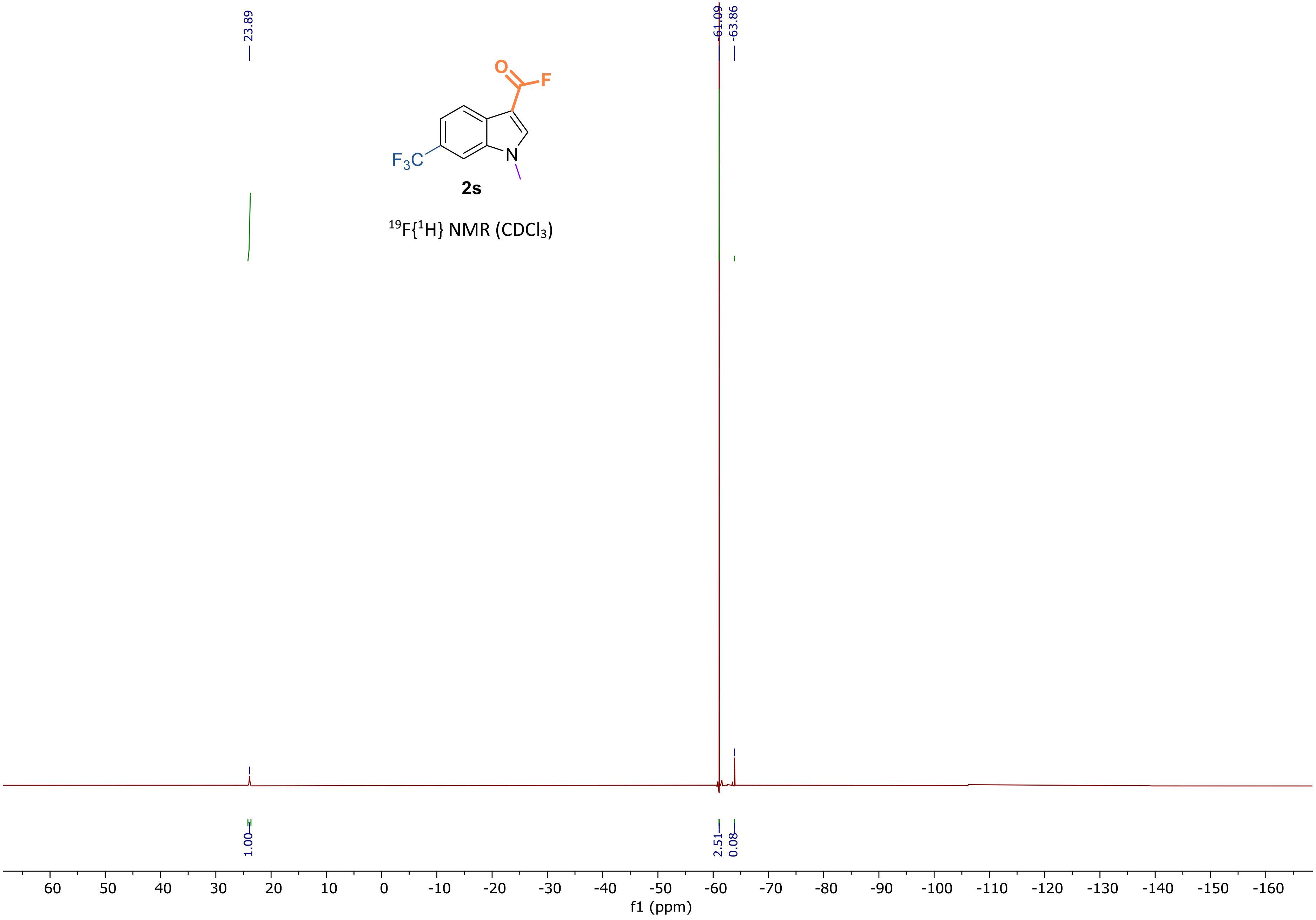

23.89
-61.09
-63.86
F3C
O
F
N
2s
19F{1H} NMR (CDCl3)
1.00
2.51
0.08
60 50 40 30 20 10 0 -10 -20 -30 -40 -50 -60 -70 -80 -90 -100 -110 -120 -130 -140 -150 -160
f1 (ppm)

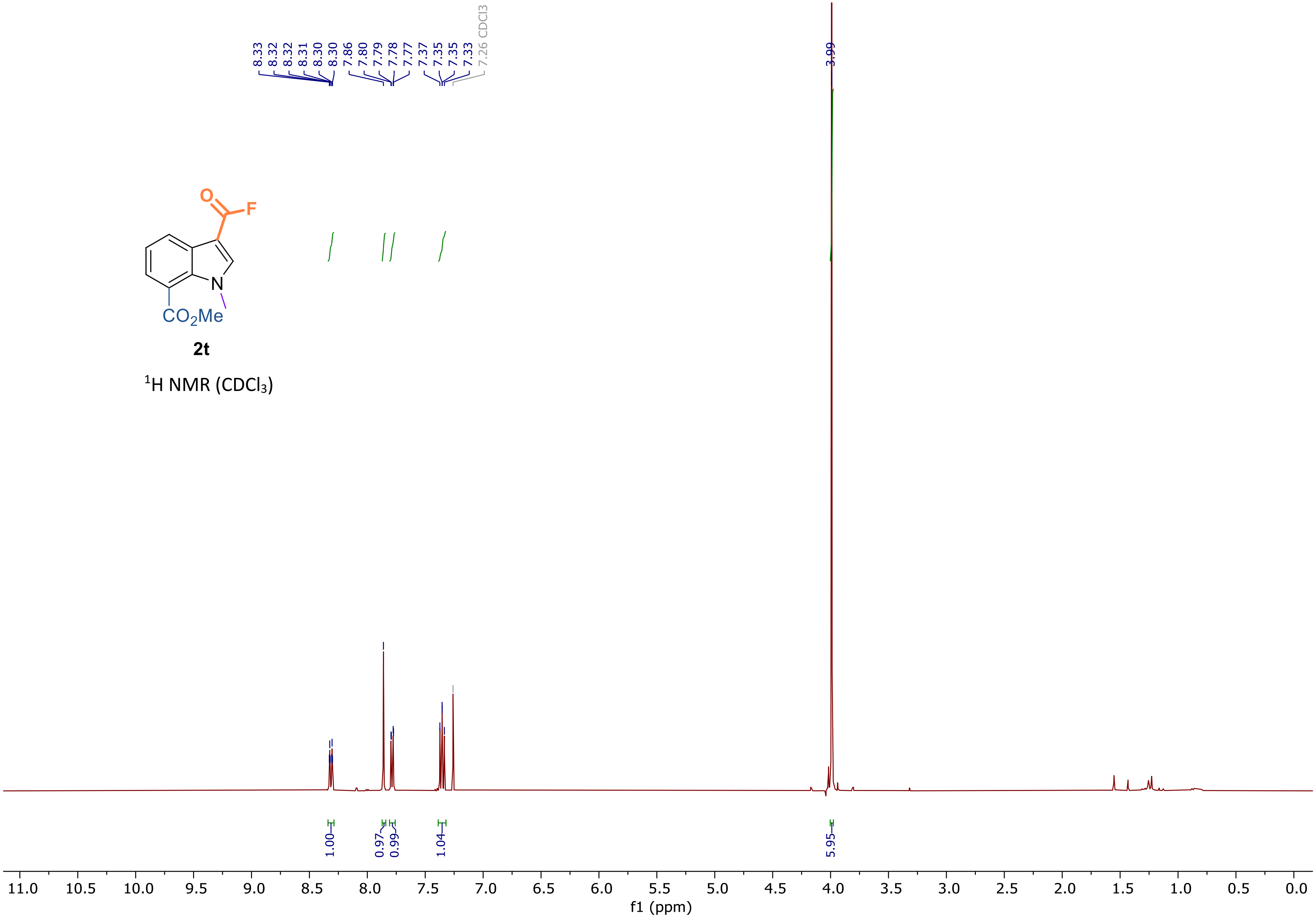

8.33
8.32
8.32
8.31
8.30
8.30
7.86
7.80
7.79
7.78
7.77
7.37
7.35
7.35
7.33
7.26 CDCl3
3.99
O
F
N
CO2Me
2t
1H NMR (CDCl3)
1.00
0.97
0.99
1.04
5.95
11.0
10.5
10.0
9.5
9.0
8.5
8.0
7.5
7.0
6.5
6.0
5.5
5.0
4.5
4.0
3.5
3.0
2.5
2.0
1.5
1.0
0.5
0.0
f1 (ppm)

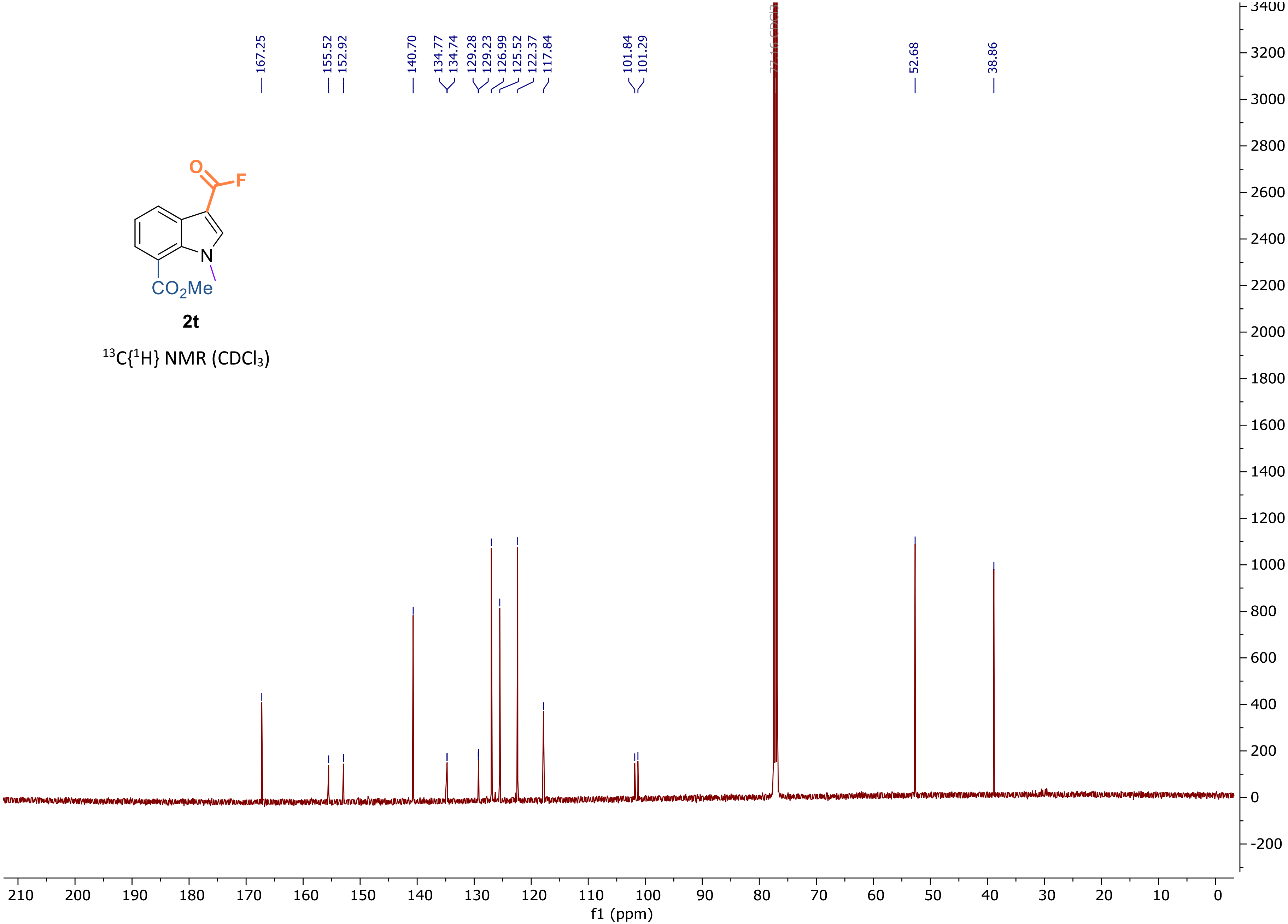

167.25
155.52
152.92
140.70
134.77
134.74
129.28
129.23
126.99
125.52
122.37
117.84
101.84
101.29
77.16 CDCl3
52.68
38.86
O
F
N
CO2Me
2t
13C{1H} NMR (CDCl3)
f1 (ppm)

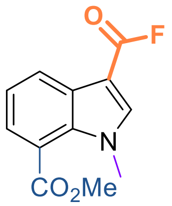

2t

$^{19}F\{^1H\}$ NMR ($CDCl_3$)

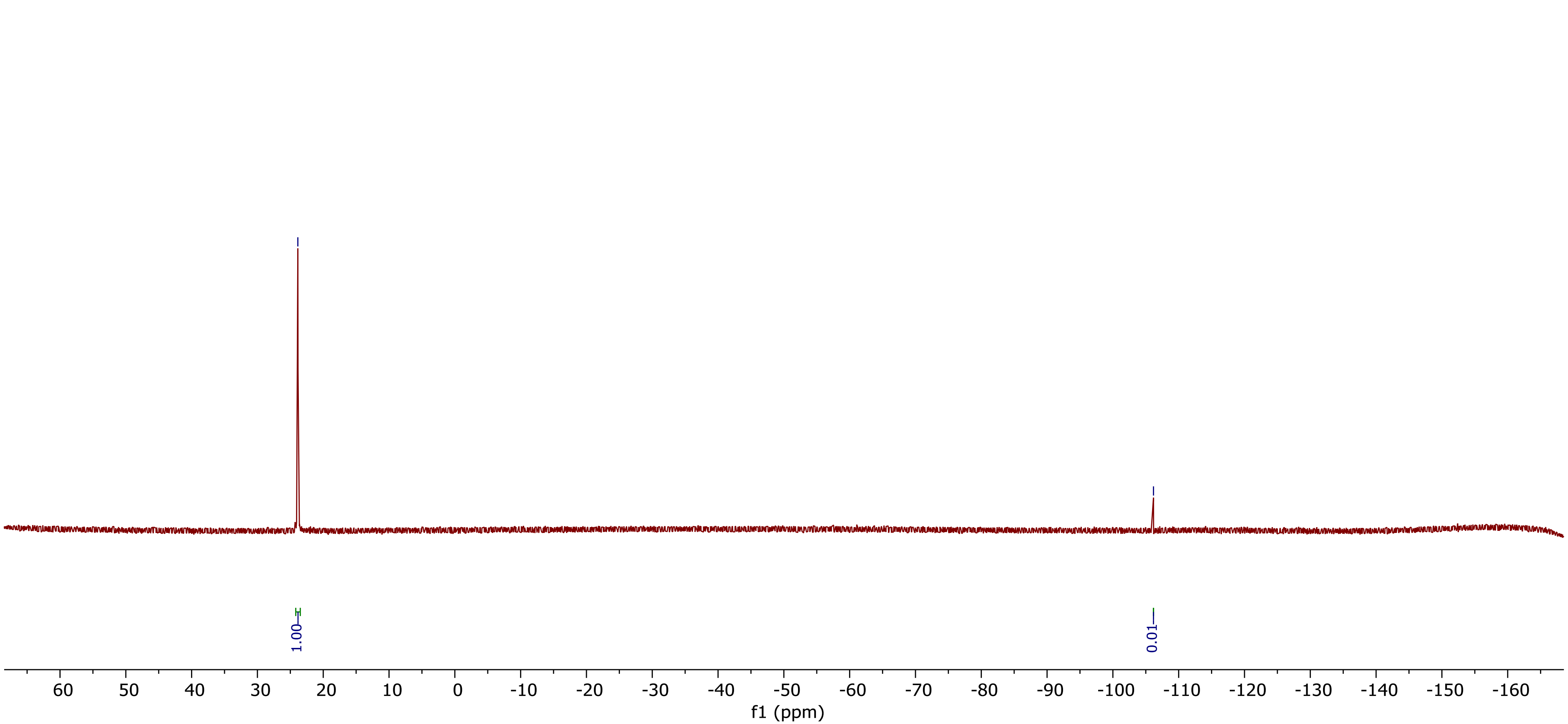

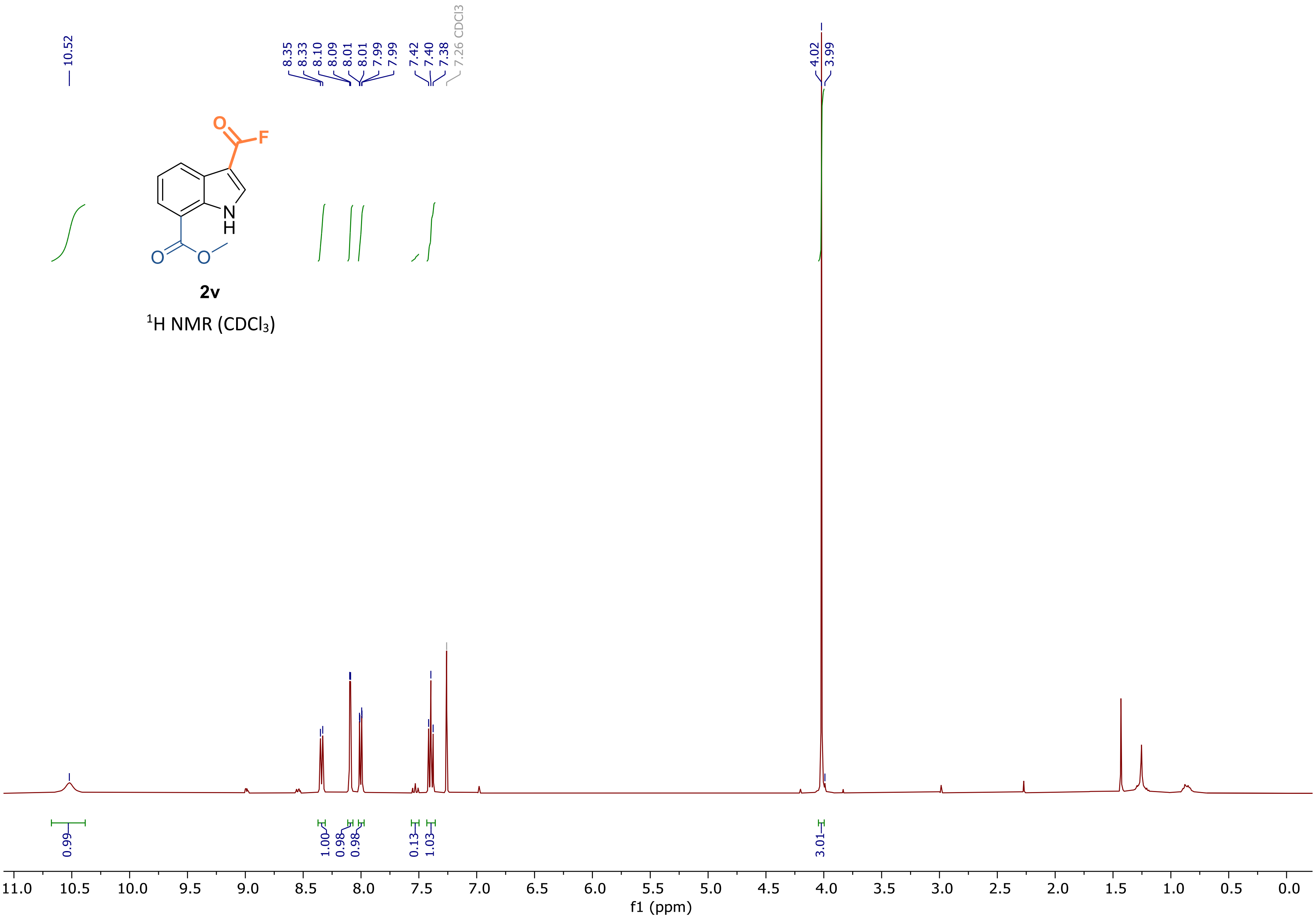

10.52
8.35
8.33
8.10
8.09
8.01
8.01
7.99
7.99
7.42
7.40
7.38
7.26 CDCl3
4.02
3.99
O
F
N
H
O
O
2v
^{1}H NMR ($CDCl_3$)
0.99
1.00
0.98
0.98
0.13
1.03
3.01
11.0 10.5 10.0 9.5 9.0 8.5 8.0 7.5 7.0 6.5 6.0 5.5 5.0 4.5 4.0 3.5 3.0 2.5 2.0 1.5 1.0 0.5 0.0
f1 (ppm)

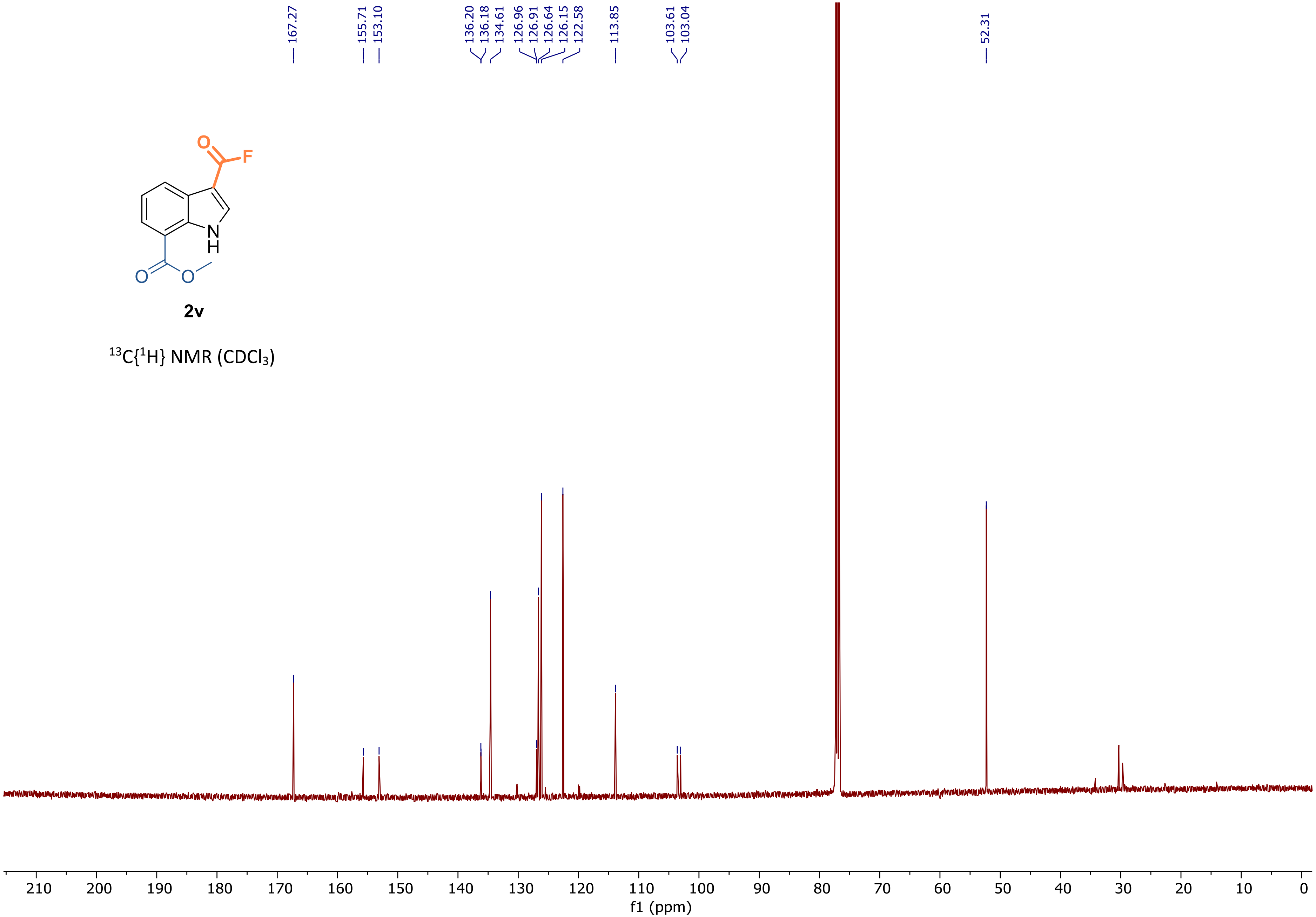

2v

$^{13}C\{^{1}H\}$ NMR ($CDCl_3$)

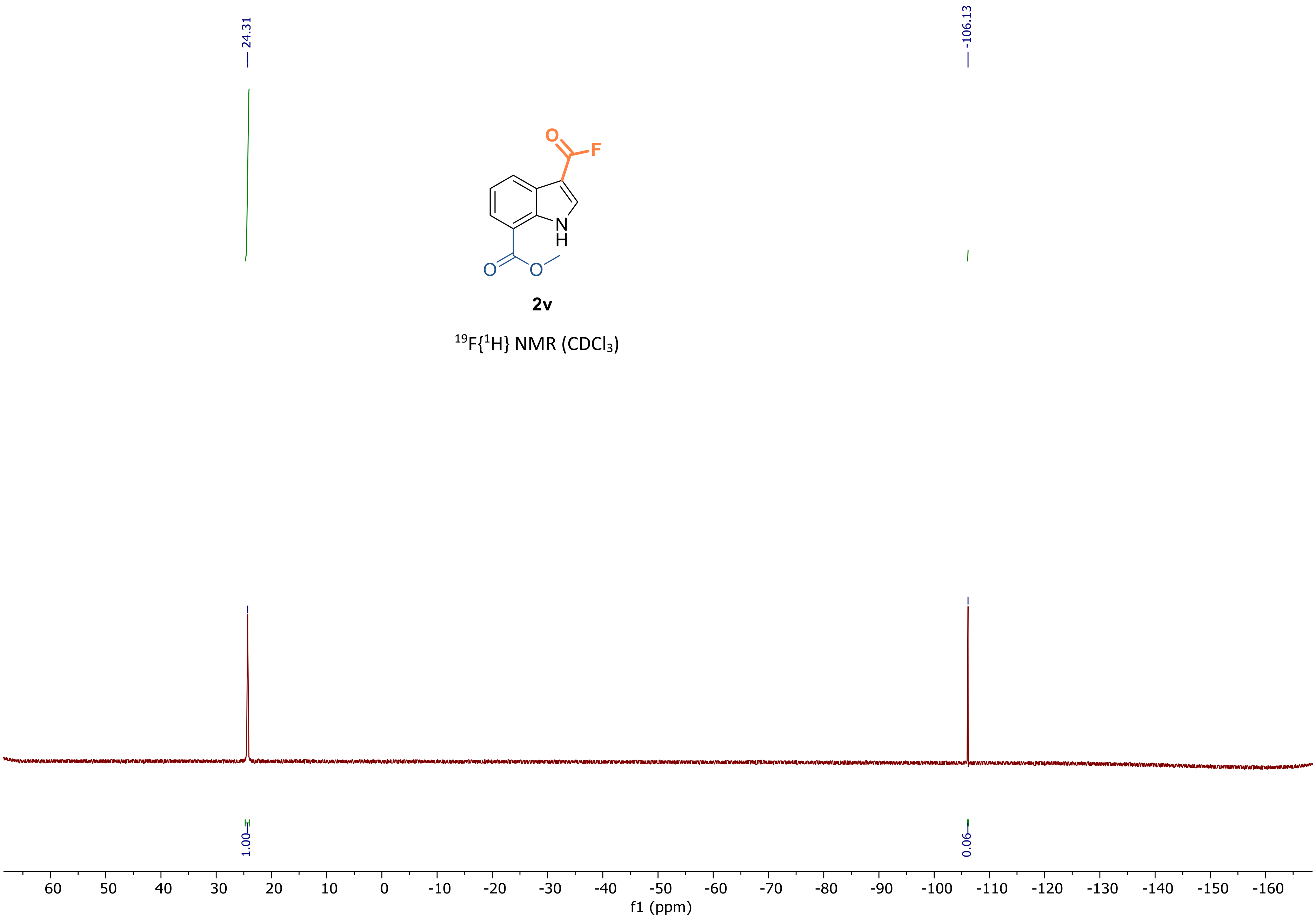
24.31
-106.13
2v
$^{19}F\{^1H\}$ NMR ($CDCl_3$)
1.00
0.06
60 50 40 30 20 10 0 -10 -20 -30 -40 -50 -60 -70 -80 -90 -100 -110 -120 -130 -140 -150 -160
f1 (ppm)

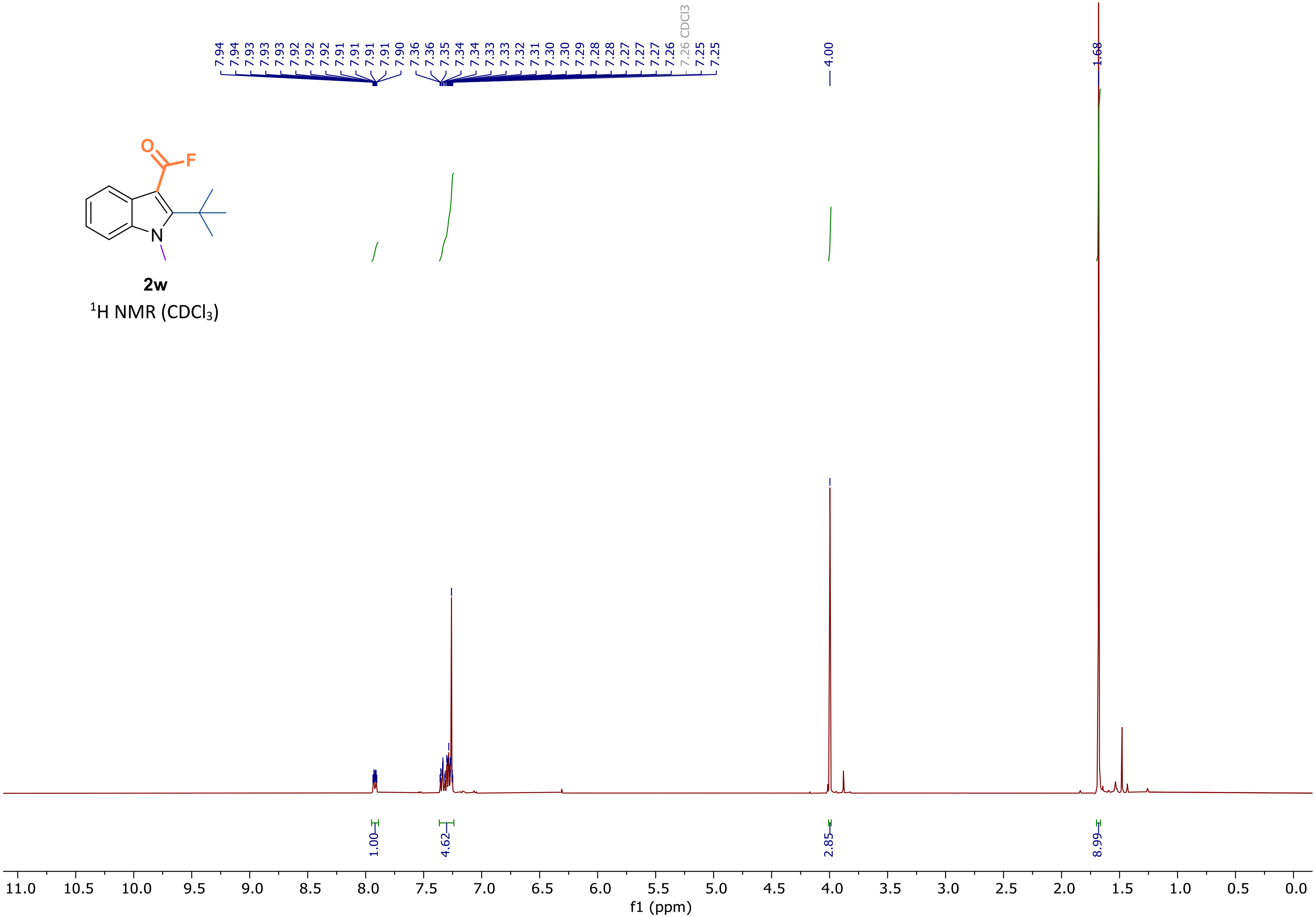

2w

^{1}H NMR ($CDCl_3$)

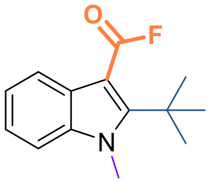

2w

$^{13}C\{^{1}H\}$ NMR ($CDCl_3$)

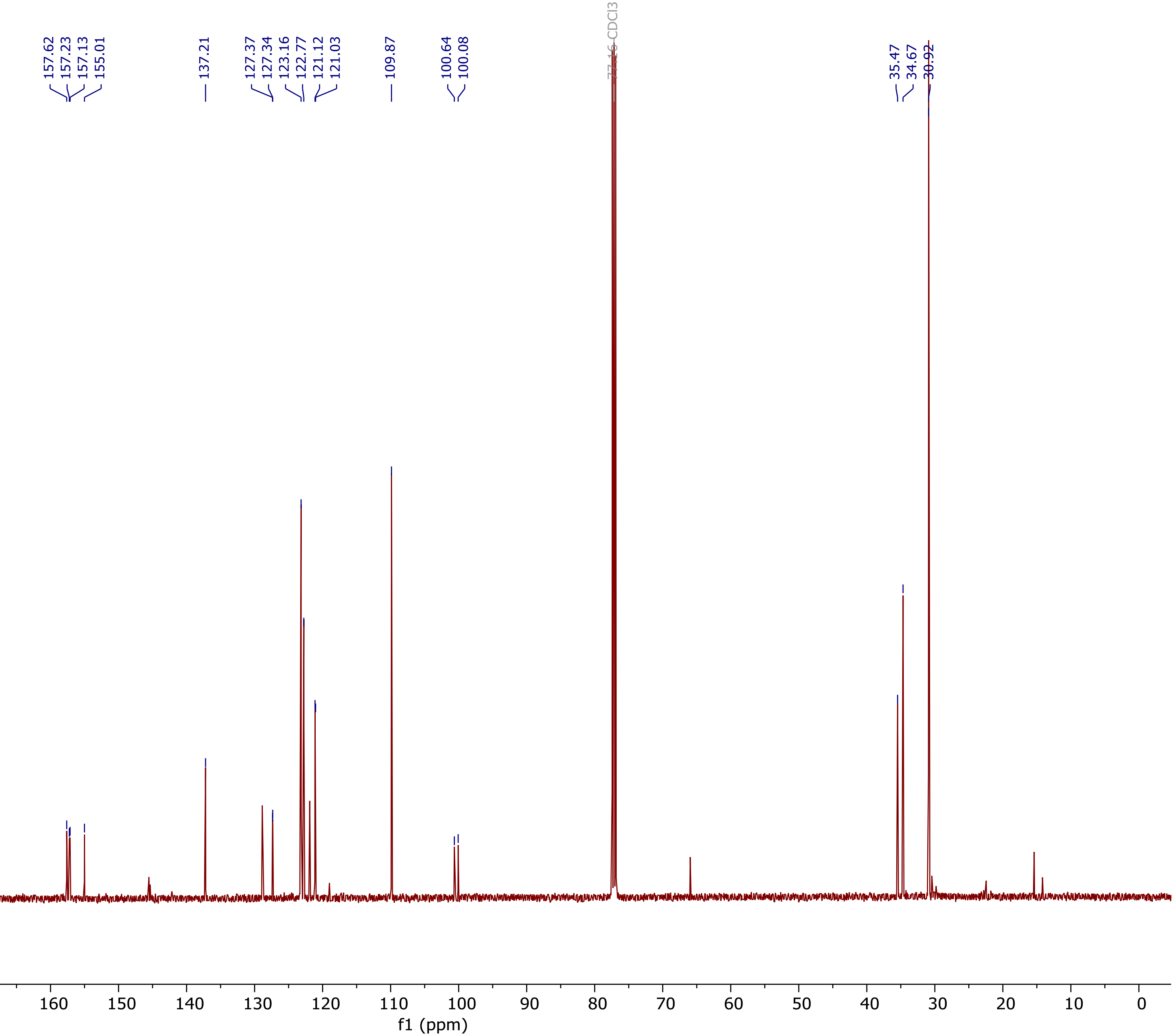

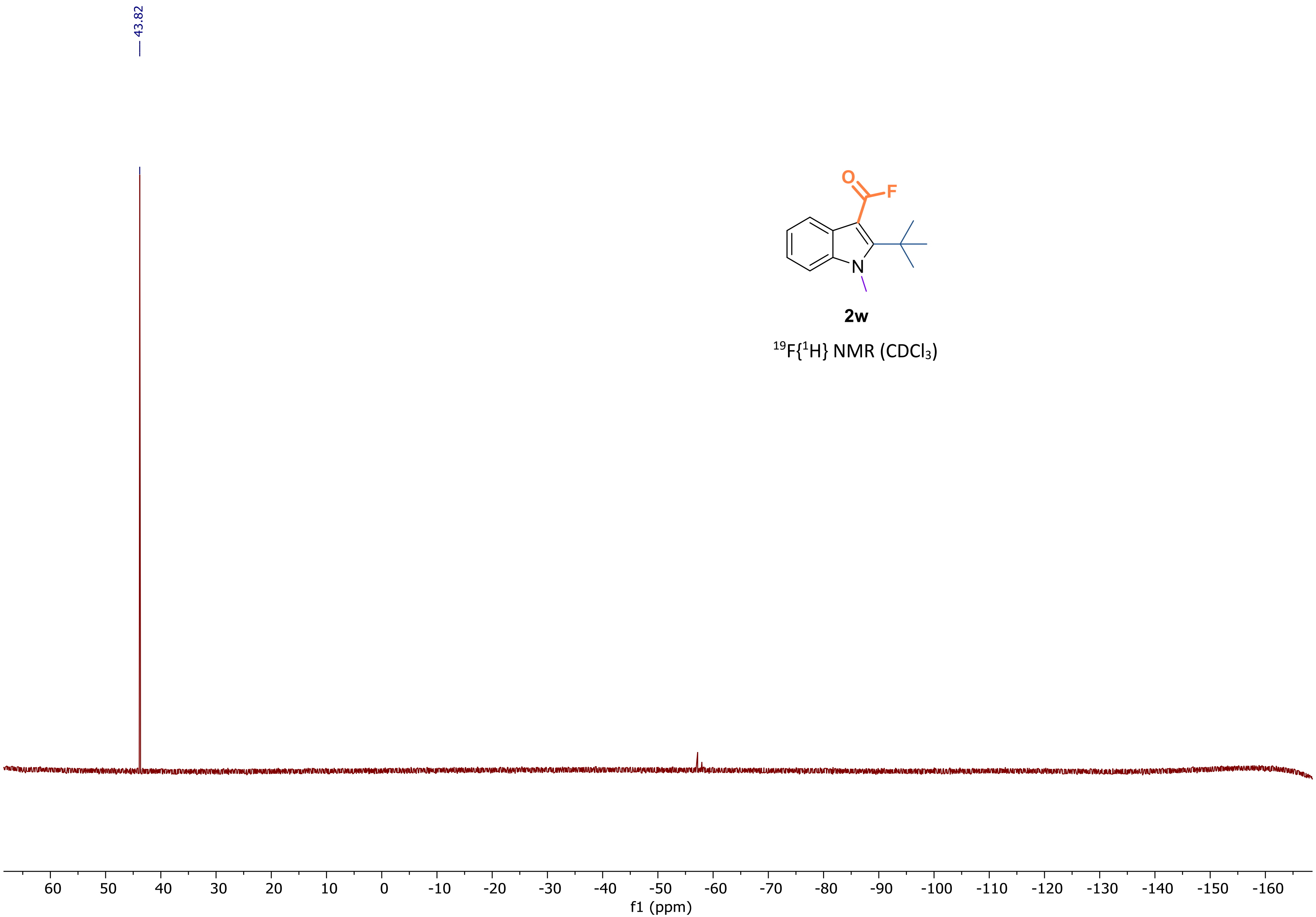
43.82
O
F
N
2w
$^{19}F\{^{1}H\}$ NMR ($CDCl_3$)
60 50 40 30 20 10 0 -10 -20 -30 -40 -50 -60 -70 -80 -90 -100 -110 -120 -130 -140 -150 -160
f1 (ppm)

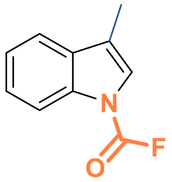

2x

^1H NMR ($CDCl_3$)

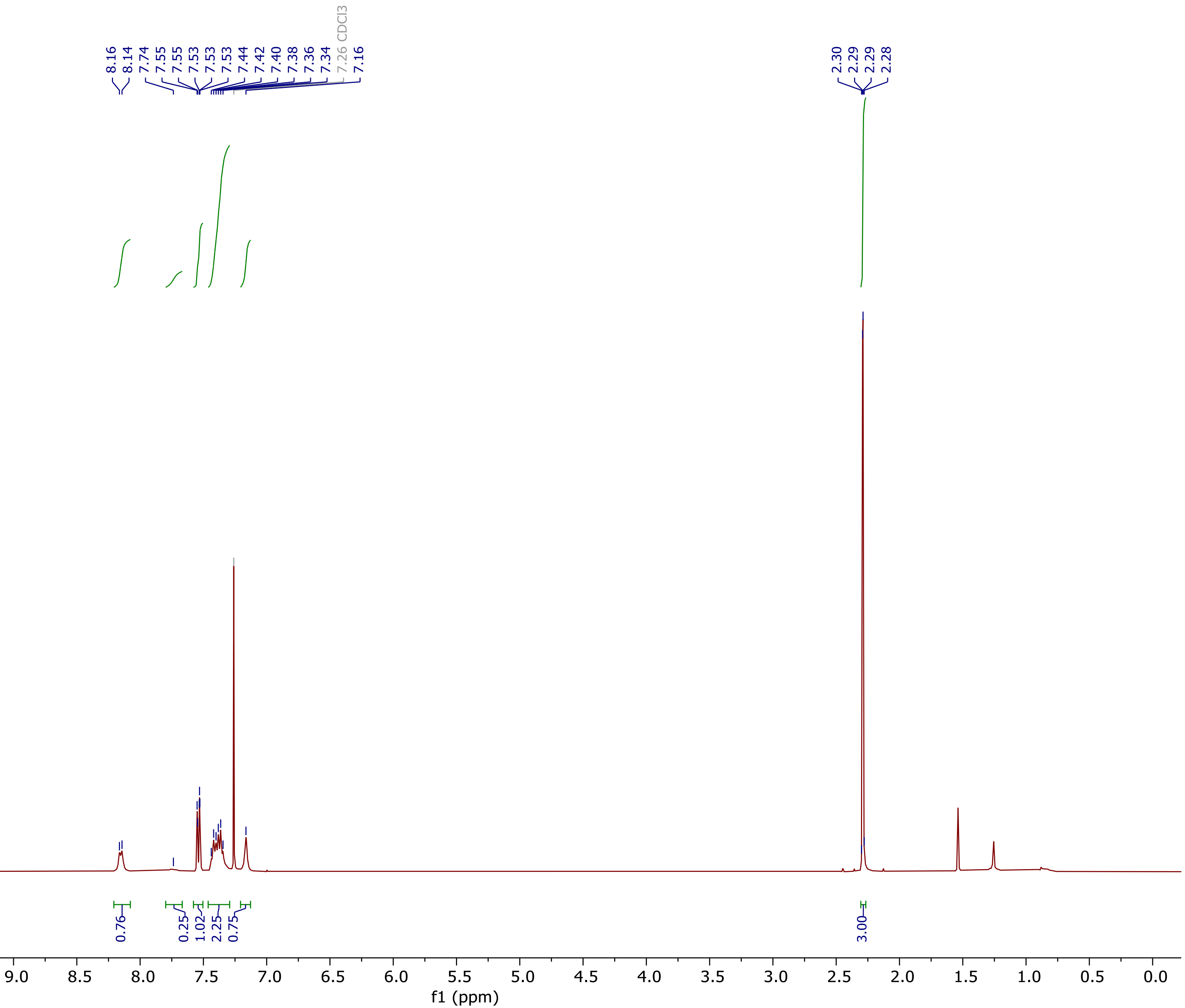

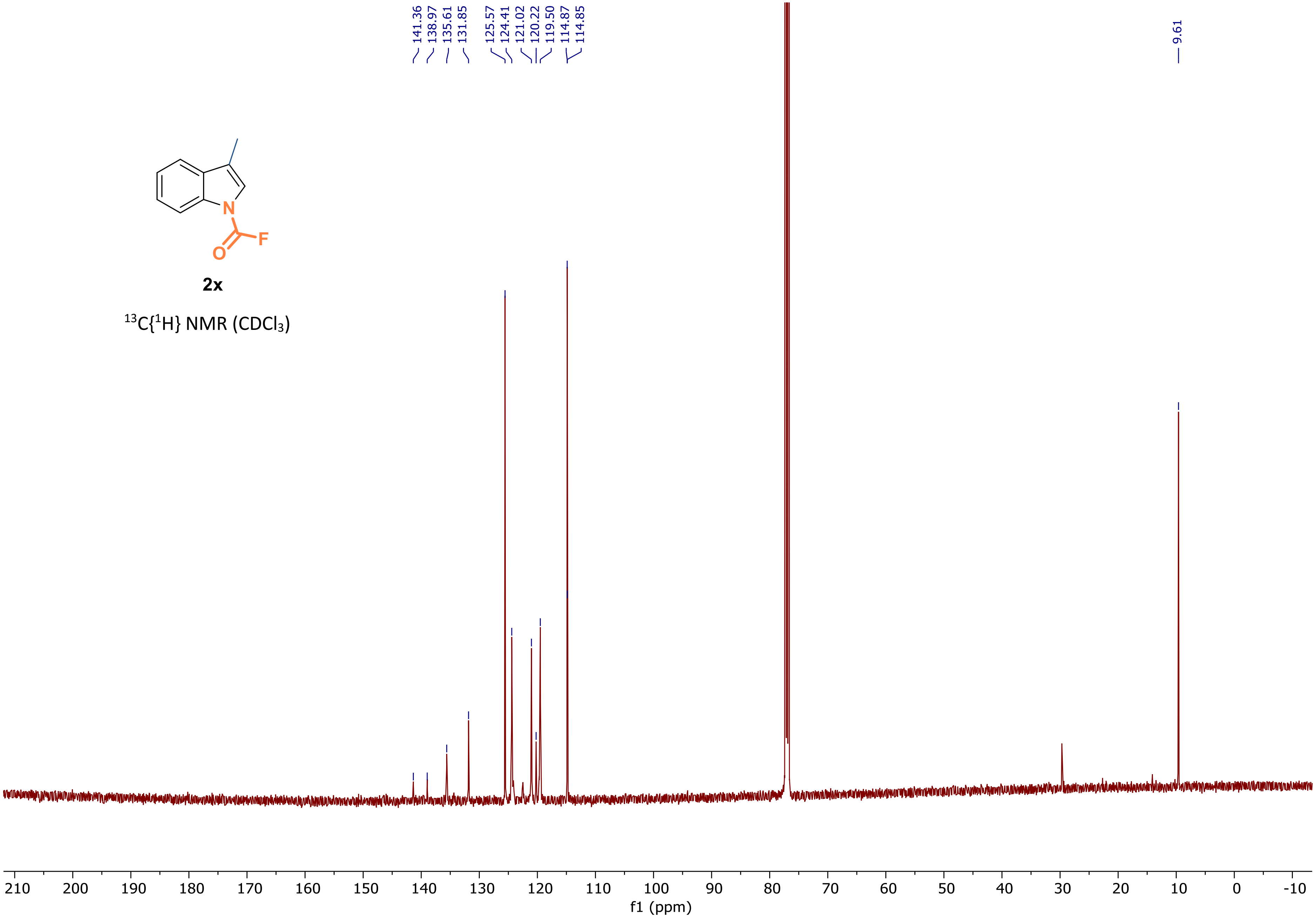

2x

$^{13}C\{^1H\}$ NMR ($CDCl_3$)

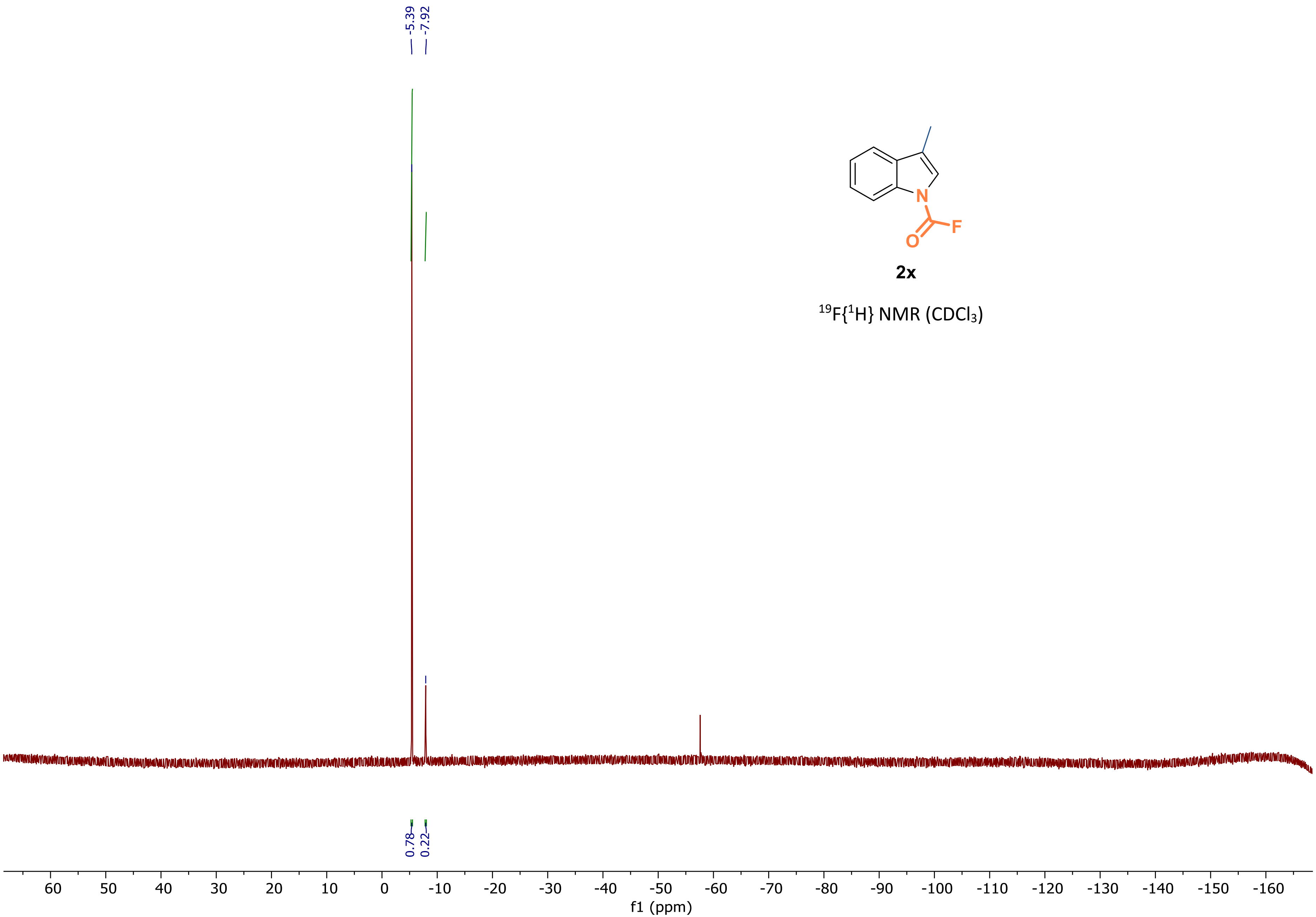
-5.39
-7.92
2x
19F{1H} NMR (CDCl3)
0.78
0.22
60 50 40 30 20 10 0 -10 -20 -30 -40 -50 -60 -70 -80 -90 -100 -110 -120 -130 -140 -150 -160
f1 (ppm)

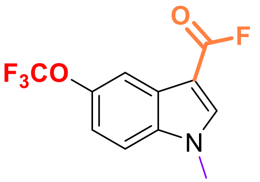

2z[5]

^{1}H NMR ($CDCl_3$)

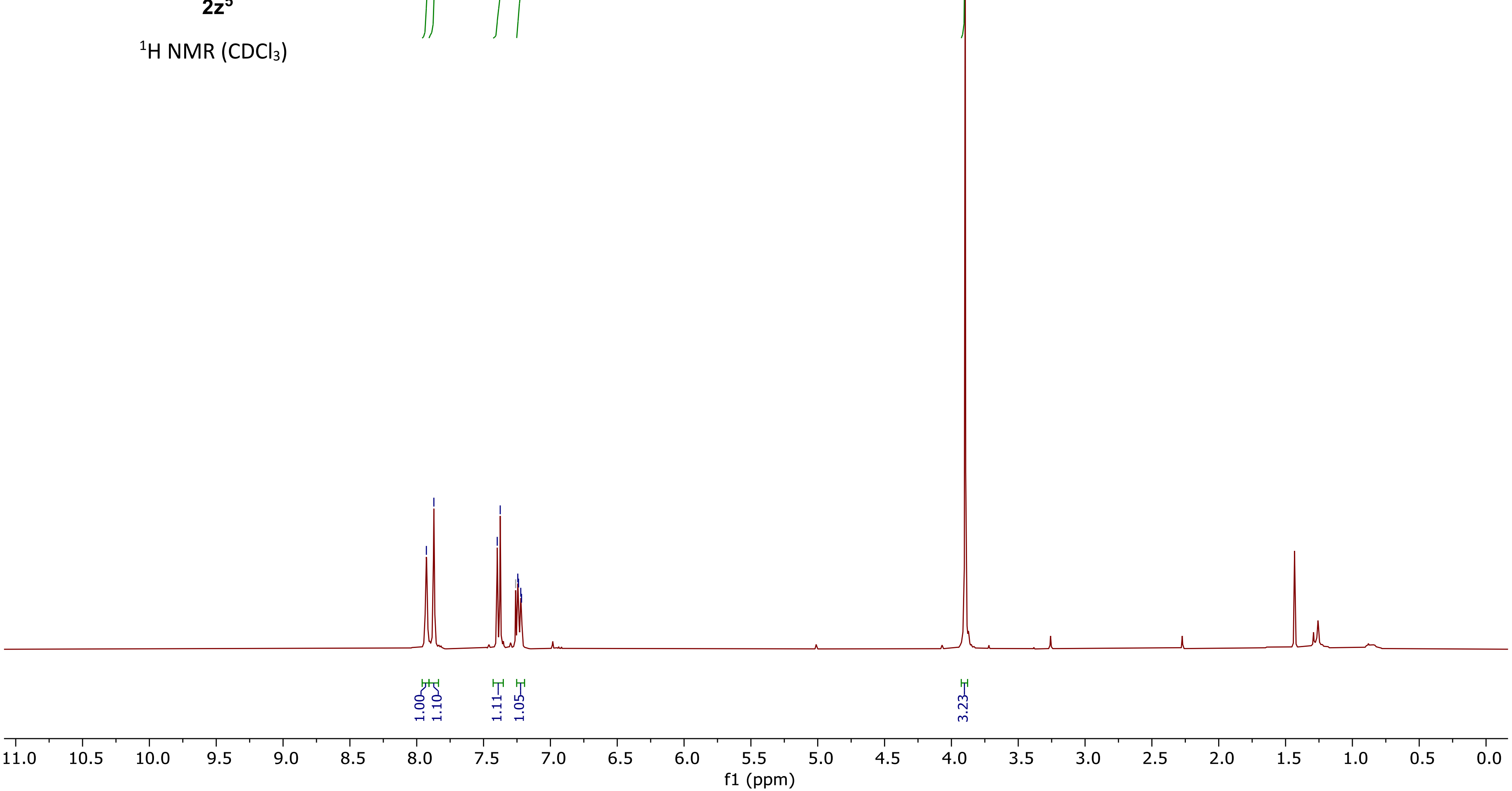

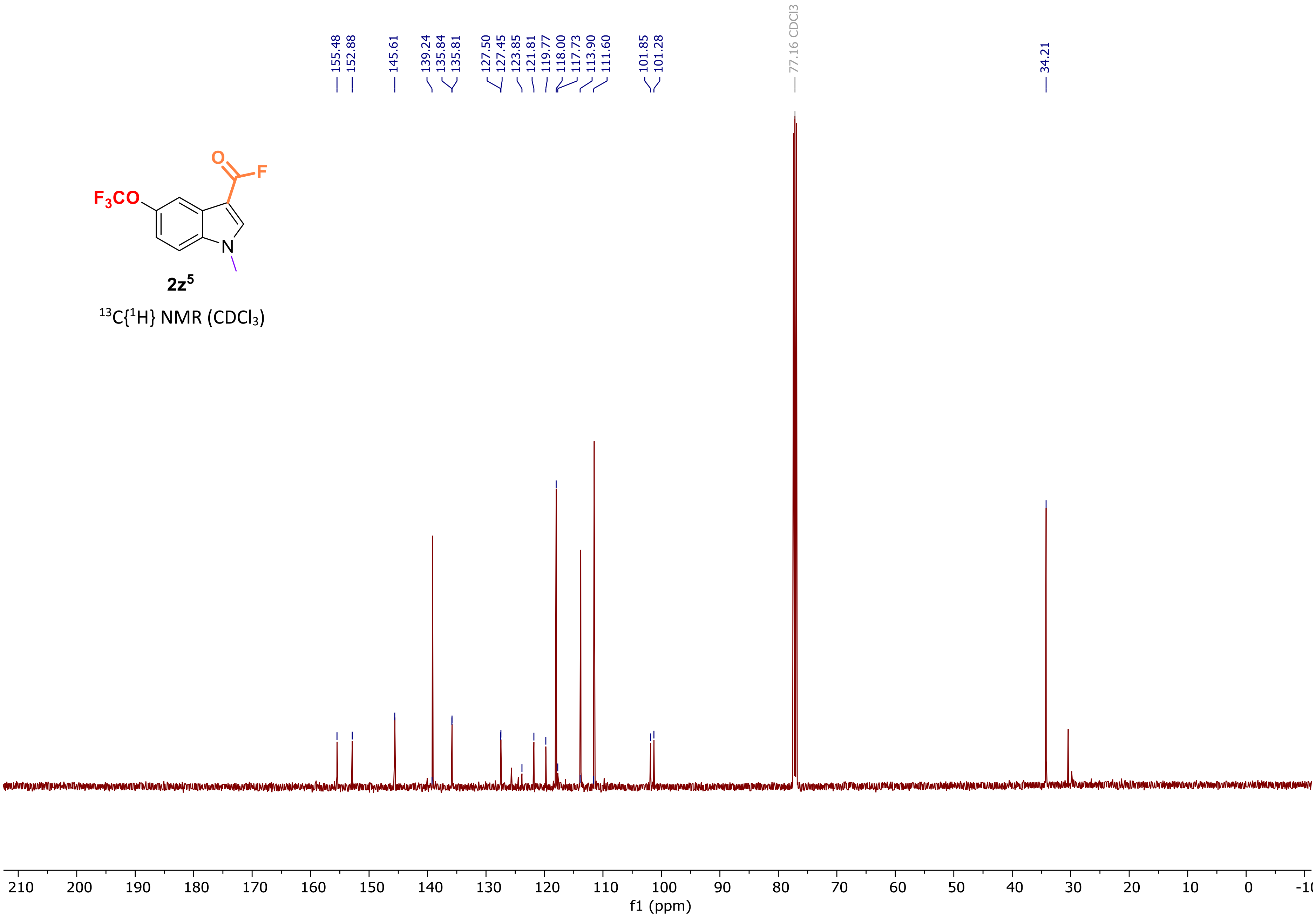

2z5

$^{13}C\{^{1}H\}$ NMR ($CDCl_3$)

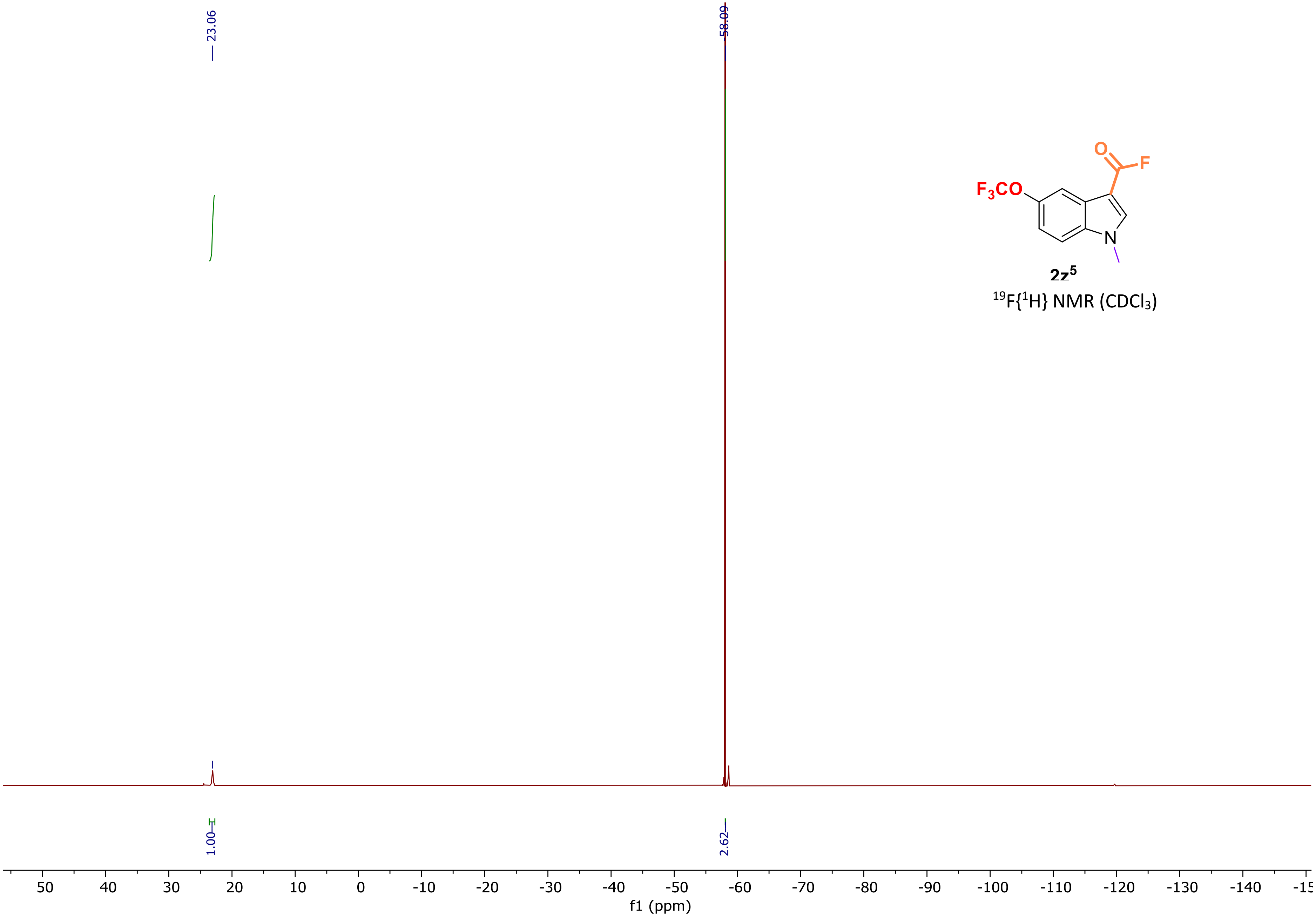

2z[5]

$^{19}F\{^1H\}$ NMR ($CDCl_3$)

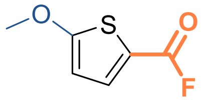

2aa

^1H NMR ($CDCl_3$)

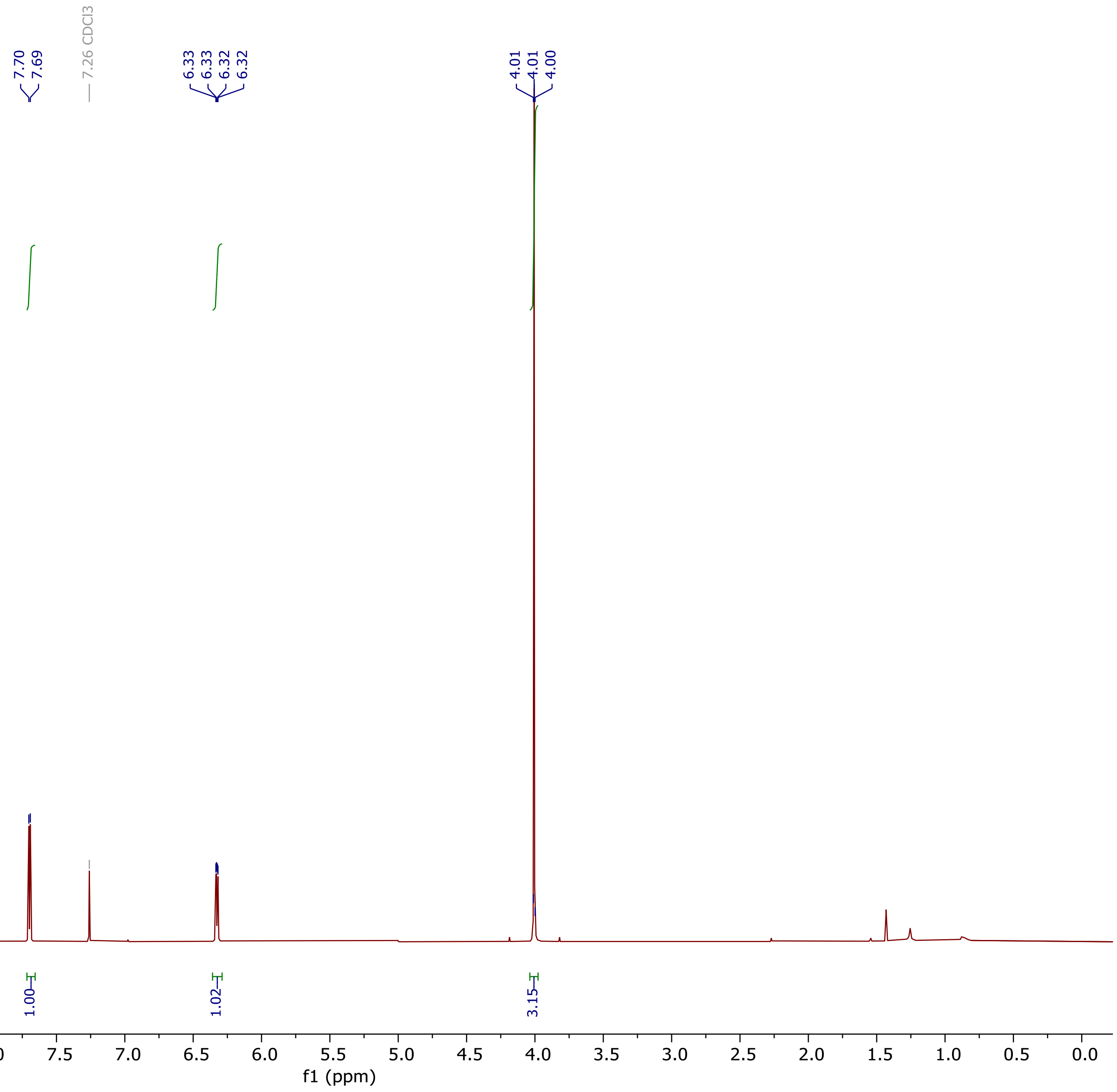

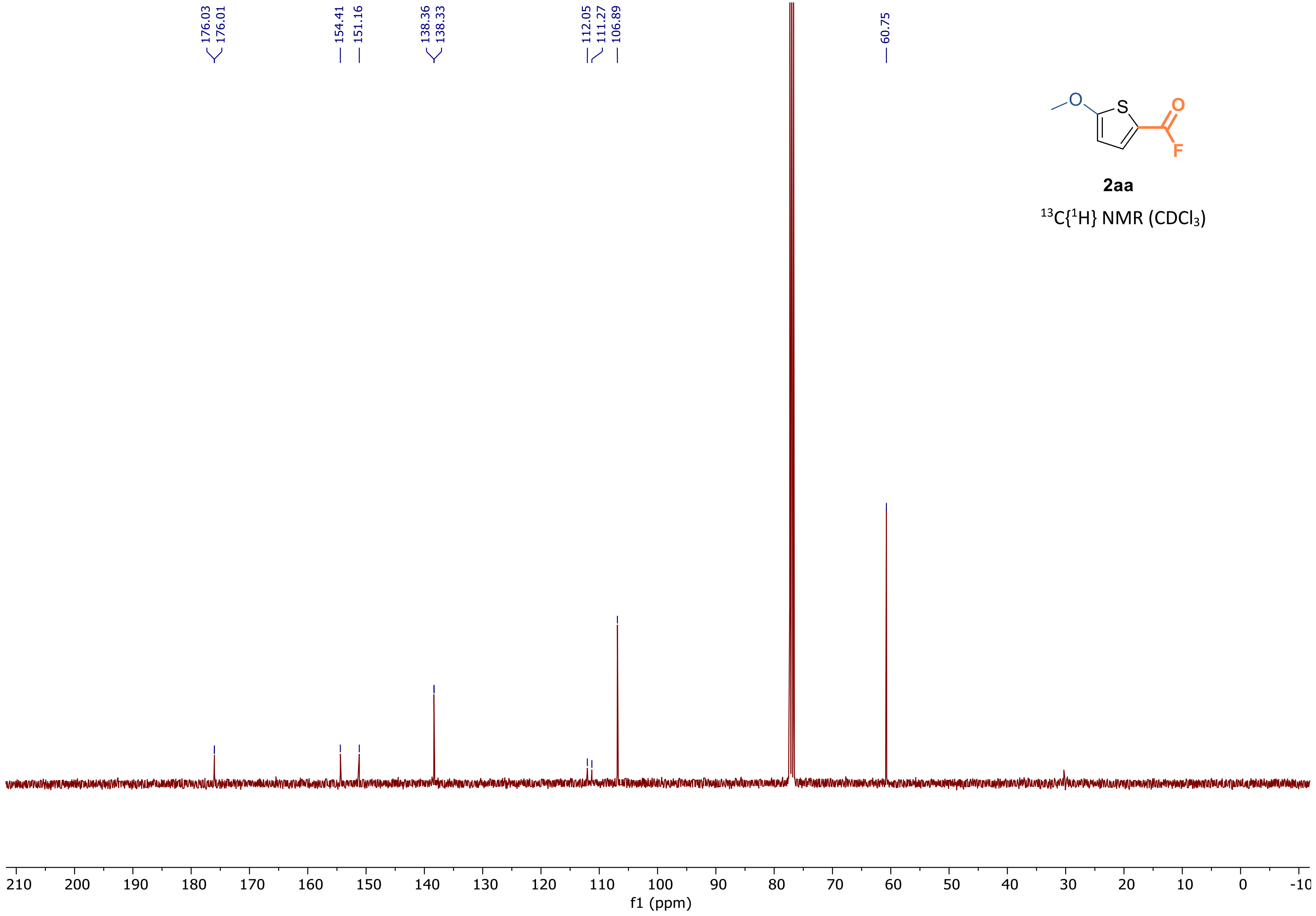

176.03
176.01
154.41
151.16
138.36
138.33
112.05
111.27
106.89
60.75
O
S
O
F
2aa
13C{1H} NMR (CDCl3)
210 200 190 180 170 160 150 140 130 120 110 100 90 80 70 60 50 40 30 20 10 0 -10
f1 (ppm)

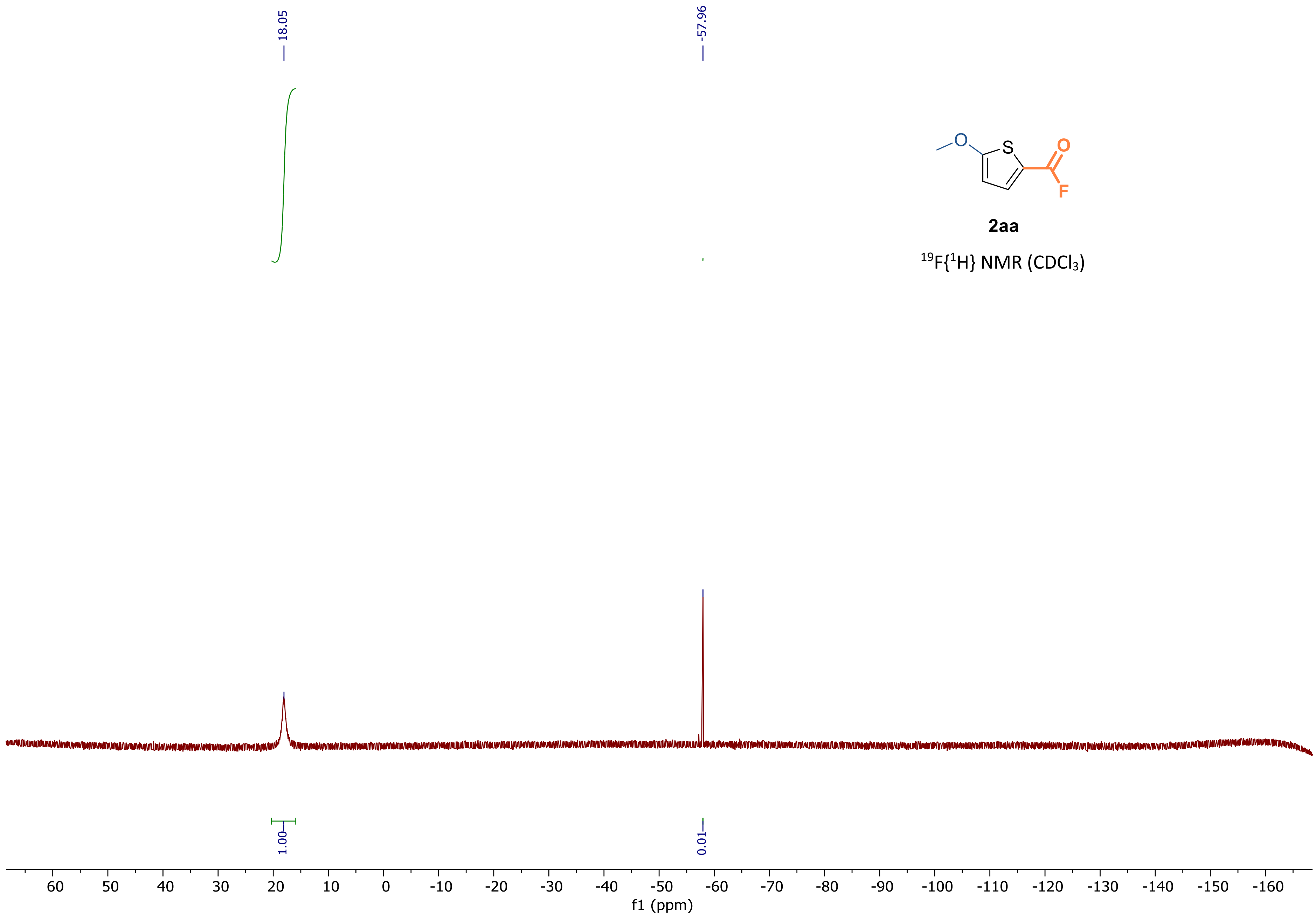

18.05
-57.96
S
O
O
F
2aa
19F{1H} NMR (CDCl3)
1.00
0.01
60 50 40 30 20 10 0 -10 -20 -30 -40 -50 -60 -70 -80 -90 -100 -110 -120 -130 -140 -150 -160
f1 (ppm)

2ab

^1H NMR ($CDCl_3$)

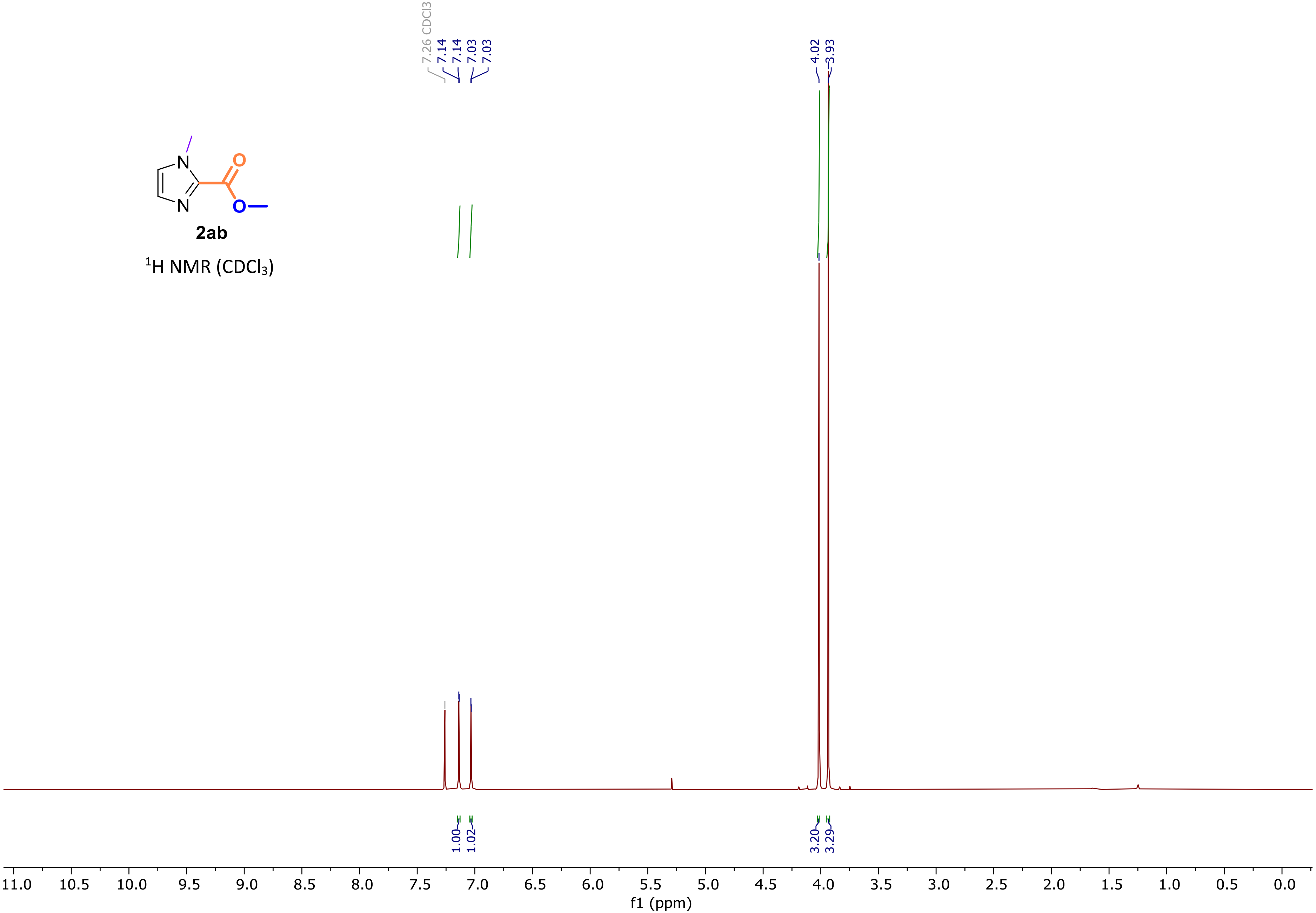

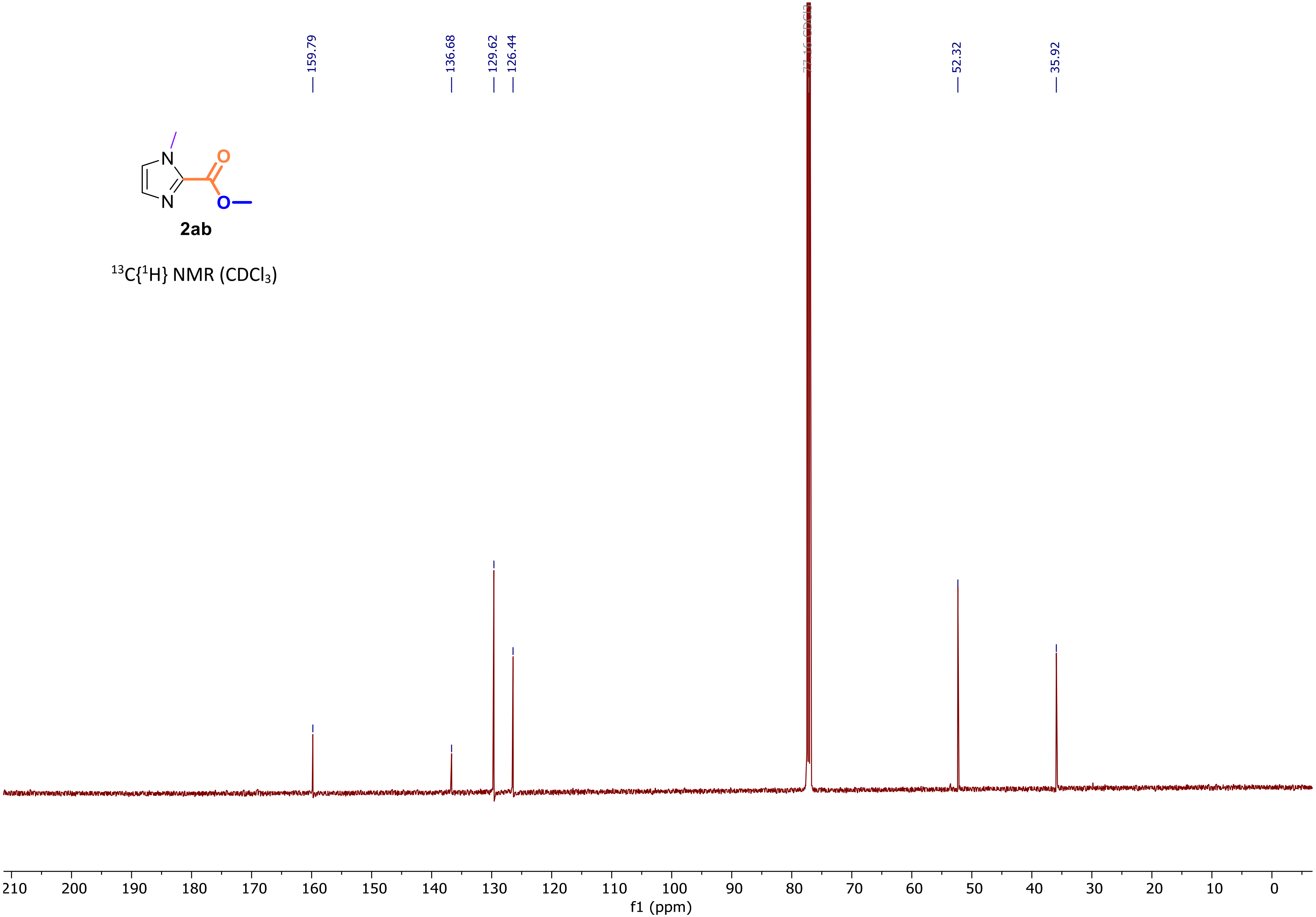

159.79
136.68
129.62
126.44
77.16 CDCl3
52.32
35.92
N
N
O
O
2ab
$^{13}C\{^{1}H\}$ NMR ($CDCl_3$)
210
200
190
180
170
160
150
140
130
120
110
100
90
80
70
60
50
40
30
20
10
0
f1 (ppm)

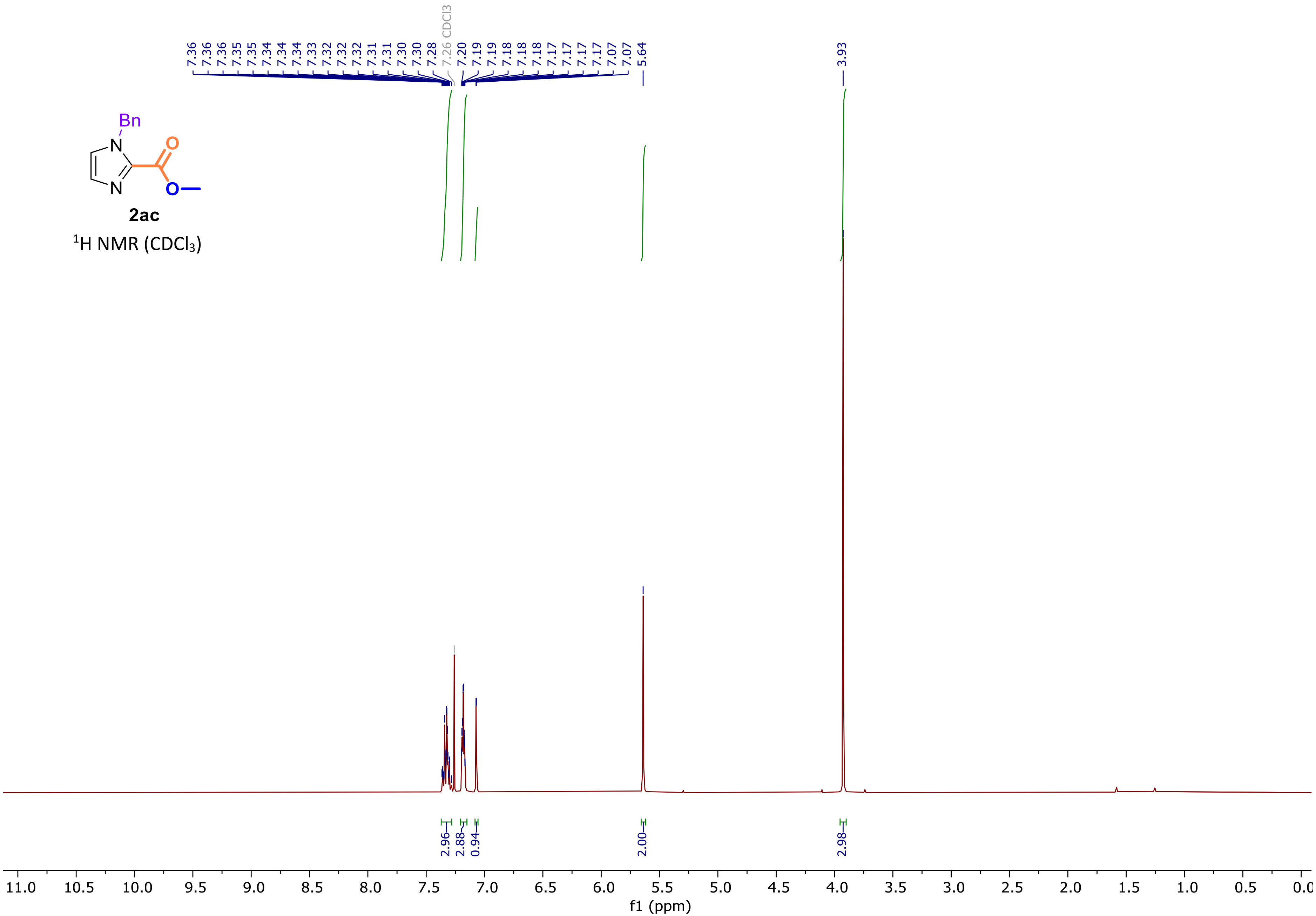

2ac

^{1}H NMR ($CDCl_3$)

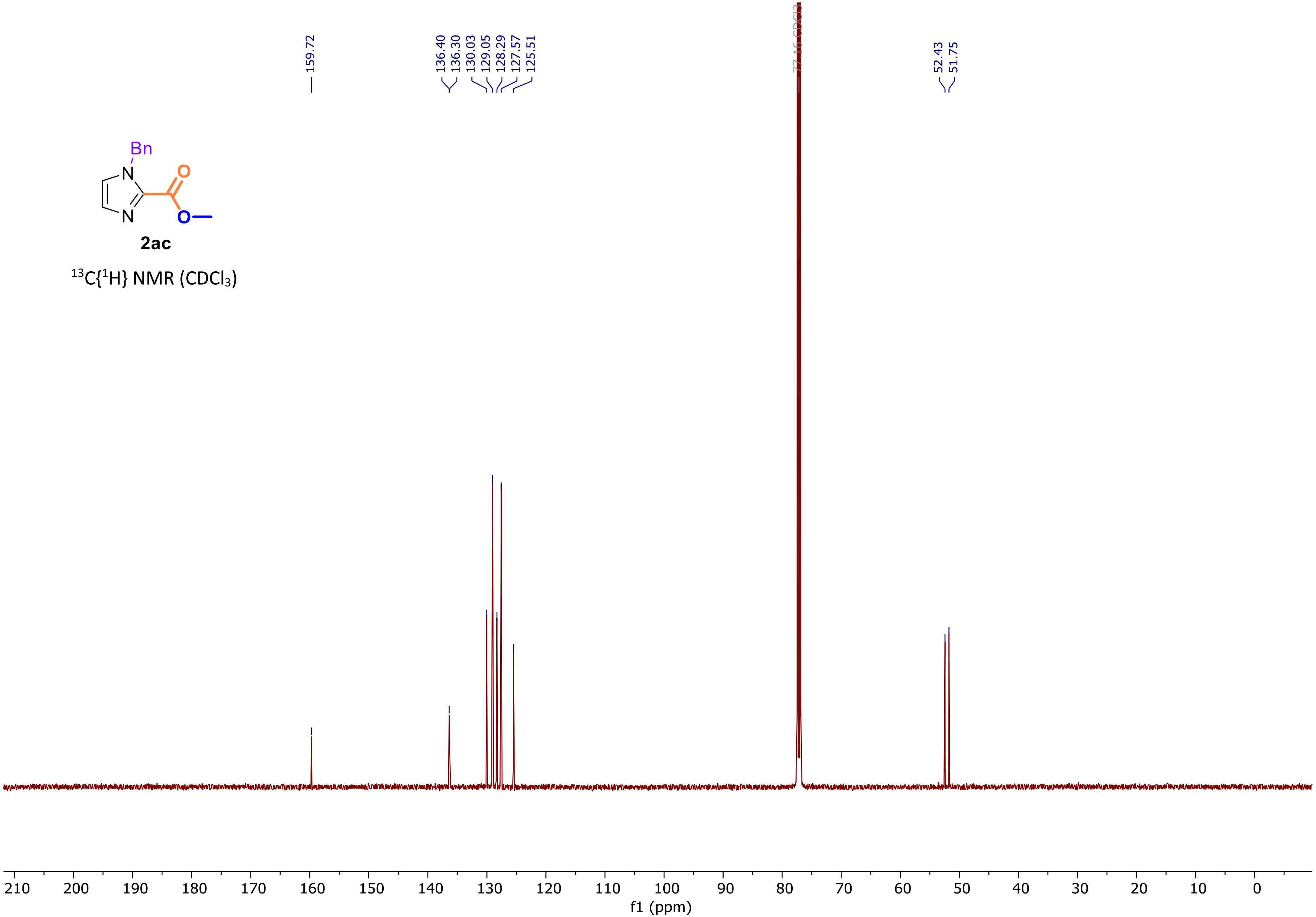
159.72
136.40
136.30
130.03
129.05
128.29
127.57
125.51
77.16 CDCl3
52.43
51.75
Bn
N
O
N
O
2ac
$^{13}C\{^{1}H\}$ NMR ($CDCl_3$)
210
200
190
180
170
160
150
140
130
120
110
100
90
80
70
60
50
40
30
20
10
0
f1 (ppm)

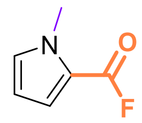

2ad

^{1}H NMR ($CDCl_3$)

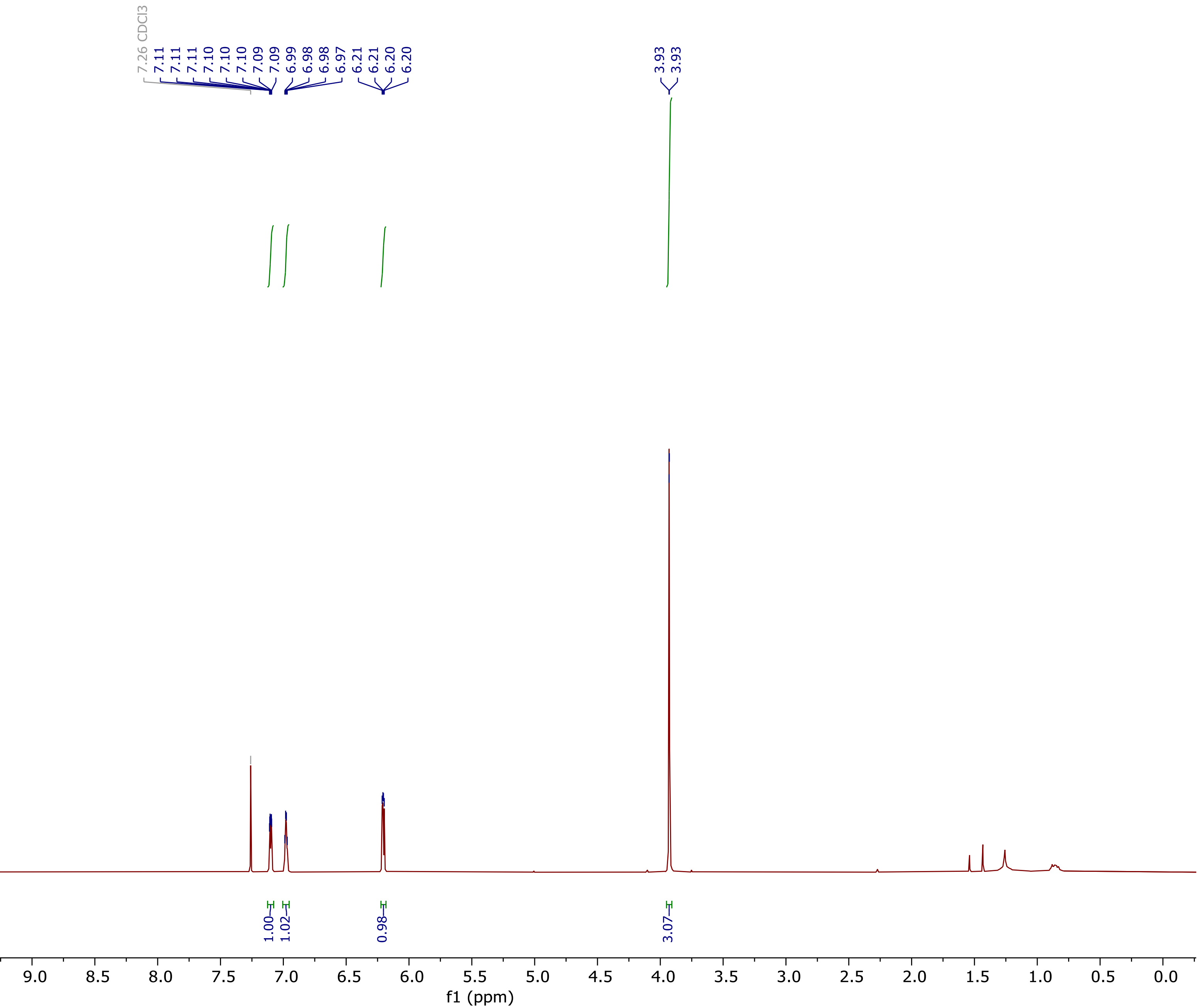

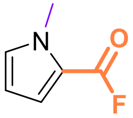

2ad

$^{13}C\{^1H\}$ NMR ($CDCl_3$)

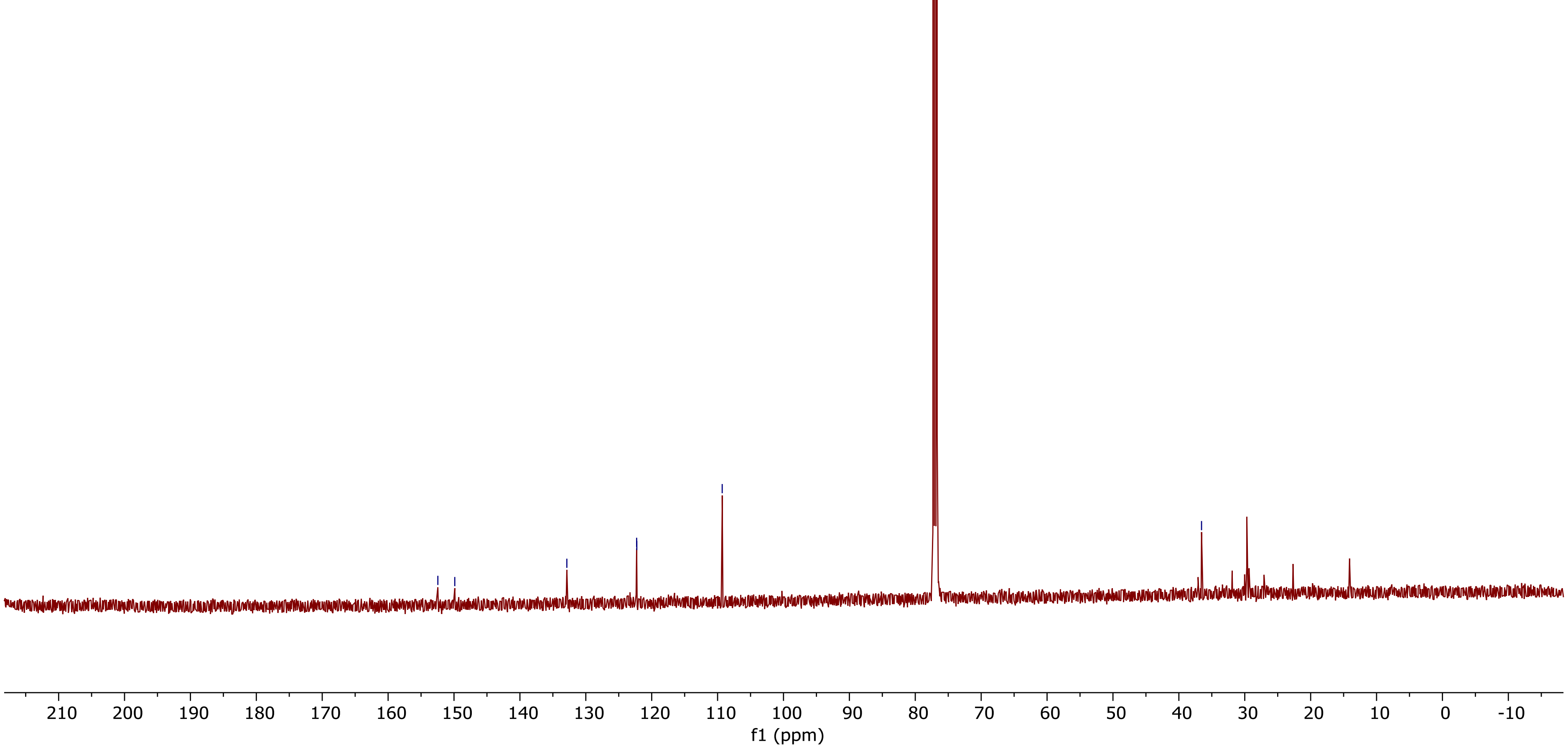

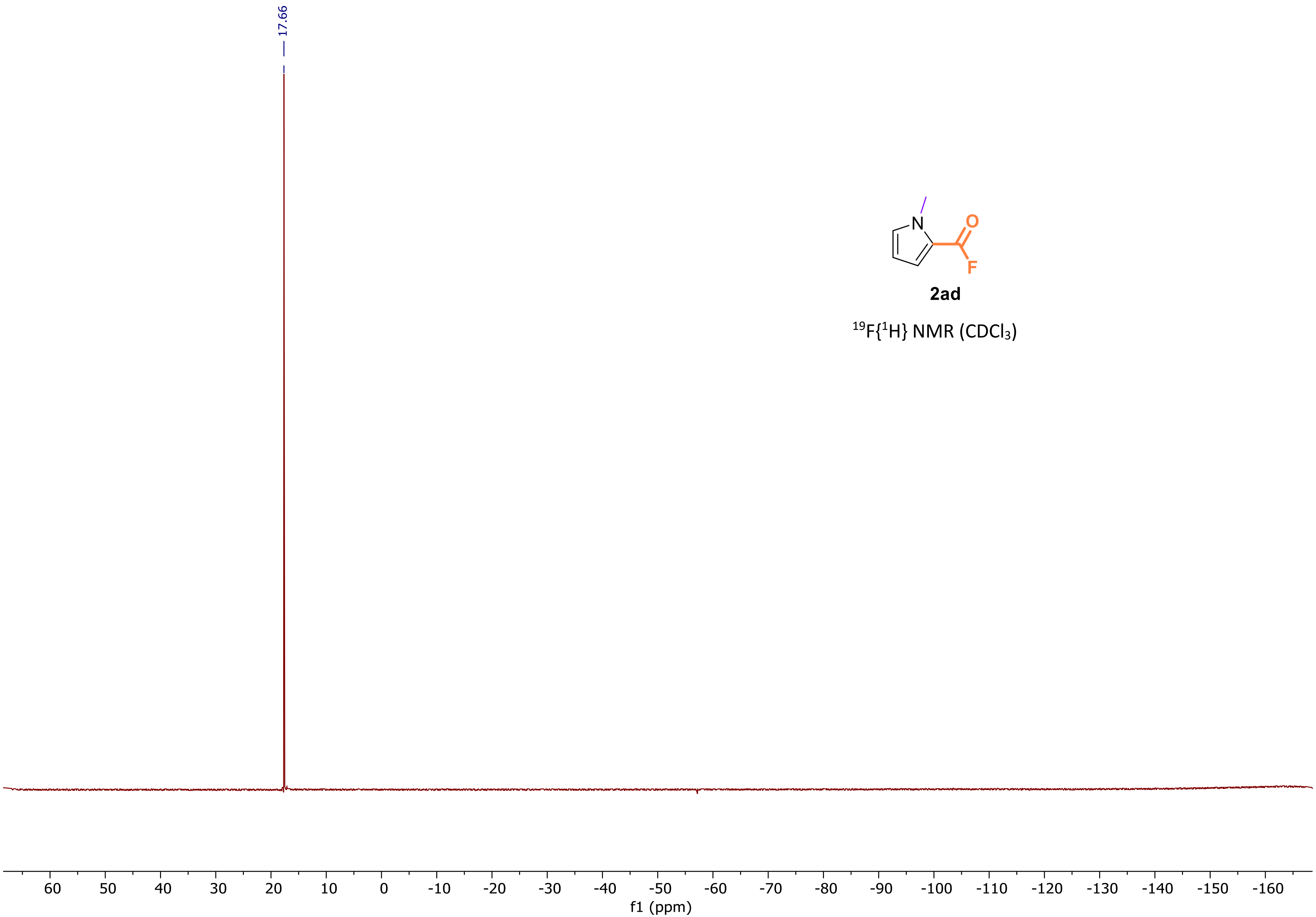

2ad

$^{19}F\{^{1}H\}$ NMR ($CDCl_3$)

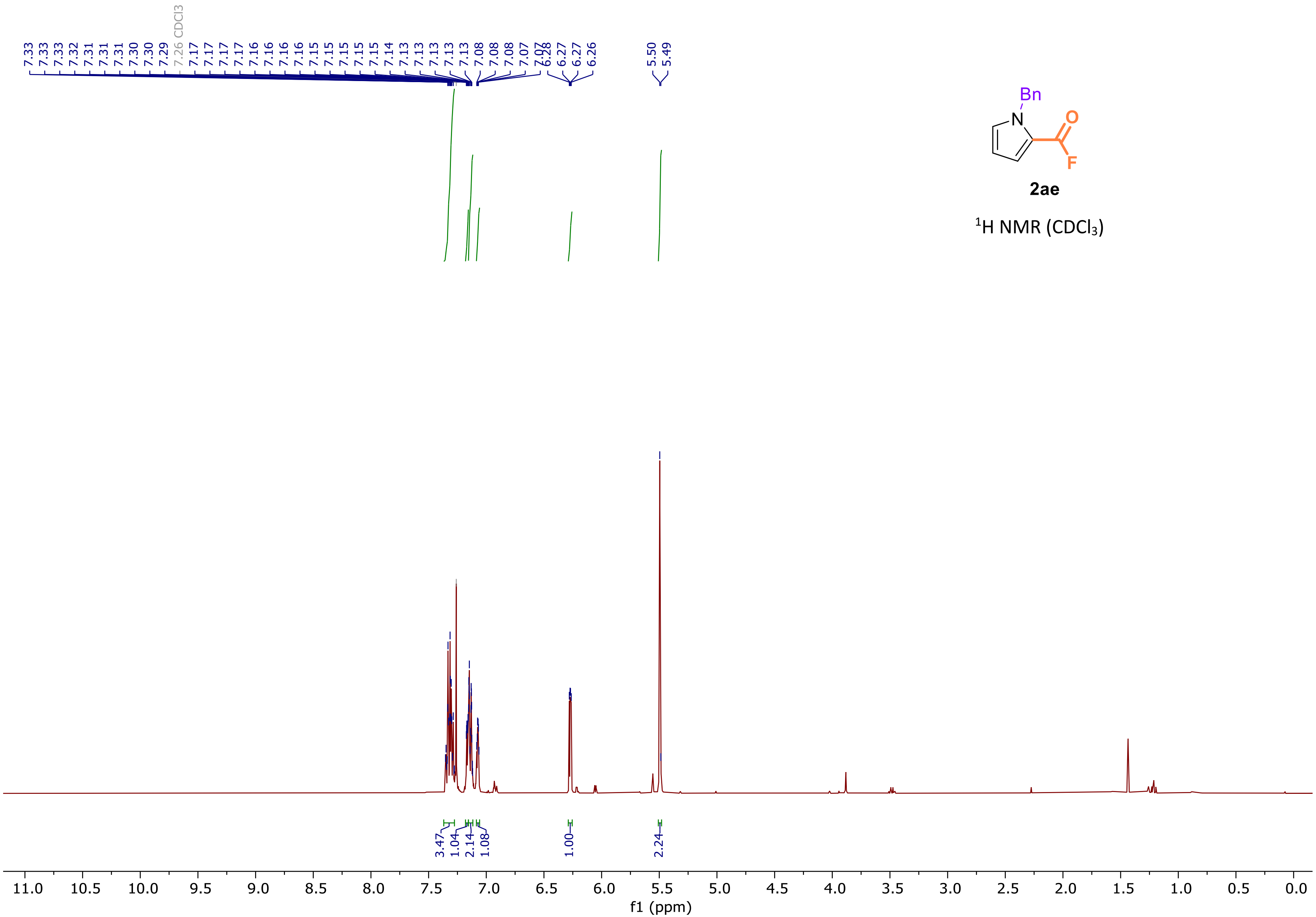

2ae

^{1}H NMR ($CDCl_3$)

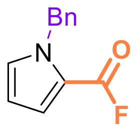

2ae

$^{13}C\{^{1}H\}$ NMR ($CDCl_3$)

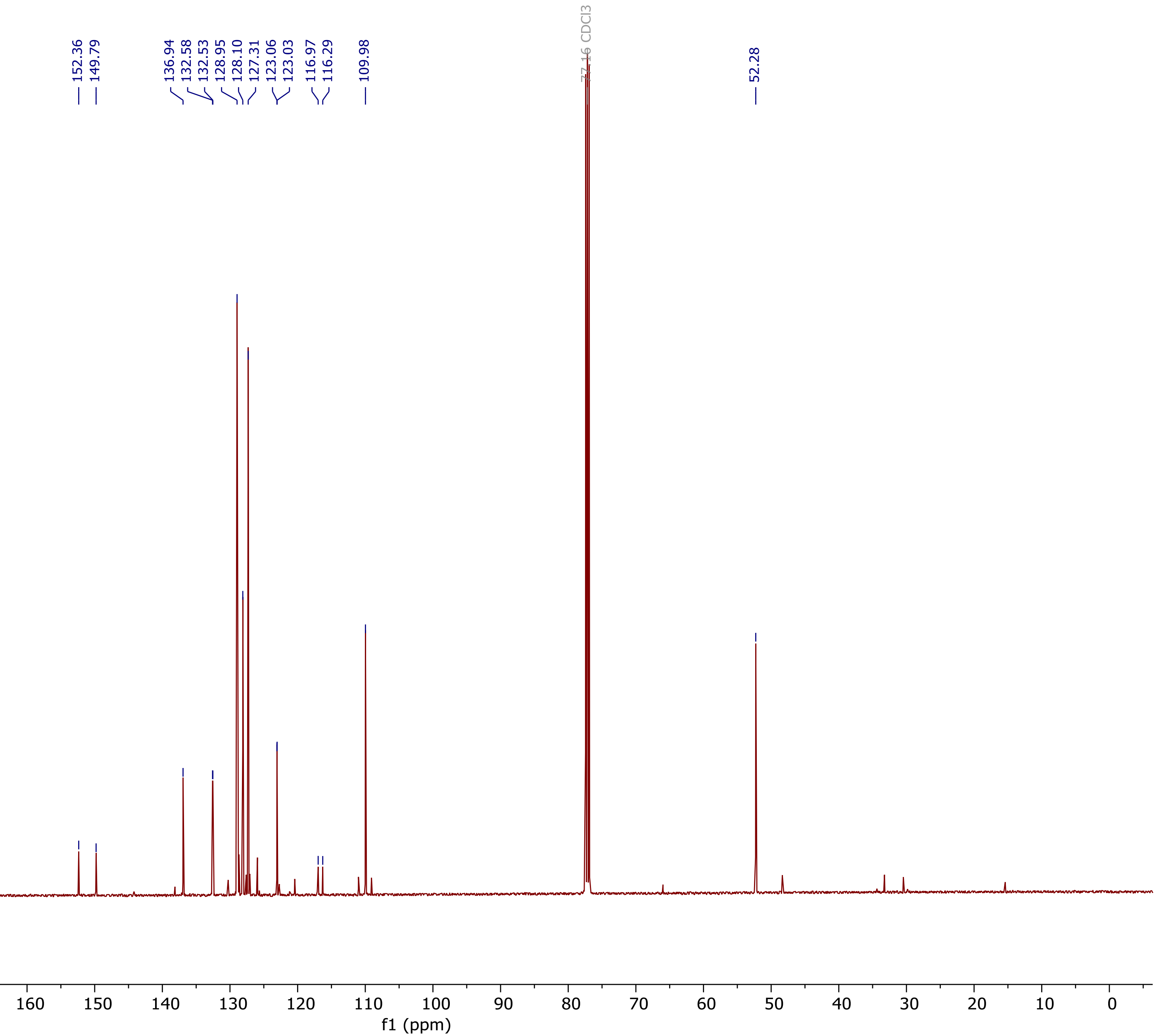

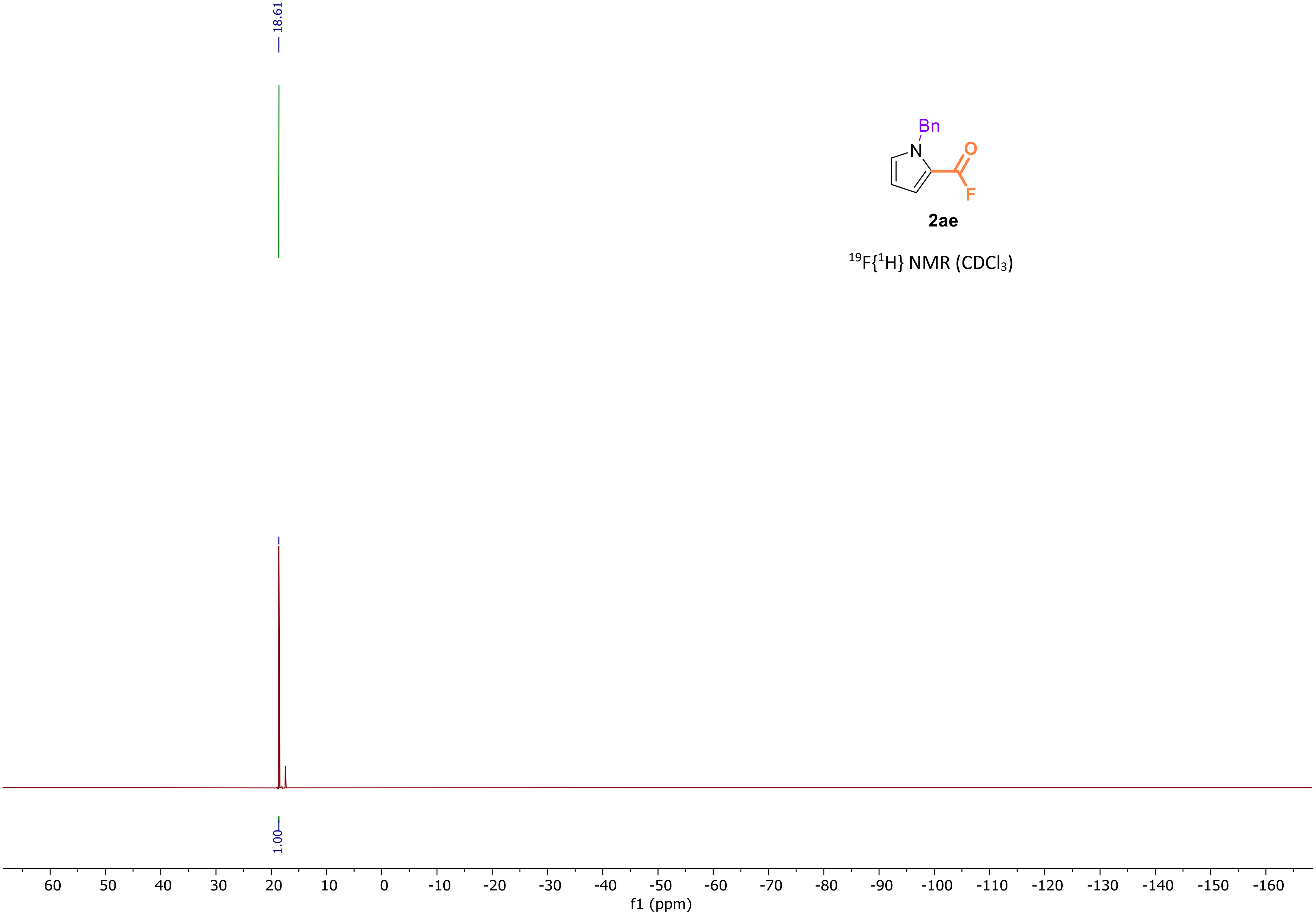

2ae

$^{19}F\{^{1}H\}$ NMR ($CDCl_3$)

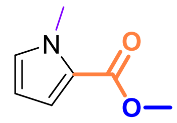

2ad'

^{1}H NMR ($CDCl_3$)

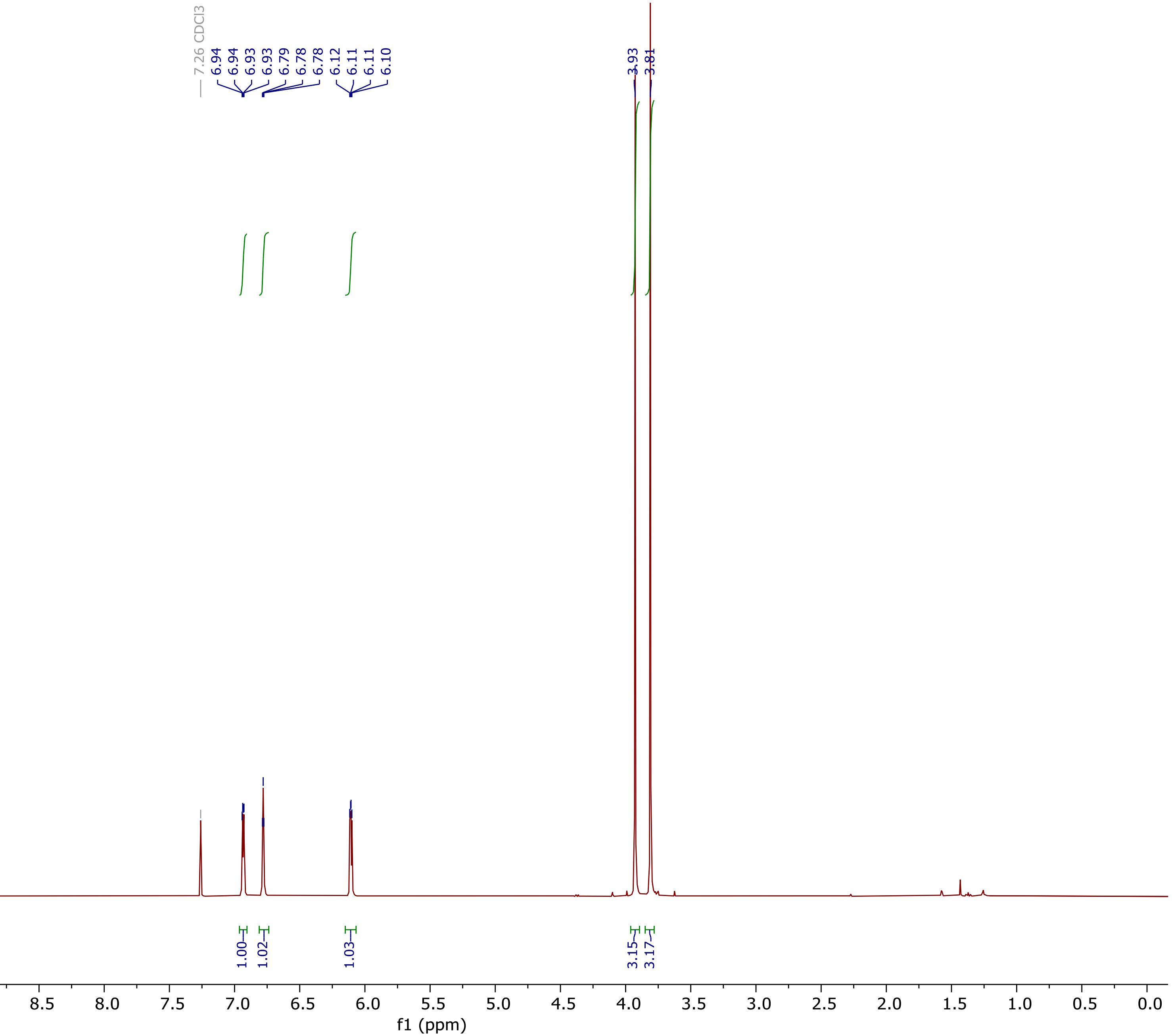

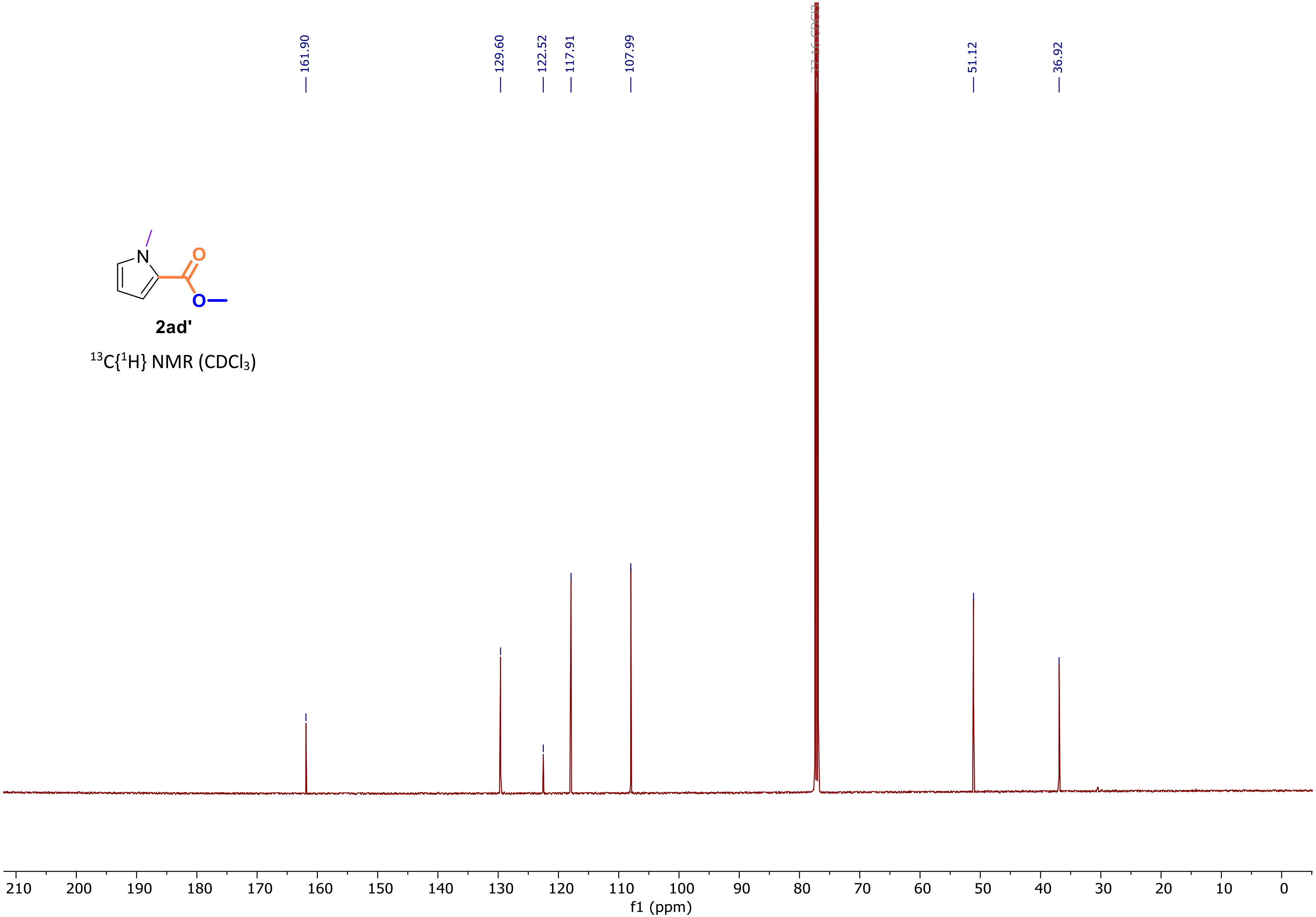

161.90
129.60
122.52
117.91
107.99
77.16 CDCl3
51.12
36.92
2ad'
13C{1H} NMR (CDCl3)
210 200 190 180 170 160 150 140 130 120 110 100 90 80 70 60 50 40 30 20 10 0
f1 (ppm)

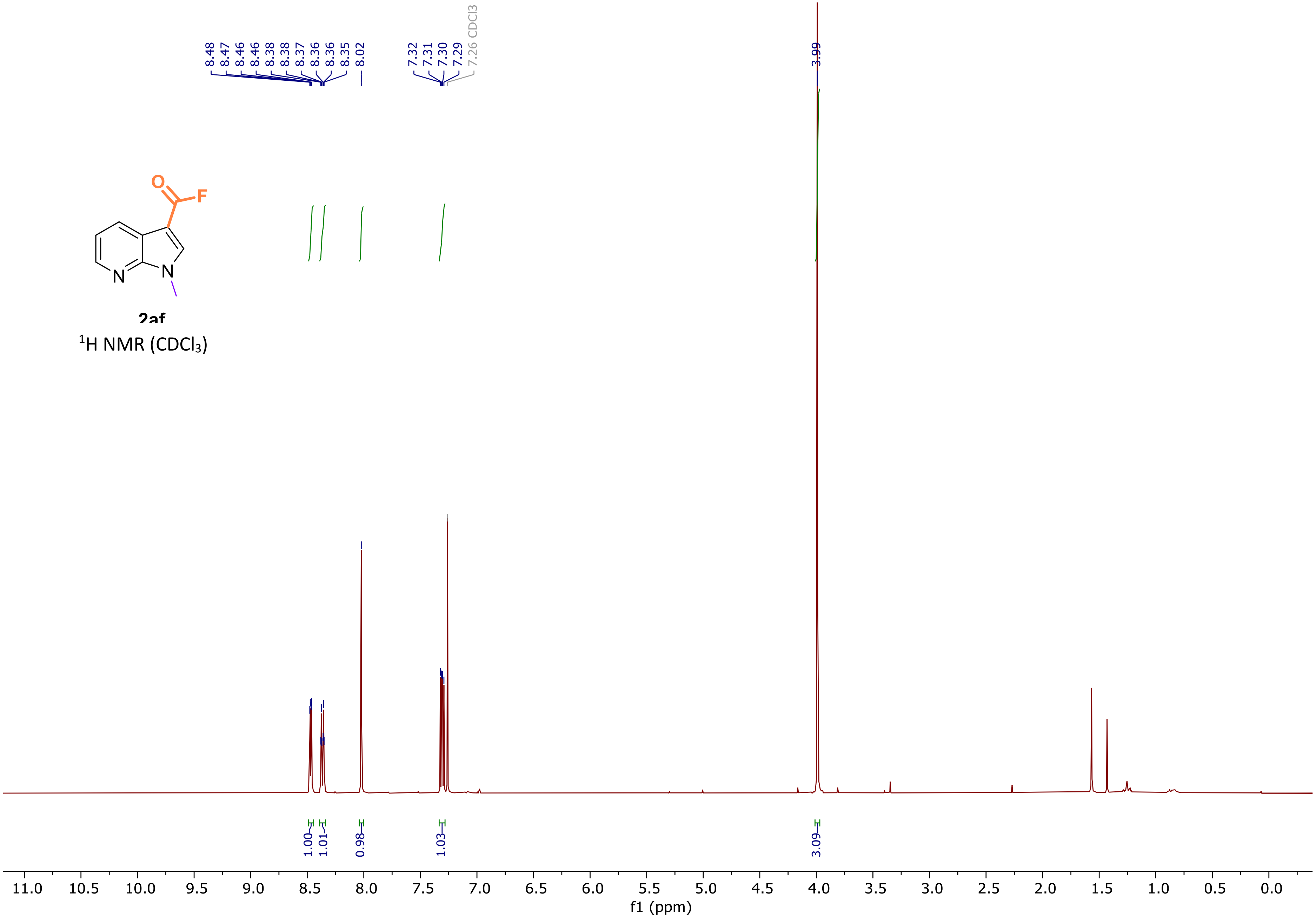

8.48
8.47
8.46
8.46
8.38
8.38
8.37
8.36
8.36
8.35
8.02
7.32
7.31
7.30
7.29
7.26 CDCl3
3.99
O
F
N
N
2af
1H NMR (CDCl3)
1.00
1.01
0.98
1.03
3.09
11.0
10.5
10.0
9.5
9.0
8.5
8.0
7.5
7.0
6.5
6.0
5.5
5.0
4.5
4.0
3.5
3.0
2.5
2.0
1.5
1.0
0.5
0.0
f1 (ppm)

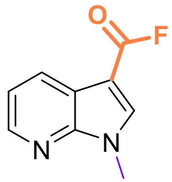

2af

$^{13}C\{^{1}H\}$ NMR ($CDCl_3$)

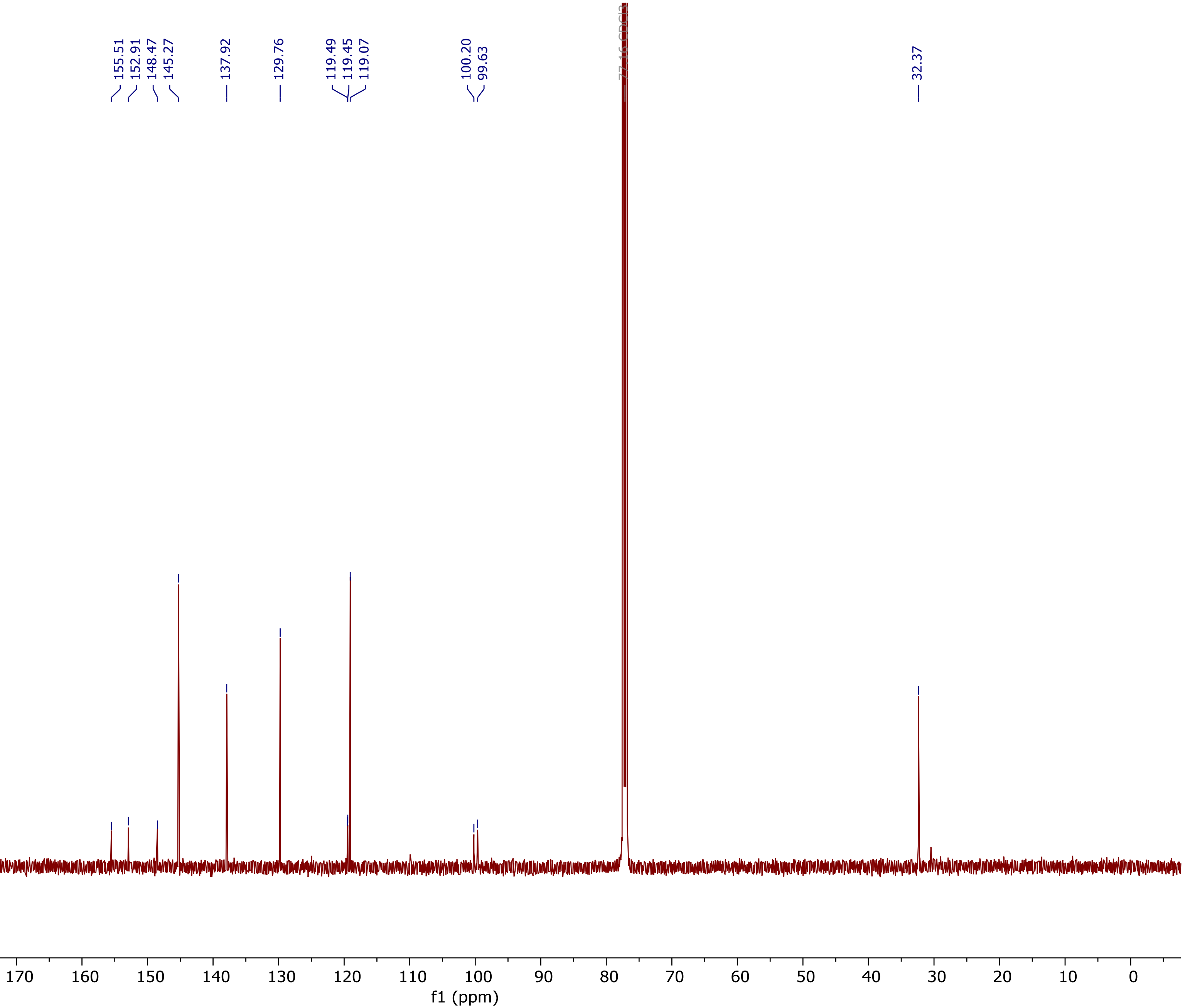

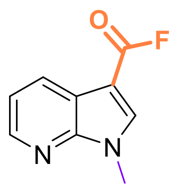

2af

$^{19}F\{^1H\}$ NMR ($CDCl_3$)

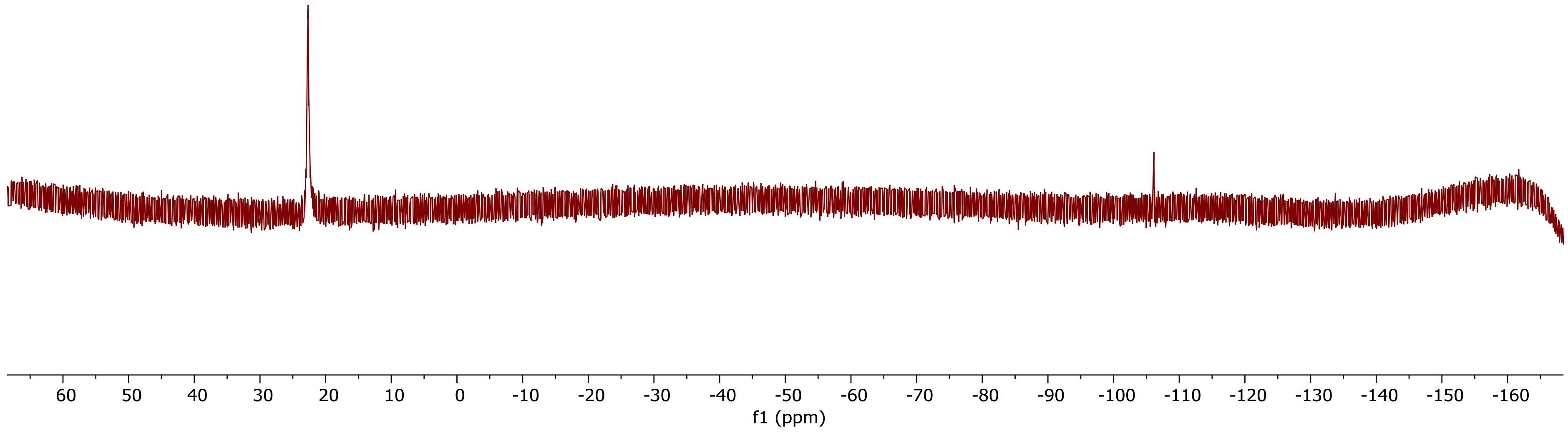

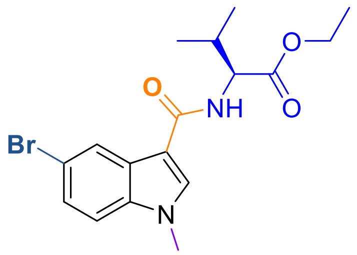

2q1

^1H NMR ($CDCl_3$)

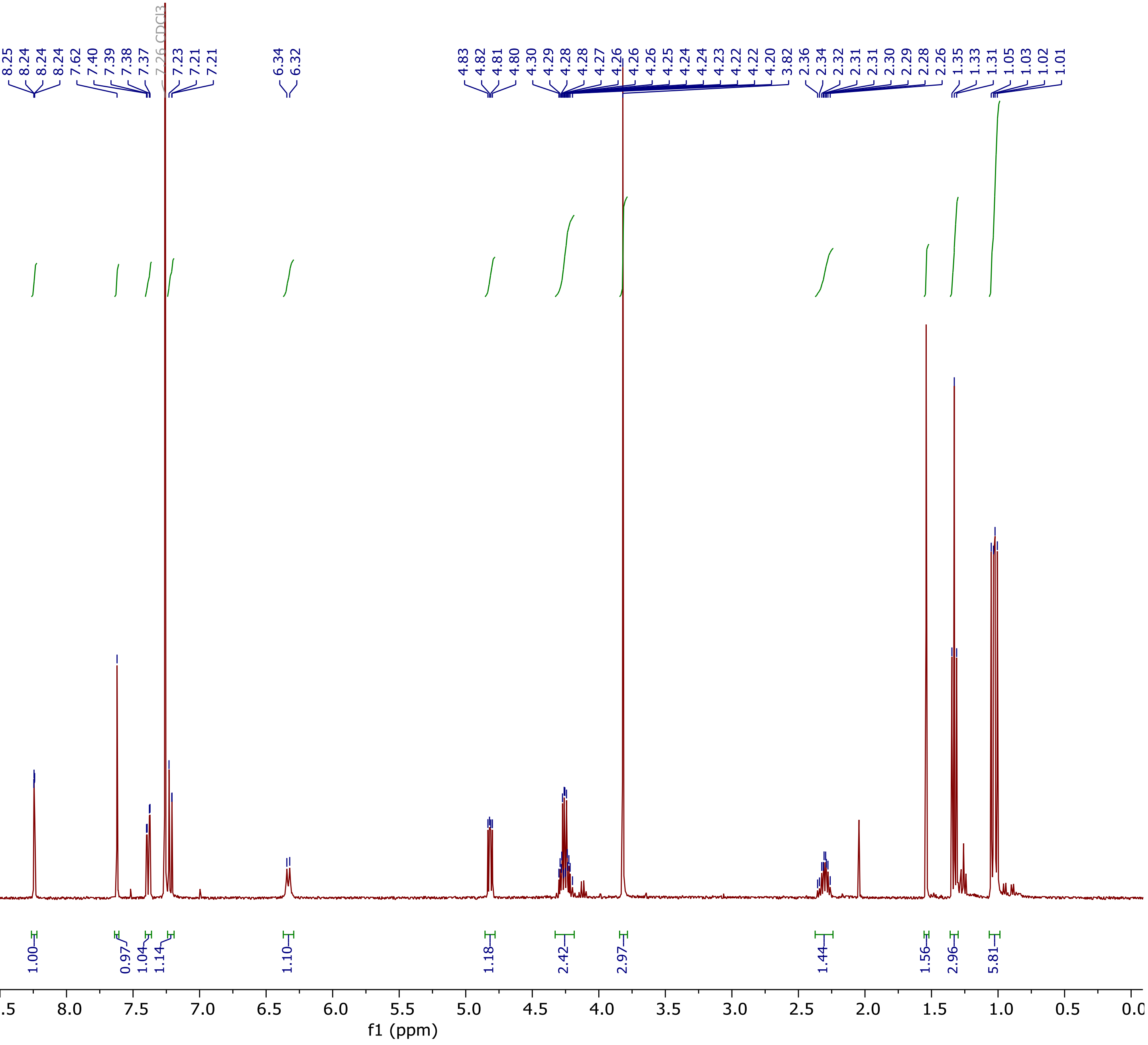

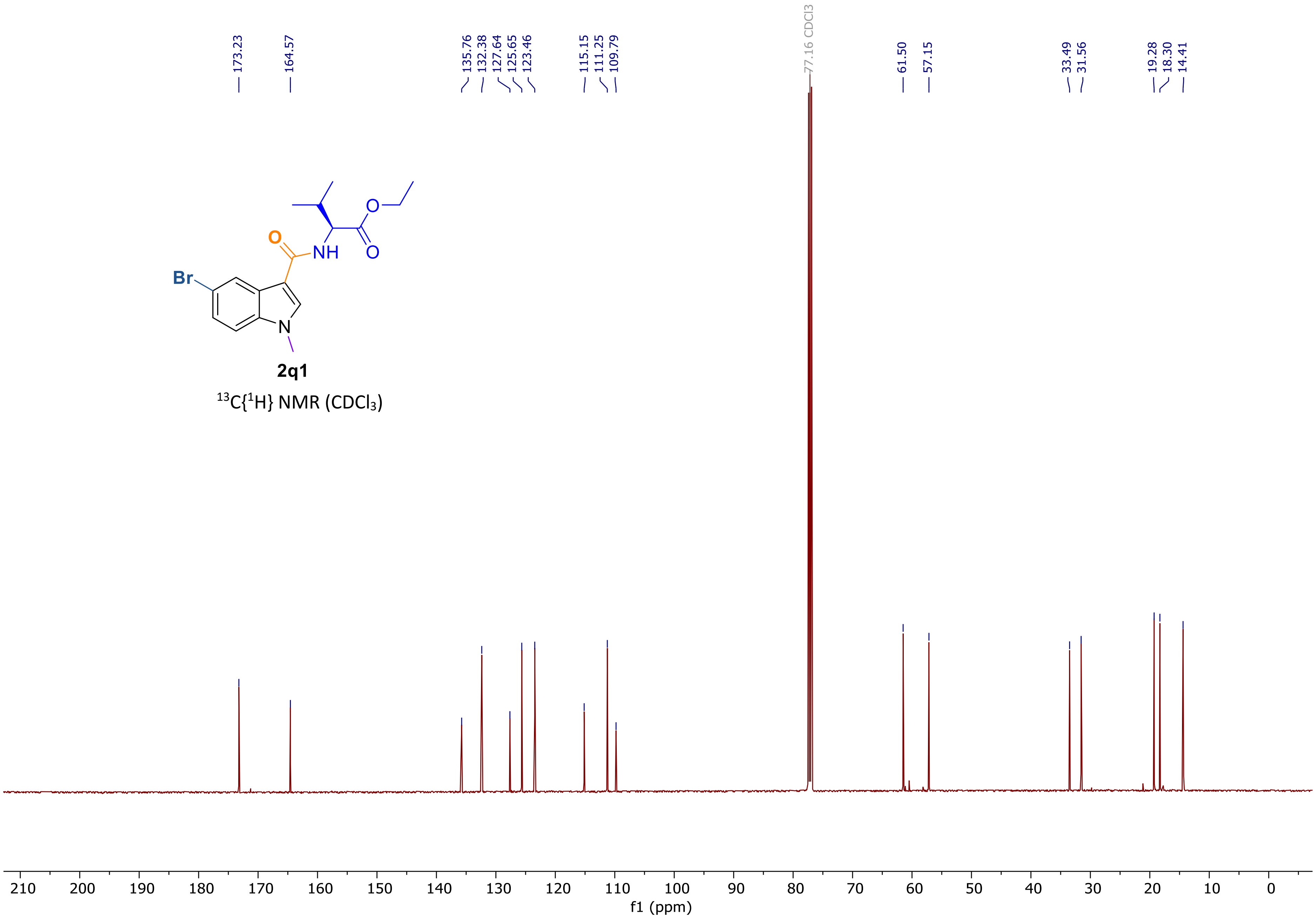

173.23
164.57
135.76
132.38
127.64
125.65
123.46
115.15
111.25
109.79
77.16 CDCl3
61.50
57.15
33.49
31.56
19.28
18.30
14.41
Br
O
NH
O
O
N
2q1
$^{13}C\{^{1}H\}$ NMR ($CDCl_3$)
210 200 190 180 170 160 150 140 130 120 110 100 90 80 70 60 50 40 30 20 10 0
f1 (ppm)

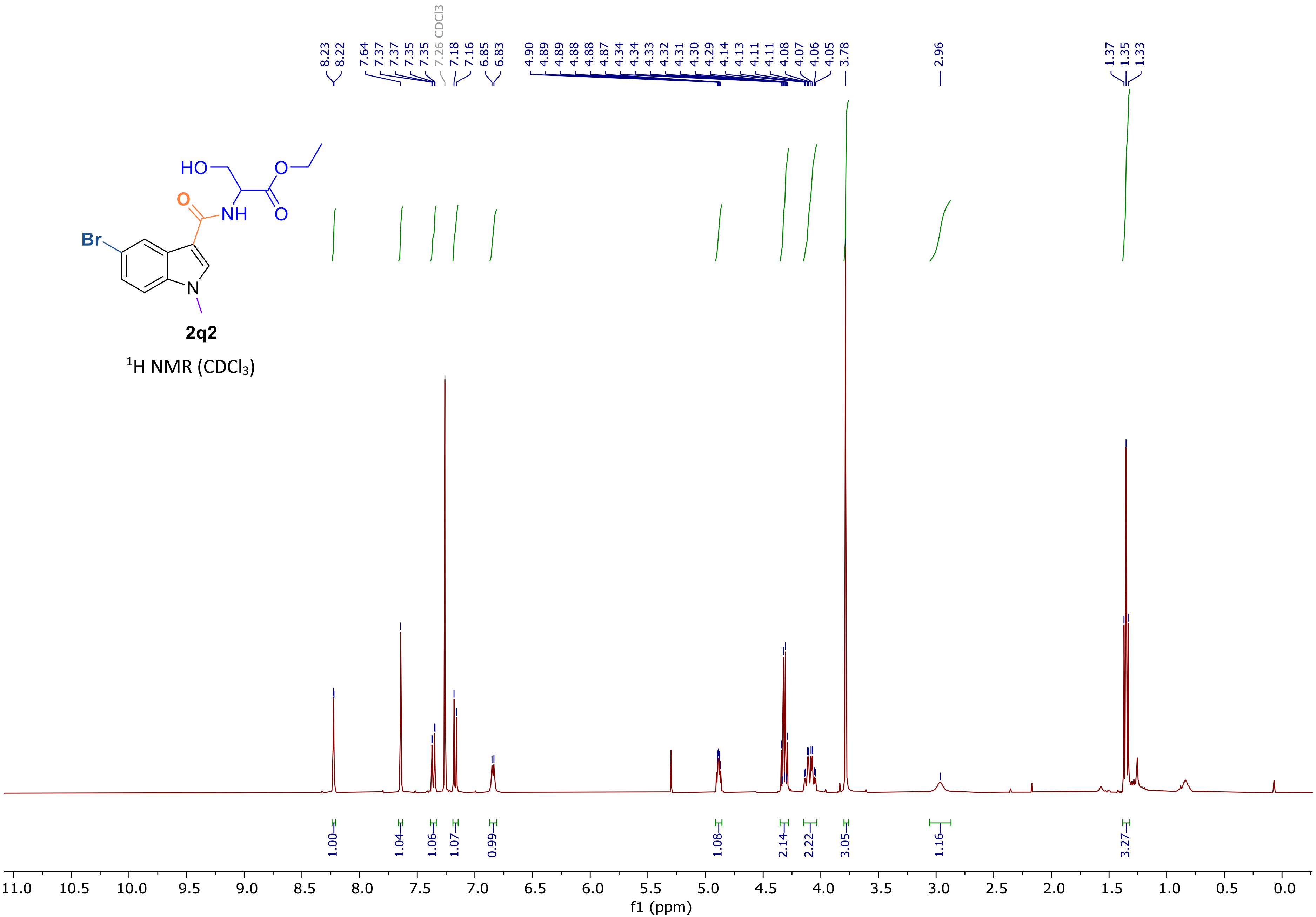
8.23
8.22
7.64
7.37
7.37
7.35
7.35
7.26 CDCl3
7.18
7.16
6.85
6.83
4.90
4.89
4.89
4.88
4.88
4.87
4.34
4.34
4.33
4.32
4.31
4.30
4.29
4.14
4.13
4.11
4.08
4.07
4.06
4.05
3.78
2.96
1.37
1.35
1.33
HO
O
O
NH
O
Br
N
2q2
^{1}H NMR ($CDCl_3$)
1.00
1.04
1.06
1.07
0.99
1.08
2.14
2.22
3.05
1.16
3.27
11.0 10.5 10.0 9.5 9.0 8.5 8.0 7.5 7.0 6.5 6.0 5.5 5.0 4.5 4.0 3.5 3.0 2.5 2.0 1.5 1.0 0.5 0.0
f1 (ppm)

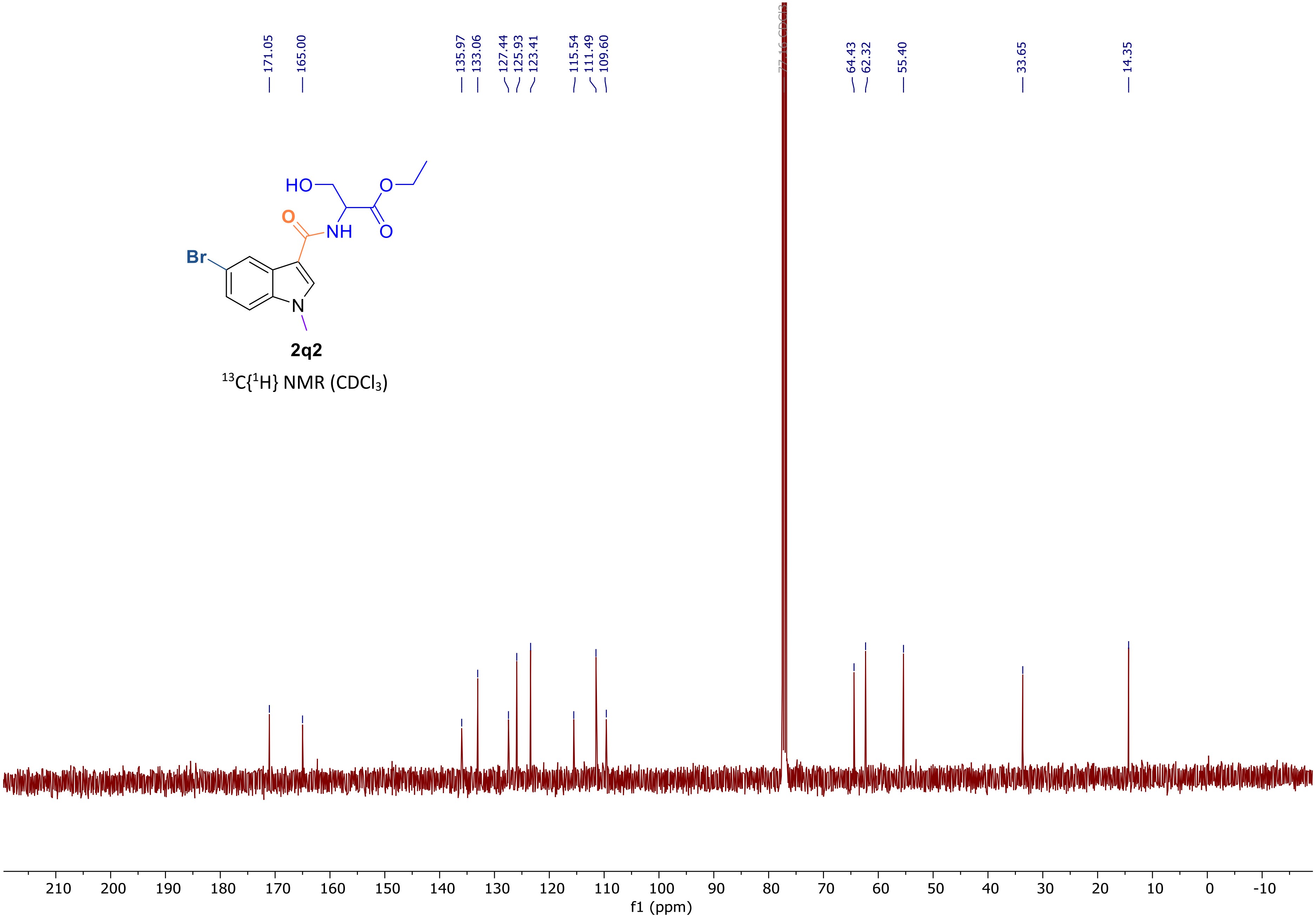
171.05
165.00
135.97
133.06
127.44
125.93
123.41
115.54
111.49
109.60
77.16 CDCl3
64.43
62.32
55.40
33.65
14.35
HO
O
O
NH
O
Br
N
2q2
$^{13}C\{^{1}H\}$ NMR ($CDCl_3$)
210
200
190
180
170
160
150
140
130
120
110
100
90
80
70
60
50
40
30
20
10
0
-10
f1 (ppm)

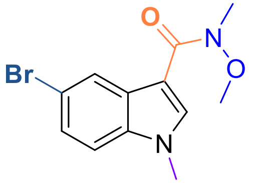

2q3

^{1}H NMR ($CDCl_3$)

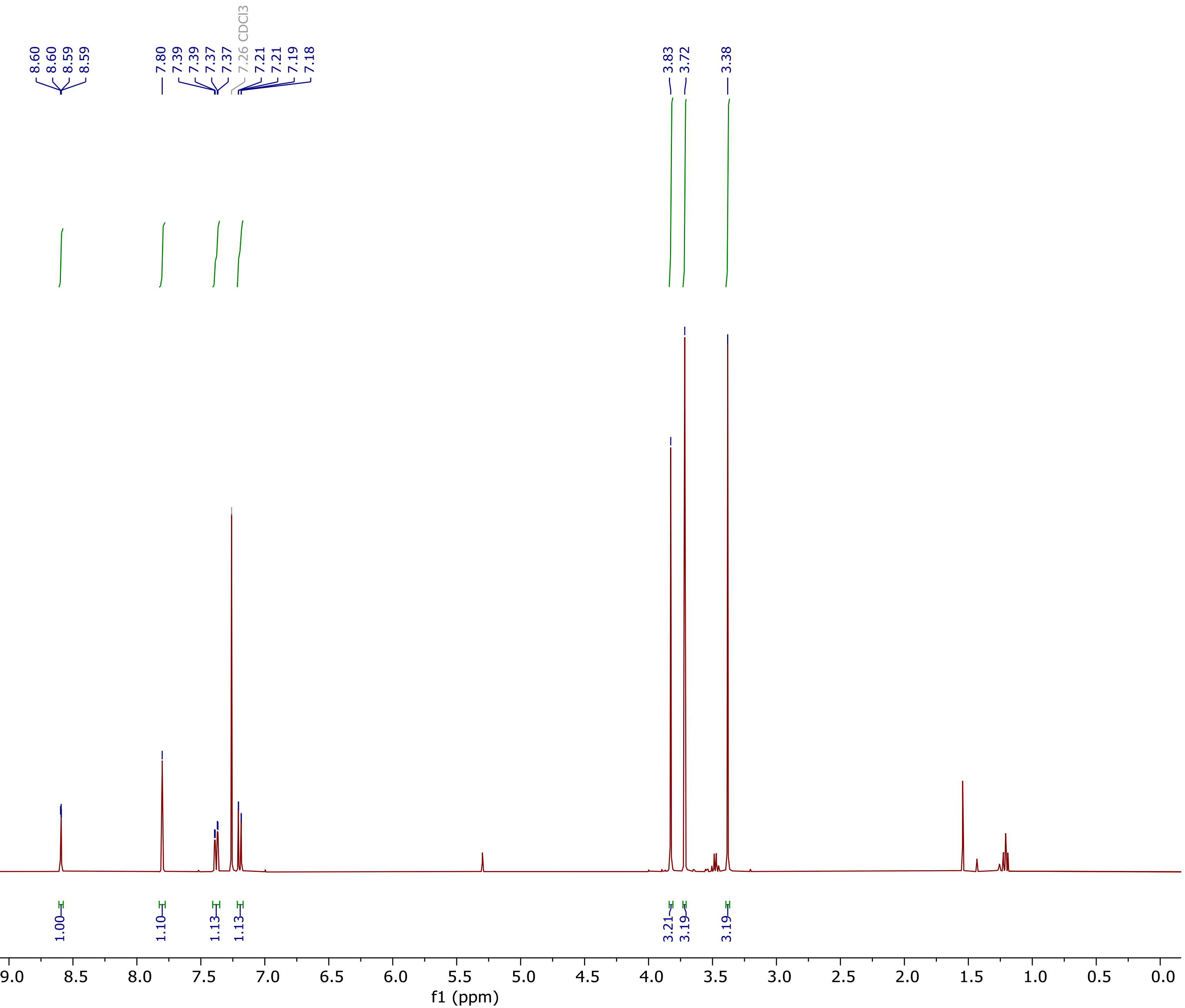

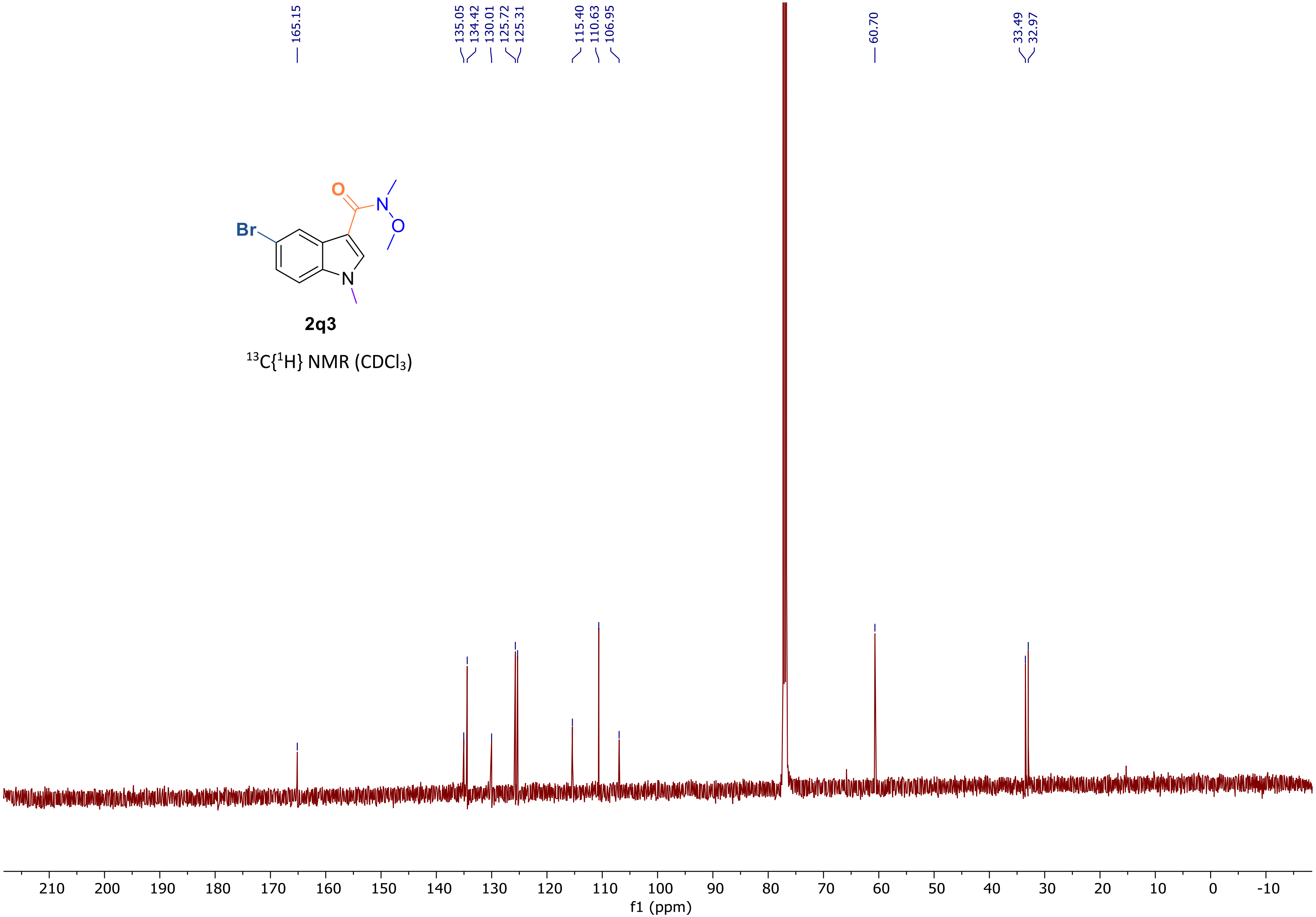

165.15
135.05
134.42
130.01
125.72
125.31
115.40
110.63
106.95
60.70
33.49
32.97
Br
O
N
O
N
2q3
13C{1H} NMR (CDCl3)
210 200 190 180 170 160 150 140 130 120 110 100 90 80 70 60 50 40 30 20 10 0 -10
f1 (ppm)

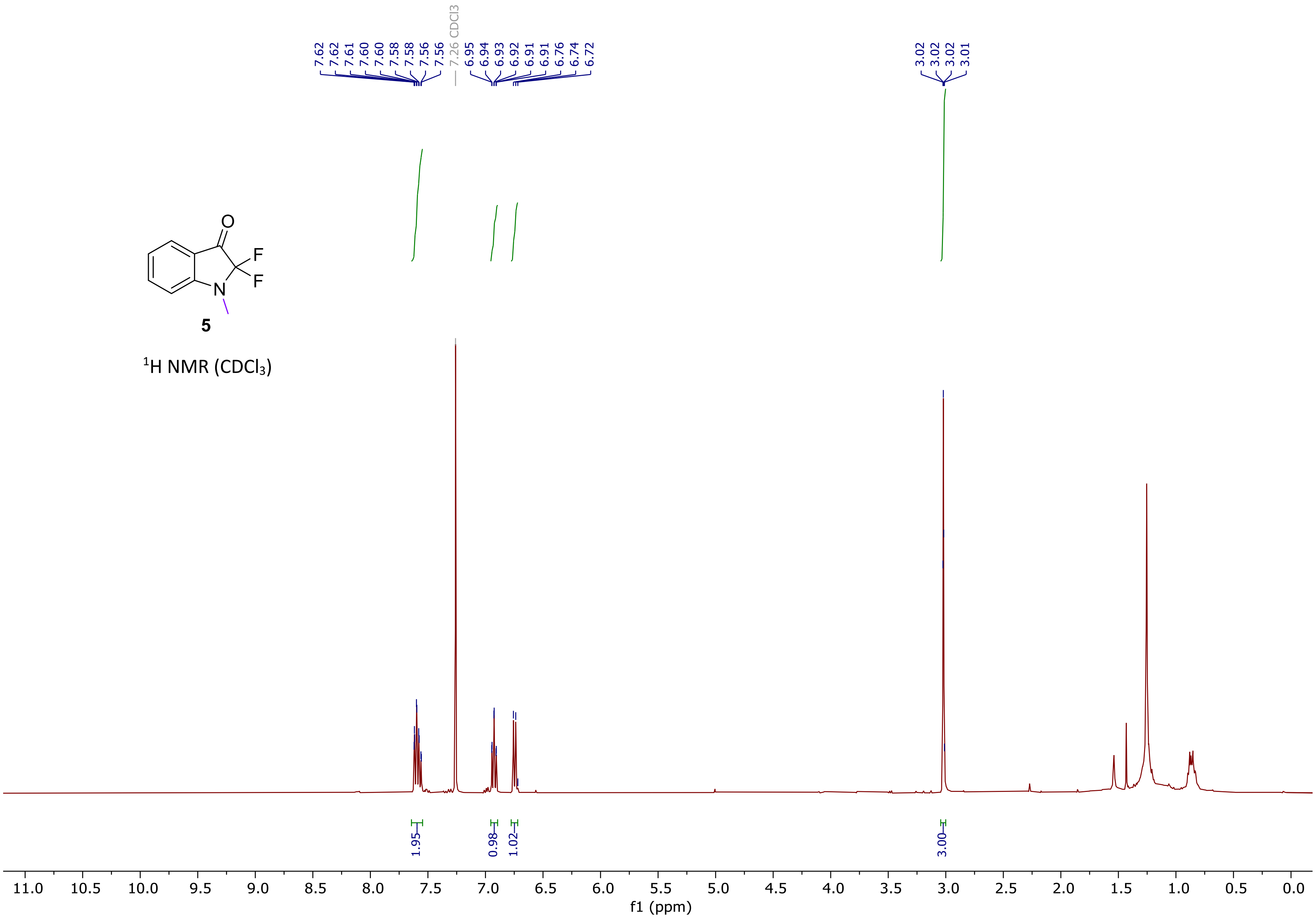
7.62
7.62
7.61
7.60
7.60
7.58
7.58
7.56
7.56
7.26 CDCl3
6.95
6.94
6.93
6.92
6.91
6.91
6.76
6.74
6.72
3.02
3.02
3.02
3.01
O
F
F
N
5
1H NMR (CDCl3)
1.95
0.98
1.02
3.00
11.0
10.5
10.0
9.5
9.0
8.5
8.0
7.5
7.0
6.5
6.0
5.5
5.0
4.5
4.0
3.5
3.0
2.5
2.0
1.5
1.0
0.5
0.0
f1 (ppm)

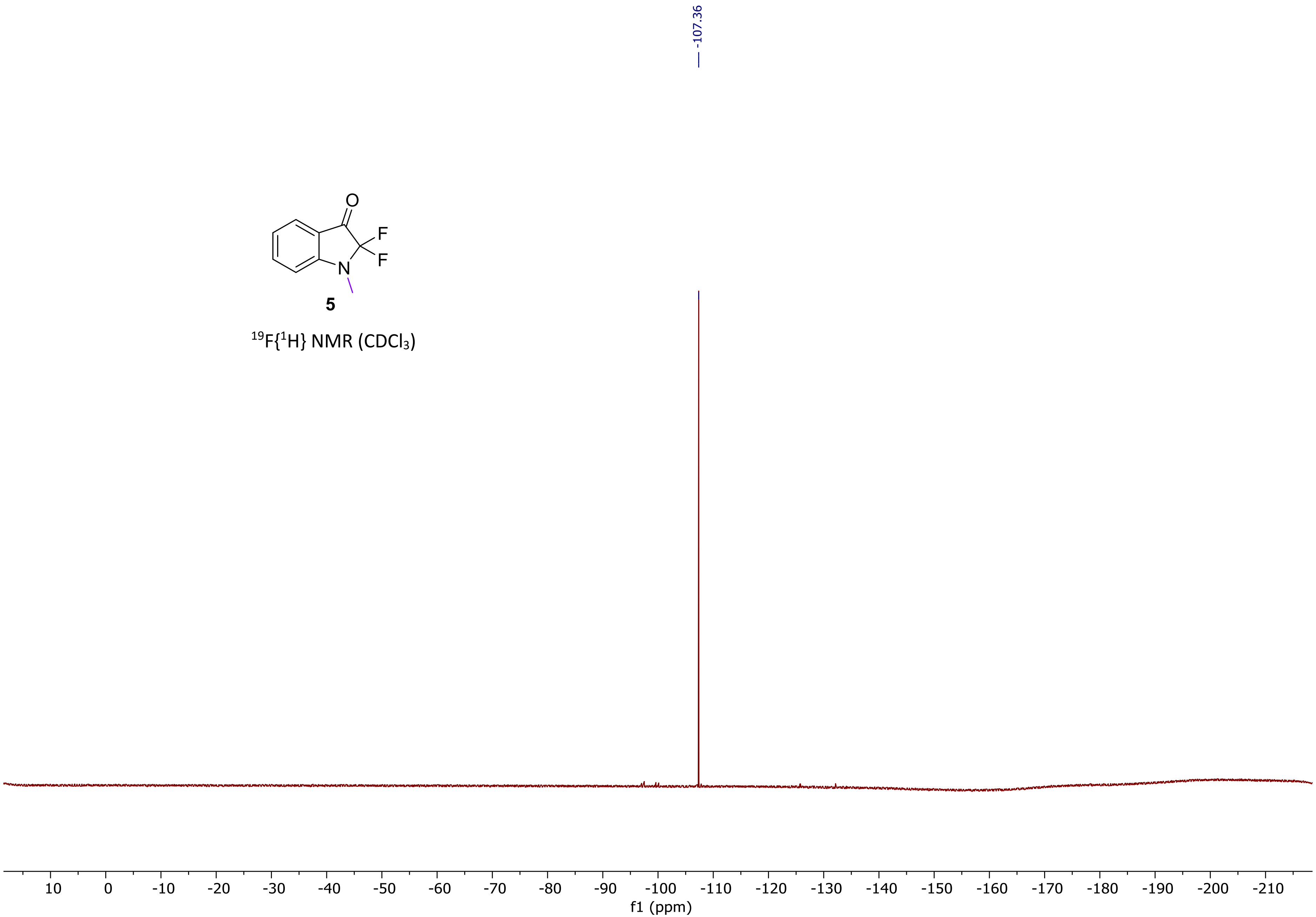

5

$^{19}F\{^{1}H\}$ NMR ($CDCl_3$)

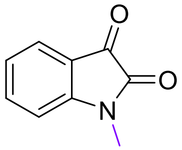

5'

^{1}H NMR ($CDCl_3$)

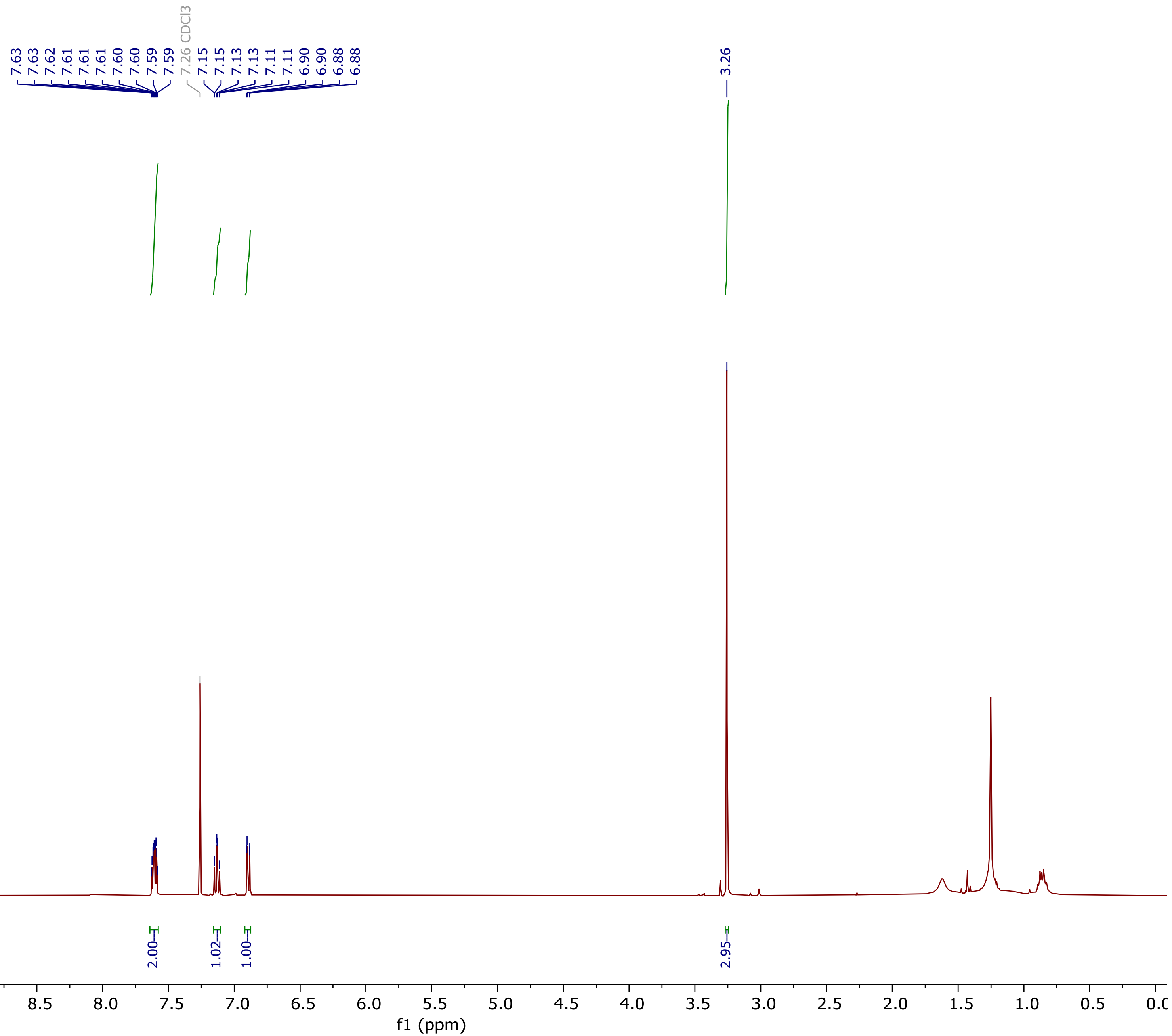

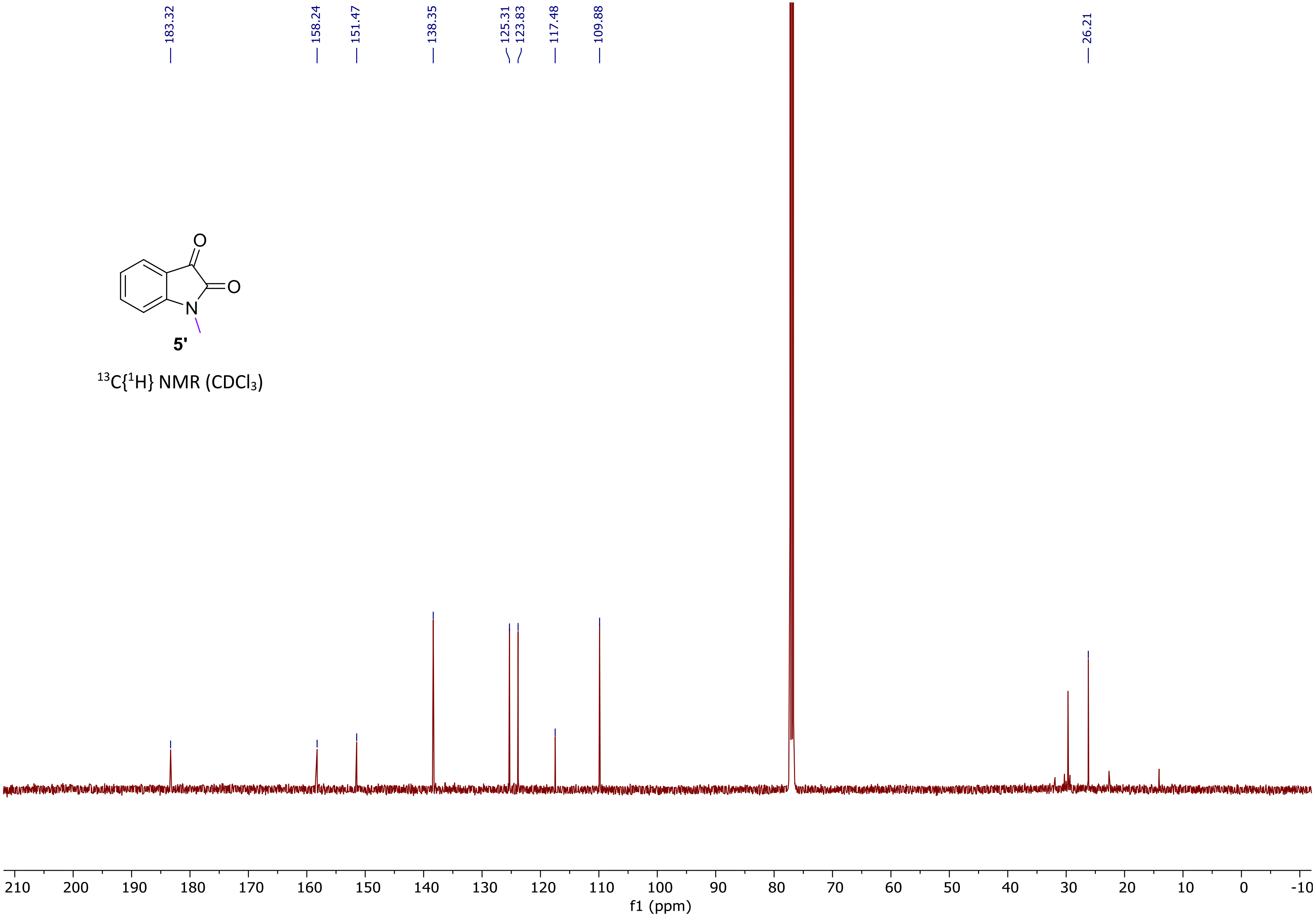
183.32
158.24
151.47
138.35
125.31
123.83
117.48
109.88
26.21
5'
$^{13}C\{^{1}H\}$ NMR ($CDCl_3$)
210 200 190 180 170 160 150 140 130 120 110 100 90 80 70 60 50 40 30 20 10 0 -10
f1 (ppm)
